# A phylogenomic profile of globins

**DOI:** 10.1186/1471-2148-6-31

**Published:** 2006-04-07

**Authors:** Serge N Vinogradov, David Hoogewijs, Xavier Bailly, Raúl Arredondo-Peter, Julian Gough, Sylvia Dewilde, Luc Moens, Jacques R Vanfleteren

**Affiliations:** 1 Department of Biochemistry and Molecular Biology, Wayne State University School of Medicine, Detroit, MI 48201, USA; 2 Department of Biology, Ghent University, B-9000 Ghent, Belgium; 3 Station Biologique de Roscoff, 29680 Roscoff, France; 4 Laboratorio de Biofísica y Biología Molecular, Facultad de Ciencias, Universidad Autónoma del Estado de Morelos, 62210 Cuernavaca, Morelos, México; 5 RIKEN Genomic Sciences Centre, Yokohama 230-0045, Japan; 6 Department of Biomedical Sciences, University of Antwerp, 2610 Antwerp, Belgium

## Abstract

**Background:**

Globins occur in all three kingdoms of life: they can be classified into single-domain globins and chimeric globins. The latter comprise the flavohemoglobins with a C-terminal FAD-binding domain and the gene-regulating globin coupled sensors, with variable C-terminal domains. The single-domain globins encompass sequences related to chimeric globins and «truncated» hemoglobins with a 2-over-2 instead of the canonical 3-over-3 α-helical fold.

**Results:**

A census of globins in 26 archaeal, 245 bacterial and 49 eukaryote genomes was carried out. Only ~25% of archaea have globins, including globin coupled sensors, related single domain globins and 2-over-2 globins. From one to seven globins per genome were found in ~65% of the bacterial genomes: the presence and number of globins are positively correlated with genome size. Globins appear to be mostly absent in Bacteroidetes/Chlorobi, Chlamydia, Lactobacillales, Mollicutes, Rickettsiales, Pastorellales and Spirochaetes. Single domain globins occur in metazoans and flavohemoglobins are found in fungi, diplomonads and mycetozoans. Although red algae have single domain globins, including 2-over-2 globins, the green algae and ciliates have only 2-over-2 globins. Plants have symbiotic and nonsymbiotic single domain hemoglobins and 2-over-2 hemoglobins. Over 90% of eukaryotes have globins: the nematode *Caenorhabditis *has the most putative globins, ~33. No globins occur in the parasitic, unicellular eukaryotes such as *Encephalitozoon, Entamoeba, Plasmodium *and *Trypanosoma*.

**Conclusion:**

Although Bacteria have all three types of globins, Archaeado not have flavohemoglobins and Eukaryotes lack globin coupled sensors. Since the hemoglobins in organisms other than animals are enzymes or sensors, it is likely that the evolution of an oxygen transport function accompanied the emergence of multicellular animals.

## Background

The number of globin families has grown markedly since the 1970's, when they were limited to the α- and β-globins and Mbs, mostly of vertebrates, and the SHbs (legHbs) of legume plants [1-3]. The ensuing years brought to light several new globins: SHbs in plants other than legumes, NsHbs in a wide variety of plants [4-6], and FHbs, chimeric proteins (~400aa) comprising an N-terminal globin domain and a C-terminal FAD- and NAD-binding domain, related to the ferredoxin-NADP^+ ^reductases, found in *E. coli *[7] and in yeasts [8,9]. Concurrently, "truncated" globins, sequences shorter than normal (<130aa), were discovered in protozoa [10], cyanobacteria [11-13], a nemertean [14], and bacteria [15,16]. Globins longer than normal (>160aa), similar to the "truncated" Hbs, were observed in a green alga [17,18] and in plants [19]. The crystal structures of several "truncated" Hbs showed them to have a novel 2-over-2 α-helical fold instead of the canonical 3-over-3 α-helical fold, with an abbreviated A helix, a decreased CE interhelical region and most of the F helix occurring as a loop [20-22]. Consequently, we advocate using 2/2 Hb instead of "truncated", to indicate the distinctive secondary structure of this group of globins and to bring order to the chaotic terminology in the existing databases, where "truncated", "cyanobacterial", "protozoan" and "2-over-2" are all in current use.

The utilization of molecular biological techniques allowed the detection of globins in organisms where their presence was unsuspected, such as the nematode *Caenorhabditis elegans *and in other nematodes [23-30], the dipteran *Drosophila melanogaster *[31] and the urochordate *Ciona intestinalis *[32]. The recent, rapid accumulation of genomic information has resulted in a substantial increase in newly recognized globins. This list includes the Ngbs [33,34] and Cygbs [35-37], which are believed to occur in all vertebrates from humans to birds and fish [38], GbE, the eye-specific globin in the domestic chicken *Gallus gallus*, related to Cygb [39], and GbX, a new and fifth type of globin gene in fish and amphibians, thought to have been lost in the higher vertebrates [40]. FHbs were found in a variety of bacterial groups [16,41-43] together with SDgbs, which align with the FHb globin domains [42,43]. Furthermore, globin coupled sensor proteins, chimeric two-domain gene regulators (~300 to >700aa) comprising an N-terminal globin domain, were discovered in an archaean and several bacterial groups [44-47]. Lastly, "protoglobins" (~195aa) related to the former, were found in archaea and in several bacteria [48] and proposed to represent the "ancestral" globin.

## Results

### Overview

**Figure 1:**
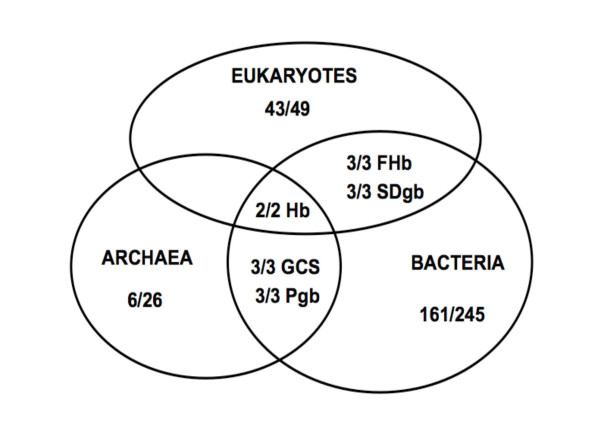
A Venn diagram of the distribution in the three kingdoms of life, of the three globin lineages: 3/3 FHbs/SDgbs, 3/3 GCSs/Pgbs and 2/2 Hbs. The number of genomes that have globins is shown as a fraction of the total number of genomes.

**Figure 2:**
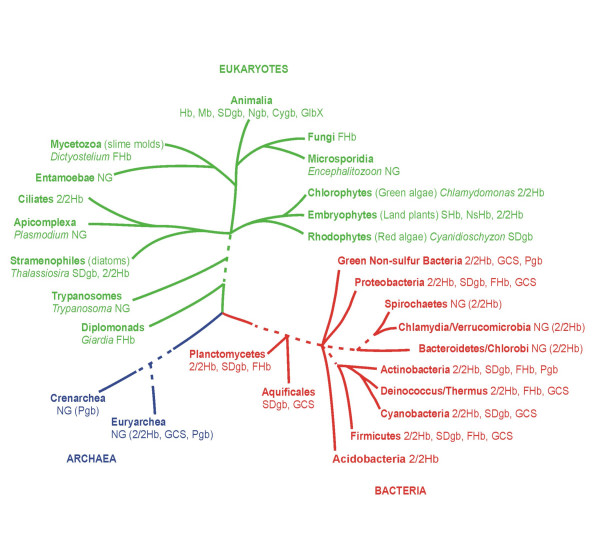
A diagrammatic representation of the three kingdoms of life and the phylogenetic relationships of their major groups based on Baldauf et al. [50], and depicting the distribution of globins.

The 245 bacterial, 26 archaeal and 49 eukaryote genomes, and the identified known and putative globins, are listed in Supplemental Data Tables 1–6 [see Additional File 1]. An alignment of the sequences is provided in Supplemental Data Fig. 1 [see Additional File 2]. All the globins identified in our survey can be assigned to two general classes: single chain globins and globin domains within chimeric proteins. The former group comprises the vertebrate α- and β-globins, Mbs, Ngbs and Cygbs, the metazoan intra- and extracellular Hbs (including multi-domain and/or multisubunit Hbs), the plant SHbs and NsHbs, the ~195aa Pgbs and the 2/2 Hbs. The two groups of chimeric proteins, which generally have an N-terminal globin domain, are the ~400aa FHbs with an NAD and FAD-binding C-terminal domain, and the GCSs with highly variable (~50 to >700a) C-terminal domains. Thus, all globins belong to one of three globin lineages: the 3/3 FHbs and SDgbs (eukaryote and bacterial), the 3/3 GCSs and Pgbs and the 2/2 SDgbs [49]. Fig. 1 provides the number of genomes that have globins and illustrates a key result: only one of the three globin lineages, the 2/2 Hbs, are represented in all three kingdoms of life. Fig. 2 shows the approximate phylogenetic relationships between the main groups of the three kingdoms of life based on Baldauf et al. [50] and depicts the phylogenomic profile of globins.

It should be emphasized, that the presence of putative globins inferred using SUPERFAMILY and blastp and psiblast searches of the GenBank database, does not require having a completed genome. On the other hand, the absence of a globin is certain only when a complete genome is searched and no sequence is found.

### Archaea

Table 2 in the Supporting Data [see Additional File 1] lists the archaeal genomes, their sizes and the presence of globins. SUPERFAMILY identified putative globins in four Archaea: *Halobacterium*, *Methanobacterium*, *Methanosarcina *and *Sulfolobus*. In addition, another globin was identified in the SWISSPROT/TrEMBL database (tr|Q96ZL6). Examination of these sequences showed that they are not likely to be globins due to lack of size (< 100aa) and/or the absence of a suitable F8His. However, Hou *et al*. [44.45,48] have recently discovered GCSs and Pgbs in a couple of archaea, and we found a 2/2 Hb1 in *Haloarcula marismortui *[49]. This survey has uncovered a 2/2 Hb1 in still another euryarcheote halophile *Haloferax volcanii*. Thus, only five of the 20 Euryarcheote genomes have globins: *Haloarcula marismortui *(2/2 Hb1, GCS)*, Halobacterium salinarum *(GCS)*, Haloferax volcanii *(2/2 Hb1, GCS)*, Methanosarcina acetivorans *and *M. barkeri *(Pgbs). Of the 5 Crenarcheote genomes, only *Aeropyrum pernix*, has a globin (Pgb) and the only Nanoarcheote genome has no globins. The paucity of globins in Archaea is one of the surprising results of our survey. Except for *Aeropyrum pernix*, the archaeal genomes comprising globins are, coincidentally, among the largest known archaeal genomes. It is interesting to note that none of the GCSs or Pgbs have been identified by the hidden Markov chain model used by SUPERFAM, although they align readily with the globin fold, based on the recent crystal structure of *Bacillus subtilis *GCS [51]. Most of the archaeal genomes sequenced to date have been those of extremophilic organisms with small genomes. It is now known that there are many more non-thermophilic terrestrial and marine archaea than hitherto suspected [52]. It remains to be seen whether additional archaeal genomes will reverse the situation.

**Table 1:** Number of bacterial genomes with one or more classes of. 2-over-2 Hbs

Class 1	Class 2	Class 3	Class 1+2	Class 1+3	Class 2+3
17	47	18	14	1	18

**Table 2:** Coexistence of FHbs with SDgbs in bacterial genomes.

FHb only	SDgb only	FHb+SDgb
65	18	6

### Bacteria

**Figure 3:**
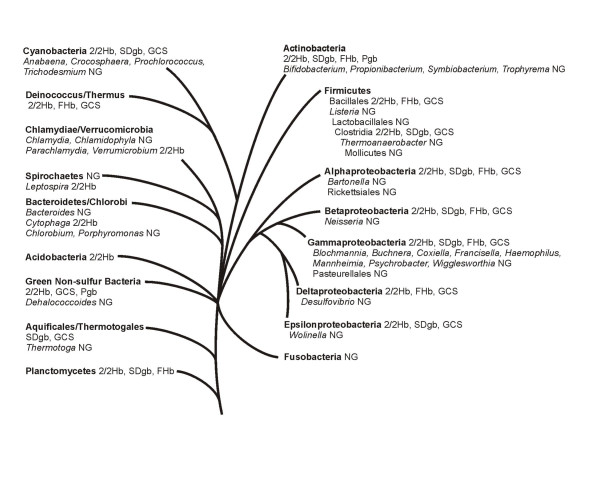
A diagrammatic representation of the phylogenetic relationships between the major bacterial groups based on Baldauf et al.[50], and showing the distribution of globins.

Table 3 in Supporting Data [see Additional File 1] lists the completed and unfinished bacterial genomes used in this study, their sizes, the type and number of globins present, 2/2 Hbs (classes 1, 2 and 3), FHbs (SDgbs) and GCSs (Pgbs), and the oxygen requirement and habitat of the organism. A diagrammatic representation of the phylogenetic relationships between the main bacterial groups based on Baldauf *et al*. [50], shown in Fig. 3, summarizes the globin distribution among the bacteria. The one representative of the Acidobacteria has globins, and only 4 of the 26 genomes representing the Actinomycetes have none: *Bifidobacterium, Propionibacterium, Symbiobacterium *and *Trophyrema*. The 3 Aquificales/Thermotogales have globins except *Thermotoga*, and the 5 Bacteroidetes/Chlorobi (except *Cytophaga*) lack globins, as well as the 6 Chlamydiae/Verrumicrobium genomes, except *Parachlamydia *and *Verrumicrobium*. Of the 3 Chloroflexi genomes, *Dehalococcoides*, has no globins. The Cyanobacteria are represented by 12 genomes, of which 5 lack globins: *Anabaena, Crocosphaera, Prochlorococcus, Synechocccus elongatus *and *Trichodesmium*. Both *Deinococcus *and *Thermus *have globins. Among the 49 Firmicutes, apart from the Lactobacillales and the Mollicutes, the 17 Bacilli have globins (except *Listeria*), as well as 4 of the 7 Clostridia. Although *Fusobacterium *lacks globins, the lone Nitrospirae and the two Planctomycetes have globins. The majority of the 35 genomes of Alphaproteobacteria have globins: all the Caulobacterales, Rhizobiales (except *Bartonella*), Rhodobacterales, Rhodospirilalles and Sphingomonadales, but only one of the 10 Rickettsiales (*Pelagibacter ubique*). All 24 genomes representing the Betaproteobacteria have globins, except 2 of the 4 Neisseriaceae. The 50 Gammaproteobacteria genomes represent 10 groups, of which only 3 of the 10 Enterobacteriales (*Buchnera*, *Blochmannia *and *Wigglesworthia*), one of two Legionellales, the 5 Pasteurellales, the two *Psychrobacter *of the 10 Pseudomonadales, and the one Thiotrichales (*Francisella*) are devoid of globins. The 10 genomes of Deltaproteobacteria have globins, except for *Desulfovibrio*. Two of the 8 genomes representing the Epsilonproteobacteria have no globins: *Helicobacter pylorii *and *Wolinella*; however, *Helicobacter hepaticus *has a globin. The lone unassigned proteobacterium *Magnetococcus *has a GCS. Of the 128 genomes representing the Proteobacteria, 99 have globins (~75%).

**Table 3:** Coexistence of FHbs/SDgbs with 2-over-2 Hbs in bacterial genomes.

FHb or SDgb only	With class 1	With class 2	With class 3	With class 1+2	With class 1+3	With class 2+3
26	4	29	7	2	0	13

Alignment of bacterial 2/2 Hb sequences indicates that they can be divided into three separate classes [16,53]. The length of the 2/2 Hbs varies from 118aa for the cyanobacterium *Nostoc *(ZP_00112318) to 260aa in *Streptomyces avermitilis *(NP_824492), with the majority (>60) being <140aa. Examination of coexistence of the three classes of 2/2 Hbs (Table 1) shows an interesting trend: although only the Alphaproteobacterium *Hyphomonas neptunium *has both 1 and 3 and two have all three classes, 14 genomes have classes 1 and 2 and 18 have classes 2 and 3. These results suggest that the class 1 and 3 2/2 Hbs are derived from the class 2 2/2 Hbs, as pointed out very recently by Vuletich and Lecomte [53].

The finding of an FHb in *E. coli *[7] led to the discovery of some 30 bacterial FHbs [16,42,43,54]; currently, the number is over 70 (Supplementary Data Table 3). R. Poole and his group [43] and Frey and Kallio [42] were the first to point out the similarity between the FHb globin domains, the first bacterial Hb to be sequenced, that of *Vitreoscilla stercoraria *[55], and several other SD bacterial globins. We now have a total of 25 SDgbs in 22 genomes. The crystal structures of the *P. aeruginosa *(PDB: 1tu9) [56] and *Vitreoscilla stercoraria *(PDB: 2vhb) [57], show them to be very similar to the globin domains of the FHbs from *E. coli *(PDB: 1gvh) [58] and *Ralstonia (Alcaligenes) eutropha *(PDB: 1cqx) (59]. It is appropriate to note here that *Ralstonia *has undergone two name alterations recently, to *Wautersia eutropha *and now to *Cupriavidus necato*r [60]. Although 22 genomes have SDgbs, three of them have two different globins: *Bradyrhizobium, Rhodopseudomonas *and *Novosphingobium*. Table 2 shows that FHbs and SDgbs coexist in only 6 genomes: *Chromobacterium, Photobacterium, Pseudomonas aeruginosa, Rhodopirellula, Thermobifida *and *Vibrio parahaemolyticus*. Table 3 shows the statistics of occurrence of 2/2 Hbs with the FHbs/SDgbs; the latter tend to occur with class 2 and with both class 2 and 3 2/2 Hbs.

M. Alam and his group have identified 27 GCSs [46,47] and 3 Pgbs from bacteria – *Chloroflexus aurantiacus *ZP_00359040 (227aa), *Thermobifida fusca *ZP_00293478 (197aa) and *Thermosynechococcus elongatus *NP_682779 (194aa) [48]. Blastp searches found an additional two dozen GCSs, and Pgbs in *Thermus thermophilus *YP_005074 (203aa) and the actinobacterium *Rubrobacter xylanophilus *ZP_00200180 (196aa) [49,61]. The recent crystal structure of *B. subtilis *GCS shows it to have a 3/3 fold [62].

The genomes with the largest number of globins, 5 to 7, are all from the Alpha- and Betaproteobacteria; they do include however, redundant sequences. The following have all three lineages of globins:*Burkholderia fungorum *(9.67 Mbp)*, Chromobacterium violaceum *(4.75 Mbp)*, Novosphingobium aromaticivorans *(4.21 Mbp) and *Silicibacter *sp.TM1040 (4.14 Mbp), each with 5 globins, the three *Bordetella *species (4.09–5.34 Mbp), *Exiguobacterum sp.255–1*5 (2.89 Mbp), *Shewanella baltica *(5.01 Mbp)*, Sinorhizobium meliloti *and *Thermobifida fusca *(3.64 Mbp), each with 4 globins, and *Azotobacter vinelandii *(5.42 Mbp), all the *Bacillus *species (except *B. licheniformis *and *B. stearothermophilus*) (4.20–5.50 Mbp) and *Geobacillus kaustophilus *(3.59 Mbp), each with 3 globins. At the other end of the scale, the smallest genome (1.31 Mbp) to have a globin, a 2/2 Hb1, is that of the marine Alphaproteobacterium *Pelagibacter ubiques *(Rickettsiales), followed by *Aquifex aeolicus *(1.59 Mbp), and the Epsilonbacterium *Campylobacter jejuni *(1.64 Mbp), with one (SDgb) and two (2/2Hb and SDgb) globins, respectively.

The bacterial divisions represented by 10 or more genomes, rank in the following order of having globins: Betaproteobacteria – 92% (22/24) > Actinobacteria – 85% (22/26) > Gammaproteobacteria – 76% (38/50) > Alphaproteobacteria – 69% (24/35) > Cyanobacteria – 58% (7/12) > Firmicutes – 49% (19/39). Overall, 161 of the 245 (~65%) bacterial genomes have globins and the number of globins varies from 1 to 7.

**Figure 4:**
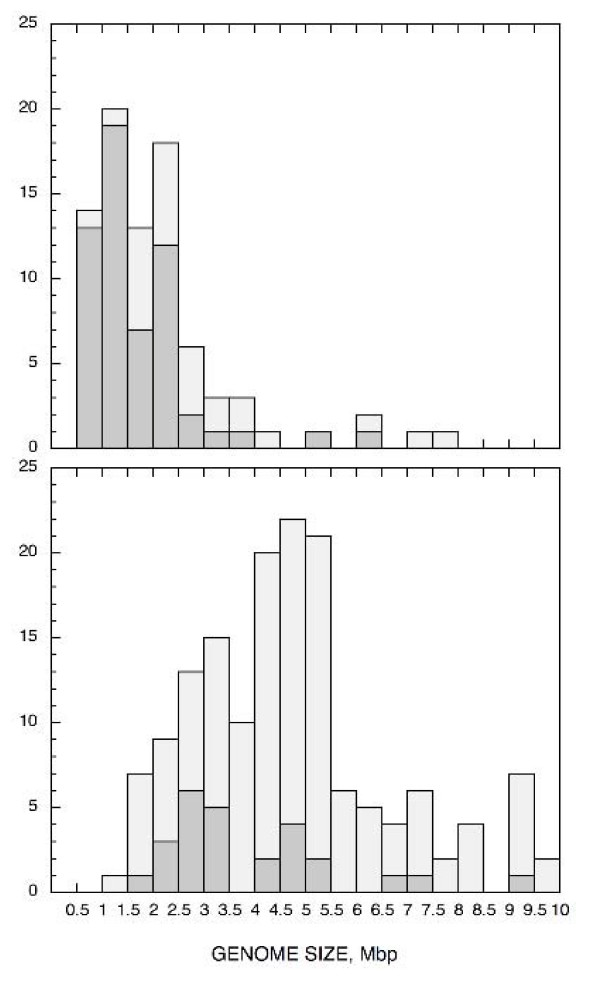
The statistics of bacterial genome size distribution: lacking globins (upper panel) and containing globins (lower panel). The genomes that are host-associated (Table 3 in Supplementary Material) are represented by stippled bars.

Fig. 4 depicts the size distribution of bacterial genomes lacking globins (upper panel) and comprising globins (lower panel); the stippled columns represent the genomes associated with a host (given in Supplemental Data Table 3 [see Additional File 1]). Although the two distributions overlap, it is evident that presence of globins is correlated with genome size and that the majority of genomes lacking globins are host associated. Table 4 shows the mean genome size for completed genomes lacking globin and with one or more globins. The mean size of the genomes shows a positive correlation with the number of globins present. About 80% (65/82) of genomes <2.5 Mbp lack globins (mean 2.1 Mbp). Very small genomes are characteristic of bacteria with lifestyles characterized by continuous association with a host [63]. The majority of the bacteria whose genomes have been sequenced, are human, animal and plant infectious agents. Thus, it is likely that the estimate of approximately two thirds of all bacteria having globins obtained in the present study, is on the low side.

**Table 4:** Number of globins found in bacterial genomes and the corresponding mean genome sizes.

No. of globins	No of completed genomes	Mean genome size, Mbp
0	75	1.9 ± 1.1
1	40	3.9 ± 1.5
2	19	4.2 ± 1.2
3	22	4.7 ± 1.2
4	11	6.5 ± 1.9

### Eukaryotes

The globins identified in eukaryote genomes are listed in Supplemental Data Tables 4–6 [see Additional File 1]. The globins listed for the vertebrate genomes, *Danio (Brachydanio) rerio, Fugu (Takifugu) rubripes, Gallus gallus, Homo sapiens, Mus musculus, Rattus norvegicus *and *Xenopus tropicalis*, include the familiar α- and β-globins and Mb, as well as the recently discovered Ngbs [33,34] and Cygbs [35-37]. A recent addition to human α-globins is μ-globin (gi|51510893|NP_001003938, 141aa) which appears to be very similar to the avian α-D globin [64]. In addition, an eye-specific globin, GbE, was found in *Gallus gallus *[39] and a new globin, GbX, was discovered in the fish, bird and amphibian genomes [40]. Curiously, no Mb-like sequence was found in either *Xenopus tropicalis *or *X. laevis*. The zebrafish *Danio rerio *has 6 embryonic globins [65], three α and three β- only 5 are listed in the GenBank. The pufferfish *Fugu rubripes*, appears to have four α-globin chains [66]; only three of them are listed in the GenBank.

The four globins found in the genome of the urochordate *Ciona intestinalis *(sea squirt), have been identified earlier [32]. This important finding corrects the erroneous claim of total absence of globins in the report of the genome [67].

At least one putative globin (gi|72169631|XP_795670, 175aa) was found in the recently completed genome assembly of the echinoderm *Strongylocentrotus purpuratus *(sea urchin). Used as query in blastp searches it hits the GlbXs and Cygbs of fish, and other vertebrate Cygbs, before recognizing the coelomic globin (gi|729576;P80018) of a fellow echinoderm, the holothurian *Caudina arenicola*.

There appear to be 4 putative globins in *Anopheles gambiae*: gi|55246163| EAA03862.3 (150aa), gi|31201271|XP_309583 (192aa), gi|31198253|XP_308074 (215aa) and gi|57914327|XP_555006 (182aa). Their alignment shows that the first three are closely related, sharing essentially identical globin domains: gi|55246163 has an 18aa deletion in the BC helix and the CD corner, while gi|31198253 lacks part of the G and all of the H helices. Thus, it is likely that gi|31201271 and gi|57914327 are the only stable globins. The C-terminal 115aa globin domain of gi|31198253 is reminiscent of the Mb gene sequence from the antarctic icefish *Champsocephalus esox *(gi|24266940|AAN52371, 103aa) that does not appear to be expressed [68]. It remains to be determined whether the N-terminal domain of gi|31198253 stabilizes it enough to be expressed.

At least 33 putative globins and globin domains were identified in *Caenorhabditis elegans*, together with their orthologs in *C. briggsae *[30]: they are listed in Supplemental Data Table 5 [see Additional File 1]; their alignment is provided in Supplemental Data Fig. 2 [see Additional File 2]. Several *C. elegans/C. briggsae *orthologs have unusually long interhelical inserts between the G and H helices: 40aa in CE01012/CBP03870) (216aa), 21aa in CE01528 (CBP00622) (266aa), 22aa in CE34964/CBP23619 (196aa) and 21aa in CE34658/CBP07299 (230aa). Although three pairs, CE17437/CBP02580, CE04582/CBP03989 and CE35828/CBP14482, have N-terminal globin domains, several have C-terminal domains: CE03523/CBP15914, CE04843/CBP01576, CE05316/CBP02907, CE12774/CBP21478), CE36044/CBP02078), CE29586/CBP02293, CE30683/CBP02390 and CE31132/CBP15597. The N-terminal portion of the latter is identified as a G protein-coupled receptor-like domain with 7 putative transmembrane helices (identified using TMHMM Server V.2.0). The nonglobin domains in the remaining proteins could not be identified via blastp searches.

Although globin genes were thought to be absent in the genome of *Drosophila melanogaster *[69], the presence of at least one (CG9734) has been demonstrated unequivocally by Burmester and Hankeln [31,70]. There appear to be two more putative globins, CG15180 (209aa) and CG14675 (195aa); both provide acceptable alignments. Three putative globins are also found in *D. pseudoobscura*, EAL28410 (152aa), EAL28093 (641aa, 40–150) and EAL 28094 (130aa) [71], and appear to be orthologs of *D. melanogaster *CG9734, CG15180 and CG14675, respectively. Although the *D. pseudoobscura *EAL28093 and EAL28094 are scored by FUGUE as certainly globins (Z ~10), the former lacks an appropriate F8 His residue and the latter appears to be missing the A helix. Psiblast searches show that the ortholog pairs CG15180/EAL28093 and CG14675/EAL28094) recognize each other but not the CG9734/EAL28410 pair, and also recognize the *Anopheles *globins (ENSANG19788, 22287 and 26474), together with distant recognition of echinoderm globins and vertebrate β-globins. The CG9734/EAL28410 pair hits the other known insect globins (*Gasterophilus*, *Chironomus *species, *Kiefferulus*, *Tokunagayusurika*), the vertebrate Cygbs and the arthropod multidomain Hbs.

Three of the four *Arabidopsis thaliana *sequences, GLBs 1–3 have been identified earlier [19,72]: GLB1 and GLB2 are NsHbs and GLB3 is a 2/2Hb. A 693aa protein (gi|7486404|T04457) was found to have an N-terminal 2/2Hb domain, with the first 138aa (up to position H13), identical to GLB3. Although the ~500aa C-terminal portion does not correspond to any known protein domain(s), a blastp search using it as query found an identical sequence in the N-terminal portion of the 2154aa protein TEBICHI (gi|62241195|BAD93700). Both proteins share the same region on *Arabidopsis *chromosome 4 and TEBICHI has a helicase domain at 490–890aa and a polymerase domain at 1700–2150aa; furthermore, this N-terminal portion is missing in animal homologues of TEB protein, MUS308/POLQ (Dr. Soichi Inagaki, personal communication).

The genome of *Oryza sativa *contains the four NsHbs identified by Arredondo-Peter et al. [73,74], a 172aa 2/2Hb (gi|50725383|BAD32857) and a 145aa globin (gi|50932383|XP_475719). A blastp search identified the latter as another NsHbs.

Several putative globins occur in the genome of the green alga *Chlamydomonas reinhardtii*: 160981 (C_240146) (136aa), 160982 (C_240147) (147aa), 157690 (C_1780015) (231aa), and two larger sequences, 168934 (C_60169) (476aa) with one N-terminal globin-like domain, and 153190 (C_100138) (837aa) with two consecutive N-terminal globin domains. Two globins were found in the genome of the diatom *Phaeodactylum tricornutum*: PTMM3909 and 05212. Two globins occur in the genome of another diatom *Thalassiosira pseudonana*, Scaffold_137 (158aa) and Scaffold_18 (160aa), and a single 185aa globin, CMR319C, in the genome of the red alga *Cyanidioschyzon merolae*. Blastp searches identified all the *C. reinhardtii *globins, the *Phaeodactylum *PTMM 05212 and the *Thalassiosira *Scaffold_18 as 2/2 Hbs, and the *Cyanidioschyzon *CMR319C,*Phaeodactylum *PTMM 3909 and *Thalassiosir*a Scaffold_137 as SDgbs, closely related to the bacterial and fungal FHbs and the bacterial SDgbs.

The globins present in the fungi are listed in Supplemental Data Table 6 [see Additional File 1]. All the recently completed fungal genomes, which belong overwhelmingly to the Ascomycota (17 of 19), have FHbs; 14 have two to four FHbs, probably due to genome duplication [75]. In addition to *Saccharomyces cerevisiae *NP_014165 (426aa, 154–302) which was known to have a central globin domain [76], we found an additional 12 FHbs to have globin domains within the central portion of their sequences. All belong to the Saccharomycotina: *Candida albicans *EAK92722 (563aa, 298–463), *Candida glabrata *XP_448033 (432aa, 124–267), *Eremothecium (Ashbya) gossypii *NP_982746 (436aa, 184–339), *Kluyveromyces lactis *XP_453939 (430aa, 181–323), *Kluyveromyces waltii *Kwal_22190 (421aa, 171–314) Kwal_4395 (460aa, 202–352) and Kwal_24852 (543, 173–315), *Saccharomyces bayanus *ORFP:20532(424aa, 171–303), *Saccharomyces mikatae *ORFP:18051 (410aa, 135–290), *Saccharomyces paradoxus *ORFP:18484 (426aa, 155–306) and *Yarrowia lipolytica *XP_502881 (463aa, 186–325) and XP_499869 (471aa, 194–333). Although the globin domains align well with the other FHbs, the N- and C-terminal portions do not recognize the C-terminal moieties of the other fungal and bacterial FHbs or any other known protein, as found earlier for the *S. cerevisiae *FHb [76]. Surprisingly, the percent identities between the CDFHbs and the fungal and bacterial FHb globin domains is about 40% and 30%, respectively.

Among the lower eukaryotes, the diplomonad *Giardia lamblia *has one FHb [77] and the mycetozoan *Dictyostelium discoideum *has two [78]. The *Giardia *sequence has a 21aa insert between helices E and F.

No globin genes appear to exist in the genomes of the Apicomplexans *Entamoeba histolytica *and *Plasmodium falciparum*, as well as the Microsporidian *Encephalitozoon cuniculi *and the Euglenazoan *Trypanosoma brucei*. Although a *Plasmodium bergei *globin-like protein of 228aa (Q86QI8) is listed in the GenBank, it is probably an artefact, since when used as a query in blastp searches, it hits only vertebrate β- and α-globins.

Compared to Archaea and Bacteria, the fraction of eukaryote genomes with globins is much higher, over 90%. Although all vertebrates have Hb and Mb and probably Ngb and Cygb as well [38], the Antarctic icefish belonging to the family Channichthyidae do not express Hb [79]. Furthermore, at least 6 of the 16 icefish species also do not express Mb [80]. The lack of Hb is due apparently to deletion of the β-globin gene [81], which occurred during the last 10 to 16 Myr, the approximate date of divergence of the Notothenioid lineage including the Channichthyidae [82]. In contrst, the lack of Mb appears to be due to errors in Mb gene transcription [68]. It is not known whether the icefish have Ngbs and/or Cygbs. In the remaining two chordate phyla, the urochordate *Ciona *has 4 globins (see above), which when used as queries in psiblast searches, recognize the vertebrate globins and the eukaryote and bacterial FHbs and SDgbs. The report of a Hb in the notochord of the cephalochordate amphioxus (*Branchiostoma californiense*) [83], suggests that globins are present in all chordates. Among the remaining deuterostome phyla, intracellular Hbs have been reported in two of the 5 classes of echinoderms [84,85], and we find a putative globin in the recent assembly of the genome from the sea urchin *Strongylocentrotus purpuratus*, which belongs to another class. No globins have been reported so far in hemichordates.

Among the remaining metazoans, direct visual observation of Hbs is highly episodic among the metazoans, and varies from total absence of Hb in porifera and cnidaria, to frequent presence in some nematode and platyhelminth groups [3]. In the largest metazoan group, the insects, of which about 900,000 species have been described, and which outnumber the combined total of all other animal species, with estimates ranging from 2 × 10^6 ^to 100 × 10^6 ^[86], globins are so far known to be present only in Dipterans, such as *Gasterophilus*, *Drosophila *and *Chironomus*. It is evident that much work needs to be done before we have any clear idea of globin distribution in metazoans other than deuterostomes.

Among the lower eukaryotes, our census suggests that globins could be ubiquitous in fungi (FHbs), mycetozoa (FHbs), diplomonads (FHbs), ciliates (2/2 Hbs), stramenopiles (SDgb, 2/2Hb), rhodophytes (SDgb), chlorophytes (2/2Hb) and plants (SHbs, NsHbs, 2/2 Hbs). In contrast, the genomes of pathogenic microsporidians, entamoebae, apicomplexans anad trypanosomes are devoid of globins. In view of a very large potential diversity of small, bacterial-sized eukaryotes revealed by recent, culture-independent surveys [87,88], much remains to be done.

Since our census indicates that GCSs do not occur in eukaryotes, it is necessary to consider the heme-regulated eukaryotic initiation factor 2α kinase (~630aa), which was found recently to have two heme-binding domains, of which the N-terminal domain was considered to be globin-like [89,90] Although it was demonstrated that His78 and His123 were the two residues involved in heme binding [91], we find that the N-terminal 138aa of the rabbit protein (gi|462439|P33279|E2AK1 RABIT) is not recognized as a globin in CD or FUGUE searches. A blastp search of the GenBank database found at least 6 mammalian counterparts; clearly, a structure is required to determine whether these sequences are globins.

### Molecular evolution

**Figure 5:**
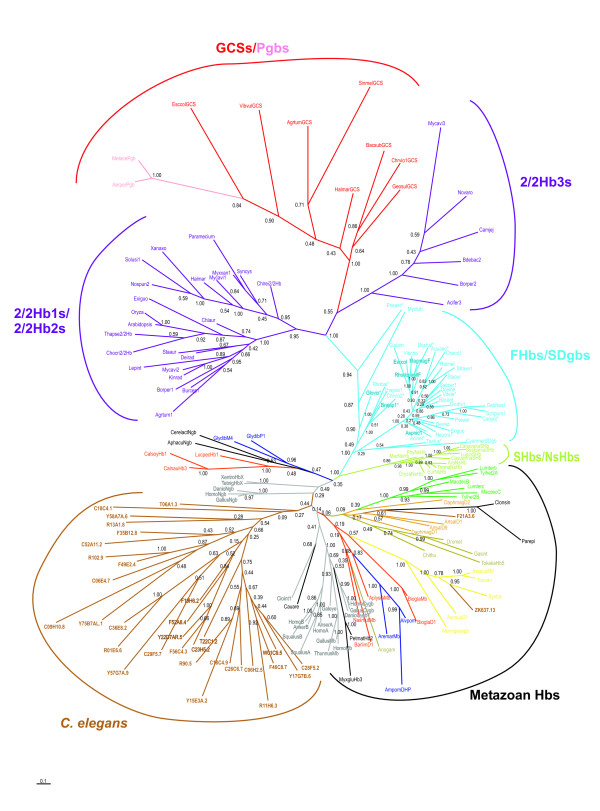
A Bayesian phylogenetic tree based on 175 sequences representing all the known globin families (alignment provided in Supplemental Data Fig. 3). The scale bar represents a distance of 0.1 accepted amino acid mutations per site.The numbers at the nodes represent Bayesian posterior probabillities.

The broad distribution of the three lineages of globins, the 3/3 FHbs/SDgbs, the 3/3 GCS/Pgbs and the 2/2 Hbs, is illustrated in the Bayesian phylogenetic tree shown in Fig. 5, based on a global manual alignment of 175 sequences representing all the major groups of globins (Supplemental Data Fig. 3, [see Additional File 2]). The 2/2 Hbs and the GCSs/Pgbs cluster separately (upper left hand side), with the three classes of 2/2 Hbs clearly delineated. Within the 2/2Hb clusters, the archaeal (*Haloarcula*), ciliate (*Paramecium*) and chlorophyte (*Chlamydomonas*) globins occur with the class 1 and the diatom (*Thalassiosira*) and plant (*Oryza, Arabidopsis*) Hbs group with the class 2. The remaining stem encompasses the bacterial and eukaryotic FHbs and related SDgbs (marked with an asterisk), including the *Cyanidioschyzon *and *Thalassiosira *SDgbs, the separate clusters of plant NsHbs and SHbs (lower right hand side) and all the known metazoan globins, including the vertebrate globins (lower left hand side). Noteworthy is the clustering of bacterial SDgbs with the globin domains of bacterial and eukaryote (fungal, diplomonad and mycetozoan) FHbs, suggestive of past lateral gene transfer events [49]. Likewise, the clustering of the archaeal 2/2 Hbs with the class 1 bacterial 2/2 Hbs, could be indicative of a past lateral gene transfer. Very recently, a 2/2Hb and an FHb were found in the multicellular red alga *Chondrus crispus *(X. Bailly *et al*., unpublished results): these two sequences cluster with the *Thalassiosira *2/2Hb and *Aquifex *SDgb, respectively. An interesting result is the close grouping of *Amphitrite ornata *dehaloperoxidase [92] with the intracellular hemoglobin of the hydrothermal vent annelid *Alvinella pompejana *[93], suggesting that the latter may be a dehaloperoxidase also. The *Gallus *and *Xenopus *HbX group with the vertebrate Ngbs as found earlier [40]. Of particular interest is the large *C. elegans *globin family: all except F21A3.6 and ZK637.13, cluster together, and appear to be closest to the vertebrate Ngbs. ZK637.13 groups with globins from other nematode species, F21A3.6 clusters with crustacean (*Daphnia *and *Artemia*) and platyhelminth (*Paramphistomum *and *Clonorchis*) sequences. It should be noted that the locations of these three globins is dependent on the number of globin sequences used to construct the phylogenetic tree.

We have discussed elsewhere [49] the possible evolutionary scenarios for the emergence of the proposed three lineages of globins in two structural classes: (1) the 3-over-3 SD globins and FHbs, (2) the 3-over-3 GCS/Pgbs, and (3), the 2-over-2 SDgbs. Here, we would like to reiterate our proposal that all metazoan and plant globins originated from a SDgb related to the present day bacterial and algal SDgbs and the N-terminal globin domains of fungal and bacterial FHbs. This proposal is based on two results, one of which, is the clustering of all metazoan and plant sequences with the bacterial and eukaryote FHbs and SDgbs and separate from the branches encompassing 2/2 Hbs and GCSs/Pgbs, in the Bayesian phylogenetic tree obtained earlier [49] and in the one shown in Fig. 5. Another key result, is that bacterial SDgbs and eukaryote FHb globin domains used as queries in iterated psiblast searches, recognize (i.e. have E values substantially lower than threshold) vertebrate globins, particularly Ngbs, and other metazoan globin groups, ahead of the 2/2 Hbs and GCSs/Pgbs [49]. Conversely, vertebrate Ngbs as a group, recognize the bacterial SDgbs ahead of other vertebrate globins. Furthermore, the tree shown in Fig. 5 is in very good agreement with recent models of vertebrate globin evolution [36,62,94], wherein duplication of the ancestral globin gene resulted in neuroglobin and cellular globin genes, with subsequent duplication of the latter into cellular and Hb gene loci, followed by additional duplications of the cellular globin locus into Mb and Cygb and of the Hb gene into the α- and β-globin genes.

The first attempt at defining the molecular phylogeny of globins [95] was a tree based on 245 sequences, all from eukaryotes. A subsequent study by Moens *et al*. [96] based on 700 sequences, including bacterial ones, proposed that all globins «evolved from a family of ancestral, ca. 17 kDa hemeproteins, which displayed the globin fold and functioned as redox proteins». The present survey of extant globins in the three kingdoms of life provides some interesting additional details: we now know that there are three lineages of globins, two with a 3/3 α-helical fold and one with the 2/2 fold, and that only the latter occurs at present in all three kingdoms of life. Furthermore, it appears likely that formation of chimeric proteins containing globin domains occurred, as separate events, prior to the emergence of the three kingdoms of life. There are no obvious explanations for the absence of FHbs in Archaea and of GCSs in eukaryotes. Although it is not possible at present to decide which of the two folds originated from the other, the occurrence of 2/2 Hbs in all three kingdoms of life would suggest that it is the ancestral fold. A recent computational study of plant Hb folding showed that one of the folding modules of rice 3/3 NsHb overlaps the 2/2 fold of *Mycobacterium tuberculosis *HbO (class 2) suggesting that it is an ancient structural feature of globins [97].

### Globin function

The pesence of multiple globins in all three kingdoms of life raises the question of their function, paticularly among the unicellular organisms. Although a complete discussion is not possible here, some general observations can be made, limited to organisms other than Animalia. The bacterial FHbs appear to have a role primarily in the detoxification of NO, via two NADH dependent activities, an NO dioxygenase activity under aerobic conditions, or an NO reductase activity under anaerobic conditions [42,43,98-100]. Furthermore, the recent demonstration that the *E. coli *FHb binds lipids and is an efficient alkylhydroperoxide reductase, suggests that it may be involved in the repair of lipid membranes damaged by oxidative/nitrosative stress [101-103]. The function(s) of bacterial SDgbs appear to be somewhat similar to the FHbs. Although the function of *Vitreoscilla *Hb is to increase the effective intracellular O_2_concentration under microaerobic conditions [42,104], that of *Campylobacter *SDgb was shown recently to function only in NO scavenging and detoxification and not provide resistance against superoxide or peroxides [105]. In eukaryotes, such as yeasts and *Dictyostelium*, FHbs also provide protection against NO [43,106,78], as well as enhancement of respiration, directly by functioning as an O_2 _buffer and indirectly, by reducing the NO concentration in the mitochondria which otherwise inhibits respiration [107-110]. In pathogenic microorganisms, FHb provides protection from human macrophage NO-mediated killing and promotes the virulence of bacteria, e.g. *Salmonella *[111], and of yeasts, *Candida *[112] and *Cryptococcus neoformans*, a worldwide pathogen causing pulmonary infection in animals and humans [113,114]. It is interesting to note, as suggested by one of the reviewers, that since most of the Archaea are not pathogenic, they may have dispensed with the FHbs and their protective role in bacteria.

In plants, the SHbs have been shown to be required for establishing a low oxygen concentration for the effective functioning of the bacterial nitrogenase necessary for nitrogen fixation [115]. Although the role of 2/2 Hbs in plants is completely unknown, the NsHbs, induced in plant cells upon exposure to low oxygen concentrations, are thought to play a role in a metabolic pathway which also involves nitric oxide and which provides an alternative type of respiration to the mitochondrial electron transport under hypoxic conditions [116,117].

The GCSs represent one of the four known families of heme-based sensors [118], and can be subdivided into two groups, the HemATs and the gene regulators. The former are aerotactic heme sensors, with a C-terminal domain that is related to chemotaxis methyl-accepting proteins. Although *Bacillus subtilis *HemAT elicits an aerophilic response, that from the archaean *Halobacterium salinarum *provides an aerophobic response [44,45]. The gene regulators have C-terminal domains, some of which may regulate second messengers and others that have unknown functions [47,48.59,118]. Although little is known about the specific function of the Pgbs, they have a Cys at position E19, similar to globins of the annelids living in sulfide-rich environments, including deep sea hydrothermal vents and marine sediments, where this residue has been implicated in sulfide binding [119,120].

Recent studies of *Mycobacterium tuberculosis *2/2 Hbs, HbN (2/2 Hb1) and HbO (2/2Hb2), indicate that the former functions in NO detoxification, while HbO, which differs substantially from HbN in structure [121], is expressed in association with cell membranes and significantly enhances respiration, suggesting an interaction with the electron transport chain [122-126]. It remains to be seen whether these findings apply to other bacterial 2/2 Hbs. Although nothing is known about the function of the 2/2Hb3s, it is safe to assume that it must differ from the other two classes, since 32 bacteria have two and *Mycobacterium avium ssp. tuberculosis *and *Methylococcus capsulatus *have all three classes of 2/2 Hbs.

Finally, it is appropriate to mention here the case of the extracellular Hb from the nematode *Ascaris suum*, an octamer of two-domain globin chains, long known for its high oxygen affinity [127], whose function appears to be that of an NO reductase [128], similar to that of fungal and bacterial FHbs. Interestingly FUGUE searches using FHb and SDgb sequences as queries, invariably include the *Ascaris *Hb domain 1 structure (1ash) among the highest scoring globin structures. This case provides an additional bit of evidence in support of our proposal that the FHbs and related SDgbs from bacteria, algae and unicellular eukaryotes, including fungi, are part of one of the three globin lineages, and the one from which originated all metazoan globins and all plant SHbs and NsHbs [49].

## Conclusion

The phylogenomic profile derived from our survey of genomes from the three kingdoms of life presented here, delineates the present day limits of the occurrence of the three lineages of globins, and provides a clear view of the work that remains to be done. It appears likely that in contrast to archaea, where ~20% of the known genomes have globins, the majority of bacteria will be shown to have globins. Based on the prevailing opinion that all plants have globins, it is likely that this will also hold true for the unicellular, photosynthesizing eukaryotes. Globin occurrence in other unicellular eukaryotes is likely to be episodic, just as in the case of nonvertebrate metazoans.

Another obvious conclusion is that globins are mostly enzymes and less frequently sensors, and that transport of oxygen is a function that developed relatively recently, accompanying the emergence of multicellular organisms. There are at least two more known instances of evolution from enzyme to transporter. One, is the convergent evolution of indoleamine dioxygenase into a muscle heme protein with Mb-like oxygen binding properties, in gastropod molluscs [129]. Another are hemocyanins, the copper containing respiratory proteins in molluscs and arthropods, which have evolved from phenoloxidases, prior to the divergence of Protostomes and Deuterostomes [130]. The recent finding of hemerythrins, similar to the nonheme iron respiratory protein of Sipunculids, Brachiopods and Priapulids, in the methanotrophic Gammaproteobacterium *Methylococcus capsulatus*, and in other prokaryotes [131], and also in the hydrothermal vent annelid *Riftia pachyptila *(X. Bailly et al., unpublished observations), where they may have an enzymatic function, suggests yet another possible instance.

## Methods

### Identification of globin sequences

Putative globins and globin domains were identified in the genomes of 49 eukaryotes, 26 archaea and 245 bacteria, listed in Supplementary Data Table 1, using two approaches. In one, we examined the gene assignments based on a library of hidden Markov models [132], listed on the SUPERFAMILY site , discarded sequences shorter than 100aa and checked the alignments for the presence of His at Mb-fold position F8. In the other, we performed blastp and tblastn (version 9.2.2) searches with pairwise alignment [133], of completed and unfinished genomes in the GenBank, using the NCBI Entrez retrieval system , including the genomes of the rhodophyte (red alga) *Cyanidioschyzon merolae *, chlorophyte (green algae) *Chlamydomonas reinhardtii *, and the diatoms *Phaeodactylum tricornutum * and *Thalassiosira pseudonana *. Blastp searches use the Expect value (E) to assess the matches between the query sequence and each of the sequences in a database. Thus, E = 0.1 signifies that the probability of finding by chance, another match with the query sequence having the same score, is 1 in 10. We define recognition to be a hit with E < 0.005, the default threshold, and with the pairwise alignment fulfilling the following two criteria: proper alignment of the F8 His residues and of helices BC through G. It should be noted that blastp searches often misalign the E helices when the E7 residues are different, e.g. His and Q; however, the rest of the alignment is unaffected.

In cases where the identification of a putative globin was uncertain, searches employing CD-Search v.2.02 [134], PFAM [135] and FUGUE [136] were used to determine whether the borderline sequence should be accepted as a globin.

### Alignment of sequences

The putative globin sequences were aligned manually, using the procedure employed earlier in the alignment of over 700 globins [137], based on the myoglobin fold [138,139], the pattern of predominantly hydrophobic residues at 37 conserved, solvent-inaccessible positions with mean solvent-accessible areas of <15Å^2 ^[140], including 33 intra-helical residues defining helices A through H, A8, A11, A12, A15, B6, B9, B10, B13, B14, C4, E4, E7, E8, E11, E12, E15, E18, E19, F1, F4, G5, G8, G11, G12, G13, G15, G16, H7, H8, H11, H12, H15, and H19, the three inter-helical residues at CD1, CD4 and FG4, and the invariant His at F8. Only amino acids which occur at the 33 intrahelical positions in the foregoing alignment were allowed in the alignment of the putative globin sequences. Although earlier alignments by Kapp et al. [137] and by Moens et al. [96] had indicated that there were two invariant residues in globins, F8His and CD1Phe, the 2/2Hb family can accommodate other hydrophobic residues, such as Tyr/Met/Leu/Ile/Val at the CD1 position as well as Ala/Ser/Thr/Leu at the distal E7 position, in addition to His and Gln [16,20-22]. Hence, in our alignments, we required a His at the proximal F8 position, a residue at the distal E7 position in the order of preference His>Gln>Leu~Thr>Ala~Val~Ser~Tyr, a hydrophobic residue at position CD1 in the order of preference Phe>Tyr>Leu>Met>Ile>Val. At position C4, usually a Pro, we accepted Ala or Ser or Thr but not a charged residue. At the interhelical position CD4, we sought a hydrophobic residue, when available, and at position FG4 we placed a hydrophobic residue in the order of preference Ile>Leu~Val>Met>Phe~Tyr. Furthermore, we avoided deletions in any of the helical regions and placed no limit on the number of residues within the interhelical regions.

### Molecular phylogeny

Bayesian phylogenetic trees were obtained employing MrBayes Version 3.1.1 [141]; four chains were run simultaneously for 2000000 generations and trees were sampled every 100 generations generating a total of 20000 trees. PAUP version 4.0b10 [142] was used for viewing and editing. The JTT transition matrix [143] was used as the stochastic model of amino acid substitution.

## Abbreviations

Cygb – 3-over-3 cytoglobin; FHb – flavohemoglobin, chimeric proteins (~400aa) comprising a 3-over-3 N-terminal globin and a C-terminal flavin reductase domain; GCS – globin-coupled sensors: chimeric proteins (~300 to >700aa) comprising a 3-over-3 N-terminal globin domain and a variable C-terminal portion; Hb – hemoglobin; SHb – 3-over-3 symbiotic Hbs of legumes and other plants; Mb – myoglobin; Ngb – 3-over-3 neuroglobin; NsHbs -3-over-3 nonsymbiotic plant Hbs; Pgb – protoglobin, single domain 3-over-3 globin related to the N-terminal domain of GCSs; SDgb – 3-over-3 single domain globin (~140aa) related to the N-terminal of FHbs; 3/3gbs – all globins that have the canonical 3-over-3 α-helical fold; 2/2 Hbs – "truncated" Hbs that are not necessarily shorter than ~140aa, which have the 2-over-2 α-helical fold.

## Authors' contributions

SNV identified globin sequences from genomic data and performed the alignments.

DH identified globin sequences and participated in sequence alignment and construction of phylogenetic trees.

XB identified globin sequences and participated in sequence alignment and construction of phylogenetic trees.

RAP provided systematic input and was involved in the preparation of the MS.

JG participated in the identification of globin sequences.

SD provided systematic input and was involved in the preparation of the MS.

LM provided systematic input and was involved in the preparation of the MS.

JVF provided systematic input and was involved in the preparation of the MS.

## Supplementary Material

Additional File 1 Table 1. List of genomes from the three kingdoms of life used in the present study. Table 2. Identified and putative globins in archaeal genomes. Table 3. Phylogenomic distribution of identified and putative globins in bacteria. Table 4. Identified and putative globins in eukaryote genomes. Table 5. The putative globin orthologs of *Caenorhabditis briggsae *and *C. elegans *Table 6. Identified and putative globins in fungal genomes. Click here for file

Additional File 2 Fig. 1. Alignment of selected globin sequences. Fig. 2. Alignment of putative globins from *Caenorhabditis briggsae *and *C. elegans*. Fig. 3. Alignment of 175 representative globin sequences used in the construction of the Bayesian phylogenetic tree shown in Fig. 5. Click here for file
