# Supplementary material for: A phylogenomic profile of globins
Source: BMC Evol Biol. 2006 Apr 7;6:31. doi: 10.1186/1471-2148-6-31 (PMC1457004; doi:10.1186/1471-2148-6-31)
Supplement: Additional File 1 — Table 1. List of genomes from the three kingdoms of life used in the present study. Table 2. Identified and putative globins in archaeal genomes. Table 3. Phylogenomic distribution of identified and putative globins in bacteria. Table 4. Identified and putative globins in eukaryote genomes. Table 5. The putative globin orthologs of Caenorhabditis briggsae and C. elegans Table 6. Identified and putative globins in fungal genomes. [file 1471-2148-6-31-S1.doc]

**TABLE 1. LIST OF GENOMES FROM THE THREE KINGDOMS OF LIFE.**

**METAZOA 14**

Unfinished Anopheles gambiae

Unfinished Brachydanio (Danio) rerio

Unfinished Caenorhabditis briggsae

Completed Caenorhabditis elegans

Completed Ciona intestinalis

Unfinished Drosophila melanogaster

Completed Gallus gallus

Completed Homo sapiens

Completed Mus musculus

Completed Rattus norvegicus

Unnfinished Strongylocentrotus purpuratus

Completed Takifugu (Fugu) rubripes

Unfinished Tetraodon nigroviridis

Completed Xenopus tropicalis

**PLANTS 6**

Completed Arabidopsis thaliana

Unfinished Chlamydomonas reinhardtii

Completed Cyanidioschyzon merolae

Completed Oryza sativa

Unnfinished Phaedactylum cornutum

Completed Thalassiosira pseudonana

**FUNGI 20**

**Ascomycota**

**Pezizomycotina**

Completed Aspergillus fumigatus

Completed Aspergillus nidulans

Completed Aspergillus niger

Completed Aspergillus oryzae

Completed Gibberella zeae

Completed Magnaporthe grisea

**Saccharomycotina**

Completed Candida albicans

Completed Candida glabrata

Completed Debaryomyces hansenii

Completed Eremothecium (Ashbya) gossypii

Completed Kluyveromyces lactis

Completed Kluyveromyces waltii

Completed Saccharomyces bayanus

Completed Saccharomyces cerevisiae

Completed Saccharomyces mikatae

Unfinished Saccharomyces paradoxus

Completed Yarrowia lipolytica

**Schizosaccharomycetes**

Completed Schizosaccharomyces pombe

**Sordariomycetes**

Completed Neurospora crassa

**Basidiomycota**

Completed Cryptococcus neoformans

Unfinished Ustilago maydis

**OTHER EUKARYOTES 9**

**Apicomplexa**

Completed Plasmodium falciparum

Unfinished Plasmodium yoelii yoelii

**Diplomonadida**

Unfinished Giardia lamblia

**Entamoebidae**

Unfinished Entamoeba histolytica

**Euglenozoa**

Completed Leishmania major

Completed Trypanosoma brucei

Unfinished Trypanosoma cruzi

**Microsporidia**

Completed Encephalitozoon cuniculi

**Mycetozoa**

Unfinished Dictyostelium discoideum

**ARCHAEA 26**

**Crenarcheota**

Completed Aeropyrum pernix

Completed Pyrobaculum aerophilum

Completed Sulfolobus acidocaldarius

Completed Sulfolobus solfataricus

Completed Sulfolobus tokodaii

**Euryarcheota**

Completed Archaeoglobus fulgidus

Unfinished Ferroplasma acidarmanus

Completed Haloarcula marismortui

Completed Halobacterium salinarum

Unfinished Haloferax volcanii

Completed Methanocaldococcus jannaschii

Completed Methanococcus maripaludis

Completed Methanopyrus kandleri

Unfinished Methanococcoides burtonii

Completed Methanosarcina acetivorans

Unfinished Methanosarcina barkeri

Completed Methanosarcina mazei

Completed Methanothermobacter thermautotrophicus

Completed Picrophilus torridus

Completed Pyrococcus abyssi

Completed Pyrococcus furiosus

Completed Pyrococcus horikoshii

Completed Thermococcus kodakaraensis

Completed Thermoplasma acidophilum

Completed Thermoplasma volcanium

**Nanoarcheota**

Completed Nanoarchaeum equitans

**BACTERIA 245**

**Acidobacteria 1**

Unfinished Solibacter usitatus

**Actinobacteridae (high G+C, Gram +) 26 Actinomycetales**

Unfinished Arthrobacter sp.FB24

Unfinished Brevibacterium linens

Completed Corynebacterium diphtheriae

Completed Corynebacterium efficiens

Completed Corynebacterium glutamicum

Completed Corynebacterium jeikeium

Unfinished Frankia sp.EAN1pec

Unfinished Frankia sp.CcI3

Unfinished Kineococcus radiotolerans

Completed Leifsonia xyli

Completed Mycobacterium avium ssp. paratuberculosis

Completed Mycobacterium bovis

Completed Mycobacterium leprae

Unfinished Mycobacterium marinum

Unfinished Mycobacterium smegmatis

Completed Mycobacterium tuberculosis

Completed Nocardia farcinica

Unfinished Nocrdioides sp.JS614

Completed Propionibacterium acnes

Completed Streptomyces avermitilis

Completed Streptomyces coelicolor

Unfinished Thermobifida fusca

Completed Tropheryma whipplei

**Bifidobacteriales**

Completed Bifidobacterium longum

**Rubrobacteridae**

Unfinished Rubrobacter xylanophilus

**Unclassified actinobacteria**

Completed Symbiobacterium thermophilum

**Aquificales 2**

Completed Aquifex aeolicus

Unfinished Sulfurihydrogenibium azorense

**Bacteroidetes/Chlorobi 5**

Completed Bacteroides fragilis

Completed Bacteroides thetaiotaomicron

Completed Chlorobium tepidum

Unfinished Cytophaga hutchinsonii

Completed Porphyromonas gingivalis

**Chlamydiae/Verrucomicrobia 6**

Completed Chlamydia muridarum

Completed Chlamydia trachomatis

Completed Chlamydophila caviae

Completed Chlamydophila pneumoniae

Completed Parachlamydia sp. UWE25

Unfinished Verrucomicrobium spinosum

**Chloroflexi (Green Nonsulfur Bacteria) 3**

Unfinished Chloroflexus aurantiacus

Completed Dehalococcoides ethenogenes

Unfinished Thermomicrobium roseum

**Cyanobacteria 12**

**Chrooccocales**

Unfinished Crocosphaera watsonii

Completed Synechococcus elongatus

Completed Synechococcus sp. WH 8102

Completed Synechocystis sp. PCC 6803

Completed Thermosynechococcus elongatus

**Gloebacterales**

Completed Gloeobacter violaceus **Nostocales**

Unfinished Anabaena variabilis

Unfinished Nostoc commune

Unfinished Nostoc punctiforme

Completed Nostoc sp. PCC 7120

**Oscillatoriales**

Unfinished Trichodesmium erythraeum

**Prochlorales**

Completed Prochlorococcus marinus

**Deinococcus/Thermus 3**

Completed Deinococcus radiodurans

Unfinished Deinococcus geothermalis

Completed Thermus thermophilus

**Firmicutes (low G+C, Gram +) 49**

**Bacillales 17**

Completed Bacillus anthracis

Completed Bacillus cereus

Completed Bacillus clausii

Completed Bacillus halodurans

Completed Bacillus licheniformis

Unfinished Bacillus stearothermophilus

Completed Bacillus subtilis

Completed Bacillus thuringiensis

Unfinished Exiguobacterium sp.255-15

Completed Geobacillus kaustophilus

Completed Listeria innocua

Completed Listeria monocytogenes

Completed Oceanobacillus iheyensis

Completed Staphylococcus aureus

Completed Staphylococcus epidermidis

**Clostridia 7**

Unfinished Carboxydothermus hydrogenoformans

Completed Clostridium acetobutylicum

Completed Clostridium perfringens

Completed Clostridium tetani

Unfinished Desulfitobacterium hafniense

Unfinished Moorella thermoacetica

Completed Thermoanaerobacter tengcongensis

**Lactobacillales 13**

Completed Enterococcus faecalis

Completed Lactobacillus acidophilus

Completed Lactobacillus johnsonii

Completed Lactobacillus plantarum

Completed Lactococcus lactis

Unfinished Leuconostoc mesenteroides

Unfinished Oenococcus oeni

Unfinished Pediococcus pentosaceus

Completed Streptococcus agalactiae

Completed Streptococcus mutans

Completed Streptococcus pneumoniae

Completed Streptococcus pyogenes

Completed Streptococcus thermophilus

**Mollicutes 12**

Completed Mesoplasma florum

Completed Mycoplasma gallisepticum

Completed Mycoplasma genitalium

Completed Mycoplasma hyopneumoniae

Completed Mycoplasma mobile

Completed Mycoplasma mycoides

Completed Mycoplasma penetrans

Completed Mycoplasma pneumoniae

Completed Mycoplasma pulmonis

Completed Mycoplasma synoviae

Completed Onion yellows phytoplasma

Completed Ureaplasma parvum

**Fusobacteria 1**

Completed Fusobacterium nucleatum

**Nitrospirae 1**

Unfinished Thermodesulfovibrio yellowstonii **Planctomycetes 2**

Unfinished Gemmata obscuriglobus

Completed Rhodopirellula baltica

**Alphaproteobacteria 35**

**Caulobacterales 1**

Completed Caulobacter crescentus

**Rhizobiales 12**

Completed Agrobacterium tumefaciens

Completed Bartonella henselae

Completed Bartonella quintana

Completed Bradyrhizobium japonicum

Completed Brucella abortus

Completed Brucella melitensis

Completed Brucella suis

Completed Mesorhizobium loti

Unfinished Mesorhizobium sp. BNC1;

Unfinished Nitrobacter hamburgensis

Completed Rhodopseudomonas palustris

Completed Sinorhizobium meliloti

**Rhodobacterales 6**

Unfinished Hyphomonas neptunium

Unfinished Jannaschia sp.CCS1

Unfinished Paracoccus denitrificans

Unfinished Rhodobacter sphaeroides

Completed Silicibacter pomeroyi

Unfinished Silicibacter sp. TM1040

**Rhodospirilalles 3**

Completed Gluconobacter oxydans

Unfinished Magnetospirillum magnetotacticum

Unfinished Rhodospirillum rubrum

**Rickettsiales 10**

Completed Anaplasma marginale

Completed Ehrlichia canis

Completed Ehrlichia ruminantium

Unfinished Pelagibacter ubique

Completed Rickettsia conorii

Completed Rickettsia felis

Completed Rickettsia prowazekii

Completed Rickettsia typhi

Completed Wolbachia endosymbiont of

Breugia malayi

Complete Wolbachia endosymbiont of

Drosophila melanogaster

**Sphingomonadales 3**

Unfinished Novosphingobium aromaticivorans

Unfinished Sphingopyxis alaskensis

Completed Zymomonas mobilis

**Betaproteobacteria 24**

**Burkholderiales 15**

Completed Bordetella bronchiseptica

Completed Bordetella parapertussis

Completed Bordetella pertussis

Unfinished Burkholderia ambifaria

Unfinished Burkholderia cepacia

Unfinished Burkholderia fungorum

Completed Burkholderia mallei

Completed Burkholderia pseudomallei

Unfinished Burkholderia vietnamiensis

Unfinished Polaromonas sp. JS666

Unfinished Ralstonia eutropha

Unfinished Ralstonia metallidurans

Completed Ralstonia solanacearum

Unfinished Rhodoferax ferrireducens

Unfinished Rubrivivax gelatinosus

**Hydrogenophilales 1**

Unfinished Thiobacillus denitrificans

**Methylophilales 1**

Unfinished Methylobacillus flagellatus

**Neisseriaceae 3**

Completed Chromobacterium violaceum

Completed Neisseria gonorrhoeae

Completed Neisseria meningitidis

**Nirosomonadales 2**

Completed Nitrosomonas europaea

Unfinished Nitrosomonas eutropha

**Rhodocyclales 2**

Completed Azoarcus sp. EbN1

Unfinished Dechloromonas aromatica

**Gammaproteobacteria 50**

**Acidiothiobacillales 1**

Unfinished Acidiothiobacillus ferrooxidans

**Alteromonadales 8**

Completed Colwellia psychoerythrae

Completed Idiomarina loihiensis

Unfinished Microbulbifer (Saccharophagus) degradans

Unfinished Shewanella amazonensis

Unfinished Shewanella baltica

Unfinished Shewanella denitrificans

Unfinished Shewanella frigidimarina

Completed Shewanella oneidensis

**Enterobacteriales 10**

Completed Buchnera aphidicola

Completed Blochmannia floridanus

Completed Erwinia carotovora

Unfinished Erwinia chrysanthemi

Completed Escherichia coli

Completed Photorhabdus luminescens

Completed Shigella flexneri

Completed Wigglesworthia glossinidia

Completed Yersinia pestis

Completed Yersinia pseudotuberculosis

**Legionellales 2**

Completed Coxiella burnetii

Completed Legionella pneumophila

**Methylococcales 1**

Completed Methylococcus capsulatus

**Oceanospirillales 1**

Unfinished Chromohalobacter salexigens

**Pasteurellales 5**

Unfinished Actinobacillus pleuropneumoniae

Completed Haemophilus ducreyi

Completed Haemophilus influenzae

Completed Mannheimia succiniciproducens

Completed Pasteurella multocida

**Pseudomonadales 10**

Completed Acinetobacter sp.ADP1

Unfinished Azotobacter vinelandii

Completed Pseudomonas aeruginosa

Completed Pseudomonas fluorescens

Completed Pseudomonas putida

Unfinished Pseudomonas stutzeri

Completed Pseudomonas syringae

Completed Psychrobacter sp.273-4

Completed Psychrobacter arcticum

Unfiinished Psychrobacter cryohelolensis

**Salmonellacea 2**

Completed Salmonella enterica

Completed Salmonella typhimurium

**Thiotrichales 1**

Completed Francisella tularensis

**Vibrionales 5**

Completed Photobacterium profundum

Completed Vibrio cholerae

Completed Vibrio fischeri

Completed Vibrio parahaemolyticus

Completed Vibrio vulnificus

**Xanthomonadacea 4**

Completed Xanthomonas axonopodis

Completed Xanthomonas campestris

Completed Xanthomonas oryzae

Completed Xylella fastidiosa

**Deltaproteobacteria 10**

**Bdellovibrionales 1**

Completed Bdellovibrio bacteriovorus

**Desulfobacterales 1**

Completed Desulfotalea psychrophila

**Desulfovibrionales 2**

Unfinished Desulfovibrio desulfuricans

Completed Desulfovibrio vulgaris

**Desulfuromonadales 4**

Unfinished Desulforomonas aceetoxidans

Unfinished Geobacter metallireducens

Completed Geobacter sulfurreducens

Unfinished Pelobacter propionicus

**Myxococcales 2**

Unfinished Myxococcus xantus

Completed Anaeromyxobacter dehalogenans

**Epsilonproteobacteria 8**

Unfinished Campylobacter coli

Completed Campylobacter jejuni

Unfinished Campylobacter lari

Unfinished Campylobacter upsaliensis

Completed Helicobacter hepaticus

Completed Helicobacter pylori

Completed Wolinella succinogenes

**Unclassified Proteobacteria 1**

Unfinished Magnetococcus sp. MC-1

**Spirochaetales 5**

Completed Borrelia burgdorferi

Completed Borrelia garinii

Completed Leptospira interrogans

Completed Treponema denticola

Completed Treponema pallidum

**Thermotogales 1**

Completed Thermotoga maritim

**TABLE 2. Globins in archeal genomes** ^1^**.**

| **Taxon** | **Genome size, Mbp ^2^** | **Identifier** | **Size and**  **GD (aa)** | **Type of**  **globin** |
| --- | --- | --- | --- | --- |
| **Crenarchaeota** |  |  |  |  |
| *Aeropyrum pernix* | 1.67 | gi|14600601|NP_147118 | 195 | Pgb |
| *Pyrobaculum aerophilum* | 2.22 | No Globins |  |  |
| *Sulfolobus acidocaldarius* | 2.23 | No Globins |  |  |
| *Sulfolobus solfataricus* | 2.99 | No Globins |  |  |
| *Sulfolobus tokodaii* | 2.69 | No Globins |  |  |
| **Euryarcheota** |  |  |  |  |
| *Archeoglobus fulgidus* | 2.18 | No Globins |  |  |
| *Haloarcula marismortui* | 4.275 | gi55379358|YP_137208  gi|55229828|AAV45247 | 120  497 | 2/2Hb1  GCS |
| *Halobacterium salinarum* | 2.57 | gi:15790497|NP280321 | 490, 42-186 | GCS |
| *Haloferax volcanii* | U, 4.03 | gnl|TIGR_309800|contig:441  gnl|TIGR_309800|contig:441 |  | 2/2Hb1  GCS |
| *Methanocaldococcus jannaschii* | 1.74 | No Globins |  |  |
| *Methanococccus maripaludis* | 1.66 | No Globins |  |  |
| *Methanopyrus kandleri* | 1.69 | No Globins |  |  |
| *Methanosarcina acetivorans* | 5.75 | gi|20091705|NP_617780 | 195 | Pgb |
| *Methanosarcina barkeri* | U, 5.33 | gi|48837815|ZP_000294773 | 195 | Pgb |
| *Methanosarcina mazei* | 4.10 | No Globins |  |  |
| *Methanothermobacter*  *thermoautotrophicus* | 1.75 | No Globins |  |  |
| *Picrophilus torridus* | 1.55 | No Globins |  |  |
| *Pyrococcus abyssi* | 1.77 | No Globins |  |  |
| *Pyrococcus furiosus* | 1.91 | No Globins |  |  |
| *Pyrococus horikoshii* | 1.74 | No Globins |  |  |
| *Thermococcus kodakaraensis* | 2.09 | No Globins |  |  |
| *Thermoplasma acidophilum* | 1.56 | No Globins |  |  |
| *Thermoplasma volcanium* | 1.58 | No Globins |  |  |
| **Nanoarcheota** |  |  |  |  |
| *Nanoarcheum equitans* | 0.49 | No Globins |  |  |

^1^GCS – globin-coupled sensors: chimeric proteins (~300 to >700aa) comprising a 3/3 N-terminal globin domain and a variable C-terminal portion; GD – globin domain; Pgb – protoglobin, single domain 3/3 globin related to the N-terminal domain of GCSs; 2/2Hbs – “truncated” Hbs that are not necessarily shorter than ~140aa, which possess a characteristic 2-over-2 α-helical fold. Sequences with <100aa were not considered.

^2^ Taken from [www.ncbi.nlm.nih.gov/genomes/lproks.cgi](http://www.ncbi.nlm.nih.gov/genomes/lproks.cgi) and [www.ncbi.nlm.nih.gov/genomes/MICROBES/](http://www.ncbi.nlm.nih.gov/genomes/MICROBES/) InProgress.html.

**TABLE 3. Phylogenomic distribution of IDENTIFIED AND PUTATIVE globins in bacteria ^1^.**

| **Taxon** | **Genome**  **size,**  **Mbp ^2^** | **2/2Hbs ^3^** | **FHbs/**  **(SDgbs)** | **GCSs/ (Pgbs)** | **Total** | **Oxygen requirement and habitat ^4^** |
| --- | --- | --- | --- | --- | --- | --- |
| **Acidobacteria**  *Solibacter usitatus* | U, 9.91 | 2 (1,2) | - | - | 2 | Ae, Th |
| **Actinobacteridae** (high G+C, Gram +) |  |  |  |  |  |  |
| **Actinomycetales** |  |  |  |  |  |  |
| *Arthrobacter sp.FB24*  *Brevibacterium linens*  *Corynebacterium diphtheriae*  *Corynebacterium efficiens*  *Corynebacterium glutamicum*  *Corynebacterium jeikeium*  *Frankia sp.EAN1pec*  *Frankia sp.CcI3*  *Kineococcus radiotolerans*  *Leifsonia xylii*  *Mycobacterium avium ssp.*  *tuberculosis*  *Mycobacterium bovis*  *Mycobacterium leprae*  *Mycobacterium marinum*  *Mycobacterium smegmatis*  *Mycobacterium tuberculosis*  *Nocardia farcinica*  *Nocardioides sp.JS614*  *Propionibacterium acnes*  *Streptomyces avermitilis*  *Streptomyces coelicolor*  *Thermobifida fusca*  *Tropheryma whipplei* | U, 5.01  U, 4.37  2.49  3.15  3.31  2.46  U, 9.04  U, 5.4  U, 4.89  2.58  4.83  4.35  3.27  U, 6.56  U, 7.04  4.40  6.29  U, 5.35  2.56  9.12  9.02  U, 3.64  0.93 | -  1(2)  1 (2)  1 (2)  1 (2)  1 (2)  5 (1,2)  2 (1,2)  1 (2)  1 (2)  3 (1,2,3)  2 (1,2)  1 (2)  1 (1,2)  2 (1,2)  2 (1,2)  1 (2)  1 (2)  -  2 (2)  1 (2)  1 (2)  - | 1  -  -  1  2  -  -  -  -  -  1  1  -  -  -  1  3  -  -  2  3  1, (1)  - | -  -  -  -  -  -  -  -  -  -  -  -  -  -  -  -  -  -  -  -  -  (1)  - | 1  1  1  2  3  1  5  2  1  1  4  3  1  1  2  3  4  1  0  4  4  4  0 | ?  Ae, M  Ae, M  F, M  F, M  F, M  Ae, M  Ae, M  Ae, M  Ae, HA  Ae, HA  Ae, HA  Ae, HA  Ae, HA  Ae, HA  Ae, HA  Ae, M  Ae, Tr  An, HA  Ae, M  Ae, M  Ae, M  Ae, HA |
| **Bifidobacteriales**  *Bifidobacterium longum* | 2.26 | - | - | - | 0 | An, HA |
| **Rubrobacteridae**  *Rubrobacter xylanophilus* | U, 3.17 | - | - | (1) | 1 | Ae, Sp |
| **Symbiobacteria**  *Symbiobacterium thermophilum*  Globins present/total | 3.57 | - | - | - | 0  22/26 | MAe, Tr |
| **Aquificales/Thermotogales**  *Aquifex aeolicus*  *Sulfurihydrogenibium azorense*  *Thermotoga maritima* | 1.59  U, ?  1.86 | -  -  - | (1)  -  - | -  1  - | 1  1  0 | Ae, Sp  MAe, Aq  An, Sp |
| **Bacteroidetes/Chlorobi**  (Green Sulfur Bacteria)  *Bacteroides fragilis*  *Bacteroides thetaiotamicron*  *Chlorobium tepidum*  *Cytophaga hutchinsonii*  *Porphyromonas gingivalis*  Globins present/total | 5.31  6.29  2.15  U, 4.42  2.34 | -  -  -  1 (2)  - | -  -  -  -  - | -  -  -  -  - | 0  0  0  1  0  1/5 | An, HA  An, HA  An, Sp  Ae, M  An, HA |
| **Chlamydiae/Verrumicrobia**  *Chlamydia muridarum*  *Chlamydia trachomatis*  *Chlamydophila caviae*  *Chlamydophila pneumoniae*  *Parachlamydia sp. UWE25*  *Verrucomicrobium spinosum*  Globins present/total | 1.07  1.04  1.18  1.23  2.41  U, 8.39 | -  -  -  -  1 (2)  1 (2) | -  -  -  -  -  - | -  -  -  -  -  - | 0  0  0  0  1  1  2/6 | HA  HA  HA  HA  HA  F, M |
| **Chloroflexi** (Green Nonsulfur bacteria)  *Chloroflexus aurantiacus*  *Dehalococcoides ethenogenes*  Thermomicrobium roseum | U, 4.93  1.47  U, ? | 2 (2)  -  - | -  -  - | (1)  -  1 | 3  0  1 | An, Sp  An, M  Ae, Sp |
| **Cyanobacteria** (Blue green algae)  **Chrooccocales**  *Crocosphaera watsonii*  *Synechococcus elongatus*  *Synechoccus sp.PCC7002*  *Synechocystis sp.PCC6803*  *Thermosynechoccus elongatus*  **Gloebacterales**  *Gloebacter violaceus*  **Nostocales**  *Anabaena variabilis*  *Nostoc commune*  *Nostoc punctiforme*  *Nostoc sp. PCC7120*  **Oscillatoriales**  *Trichodesmium erythraeum*  **Prochlorales**  Prochlorococcus marinus  Globins present/total | U, 6.17  2.70  2.43  3.57  2.59  4.66  U, 7.06  U, 6.41  U, 9.02  7.21  U, 7.79  2.41 | -  -  1 (1)  1 (1)  -  -  -  1 (1)  2 (1)  1 (1)  -  - | -  -  -  -  -  (1)  -  -  (1)  -  -  - | -  -  -  -  1  -  -  -  -  -  -  - | 0  0  1  1  1  1  0  1  3  1  0  0  7/12 | Aq  Aq  Aq  Aq  Sp  Tr  Ae, M  Ae, M  Ae, M  Ae, M  Ae, Aq  Aq |
| **Deinocococcus/Thermus**  *Deinococcus radiodurans*  *Deinococcus geothermalis*  *Thermus thermophilus* | 3.28  U, 3.16  1.89 | 1 (2)  3 (2,3)  - | 1  -  - | -  -  1 | 2  3  1 | Ae, Tr  Ae, Aq  Ae, Sp |
| **Firmicutes** (Low G + C, Gram +)  **Bacilli**  Bacillales  *Bacillus anthracis*  *Bacillus cereus*  *Bacillus clausii*  *Bacillus halodurans*  *Bacillus licheniformis*  *Bacillus stearothermophilus*  *Bacillus subtilis*  *Bacillus thuringiensis*  *Exiguobacterium sp. 255-15*  *Geobacillus kaustophilus*  *Listeria innocua*  *Listeria monocytogenes*  *Oceanobacillus iheyensis*  *Staphylococcus aureus*  *Staphylococcus epidermidis*  *Staphylococcus haemolyticus*  *Staphylococcus saprophyticus*  Globins present/total | 5.50  5.43  4.30  4.20  4.22  U, 3.34  4.21  5.31  U, 2.89  3.59  3.09  2.91  3.63  2.85  2.56  2.69  2.58 | 1 (2)  1 (2)  1 (2)  1 (2)  1 (2)  1 (2)  1 (2)  1 (2)  1 (2)  1 (2)  -  -  1 (2)  1 (2)  2 (2)  1 (2)  - | 1  1  1  1  -  -  1  1  1  1  -  -  1  1  1  1  1 | 1  1  1  1  1  -  1  1  2  -  -  -  -  -  -  -  - | 3  3  3  3  2  1  3  3  4  2  0  0  2  2  3  2  1  15/17 | F, M  Ae, Tr  ?  F, M  F, Tr  ?  F, Tr  F, M  F, Sp  Ae, Aq  F, M  F, M  Ae, M  F, HA  F, HA  F, HA  Ae, HA |
| **Lactobacillales**  *Enterococcus faecalis*  *Lactobacillus acidophilus*  *Lactobacillus johnsonii*  *Lactobacillus plantarum*  *Lactococcus lactis*  *Leuconostoc mesenteroides*  *Oenococcus oeni*  *Pediococcus pentosaceus*  *Streptococcus agalactiae*  *Streptococcus mutans*  *Streptococcus pneumoniae*  *Streptococcus pyogenes*  *Streptococcus thermophilus* | 3.36  1.99  1.99  3.35  2.37  U, 2.13  U, 1.76  U, 1.76  2.20  2.03  2.16  1.90  1.80 | -  -  -  -  -  -  -  -  -  -  -  -  - | -  -  -  -  -  -  -  -  -  -  -  -  - | -  -  -  -  -  -  -  -  -  -  -  -  - | 0  0  0  0  0  0  0  0  0  0  0  0  0 | FAn  F, M  F, HA  F, HA  F, M  F, M  F, M  F, M  F, HA  F, HA  F, HA  F, HA  F, M |
| **Clostridia**  *Carboxydothermus hydrogenformans*  *Clostridium acetobutylicum*  *Clostridium perfringens*  *Clostridium tetani*  *Desulfitobacterium hafniense*  *Moorella thermoacetica*  *Thermoanaerobacter tengcongensis*  Globins present/total | U, 2.4  4.13  3.09  2.87  U, 5.16  U, 2.63  2.69 | -  -  -  -  1 (3)  -  - | -  -  (1)  -  (1)  -  - | 1  -  -  -  1  1  - | 1  0  1  0  3  1  0  4/7 | An, Aq  An, M  An, M  An, M  An, Sp  An, Aq  An, Sp |
| **Mollicutes**  *Mesoplasma florum*  *Mycoplasma gallisepticum*  *Mycoplasma genitalium*  *Mycoplasma hyopneumoniae*  *Mycoplasma mobile*  *Mycoplasma mycoides*  *Mycoplasma penetrans*  *Mycoplasma pneumoniae*  *Mycoplasma pulmonis*  *Mycoplasma synoviae*  *Onionyellow phytoplasma*  *Ureaplasma parvum*  Globins present/total | 0.79  1.00  0.58  0.89  0.78  1.21  1.36  0.82  0.96  0.60  0.86  0.75 | -  -  -  -  -  -  -  -  -  -  -  - | -  -  -  -  -  -  -  -  -  -  -  - | -  -  -  -  -  -  -  -  -  -  -  - | 0  0  0  0  0  0  0  0  0  0  0  0  19/49 | F, HA  F, HA  F, HA  F, HA  F, HA  F, HA  F, HA  F, HA  F, HA  F, HA  Ae, HA  F, HA |
| **Fusobacteria**  *Fusobacterium nucleatum* | 2.17 | - | - | - | 0 | An, HA |
| **Nitrospirae**  *Thermodesulfovibrio yellowstonii* | U, ? | - | - | 1 | 1 | Aq |
| **Planctomycetes**  *Gemmata obscuriglobus*  *Rhodopirellula baltica* | U, 9.02  7.15 | 1 (1)  1 (1) | -  1, (1) | -  - | 1  3 | Ae, M  Ae, Aq |
| **Proteobacteria** (Purple bacteria) |  |  |  |  |  |  |
| **Alphaproteobacteria**  **Caulobacterales**  *Caulobacter crescentus* | 4.02 | 1 (3) | - | 2 | 3 | Ae, Aq |
| **Rhizobiales**  *Agrobacterium tumefaciens*  *Bartonella henselae*  *Bartonella quintana*  *Bradyrhizobium japonicum*  *Brucella abortus*  *Brucella melitensis*  *Brucella suis*  *Mezorhizobium loti*  *Mezorhizobium sp. BNC1*  *Nitrobacter hamburgensis*  *Rhodopseudomonas palustris*  *Sinorhizobium meliloti* **Rhodobacterales**  *Hyphomonas neptunium*  *Jannaschia sp.CCS1*  *Paracoccus denitroficans*  *Rhodobacter sphaeroides*  *Silicibacter pomeroyi*  *Silicibacter sp.TM1040*  **Rhodospirilalles**  *Gluconobacter oxydans*  *Magnetospirillum magnetotacticum*  *Rhodospirillum rubrum*  **Rickettsiales**  *Anaplasma marginale*  *Ehrlichia canis*  *Ehrlichia ruminantium*  *Pelagibacter ubique*  *Rickettsia conorii*  *Rickettsia felis*  *Rickettsia prowazekii*  *Rickettsia typhi*  *Wolbachia endosymbiont*  *of Breugia malayi*  *Wolbachia endosymbiont*  *of Drosophila melanogaste*r  **Sphingomonadales**  *Novosphingobium aromaticivorans*  *Sphingopyxis alaskensis*  *Zymomonas mobilis*  Globins present/total | 5.67  1.93  1.58  9.11  3.29  3.29  3.32  7.60  U, 4.92  U, 5.01  5.46  6.69  U, 3.71  U, 4.40  U, 5.18  U, 4.58  4.60  U, 4.14  2.92  U, 9.21  U, 4.40  1.20  1.35  1.52  U, 1.31  1.27  1.49  1.11  1.11  1.08  1.27  U, 4.21  U, 3.34  2.06 | 4 (2,3)  -  -  2 (2,3)  1 (3) ^5^  1 (3) ^5^  1 (3) ^5^  1 (2)  1 (3)  1 (3)  1 (3)  2 (2,3)  1 (1)  1 (2)  1 (3)  -  2 (2,3)  1 (3)  -  -  -  -  -  -  1 (1)  -  -  -  -  -  -  3 (2,3)  1 (3)  - | -  -  -  (2)  -  -  -  -  -  -  (2)  1  -  (1)  1  -  (1)  (1)  1  1  -  -  -  -  -  -  -  -  -  -  -  (1)  -  - | 3  -  -  -  -  -  -  -  -  -  -  1  -  -  -  1  -  3  1  2  3  -  -  -  -  -  -  -  -  -  -  1  1  1 | 7  0  0  4  1  1  1  1  1  1  3  4  1  2  2  1  3  5  2  3  3  0  0  0  1  0  0  0  0  0  0  5  2  1  24/35 | Ae, M  Ae, HA  Ae, HA  Ae, HA  F, HA  F, HA  Ae, HA  Ae, M  Ae, M  Ae, Tr  F, M  Ae, M  An, Aq  Ae, Aq  Ae, M  F, M  Ae, Aq  M  Ae, M  MAe, Aq  F, M  Ae, HA  HA  HA  ?  Ae, HA  HA  Ae, HA  Ae, HA  HA  HA  Ae, M  Ae, Aq  An, M |
| **Betaproteobacteria** |  |  |  |  |  |  |
| **Burkholderiales**  *Bordetella bronchiseptica*  *Bordetella parapertussis*  *Bordetella pertussis*  *Burkholderia ambifaria*  *Burkholderia cepacia*  *Burkholderia fungorum*  *Burkholderia mallei*  *Burkholderia pseudomalllei*  *Burkholderia vietnamiensis*  *Polaromonas sp.JS666*  *Ralstonia eutropha ^6^*  *(Alcaligenes eutrophus)*  *Ralstonia metallidurans*  *Ralstonia solanacearum*  *Rhodoferax ferrireducens*  *Rubrivivax gelatinosus* | 5.34  4.77  4.09  U, 7.5  U, 8.23  U, 9.67  5.98  7.25  U, 8.23  U, 5.89  U, 7.24  U, 6.82  5.80  U, ?  U, 4.57 | 2 (2,3) ^5^  2 (2,3) ^5^  2 (2,3) ^5^  2 (2,3)  4 (2,3)  3 (2,3)  2 (2,3)  3 (2,3)  2 (2,3)  1 (2)  1 (2)  1 (2)  1 (2)  2 (1,2)  2 (1,2) | 1  1  1  1  2  1  1  1  2  1  1  1  1  -  - | 1  1  1  -  -  1  -  -  -  -  -  -  -  -  -- | 4  4  4  3  6  5  3  4  4  2  2  2  2  2  2 | Ae  Ae  Ae  Ae  Ae  Ae  Ae  Ae  Ae  Ae, M  An  F, M  F, Sp  Ae, M  F, M  F, Aq |
| **Hydrogenophilales**  *Thiobacillus denitrificans*  **Methylophilales**  *Methylobacillus flagellatus*  **Neisseriacea**e  *Chromobacterium violaceum*  *Neisseria gonorrhoeae*  *Neisseria meningitidis*  *Vitreoscilla stercoraria*  **Nitrosomonadales**  *Nitrosomonas europea*  *Nitrosomona eutropha*  **Rhodocyclale**s  *Azoarcus sp.EbN1*  *Dechloromonas aromatica*  Globins present/total | U, 2.90  U, 2.78  4.75  2.15  2.18  ?  2.81  U, 2.71  4.73  U, 4.48 | 1 (2)  1 (2)  1 (2)  -  -  -  1 (3)  2 (3)  -  1 (2) | 1  1  1, (1)  -  -  (1)  -  -  -  - | -  -  2  -  -  -  -  -  1  - | 2  2  5  0  0  1  1  2  1  1  22/24 | F, M  Ae, Sp  F, M  Ae, HA  Ae, HA  Ae  Ae, M  M  F, Tr  F, M |
| **Gammaproteobacteria** |  |  |  |  |  |  |
| **Acidiothiobacillales**  *Acidiothiobacillus ferrooxidans*  **Alteromonadale**s  *Colwellia psychoerythrae*  *Idiomarina loihiensis*  *Microbulbifer degradans*  *Shewanellla amazonensis*  *Shewanella baltics*  *Shewanella denitrificans*  *Shewanella frigidimarina*  *Shewanella oneidensis* | U, 2.99  5.37  2.84  U, 5.04  4.23  5.01  4.45  4.76  5.13 | 2 (2,3)  -  1 (1)  2 (1,2)  2 (1,2)  2 (1,2)  2 (1,2)  2 (1,2)  1 (2) | -  1  -  -  (1)  1  -  1  - | 1  -  -  -  -  1  -  -  1 | 3  1  1  2  3  4  2  3  2 | ?  F, Sp  Ae, Sp  ?  F, M  F, Aq  F, Aq  F, M  F, M |
| **Enterobacteriales**  *Blochmannia floridanus*  *Buchnera aphidicola*  *Erwinia carotovara*  *Erwinia chrysanthemi*  *Escherischia coli*  *Photorhabdus luminescens*  *Shigella flexneri*  *Wigglesworthia glossinidia*  *Yersinia pestis*  *Yersinia pseudotuberculosis* | 0.70  0.64  5.06  U, 4.92  4.64  5.69  4.83  0.70  4.83  4.84 | -  -  -  -  -  -  -  -  -  - | -  -  1  1  1  1  1  -  1  1 | -  -  1  -  1  -  1  -  -  - | 0  0  2  1  2  1  2  0  1  1 | Sp  HA  F, M  F, HA  F, HA  F, HA  F, HA  HA  F, M  F, M |
| **Legionellales**  *Coxiella burnetii*  *Legionella pneumophila*  **Methylococcale**s  *Methylococcus capsulatus*  **Oceanospirillales**  *Chromohalobacter salexigens*  **Pasteurellales**  *Actinobacillus pleuropneumoniae*  *Haemophilus ducreyi*  *Haemophilus influenzae*  *Mannheimia succiniciproducens*  *Pasteurella multocida* | 2.03  3.41  3.30  U, 3.67  U, 2.19  1.70  1.83  2.31  2.26 | -  1 (1)  3 (1,2,3)  1 (2)  -  -  -  -  - | -  -  -  1  -  -  -  -  - | -  -  -  -  -  -  -  -  - | 0  1  3  2  0  0  0  0  0 | F, M  Ae, HA  Ae, M  F, Aq  F, HA  FAn  FAn  FAn  FAn |
| **Pseudomonadales**  *Acinetobacter sp. ADP1*  *Azotobacter vinelandii*  *Pseudomonas aeruginosa*  *Pseudomonas fluorescens*  *Pseudomonas putida*  *Pseudomonas stutzeri*  *Pseudomonas syringae*  *Psychrobacter sp. 273-4*  *Psychrobacter arcticum*  *Psychrobacter cryohalolentis* | 3.60  U, 5.42  6.26  7.07  6.18  U, ?  6.54  2.63  2.65  U, 3.07 | -  1 (2)  -  -  -  -  2 (1)  -  -  - | (1)  1  1, (1)  1  1  1  1  -  -  1 | -  1  -  -  -  -  -  -  -  - | 1  3  2  1  1  1  3  0  0  1 | Ae, M  Ae, M  Ae, M  Ae, M  Ae, M  Ae, M  Ae, M  Ae, M  Sp  M |
| **Salmonellacea**  *Salmonella enterica*  *Salmonella typhimurium* | 4.80  4.95 | -  - | 1  1 | -  - | 1  1 | F, HA  F, HA |
| **Thiotrichales**  *Francisella tularensis* | 1.89 | - | - | - | 0 | Ae, Aq |
| **Vibrionales**  *Photobacterium profundum*  *Vibrio cholerae*  *Vibrio fischeri*  *Vibrio parahaemolyticus*  *Vibrio vulnificus* | 6.40  4.03  4.28  5.17  5.20 | 1 (2)  -  -  -  - | 1, (1)  1  1  1, (1)  1 | -  -  -  -  1 | 3  1  1  2  2 | F, M  F, Aq  M  F, Aq  F, Aq |
| **Xanthomonadales**  *Xanthomonas axonopodis*  *Xanthomonas campestri*  *Xanthomonas oryzae*  *Xyllella fastidiosa*  Globins present/total | 5.27  5.08  4.94  2.73 | 1 (1)  1 (1)  1 (1)  - | -  -  -  2 | -  -  -  1 | 1  1  1  3  38/50 | Ae, HA  Ae, HA  Ae, HA  Ae, HA |
| **Deltaproteobacteria**  **Bdellovibrionales**  *Bdellovibrio bacteriovorus* | 3.78 | 2 (2,3) | - | - | 2 | Ae, M |
| **Desulfobacterales**  *Desulfotalea psychrophila* | 3.66 | - | - | 1 | 1 | An, Sp |
| **Desulfovibrionales**  *Desulfovibrio desulfuricans*  *Desulfovibrio vulgaris* | U, 3.87  3.77 | -  - | -  - | -  - | 0  0 | An, M  An, M |
| **Desulfuromonadales**  *Desulforomonas aceetoxidans Geobacter metallireducens*  *Geobacter sulfurreducens*  *Pelobacter propionicus* | U, ?  U, 4.07  3.81  U, 4.20 | -  -  -  - | -  -  -  - | 1  1  1  1 | 1  1  1  1 | An  An, Aq  An, M  An, M |
| **Myxococcales**  *Anaeromyxobacter dehalogenans*  *Myxococcus xanthus*  Globins present/total | 5.03  U, 9.17 | 1(1)  1 (1) | 1  - | -  1 | 2  1  7/9 | An, Tr  Ae, Tr |
| **Epsilonproteobacteria**  *Campylobacter coli*  *Campylobacter jejuni*  *Campylobacter lari*  *Campylobacter upsaliensis*  *Helicobacter hepaticus*  *Helicobacter pylorii*  *Thiomicrospira denitrificans*  *Wolinella succinogenes*  Globins present/total | U, 1.86  1.64  U, 1.56  U, 1.77  1.80  1.67  U, 2.21  2.11 | 1 (3)  1 (3)  1 (3)  1 (3)  1 (3)  -  2 (2,3)  - | (1)  (1)  (1)  -  -  -  -  - | -  -  -  -  -  -  1  - | 2  2  2  1  1  0  3  0  6/8 | MAe, M  MAe, M  MAe, M  M  Ae, HA  Ae, HA  An  MAe, HA |
| **Unclassified proteobacteria**  *Magnetococcus sp. MC-1*  Globins present/total | U, 4.63 | - | - | 2 | 2  98/126 | F, Aq |
| **Spirochaetes**  *Borrelia burgdorfi*  *Borrelia garinii*  *Leptospira interrogans*  *Treponema denticola*  *Treponema pallidum* | 1.52  0.99  4.66  2.84  1.14 | -  2 (2)  -  - | -  -  -  -  - | -  -  -  -  - | 0  0  2  0  0 | Ae, HA  HA  Ae, HA  An, HA  An, HA |

^1^ Based on NCBI Taxonomy Database ([www.ncbi.nlm.nih.gov/Taxonomy/Browser/wwwtax.cgi](http://www.ncbi.nlm.nih.gov/Taxonomy/Browser/wwwtax.cgi)).

^2^ From www.ncbi.nlm.nih.gov/genomes/MICROBES/Complete.html and [www.ncbi.nlm.nih.gov/](http://www.ncbi.nlm.nih.gov/) genomes/MICROBES/ InProgress.html.

^3^ The numbers in parenthese indicate the class (1, 2 or 3).

^4^ From Singleton and Sainsbury [144], Singleton [145] and <http://www.ncbi.nlm.nih.gov/genomes/lproks.cgi>. Abbreviations: Ae – aerobe; An – anaerobe; Aq – aquatic; F – facultative; HA – host associated; M – multiple habitats; MAe – microaerophilic; Sp – specialized; Th – thermophilic; Tr - terrestrial.

^5^ The species have similar Hbs; thus, only one representative sequences is provided in the alignment provided in Supplemental Data Fig.1.

^6^ The genus name *Ralstonia* (formerly *Alcaligenes*) was changed to *Wautersia* briefly, and is now *Cupriavidus* [58].

**TABLE 4. Identified and putative globins in eukaryote genomes^1^.**

| Genome | **Genome**  **Size, Mbp ^2^** | **Identifier** | **Size and**  **GD (aa)** | Type ofglobin |
| --- | --- | --- | --- | --- |
| *Anopheles gambiae*  (Arthropoda; Insecta; Diptera) | 278 | gi|58377826|XP_308074.2|ENSANGP19788  gi|58377380|XP_309583.2|ENSANGP22287  gi|31198253|XP_308074|ENSANGP19788  gi|57914327|XP_555006.1|ENSANGP26474 | 150, 26 - 140  192, 51 - 190  215, 110-215  182, 4 - 146 | PG  PG  PG  PG |
| *Arabidopsis thaliana*  (Viridiplantae; Streptophyta;  Embryophyta) | 123.5 | gi|15226675|NP_179204  gi|15228313||NP_187663  gi|18418064||NP_567901  gi|7486404|T04457 | 160, 7 - 156  158, 4 - 154  175, 14 – 145  671, 15-164 | GLB1; NsHb  GLB2; NsHb  GLB3; 2/2Hb2  2/2HbGD |
| *Caenorhabditis briggsae/elegans*  (Nematoda; Rhabditida) | 97 | 33 globins; see Table 3 |  |  |
| *Chlamydomonas reinhardtii*  (Viridiplantae; Chlorophyta;  Volvocales) | ~100 | 160981  160982  157690  168933  168934  168800  153190    168904 | 136  147  231, 75-185  651, 54-155  476, 2-120  432, 47-163  837, 42-148; 180-299  347, 113-224 | 2/2Hb1  2/2Hb1  2/2Hb1  2/2Hb1  2/2Hb1  2/2Hb1  2/2Hb1  2/2Hb1  2/2Hb1 |
| *Ciona intestinalis*  (Chordata; Urochordata;  Ascidiacea) | 155 | gi|34364574|emb|CAD89600.1  gi|34364572|emb|CAD68147.1  gi|34364568|emb|CAD68145.1  gi|34364570|emb|CAD68146.1 | 293, 135-247  153  156  159 | GD  SDgb  SDgb  SDgb |
| *Cyanidioschyzon merolae*  (Rhodophyta; Bangiophyceae) | 16.52 | gnl|CMER|CMR319C | 185, 5-124 | SDgb |
| *Danio (Brachydanio) rerio*  (Vertebrata; Euteleostomi;  Teleostei) | 1571 | gi|15387697|NP_571928  gi|21263503|NP_694484  gi|20138124|Q90487|HBA_BRARE  gi|33468638|CAE30440  gi|33468642|CAE30444  gi|37606095|CAE48980  gi|37606099|CAE48989  gi|18858329|NP_571095  gi|18858331|NP_571096  gi|33636711|NP_891985  -  gi|34098940|NP_898889  gi|3676659|NP_571834  gi|47086345|NP_998011  gi|33089399|AAP93667  gi|59276060|NP_001012261  gi|55977280|MYG_BRARE | 159  174  143  143  143  143  143  148  148  143  143  143 ^4^  147  147  147 ^4^  200  147 | Ngb  Cygb  Alpha A1  Alpha  Alpha  Alpha  Alpha  Beta a1  Beta a2  Em alpha 1  Em alpha 2  Em alpha 3  Em beta 1  Em beta 2  Em beta 3  GbX  Mb |
| *Dictyostelium discoideum*  (Mycetozoa; Dictyostelida) | 34 | gi|5821408|BAA83810.1  gi|5821410|BAA83811.1 | 397, 2-142  423, 1-141 | FHb  FHb |
| *Drosophila melanogaster*  (Arthropoda; Insecta; Diptera) | 157 | gi|24647276|NP_732081|CG9734  gi|28571530|NP_649597|CG14675  gi|45550703|NP_649669|CG15180 | 153, 1-148  195, 33-168  209, 3-122 | PG  PG  PG |
| *Drosophila psudoobscura*  (Arthropoda; Insecta; Diptera) | 156 | gi|54639008|EAL28410|GA21995  gi|54638691|EAL28093|GA11381  gi|54638692|EAL28094|GA13167 | 152  641, 6-141  130 | PG  PG  PG |
| *Encephalitozoon cuniculi*  (Fungi; Microsporidia) | 2.9 | No globins |  |  |
| *Entamoeba histolytica* (Apicomplexa; Entamoebidae) | 18-20 | No globins |  |  |
| *Fugu (Takifugu) rubripes*  (Vertebrata; Euteleostomi;  Actinoperigii; Teleostei) | 329 | SINFRUP00000152742  SINFRUP000000147528  - ^5^  SINFRUP00000147719  gi|29568121|AAO61492  gi|29568123|AAO61494  gi|29568122|AAO61493  SINFRUP00000139232 | 159  139  142  144  143  141  147  145 | Ngb  Cygb  Alpyha 1  Alpha 2  Alpha 3  Alpha 4  Beta  Mb |
| *Gallus gallus*  (Vertebrata; Euteleostomi; Archosauria; Aves) | 1054 | gi|56368392|CAG25721  gi|56368400|CAH23229  gi|122315|P02001|HBAD_CHICK  gi|52138655|NP_001004376  gi|63035|CAA23678  gi|52138651|NP_001004374  gi|49169791|NP_990820  gi|50731454|XP_425673  gi|52138683|NP_001004390  gi|50728806|XP_416292  gi|56961659|NP_001008786 | 160  175  141  142  142  142  147  188, 42-188  147  153  151 | Ngb  Cygb  Alpha D  Alpha  Alpha  Em alpha  Beta  Epsilon  Rho  Mb  GlbE |
| *Giardia lamblia (intestinalis)*  (Diplomonadida; Hexamitidae) | 12 | gi|27981493|AAM94640.1 | 458, 4-150 | FHb |
| *Homo sapiens*  (Vertebrata; Mammalia; Eutheria;  Primates) | 2910 | gi|32171399|Q9NPG2|NGB_HUMAN  gi|21263504|Q8WWM9|CYGB_HUMAN  gi|57013850|P69905|HBA_HUMAN  gi|122330|P09105|HBAT_HUMAN  gi|122335|P02008|HBAZ_HUMAN  gi|51510893|NP_001003938  gi|56749856|P68871|HBB_HUMAN  gi|56749860|P69891|HBG1_HUMAN  gi|56749861|P69892|HBG2_HUMAN  gi|122713|P02042|HBD_HUMAN  gi|122726|P02100|HBE_HUMAN  gi|127661|P02144|MYG_HUMAN | 151  190  142  142  142  141  147  147  147  147  147  154 | Ngb  Cygb  Alpha  Theta  Zeta  Mu  Beta  Gamma A  Gamma G  Delta  Epsilon  Mb |
| *Mus musculus*  (Chordata; Vertebrata;  Mammalia; Eutheria;  Rodentia) | 2932 | gi|11967939|NP_071859  gi|21263506|NP_084482  gi|122441|P01942|HBA_MOUSE  gi|12846939|BAB27370  gi|309259|gb|AAA37702  gi|33239411|NP_778165  gi|31982300|NP_032246.2  gi|122513|P02088  gi|122526|P02089  gi|122550|P04444  gi|122510|P04443  gi|6680177|NP_032247  gi|127676|P04247|MYG_MOUSE | 151  190, 19-170  142  142  142  145  147  147  147  147  147  147  154 | Ngb  Cygb  Alpha  Alpha  Zeta  Theta 1  Beta major  Beta 1  Beta 2  Beta H1  Beta H0  Epsilon Y  Mb |
| *Oryza sativa ssp. indica*  (Viridiplantae; Embryophyta;  Tracheophyta; Spermatophyta;  Magnoliophyta; Liliopsida) | 420 | gi|17366135|O04986|HBL1_ORYSA  gi|17432965|O04985|HBL2_ORYSA  gi|22001645|Q94FT8|HBL3_ORYSA  gi|22001644|Q94FT7|HBL4_ORYSA  gi|50932383|XP_475719  gi|50725383|BAD32857 | 166  169  169  167  145  172 | NsHb  NsHb  NsHb  NsHb  NsHb  2/2Hb |
| *Phaeodactylum tricornutum*  (Bacillariophyta) | ~13 | PTMM 03909  PTMM 05212 | 157  167 | SDgb  2/2Hb |
| *Plasmodium falciparum*  (Alveolata; Apicomplexa;  Haemosporida) | 23 | No globins |  |  |
| *Rattus norvegicus*  (Vertebrata; Mammalia; Eutheria;  Rodentia) | 2571 | gi|19923711|NP_203523.2  gi|18543351|NP_570100.1  gi|122477|P01946|HBA_RAT  gi|27668422|XP_213262.1  gi|34871150|XP_220266.2  gi|27668426|XP_213268.1  gi|122514|P02091|HBB1_RAT  gi|122529|P11517|HBB2_RAT  gi|40445397|NP_942071.1  gi|423675|S34719  gi|27678778|XP_215029.1  gi|4539553|CAA39766  gi|3367728|CAA39767  gi|11024650|NP_067599.1 | 151  190, 25-160  142  140  142  142  147  147  147  147  147  147  147  154 | Ngb  Cygb  Alpha  Alpha  Theta  Zeta  Beta major  Beta minor  Zero beta1  Zero beta 2  Epsilon 1  Epsilon 2  Epsilon 3  Mb |
| *Strongylocentrotus purpuratus* (Echinodermata; Echinoidea) | ~870 | gi|72169631|XP_795670.1 | 175 | GlbX or Cygb |
| *Tetraodon nigroviridis*  (Vertebrata; Euteleostomi;  Actinoperigii; Teleostei) | 340 ^3^ | gi|32171394|Q90W04  gi|47219636|CAG02681  gi|47226107|CAG04481.1  gi|47220053|CAG12201.1  gi|47220054|CAG12202.1  gi|42661496|CAF31356  gi|56368394|CAG25722.1 | 159  174  143  144  143  146  205 | Ngb  Cygb  Alpha  Alpha  Alpha  Mb  GbX |
| *Thalassiosira pseudonana*  (Bacillariophyta) | ~34 | 120695|scaffold_137:50991:51371  126138|scaffold_18:64010:64489 | 127  160, 26-150 | SDgb  2/2Hb |
| *Trypanosoma brucei* | ~35 | No globins |  |  |
| *Xenopus (Silurana) tropicalis*  (Vertebrata; Euteleostomi;  Amphibia) | 1630 | gi|62146705|CAG25550  gi|55742013|NP_001006870  gi|122509|P07428|HBA_XENTR  gi|52346090|NP_001005092  gi|122303|P08422|HBA3_XENTR  gi|45360951|NP_988859|HBB1_XENTR  gi|122537|P08423|HBB2_XENTR  gi|52346092|NP_001005089  gi|52345706|NP_001004899  gi|85881|B25929  gi|58332516|NP_001011196 | 164  179  142  142  142  147  147  147  147  146  200 | Ngb  Cygb  Alpha  Alpha  Alpha 3  Beta 1  Beta 2  Beta  Beta  Beta  GbX |

^1^ Cygb – 3/3 cytoglobin; Em – embryonic; FHb – flavohemoglobin, chimeric proteins (~400aa) comprising a 3/3 N-terminal globin and a flavin reductase domain; GbX – new globin found in fish and amphibians [40]; GCS – globin-coupled sensors: chimeric proteins (~300 to >700aa) comprising a 3/3 N-terminal globin domain and a variable C-terminal portion; GD – globin domain; GlbE – eye globin [39]; Hb – hemoglobin; SHb – 3/3 symbiotic Hbs of legumes and other plants; Mb – myoglobin; Ngb – 3/3 neuroglobin; NsHbs – 3/3 nonsymbiotic plant Hbs; PG – putative globin, defined by manual alignment (>100 to 200aa), iidentified by CD or FUGUE search; Pgb – protoglobin, single domain 3/3 globin related to the N-terminal domain of GCSs; PGD – putative globin domain (>100 – 200aa) within a larger protein; SDgb – 3/3 single domain globin (~140aa) aligning with the N-terminal of FHbs; 2/2Hbs – “truncated” Hbs that are not necessarily shorter than ~140aa, which possess a characteristic 2-over-2 helical fold. Assigned sequences with <100aa were not considered.

^2^ From Gregory, T.R. 2005.  Animal Genome Size Databas*e* (http://www.genomesize.com.)

^3^ From ref [146].

^4^ The 123aa embryonic alpha 3 and 143aa embryonic beta 3 sequences provided in the NCBI, lack 20aa and the N-terminal

4aa [61] .

^5^ From Gillemans et al. [62].

**TABLE 5. The putative globin orthologs of *Caenorhabditis briggsae* AND *C. elegans* ^1^.**

|  | Protein/Gene | Identifier | Size and  GD (aa) | Type of  globin | Protein/Gene | Identifier | Size,  GD (aa) | Type of  globin |
| --- | --- | --- | --- | --- | --- | --- | --- | --- |
| 1 | CBP07494/CBG0687 | gi|39585062|CAE62713 | 160 | PG | CE36418/ZK637.13 | gi|42734289|CAA77458 | 159 | PG |
| 2 | CBP03870/CBG16082 | gi|39596154|CAE6979 | 218, 13 -207 | Cygb | CE01012/F49E2.4 | gi|3877381|CAA86423 | 216, 13 - 205 | Cygb |
| 3 | CBP00622/CBG03023 | gi|39597385|CAE59614 | 266, 31 - 215 | PG | CE01528/C52A11.2 | gi|3875223|CAA8676 | 266, 31 - 215 | PG |
| 4 | CBP09240/CBG13047 | gi|39596023|CAE67526 | 244, 48 - 207 | PG | CE01816/C28F5.2 | - ^2^ | 217, 52 - 211 | PG |
| 5 | CBP00206/CBG00571 | gi|39586983|CAE62918 | 224, 23 - 178 | PG | CE02371/W01C9.5 | gi|3880381|CAA90269 | 224, 23 - 178 | PG |
| 6 | CBP00844/CBG02622 | gi|39597062|CAE59289 | 198, 27 - 187 | PG | CE02514/C29F5.7 | gi|17532151|NP_495268 | 198, 27 - 187 | PG |
| 7 | CBP00055/ BG00138 | gi|39588027|CAE57258 | 209, 27 - 183 | Ngb | CE03233/F19H6.2 | gi|3876112|CAA92164 | 231, 49 - 205 | Ngb |
| 8 | CBP15914/CBG07422 | gi|39596505|CAE63124 | 353, 95 - 241 | PG | CE03523/R01E6.6 | gi|3878772|CAA92187 | 311, 95 – 241 | PG |
| 9 | CBP03989/CBG16720 | gi|39594123|CAE70233 | 314, 61 - 223 | Cygb | CE04582/F46C8.7 | gi|17567747|NP_509275 | 322, 60 - 222 | PG |
| 10 | CBP01576/CBG05809 | gi|39593545|CAE61837 | 342, 160-321 | PG | CE04843/R13A1.8 | gi|1065933|AAA81477 | 342, 160-321 | PG |
| 11 | CBP05578/CBG23115 | gi|39591955|CAE75175 | 206, 33 - 189 | PG | CE05237/C06H2.5 | gi|3874041|CAA99771 | 209, 33 - 189 | PG |
| 12 | CBP02907/CBG11881 | gi|39582475|CAE66566 | 377, 206-361 | Cygb | CE05316/C26C6.7 | - ^3^ | 370, 194-353 | Cygb |
| 13 | CBP02911/CBG11915 | gi|39586180|CAE6659 | 237, 68 - 231 | Cygb | CE05911/F52A8.4 | gi|3877400|CAA95823 | 238, 69 - 232 | Cygb |
| 14 | CBP02022/CBG08252 | gi|39582084|CAE63727 | 181, 24 - 170 | PG | CE06490/T22C1.2 | gi|3880027|CAA99921 | 183, 26 - 172 | PG |
| 15 | cbp22220/CBG09371 | gi|39593145|CAE64614 | 364, 88 - 248 | Cygb | CE36271/C18C4.1 | - ^4^ | 258, 74 - 234 | PG |
| 16 | CBP21478/CBG04577 | gi|39589868|CAE6086 | 330, 73-245 | PG | CE12774/R11H6.3 | gi|3879147|CAB07647 | 387, 133-305 | Cygb |
| 17 | CBP02580/CBG10551 | gi|39580931|CAE65561 | 278, 6 - 168 | PG | CE17437/C23H5.2b | gi|32453028|AAP82661 | 278, 6 - 168 | Ngb |
| 18 | CBP13608/CBG24799 | gi|39579284|CAE56950 | 147 | Ngb | CE18207/T06A1.3 | - ^5^ | 136 | PG |
| 19 | CBP04921/CBG21021 | gi|39591498|CAE73552 | 216, 37 - 201 | PG | CE19039/Y17G7B.6 | gi|3947607|CAA19459 | 216, 37 - 201 | Cygb |
| 20 | CBP01805/CBG07112 | gi|39586983|CAE62918 | 169 | PG | CE19638/Y57G7A.9 | gi|17537679|NP_493944 | 169 | PG |
| 21 | CBP07717/CBG07681 | gi|39596678|CAE63297 | 217, 46 - 201 | PG | CE21369/Y15E3A.2 | gi|6425266|CAB60330 | 210, 46 - 199 | PG |
| 22 | CBP02078/CBG08670 | gi|39583978|CAE64068 | 246, 114-246 | PG | CE36044/Y58A7A.6 | gi|17566038|NP_504363 | 230, 91 - 230 | PG |
| 23 | CBP02293/CBG09369 | gi|39593143|CAE64612 | 390, 197-354 | PG | CE29586/C18C4.9 | gi|17558104|NP_504469 | 389, 198-354 | PG |
| 24 | CBP02390/CBG09511 | gi|39593254|CAE64724 | 322, 142-303 | Ngb | CE30683/R90.5 | gi|20803774|CAD31696 | 322, 142-303 | PG |
| 25 | CBP14781/CBG03635 | gi|39583310|CAE60102 | 235, 45 - 191 | PG | CE30888/C36E8.2 | gi|25146396|NP_497802 | 254, 42 - 188 | PG |
| 26 | CBP15597/CBG06424 | gi|39592734|CAE62348 | 542, 371-539 | PG | CE31132/Y75B7AL.1 | gi|25148647|NP_503405 | 542, 373-539 | PG |
| 27 | CBP12391/CBG23302 | gi|39592105|CAE75325 | 234, 35 - 191 | PG | CE31930/F35B12.8 | gi|25809206|CAD57695 | 234, 35 - 191 | PG |
| 28 | CBP19339/CBG18593 | gi|39582852|CAE71628 | 227, 75 - 227 | Cygb | CE34541/F21A3.6 | gi|32697997|CAB04152 | 236, 84 - 236 | Cygb |
| 29 | CBP07299/CBG05824 | gi|39593557|CAE61849 | 229, 26 - 208 | PG | CE34658/C06E4.7 | gi|33285222|AAA82476 | 230, 27 - 209 | PG |
| 30 | CBP06748/CBG04428 | gi|39581850|CAE60743 | 220, 24 - 186 | Ngb | CE34888/F56C4.3 | gi|33300043|CAE17840 | 214, 10 - 172 | PG |
| 31 | CBP23619/CBG17640 | gi|39594987|CAE70855 | 112 | Cygb | CE34964/R102.9 | gi|33300310|CAE17922 | 196, 16 - 189 | PG |
| 32 | CBP14482/CBG02965 | gi|39597340|CAE59568 ^6^ | 325, 9 - 173 | PG | CE35828/C09H10.8 | gi|7495791|T19164 ^6^ | 344, 26 - 190 | PG |
| 33 | CBP02870/CBG11737 | gi|39584054|CAE66460 ^7^ | 278, 12-181 | Ngb | CE23430/Y22D7AR.5 | - ^8^ | 272, 6 - 175 | Ngb |

^1^ The protein and gene assignments are from WormBase Release WS139 ([www.wormbase.org/](http://www.wormbase.org/)). Abbreviations: Cygb – 3/3 cytoglobin; GD – globin domain; Ngb – 3/3 neuroglobin; PG – putative globin, defined by manual alignment (>100 to 200aa), identified by CD or FUGUE search.

^2^ GenBank sequence gi|17532131|NP_495572 was corrected (D. Hoogewijs and J. Vanfleteren, unpublished results).

^3^ GenBank sequence gi|3874041|CAA96599 was corrected (D. Hoogewijs and J. Vanfleteren, unpublished results).

^4^ GenBank sequence gi|17558092|NP_504466 was corrected (D. Hoogewijs and J. Vanfleteren, unpublished results).

^5^ GenBank sequence gi|7505252|T33271 was corrected (D. Hoogewijs and J. Vanfleteren, unpublished results).

^6^ Although the *C. elegans* sequence is a likely globin according to FUGUE (Z = 4.69), the *C. briggsae* sequence is not identified as a globin.

^7^ GenBank sequence gi|39584054|CAE66460 was corrected (D. Hoogewijs and J.. Vanfleteren, unpublished results).

^8^ GenBank sequence gi|17555588|NP_497441 was corrected (D. Hoogewijs and J. Vanfleteren, unpublished results).

**TABLE 6. Identified and putative globins in fungal genomes^1^.**

| Genome | **Genome**  **size, Mbp ^2^** | **Identifiers** | **Size and**  **GD (aa)** | Type ofglobin |
| --- | --- | --- | --- | --- |
| Ascomycota  Pezizomycotina |  |  |  |  |
| *Aspergillus fumigatus* | ~30 | gi|50788082|CAE17673  gi|70981999|XP_746528  gi|70983767|XP_747310 | 413, 3-154  413, 3-154  433, 31-154 | FHb  FHb  FHb |
| *Aspergillus nidulans* | ~31 | gi|49092396|XP_407659|AN3522.2  gi|40742231|EAA61421|AN7169.2 | 426, 25-166  410, 2-145 | FHb  FHb |
| *Aspergillus niger* | ~36 | gi|60417044|CAF32309.1  gi|58864970|CAF25490.1 | 412, 3-154  455, 42-197 | FHb  FHb |
| *Aspergillus oryzae* |  | gi|60417040|CAF32307.1 | 147 | SDgb |
| *Giberella zeae*  *(Fusarium gramineatum)* | ~40 | gi|42550399|EAA73242|FG04458.1  gi|42547868|EAA70711|FG00765.1 | 415, 3-151  457, 3-149 | FHb  FHb |
| *Magnaporthe grisea* | ~40 | gi|38101598|EAA48540|MG00198.4 | 447, 26-169 | FHb |
| Saccharomycotina |  |  |  |  |
| *Candida albicans* | 16 | gi|46432319|EAK91807.1  gi|46432322|EAK91810.1  gi|46441212|EAL00511.1  gi|46433276|EAK92722.1 | 398, 10-146  400, 17-155  400, 17-155  563, 298-437 | FHb  FHb  FHb  CDFHb* |
| *Candida glabrata* | 12.3 | gi|50293309|XP_449066  gi|50291201|XP_448033 | 398, 1-138  432, 124-267 | FHb  CDFHb* |
| *Debaryomyces hansenii* | 12.2 | gi|50304451|XP_452175.1 gi|50428041|XP_462633.1  gi|50428015|XP_462620.1 | 395, 5-145  401, 14-151  403, 14-149 | FHb  FHb  FHb |
| *Eremothecium (Ashbya)*  *gossypii* | 9.2 | gi|45185029|NP_982746 | 436, 184-339 | CDFHb* |
| *Kluyveromyces lactis* | 10.6 | gi|50304451|XP_452175  gi|50307891|XP_453939 | 395, 1-134  430, 181-323 | FHb  CDFHb* |
| *Kluyveromyces waltii* | 10.6 | Kwal_9681  Kwal_22190  Kwal_4395  Kwal_24852 | 396, 1-138  421, 171-314  460, 202-352  543, 173-315 | FHb  CDFHb*  CDFHb*  CDFHb* |
| *Saccharomyces bayanus* | 11.54 ^3^ | ORFP:9664  ORFP:20532 | 399, 1-138  424, 171-303 | FHb  FHb |
| *Saccharomyces cerevisiae* | 12.16 ^3^ | gi|6321673|NP_011750.1  gi|6324095|NP_014165.1 | 399, 1-148  426, 154-302 | FHb  CDFHb* |
| *Saccharomyces mikatae* | 12.12 ^3^ | ORFP:9249  ORFP:18051 | 399, 1-138  410, 135-290 | FHb  CDFHb* |
| *Saccharomyces paradoxus* | 11.75 ^3^ | ORFP:9231  ORFP:18484 | 399, 1-138  426, 155-306 | FHb  CDFHb* |
| *Yarrowia lipolytica* | 20.5 | gi|50548235|XP_502088  gi|50550817|XP_502881  gi|50543406|XP_499869 | 374, 1-138  463, 186-325  471, 194-333 | FHb  CDFHb*  CDFHb* |
| Schizosaccharomycetes |  |  |  |  |
| *Schizosaccharomyces pombe* | 13.8 | gi|19115929|NP_595017.1 | 427, 31-178 | FHb |
| Sordariomycetes |  |  |  |  |
| *Neurospora crassa* | ~43 | gi|32405610|XP_323418|NCU10051.1  gi|32417616|XP_329286|NCU02096.1 | 418, 3-151  537, 3-149 | FHb  FHb |
| **Basidiomycota**  Hymenomycetes |  |  |  |  |
| *Cryptococcus neoformans* | ~24 | gi|50259618|EAL22289.1 | 504, 80-212 | FHb |
| *Ustilago maydis (zeae)* | U, ~20 | No globins |  |  |

^1^ FHb – flavohemoglobin, chimeric proteins (~400aa) comprising a 3/3 N-terminal globin and a flavin reductase domain; CDFHb – FHbs ranging from 410 to 563aa, with centrally located globin domains; GD – globin domain.

^2^ From Kullman B, Tamm H, Kullman K: Fungal Genome Size Database, 200*5* (<http://www.zbi.ee/fungal->genomesize).

^3^ From Kellis et al. [147].
