# Supplementary material for: A phylogenomic profile of globins
Source: BMC Evol Biol. 2006 Apr 7;6:31. doi: 10.1186/1471-2148-6-31 (PMC1457004; doi:10.1186/1471-2148-6-31)
Supplement: Additional File 2 — Fig. 1. Alignment of selected globin sequences. Fig. 2. Alignment of putative globins from Caenorhabditis briggsae and C. elegans. Fig. 3. Alignment of 175 representative globin sequences used in the construction of the Bayesian phylogenetic tree shown in Fig. 5. [file 1471-2148-6-31-S2.doc]

**Supporting Data**

**Fig.1**

**Mb fold**

**nn...................................................Cccc...................................................................F**

# aaaaaaaaaAaaAAaaAa..........bbbbbBbbBBbbBBbbcCccccc..Dddd........dddddddeeeEeeEEeeEEeeEeeEEe..............FffFfffFFf........G.....ggggGggGggGGGgGGggg.............hhhhhhHHhhHHhhHhhhHhhhhhhh

**VLSEGEWQLVLHVWAKVEA.........DVAGHGQDILIRLFKSHPETLEK..FDRF....khlkteaeMKASEDLKKHGVTVLTALGAILKKK......GHHEAELKPLAQSHATKHK.....I.....PIKYLEFISEAIIHVLHSRHP........GDFGADAQGAMNKALELFRKDIAAKYKelgyqg**

**.........|..||..|................|..||..||...|.......|..|..................|..||..||..|..||...............|..|...||.........|.........|..|..|||.||......................||..||..|...|......**.....

**Danio rerio**

**gi|15387697|NP_571928 159aa Ngb**

**KLSEKDKGLIRDSWESLGK.........NKVPHGIVLFTRLFELDPALLTL..FSYS....TNCGDAPECLSSPEFLEHVTKVMLVIDAAVSHL...DDLHTLEDFLLNLGRKHQAVG......V.....NTQSFALVGESLLYMLQSSLG........PAYTTSLRQAWLTMYSIVVSAMTRGWAKNGEHK.**

**.........|..||..|................|..||..||...|.......|..|..................|..||..||..|..||...............|..|...||.........|.........|..|..|||.||......................||..||..|...|............**

**gi|21263503|NP_694484 174aa Cygb (15-171)**

**SLTEEDVCVIQDTWKPVYA.........ERDNAGVAVLVRFFTNFPSAKQY..FEHF....RELQDPAEMQQNAQLKKHGQRVLNALNTLVENL...RDADKLNTIFNQMGKSHALRHK.....V.....DPVYFKILAGVILEVLVEAFP.......QCFSPAEVQSSWSKLMGILYWQMNRVYAEVGWEN.**

**.........|..||..|................|..||..||...|.......|..|..................|..||..||..|..||...............|..|...||.........|.........|..|..|||.||......................||..||..|...|............**

**gi|18858787|NP_571332 143aa Alpha 1**

**slsdtdkavvkaiwakisp.........kadeigaealarmltvypqtkty..fshw.........adlspgsgpvkkhgktimgavgeaiski......ddlvgglaalselhafklr.....v.....dpanfkilshnvivviamlfp........adftpevhvsvdkffnnlalalsekyr.......**

**.........|..||..|................|..||..||...|.......|..|..................|..||..||..|..||...............|..|...||.........|.........|..|..|||.||......................||..||..|...|............**

**gi|18858329|NP_571095 148aa Beta A1**

**ewtdaertailglwgklni...........deigpqalsrclivypwtqry..fatf....gnlsspaaimgnpkvaahgrtvmggleraiknm......dnvkntyaalsvmhseklh.....v.....dpdnfrlladcitvcaamkfg.......qagfnadvqeawqkflavvvsalcrqyh.......**

**.........|..||..|................|..||..||...|.......|..|..................|..||..||..|..||...............|..|...||.........|.........|..|..|||.||......................||..||..|...|............**

**gi|33636711|NP_891985 143aa Alpha embryonic 1**

**slsakdkaavktlwakiag.........kaddighdalsrmlivypqtkty..fshw.........kdlspgsapvrkhgktvmggvaeavski......ddlnagllnlselhafqlr.....v.....dpanfkilshnilvvlatlfp........adftpeahvamdkflsalalamsekyr.......**

**.........|..||..|................|..||..||...|.......|..|..................|..||..||..|..||...............|..|...||.........|.........|..|..|||.||......................||..||..|...|............**

**Alpha embryonic 2 143aa (from Brownlie et al., 2004)**

**slsakdkaavkalwakia.........gkaddighdalsrmlivypqtkty..fshw.........kdlspgsapvrkhgktvmggvaeavsRi......ddltagllnlselhafqlr.....v.....dpanfkilshnilvvlatlfp........adftpeahvamdkflsalalamTekyr.......**

**.........|..||..|................|..||..||...|.......|..|..................|..||..||..|..||...............|..|...||.........|.........|..|..|||.||......................||..||..|...|............**

**Alpha embryonic-3 143aa (from Brownlie et al., 2004) (gi|34098940|NP_898889 123aa is incorrect)**

**slsakdkanvkaffdkvap.........kaeeigretlsrtlfvypqtkty..fspl.........adlspnspqvkkhgttvingvltavglm......ddlkgglltlselhafmlr.....v.....dpanfkiinhnllvsLAMMFP........DDFTPEVHVSVDKFlaqvslalsekyr.......**

**.........|..||..|................|..||..||...|.......|..|..................|..||..||..|..||...............|..|...||.........|.........|..|..|||.||......................||..||..|...|............**

**gi|18858353|NP_571834 147aa Beta-globin embryonic 1**

**vwtdfekatiqdifakady...........dvigpqalarclivypwtqry..fakf....gnlynaaailgnpmvaahgktvlkglelavknm......dnikatyadlsvlhseklh.....v.....dpdnfrlladcltivvaaqmg........agftpevqaafqkfiavavsalgrqyh.......**

**.........|..||..|................|..||..||...|.......|..|..................|..||..||..|..||...............|..|...||.........|.........|..|..|||.||......................||..||..|...|............**

**gi|47086345|NP_998011.1 147aa Beta embryonic 2**

**awtaeerafiqdifsklny...........eeagpkalqralivypwtqry..fgsf....gnlynaeaiinnpkvaahgtvvlhgldramknm......ddikntyaelsvlhseklh.....v.....dpdnfrlladcltiviastmg........aaftadmqaawqkflavvvsalqrqyh.......**

**.........|..||..|................|..||..||...|.......|..|..................|..||..||..|..||...............|..|...||.........|.........|..|..|||.||......................||..||..|...|............**

**Beta embryonic 3 147aa (gi|33089399|AAP93667.1 143aa corrected)**

**VWtaeeraaiqnifakldf...........esvgletltrclvvypwtqry..fggf....gnlynteaimanpkvkahgvvvlkglekalnnm......dnikstfaslselhseklq.....v.....dpgnfrlladcltvviatrmr........seftpdiqaawqkflsvvvsalrrqyf.......**

**.........|..||..|................|..||..||...|.......|..|..................|..||..||..|..||...............|..|...||.........|.........|..|..|||.||......................||..||..|...|............**

**gi|55977280|Q6VN46 147aa Mb**

**...madhdlvlkcwgavea.........dyaanggevlnrlfkeypdtlkl..fpkf.....sgisqgdlagspavaahgatvlkklgellkak......gdhaallkplanthanihk.....v.....alnnfrlitevlvkvmaekag......ldaagqgalrrvmdavigdidgyykeigfag.....**

**.........|..||..|................|..||..||...|.......|..|..................|..||..||..|..||...............|..|...||.........|.........|..|..|||.||......................||..||..|...|............**

**gi|59276060|NP_001012261.1 200aa HbX (27-181)**

**glttnhirlikeswrliqe.........diakvgiimfvrlfethpeckdv..fflf....rdvedlerlrtsrelrahglrvmsfieksvarl...dqlerletlalelgkshyryn......a.....ppkyygyvgaeficavrpilk........drwtpeleeawktlfqyvtsimregfleeernkR**

**.........|..||..|................|..||..||...|.......|..|..................|..||..||..|..||...............|..|...||.........|.........|..|..|||.||......................||..||..|...|............**

**Fugu (Takifugu) rubripes**

**SINFRUP00000152742 159aa Ngb**

**KLSSKDKELIRGSWDSLGK.........NKVPHGVIMFSRLFELDPELLSL..FH.Y...TTNCGSTQDCLSSPEFLEHVTKVMLVIDAAVSHL...DDLHSLEDFLLNLGRKHQAVG......V.....NPQSFATVGESLLYMLQCSLG........QAYTASLRQAWLNMYSVVVAAMSRGWAKNGEDKA**

**.........|..||..|................|..||..||...|.......|..|..................|..||..||..|..||...............|..|...||.........|.........|..|..|||.||......................||..||..|...|............**

**SINFRUP00000147528 178aa (18- Cygb**

**PLTDKEKVMIQDSWAVVFQ.........SCDDAGVAILVRLFVSFPSSKQL..FKDF....KDIEEPEEMQRSIQLRKHAHRVMTTINTLVENL...DDADAMASALKSVGRAHALRHK.....V.....DPKYFKILSGVILEVLGEAFT........EIITAEVASAWTKLLANMCCAVAAVYEEAGWTKL**

**.........|..||..|................|..||..||...|.......|..|..................|..||..||..|..||...............|..|...||.........|.........|..|..|||.||......................||..||..|...|............**

**SINFRUP00000147719 144aa Alpha**

**SLSAKDKDTVRLFWGKLAP.........KREEVGANALCRLLSVYPQTKTY..FAHW.........KDQSPTSASAKKHGITIMNAIGDSVSKI......DDLKGGLFNLSELHAFTLR.....V.....DPANFKLLAHCIMVEIGIMYP........TEFTPEVHVAVDKFLASVALALAEKYR.......**

**.........|..||..|................|..||..||...|.......|..|..................|..||..||..|..||...............|..|...||.........|.........|..|..|||.||......................||..||..|...|............**

**gi|29568122|AAO61493| 147aa Beta**

**ewtdqertiisnifstldy...........edvgskslirclivypwtqry..fagf....gnlynaeaiknnpniakhgvtvlhgldravknm......dniketykelselhseklh.....v.....dpdnfkllsdcltivvatkmg........skftpeiqatfqkflavvvsalgrqyh.......**

**.........|..||..|................|..||..||...|.......|..|..................|..||..||..|..||...............|..|...||.........|.........|..|..|||.||......................||..||..|...|............**

**SINFRUP00000139232 145aa Mb**

**....ADFETVLKFWGPVEA.........DYGAHGGIVLTRLFTENPETQKL..FPKF.....AGITQSDLAGNAAVSAHGATVLKKLGELLKAK......GNHAALLQPLANTHATKHK.....I.....PINNFKLIAEVIGKVMEEKAG..........LDAAGQQALKNVMATIIADIDVTYKDLGFS..**

**.........|..||..|................|..||..||...|.......|..|..................|..||..||..|..||...............|..|...||.........|.........|..|..|||.||......................||..||..|...|............**

**Gallus gallus**

**gi|56368392|CAG25721.1 160 aa Ngb (4-160)**

**mlsrtqqalireswrrvsg.........spvqhgvvlfsrlfdldpdllpl..fq.y..nckrfaspqeclaapefldhirkvmlvidaavshl...edlpcleeylcnlgkkhqavg......v.....KVesfstvgesllymlekclga........afspdvreawielysavvkamqrgwevlpegd.**

**.........|..||..|................|..||..||...|.......|..|..................|..||..||..|..||...............|..|...||.........|.........|..|..|||.||......................||..||..|...|............**

**gi|56368400|CAH23229.1 179aa Cygb (17-174)**

**eisdaekkviqetwsrvya.........ncedvgvsilirffvnfpsakqy..fsqf....khmddtlemerslqlrkhaqrvmgaintvvenl...ddpekvssvlalvgkahalkhk.....v.....epvyfkkltgvmleviaeaygn........dftpeahgawtkmrtliythvtaaykeagwvsy**

**.........|..||..|................|..||..||...|.......|..|..................|..||..||..|..||...............|..|...||.........|.........|..|..|||.||......................||..||..|...|............**

**gi|122315|P02001|HBAD_CHICK 141aa Alpha D**

**mltaedkkliqqawekaas.........hqeefgaealtrmfttypqtkty..fphf..........dlspgsdqvrghgkkvlgalgnavknv......dnlsqamaelsnlhaynlr.....v.....dpvnfkllsqciqvvlavhmgk........dytpevhaafdkflsavsavlaekyr.......**

**.........|..||..|................|..||..||...|.......|..|..................|..||..||..|..||...............|..|...||.........|.........|..|..|||.||......................||..||..|...|............**

**gi|49169791|NP_990820 147aa Beta**

**HWTAEEKQLITGLWGKVNV...........AECGAEALARLLIVYPWTQRF..FASF....GNLSSPTAILGNPMVRAHGKKVLTSFGDAVKNL......DNIKNTFSQLSELHCDKLH.....V.....DPENFRLLGDILIIVLAAHFSK........DFTPECQAAWQKLVRVVAHALARKYH.......**

**.........|..||..|................|..||..||...|.......|..|..................|..||..||..|..||...............|..|...||.........|.........|..|..|||.||......................||..||..|...|............**

**gi|50728806|XP_416292 153aa Mb**

**GLSDQEWQQVLTIWGKVEA.........DIAGHGHEVLMRLFHDHPETLDR..FDKF....KGLKTPDQMKGSEDLKKHGATVLTQLGKILKQK......GNHESELKPLAQTHATKHK.....I.....PVKYLEFISEVIIKVIAEKHAA........DFGADSQAAMKKALELFRNDMASKYKEFGFQG.**

**.........|..||..|................|..||..||...|.......|..|..................|..||..||..|..||...............|..|...||.........|.........|..|..|||.||......................||..||..|...|............**

**gi|56961659|NP_001008786.1 151aa eye-globin**

**sfseaevqsargawekmyv.........daedngtavlvrmftehpdtksy..fthf....kgmdsaeemkqsdqvrghgkrvftaindmvqhl...Dnteaflgilnplgqkhatqlk.....i.....dpknfriicdiilqlmeekfgg............dckasfekvtneicthltniykeagw...**

**.........|..||..|................|..||..||...|.......|..|..................|..||..||..|..||...............|..|...||.........|.........|..|..|||.||......................||..||..|...|............**

**Homo sapiens**

**gi|10864065|NP_067080 151aa Ngb**

**.merpepelirqswravsr.........splehgtvlfarlfalepdllpl..fqy...ncrqfsspedclsspefldhirkvmlvidaavtnv...edlssleeylaslgrkhravg......v.....klssfstvgesllymlekclgp........aftpatraawsqlygavvqamsrgwdge.....**

**.........|..||..|................|..||..||...|.......|..|..................|..||..||..|..||...............|..|...||.........|.........|..|..|||.||......................||..||..|...|............**

**gi|19549331|NP_599030 190 aa Cgb**

**elseaerkavqamwarlya.........ncedvgvailvrffvnfpsakqy..fsqf....khmedplemerspqlrkhacrvmgalntvvenl...hdpdkvssvlalvgkahalkhk.....v.....epvyfkilsgvilevvaeefas........dfppetqrawaklrgliyshvtaaykevgwvqq**

**gi|57013850|sp|P69905|HBA_HUMAN 142aa Alpha**

**vlspadktnvkaawgkvga.....hageygaealermflsfpttktyfph...fd.l............shgsaqvkghgkkvadaltnavahv....ddmpnalsalsdlhahklrvdp....v........nfkllshcllvtlaahlpa........eftpavhasldkflasvstvltskyr.......**

**.........|..||..|................|..||..||...|.......|..|..................|..||..||..|..||...............|..|...||.........|.........|..|..|||.||......................||..||..|...|............**

**gi|122335|sp|P02008|HBAZ_HUMAN 142aa Zeta**

**SLTKTERTIIVSMWAKIST.........QADTIGTETLERLFLSHPQTKTY..FPHF..........DLHPGSAQLRAHGSKVVAAVGDAVKSI......DDIGGALSKLSELHAYILR.....V.....DPVNFKLLSHCLLVTLAARFPA........DFTAEAHAAWDKFLSVVSSVLTEKYR.......**

**.........|..||..|................|..||..||...|.......|..|..................|..||..||..|..||...............|..|...||.........|.........|..|..|||.||......................||..||..|...|............**

**gi|4885395|NP_005322 142aa theta 1**

**alsaedralvralwkklgs.........nvgvyttealertflafpatkty..fshl..........dlspgssqvrahgqkvadalslaverl......ddlphalsalshlhacqlr.....v.....dpasfqllghcllvtlarhypg........dfspalqasldkflshvisalvseyr.......**

**.........|..||..|................|..||..||...|.......|..|..................|..||..||..|..||...............|..|...||.........|.........|..|..|||.||......................||..||..|...|............**

**gi|51510893|NP_001003938 141aa hemoglobin mu**

**mlsaqeraqiaqvwdliag.........heaqfgaelllrlftvypstkvy..fphl..........sacqdatqllshgqrmlaavgaavqhv......dnlraalspladlhalvlr.....v.....dpanfplliqcfhvvlashlqd........eftvqmqaawdkfltgvavvltekyr.......**

**.........|..||..|................|..||..||...|.......|..|..................|..||..||..|..||...............|..|...||.........|.........|..|..|||.||......................||..||..|...|............**

**gi|56749856|sp|P68871|HBB_HUMAN 147aa beta**

**hltpeeksavtalwgkvnv...........devggealgrllvvypwtqrf..fesf....gdlstpdavmgnpkvkahgkkvlgafsdglahl......dnlkgtfatlselhcdklh.....v.....dpenfrllgnvlvcvlahhfgk........eftppvqaayqkvvagvanalahkyh.......**

**.........|..||..|................|..||..||...|.......|..|..................|..||..||..|..||...............|..|...||.........|.........|..|..|||.||......................||..||..|...|............**

**gi|28302131|NP_000550.2 147aa gamma A**

**hfteedkatitslwgkvnv...........edaggetlgrllvvypwtqrf..fdsf....gnlssasaimgnpkvkahgkkvltslgdatkhl......ddlkgtfaqlselhcdklh.....v.....dpenfkllgnvlvtvlaihfgk........eftpevqaswqkmvtavasalssryh.......**

**gi|4504351|NP_000510 147aa delta**

**hltpeektavnalwgkv...........nvdavggealgrllvvypwtqrf..fesf....gdlsspdavmgnpkvkahgkkvlgafsdglahl......dnlkgtfsqlselhcdklh.....v.....dpenfrllgnvlvcvlarnfgk........eftpqmqaayqkvvagvanalahkyh.......**

**gi|122726|sp|P02100|HBE_HUMAN 147aa epsilon**

**HFTAEEKAAVTSLWSKMNV...........EEAGGEALGRLLVVYPWTQRF..FDSF....GNLSSPSAILGNPKVKAHGKKVLTSFGDAIKNM......DNLKPAFAKLSELHCDKLH.....V.....DPENFKLLGNVMVIILATHFGK........EFTPEVQAAWQKLVSAVAIALAHKYH.......**

**.........|..||..|................|..||..||...|.......|..|..................|..||..||..|..||...............|..|...||.........|.........|..|..|||.||......................||..||..|...|............**

**gi|44955888|NP_976312 154aa Mb**

**glsdgewqlvlnvwgkvea.........dipghgqevlirlfkghpetlek..fdkf....khlksedemkasedlkkhgatvltalggilkkk......ghheaeikplaqshatkhk.....i.....pvkylefiseciiqvlqskhpg........dfgadaqgamnkalelfrkdmasnykelgfqg.**

**Mus musculus**

**gi|11967939|NP_071859 151aa Ngb**

**.MERPESELIRQSWRVVSR.........SPLEHGTVLFARLFALEPSLLPL..FQ.Y..NGRQFSSPEDCLSSPEFLDHIRKVMLVIDAAVTNV...EDLSSLEEYLTSLGRKHRAVG......V.....RLSSFSVVGESLLYMLEKCLGP........DFTPATRTAWSRLYGAVVQAMSRGWDGE.....**

**.........|..||..|................|..||..||...|.......|..|..................|..||..||..|..||...............|..|...||.........|.........|..|..|||.||......................||..||..|...|............**

**gi|19549320|NP_084482 190aa Cygb**

**ELSEAERKAVQATWARLYA.........NCEDVGVAILVRFFVNFPSAKQY..FSQF....RHMEDPLEMERSPQLRKHACRVMGALNTVVENL...HDPDKVSSVLALVGKAHALKHK.....V...,,EPMYFKILSGVILEVIAEEFAN........DFPVETQKAWAKLRGLIYSHVTAAYKEVGWVQQ**

**gi|122441|sp|P01942|HBA_MOUSE 142AA Alpha**

**VLSGEDKSNIKAAWGKIGG.........HGAEYGAEALERMFASFPTTKTY..FPHF..........DVSHGSAQVKGHGKKVADALANAAGHL......DDLPGALSALSDLHAHKLR.....V.....DPVNFKLLSHCLLVTLASHHPA........DFTPAVHASLDKFLASVSTVLTSKYR.......**

**.........|..||..|................|..||..||...|.......|..|..................|..||..||..|..||...............|..|...||.........|.........|..|..|||.||......................||..||..|...|............**

**gi|31982300|ref|NP_032246.2 147aa Beta**

**hltdaekaavsglwgkvna...........devggealgrllvvypwtqry..fdsf....gdlssasaimgnakvkahgkkvitafndglnhl......dslkgtfaslselhcdklh.....v.....dpenfrllgnmivivlghhlgk........dftpaaqaafqkvvagvaaalahkyh.......**

**.........|..||..|................|..||..||...|.......|..|..................|..||..||..|..||...............|..|...||.........|.........|..|..|||.||......................||..||..|...|............**

**gi|6680177|NP_032247.1 147aa HbY**

**NFTAEEKTLINGLWSKVNV...........EEVGGEALGRLLVVYPWTHRF..FDSF....GNLSSASAIMGNPRVKAHGKKVLTAFGESIKNL......DNLKSALAKLSELHCDKLH.....V.....DPENFKLLGNVLVIVLASHFGN........EFTAEMQAAWQKLVAGVATALSHKYH.......**

**.........|..||..|................|..||..||...|.......|..|..................|..||..||..|..||...............|..|...||.........|.........|..|..|||.||......................||..||..|...|............**

**gi|127676|P04247|MYG_MOUSE 154aa Mb**

**GLSDGEWQLVLNVWGKVEA.........DLAGHGQEVLIGLFKTHPETLDK..FDKF....KNLKSEEDMKGSEDLKKHGCTVLTALGTILKKK......GQHAAEIQPLAQSHATKHK.....I.....PVKYLEFISEIIIEVLKKRHSG........DFGADAQGAMSKALELFRNDIAAKYKELGFQG.**

**Rattus norvegicus**

**gi|19923711|NP_203523.2|Q8VH38 (Q99JA8) 151aa Ngb**

**.MERLESELIRQSWRAVSR.........SPLEHGTVLFSRLFALEPSLLPL..FQ.Y..NGRQFSSPEDCLSSPEFLDHIRKVMLVIDAAVTNV...EDLSSLEEYLATLGRKHRAVG......V.....RLSSFSTVGESLLYMLEKCLGP........DFTPATRTAWSQLYGAVVQAMSRGWDGE.....**

**.........|..||..|................|..||..||...|.......|..|..................|..||..||..|..||...............|..|...||.........|.........|..|..|||.||......................||..||..|...|............**

**gi|18543351|NP_570100.1|sp|Q921A4|CYGB_RAT 190aa (18-173) Cygb**

**ELSEAERKAVQATWARLYA.........NCEDVGVAILVRFFVNFPSAKQY..FSQF....KHMEDPLEMERSPQLRKHACRVMGALNTVVENL...HDPDKVSSVLALVGKAHALKHK.....V.....EPMYFKILSGVILDVIAEEFAN........DFPVETQKAWTKLRGLIYSHVTAAYKEVGWVQQ**

**.........|..||..|................|..||..||...|.......|..|..................|..||..||..|..||...............|..|...||.........|.........|..|..|||.||......................||..||..|...|............**

**gi|122477|sp|P01946|HBA_RAT 142aa alpha**

**vlsaadktnikncwgkigg.........hggeygeealqrmfaafpttkty..fshi..........dvspgsaqvkahgkkvadalakaadhv....edlpgalstlsdlhahklr.......v.....dpvnfkflshcllvtlachhpg........dftpamhasldkflasvstvltskyr.......**

**.........|..||..|................|..||..||...|.......|..|..................|..||..||..|..||...............|..|...||.........|.........|..|..|||.||......................||..||..|...|............**

**gi|27668422|XP_213262 140aa alpha**

**vlseedknnikkawvkign.........haaeigaetigrlfivfpsskty..fphf..........ntsegsdqvkahgkkvadaltnaashl....ddlpgalstlsdlhahklr.......v.......dpvnflshcllvtlashhpg........dftpamhasldkffasvstvltskyr.......**

**gi|122514|sp|P02091|HBB1_RAT 147aa Beta**

**hltdaekaavnglwgkvnp...........ddvggealgrllvvypwtqry..fdsf....gdlssasaimgnpkvkahgkkvinafndglkhl......dnlkgtfahlselhcdklh.....v.....dpenfrllgnmivivlghhlgk........eftpcaqaafqkvvagvasalahkyh.......**

**.........|..||..|................|..||..||...|.......|..|..................|..||..||..|..||...............|..|...||.........|.........|..|..|||.||......................||..||..|...|............**

**gi|27678778|XP_215029  147aa   epsilon 1**

**nftaeekslinglwskvnv...........eevggealgrllvvypwtqrf..fdsf....gnlssasaimgnprvkahgkkvltafgetiknl......dnlksalaklselhcdklh.....v.....dpenfkllgnvlvivlashfgn........eftaevqaawqklvagvatalshkyh.......**

**gi|11024650|NP_067599 154aa Mb**

**glsdgewqmvlniwgkveg.........dlaghgqevlislfkahpetlek..fdkf....knlkseeemkssedlkkhgctvltalgtilkkk......gqhaaeiqplaqshatkhk.....i.....pvkylefiseviiqvlkkrysg........dfgadaqgamskalelfrndiaakykelgfqg.**

**.........|..||..|................|..||..||...|.......|..|..................|..||..||..|..||...............|..|...||.........|.........|..|..|||.||......................||..||..|...|............**

**Tetraodon nigroviridis**

### gi|32171394|Q90W04 159aa Ngb

**klsskdkelirgswdslgk.........nkvphgvilfsrlfeldpellnl..fh.y...ttncgstqdclsspeflehvtkvmlvidaavshl...ddlhsledfllnlgrkhqavg......v.....kpqsfamvgesllymlqcslgq.......aytaslrqawlnmysvvvasmsrgwakngedkad**

**.........|..||..|................|..||..||...|.......|..|..................|..||..||..|..||...............|..|...||.........|.........|..|..|||.||......................||..||..|...|............**

**gi|47219636|CAG02681 174aa Cygb**

### pltekekvriqdswakvfq.........scddagvailvrffvnfpsskqf..fkdf....khmeepeemqqsvqlrkhahrvmtalntlvesl...nnadrvasvlksvgrahalkhn.....v.....dpkyfkilsgvilevlgeafte........iitaevasawtkllanmccgiaavykeagwtel

**.........|..||..|................|..||..||...|.......|..|..................|..||..||..|..||...............|..|...||.........|.........|..|..|||.||......................||..||..|...|............**

**gi|47226107|CAG04481 143aa Alpha**

### sltktdkaavkalwnklsk.........svdvigaeafgrmllvypqtkiy..fskw.........gdisfgssqvknhgkivmggiatavani......ddltsglqklsevhafdlk.....v.....dpanfkilgqcvvvvtamlfpk........dftpevhvsfdkflaavalalsekyr.......

**gi|42661496|CAF31356 146aa Mb**

**...mgdfdmvlkfwgpvea.........dysahggmvltrlftenpetqql..fpkf.....vgiaqselagnaavsahgatvlkklgellkak......gnhaailqplanshatkhk.....i.....piknfkliaevigkvmaekagl..........daagqqalrnimatiiadidatykelgfs..**

**.........|..||..|................|..||..||...|.......|..|..................|..||..||..|..||...............|..|...||.........|.........|..|..|||.||......................||..||..|...|...........**

**gi|56368394|CAG25722.1 205aa (32-187) globin X**

**ypredqiqmikdswkvird.........diakvgiimfvrlfethpeckdvf.fl.f....rdvedlerlrssrelrahglrvmsfieksvarl...dqqdrlealavelgkshyhyn......a.....PPKYysyvgaeficavqpilke........rftseleeawktlfqyvtglmrkghqeegsrqr**

**.........|..||..|................|..||..||...|.......|..|..................|..||..||..|..||...............|..|...||.........|.........|..|..|||.||......................||..||..|...|............**

**Xenopus (Silurana) tropicalis**

**gi|62146705|emb|CAG25550 164aa  (6-154)  Ngb**

**QLSGPQKELIRESWQTVSQ.........DQLHHGTVLFSRLFELEPELVFL..FQ.Y..NSSHFSKVQDCLSSAEFTEHIRKVMTVIDAAVSSL...DCLSSLDEYLTSLGRKHRAVG......V.....KLESFNTVGESLLFALESCLGD........AFTSDTREAWSLLYANVVQSMSRGWHRDSQEQR**

**.........|..||..|................|..||..||...|.......|..|..................|..||..||..|..||...............|..|...||.........|.........|..|..|||.||......................||..||..|...|............**

**>gi|55742013|NP_001006870 179aa (18-177) Cygb**

**EITESERGVIKETWARVYA.........NCEDVGVSILIRFFVNFPSAKQH..FSQF....KHMEDPLEMEGSVQLRKHARRVMGAVNSVVENL...GDPEKITTVLSIVGKSHALKHK.....V....DPVYFKILTGVMLEVIAEEYAKD.........FTPDVQLAWNKLRSHLYSHVLSAYKEAGWTQY**

**.........|..||..|................|..||..||...|.......|..|..................|..||..||..|..||...............|..|...||.........|.........|..|..|||.||......................||..||..|...|............**

**gi|122509|P07428|HBA_XENTR 142aa alpha**

**HLTADDKKHIKAIWPSVAA.........HGDKYGGEALHRMFMCAPKTKTY..FPDF..........DFSEHSKHILAHGKKVSDALNEACNHL......DNIAGCLSKLSDLHAYDLR.....V.....DPGNFPLLAHQILVVVAIHFPK........QFDPATHKALDKFLVSVSNVLTSKYR.......**

**.........|..||..|................|..||..||...|.......|..|..................|..||..||..|..||...............|..|...||.........|.........|..|..|||.||......................||..||..|...|............**

**gi|122524|sp|P07429|HBB1_XENTR 147aa beta-1**

**NLTAKERQLITGTWSKICA...........KTLGKQALGSMLYTYPWTQRY..FSSF....GNLSSIEAIFHNAAVATHGEKVLTSIGEAIKHM......DDIKGYYAQLSKYHSETLH.....V.....DPYNFKRFCSCTIISMAQTLQE........DFTPELQAAFEKLFAAIADALGKGYH.......**

**.........|..||..|................|..||..||...|.......|..|..................|..||..||..|..||...............|..|...||.........|.........|..|..|||.||......................||..||..|...|............**

**gi|58332516|NP_001011196 200aa (29-183) globin X**

**NLSEQQQQLLVESWRLIQH.........DIAKVGVILFVRLFETHPECKDVF.FL.F....RDVDDLQALRANKDLRAHGLRVLSFVEKSVARI...ADCARLEELALELGRSHYRYN......A.....PPRYYQYVGTEFISAVCPMLHD........KWTAEVEEAWKGLFAYICTVMERGYQEEERRHS**

**.........|..||..|................|..||..||...|.......|..|..................|..||..||..|..||...............|..|...||.........|.........|..|..|||.||......................||..||..|...|............**

**UROCHORDATE Hbs**

**Ciona intestinalis**

**gi|34364568|emb|CAD68145 156aa**

**PFTDEELKLLRNSWDEVKK........lGMKEVGLHIFTGLLNAAPSLRTL..FYTIdlpdeeeltidvmrenkkvvahatrianaiskfikfl...dqpdeleklltslgesharrq......v.....dpesfeyvapvilsvigghlkl........psnsptlqawvkaygvlrngivsamea......**

**.........|..||..|................|..||..||...|.......|..|..................|..||..||..|..||...............|..|...||.........|.........|..|..|||.||......................||..||..|...|............**

**gi|34364574|emb|CAD89600 293aa (134-293)**

**GLKRSDIINIQDSWNTLKG........FGYETVGMLVLHRLFNDAPQTRYL..FSQLslssnesftleqmrnnsrvvyhanrvaravgrlvdli...elptnftdhlvwlgqrhayhg......v.....apvnfdymgpvlletikvnlel........psdsptlsawakaygvikngikdaiiatyaeg.**

**.........|..||..|................|..||..||...|.......|..|..................|..||..||..|..||...............|..|...||.........|.........|..|..|||.||......................||..||..|...|............**

**gi|34364570|emb|CAD68146 159aa**

**GLTTEEIGLLRSSWNEMKT.......IGMKELGLLIFHRLFSDVPRIRKM...FYNLelpddetltmeamrsnqkmsrhatriatsistylkla...dqpeelktflnglgelhaghn......v.....epedfeylapvmlaviggqlnl........nsnssilqawvkaygvlrngivrgmyayqg...**

**.........|..||..|................|..||..||...|.......|..|..................|..||..||..|..||...............|..|...||.........|.........|..|..|||.||......................||..||..|...|............**

**gi|34364572|emb|CAD68147 153aa**

**SLTSEQVVLLRSSWQTIGK........LGMSNVGLAVLHRLFNDVPETLPF..FHSV.lsptqqteievlksnakvvrhasrvglsidkiinll...dngeelvkyllflgqvhvkrs......i.....prkyfsamgpvllsvisavlek........dldapvmqawataygvieqgiidgm........**

**.........|..||..|................|..||..||...|.......|..|..................|..||..||..|..||...............|..|...||.........|.........|..|..|||.||......................||..||..|...|............**

**ECHINODERM Hbs**

**Strongylocentrotus purpuratus (Echinodermata; Echinoidea)**

**gi|72169631|XP_795670.1 175 aa (18-173)**

**gltkqqkalikkswtyvle.........dklrigviifiklfkafpasqql..fekl....kdytdfeelarnkkmkahatrvmaaltslveni...dqpdildellrntsvthyrmr......m.....pphyfedlggviiealvenlgdk........ftpktkeawliyygymcrimleemeelepsnd**

**.........|..||..|................|..||..||...|.......|..|..................|..||..||..|..||...............|..|...||.........|.........|..|..|||.||......................||..||..|...|............**

**INSECT Hbs**

**Anopheles gambiae**

**gi|55246163|EAA03862.3|ENSANGP00000019788 150aa (25-150)**

**gltksqkvaliaawsivkk.........dlvthgrnifvia..........................gelgenrslhahalnvmnfigtlidyg..lndpallkcslgklvrnhrkrn......v.....tkedvaavggvimryslkaLE........qhktktleeafgaflgtvaaafe...........**

**.........|..||..|................|..||..||...|.......|..|..................|..||..||..|..||...............|..|...||.........|.........|..|..|||.||......................||..||..|...|............**

**gi|31201271|XP_309583.1|ENSANGP00000022287 192aa (49-192) GLTKSQKVALIAAWSIVKK.........DLVTHGRNIFVMFFEEYPQYLDY..FD.F.....GggsaGELGENRSLHAHALNVMNFIGTLIDYG..LNdpalLKCSLAKLVRNHRKRN......V.....TKEDVAAVGGVIMRYSLKALE........QHKTKTLEEAFGAFLGTVAAAFE...........**

**.........|..||..|................|..||..||...|.......|..|..................|..||..||..|..||...............|..|...||.........|.........|..|..|||.||......................||..||..|...|............**

**gi|31198253|XP_308074|ENSANGP00000019788 215aa (110-215)**

**gltksqkvaliaawsivkk.........dlvthgrnifvmffeeypqyldy..fd.f.....gggsagelgenrslhahalnvmnfigtlidyg..lndpallkcslgklvrnhrkrn......v.....tkedva.........................................................**

**.........|..||..|................|..||..||...|.......|..|..................|..||..||..|..||...............|..|...||.........|.........|..|..|||.||......................||..||..|...|............**

**gi|57914327|XP_555006.1 ENSANGP00000026474 182aa (5-159)**

**GLTASEKITLFSAWGLIRK.........DLDVHGRNVLLLLFHKHPRYIAY..FD.F....TDDPNAQSLVDNKSLYDQAIHVFKAVGALIEYG..FKDPVLFDATLRKITRRHKDRP......V.....YTEDILTIGEVLLNYLEQALG........RQMSDSLPDAFWKLFQTIAGRFPATPKTPIADE.**

**.........|..||..|................|..||..||...|.......|..|..................|..||..||..|..||...............|..|...||.........|.........|..|..|||.||......................||..||..|...|............**

**Drosophila melanogaster**

**gi|5731771|CAB52584|CG9734 153aa**

**.mnsdevqlikktweipva.........tptdsgaailtqffnrfpsnlek..fp.f.....rdvpleelsgnarfrahagriirvfdesiqvlgqdgdlekldeiwtkiavshiprt......v.....skesynqlkgvildvltaacsl..........desqaatwaklvdhvygiifkaidddgnak.**

**.........|..||..|................|..||..||...|.......|..|..................|..||..||..|..||...............|..|...||.........|.........|..|..|||.||......................||..||..|...|............**

**gi|28571530|NP_649597|CG14675 195aa (34-183)**

**GFTLSERLALRQAWNLVRP.........FERRYGQDVFYSFLNDY.YWGIKK.F...........RNGaELNVKALHSHALRFINFFGLLIEEK....DPVVFQLMINDNNHTHNRCHVGSVN.I......GHLAQALVDYVLKVFHKVSSP........SLEQGLSKLVEKFQNYQDQQSNTSGYNRLSK..**

**.........|..||..|................|..||..||...|.......|..|..................|..||..||..|..||...............|..|...||.........|.........|..|..|||.||......................||..||..|...|............**

**gi|45550703|NP_649669|CG15180 209aa (32-189)**

**fpvypkplpdrdlsykade.....NEFTMVEKASLRNAWRLIE..Pfqrr...fgk.....enfyrkdgkinlsklhGHAMAMMKLMSKLVQTL...DCNLAFRLaldenlpthlkng......i.....dpdymrmlatalksyilassvi......enhnscslsnglarlveivgeyavvdearkramst**

**.........|..||..|................|..||..||...|.......|..|..................|..||..||..|..||...............|..|...||.........|.........|..|..|||.||......................||..||..|...|............**

**Drosophila pseudoobscura**

**gi|54639008|EAL28410.1 152aa**

**.mnsdevqvikktweipva.........tptdsgaailqefftrfpsnlek..fs.f.....rdvpledlsgnsrfrahagriirvfddsirvlgeegdvekleeiwtkiavshiprt......i.....skesynqlkgvilevltaacsl..........desqaatwaklvdhiygiifkaidddgna..**

**.........|..||..|................|..||..||...|.......|..|..................|..||..||..|..||...............|..|...||.........|.........|..|..|||.||......................||..||..|...|............**

**gi|54638691|EAL28093.1 641aa (45-189) FUGUE ALIGNMENT**

**gftlcekvalrqawnlirp.........rerrfgqdvfytflnewywsisk..fkk...........gediniallhahaltfirfvgalines.dpimfqmlgqaltdyilkvldkt......v.....sqedadpsarnpmlydplqdgv........vktsktrqyvaalimclgavaagtalawtspvl**

**.........|..||..|................|..||..||...|.......|..|..................|..||..||..|..||...............|..|...||.........|.........|..|..|||.||......................||..||..|...|............**

**gi|54638692|EAL28094.1  130aa FUGUE ALIGNMENT**

**....................................iicsflnewywsisk..FKK...........gediniallhahaltfirfvgalines.dpimfqvm...inennqthsrcr......v.....gadyiamlgqaltdyilkvldkv........rspsleqglqrivekfksyqdiqmdrsktsyr**

**.........|..||..|................|..||..||...|.......|..|..................|..||..||..|..||...............|..|...||.........|.........|..|..|||.||......................||..||..|...|............**

**PLANT Hbs**

**Arabidopsis thaliana**

**GLB1 gi|15226675|NP_179204 160aa NsHb**

**VFTEEQEALVVKSWSVMKK.........NSAELGLKLFIKIFEIAPTTKKM..FSFL......rdspipaeqnpklkphamsvfvmccesavqlrktgkvtvrettlkrlgashskygv.....v......dehfevakyalletikeaVP........emwspemkvawgqaydhlvaaikaemnlsn....**

**.........|..||..|................|..||..||...|.......|..|..................|..||..||..|..||...............|..|...||.........|.........|..|..|||.||......................||..||..|...|............**

**GLB2 gi|15228313|NP_187663 158aa NsHb**

**GFTEKQEALVKESWEILK.........QDIPKYSLHFFSQILEIAPAAKGL..FSFL......rdsdevphnnpklkahavkvfkmtcetaiqlreegkvvvadttlqylgsihlksgv.....i......dphfevvkeallrtlkeglg........ekyneevegawsqaydhlalaiktemkqees...**

**.........|..||..|................|..||..||...|.......|..|..................|..||..||..|..||...............|..|...||.........|.........|..|..|||.||......................||..||..|...|............**

**GLB3 gi|18418064|NP_567901 175aa 2/2Hb**

**DQAEAFAIDESNLFDKLGL........QTFINLSTNFYTRVYDDE.EEW....FQSI.............FSNSnkedaiqnqyeffvqrmggp....plysqrkghpaligrhrpfp......v.....thqaaerwlehmqnalddsvd.........idqdskikmmkffrhtafflvagnelknqnekp**

**.........|..||..|................|..||..||...|.......|..|..................|..||..||..|..||...............|..|...||.........|.........|..|..|||.||......................||..||..|...|............**

**gi|7486404|T04457 671aa (15-164) 2/2Hb**

**dqaeafaidesnlfdklgl........Qtfinlstnfytrvydde.eew....fqsi.............fsnsnkedaiqnqyeffvqrmggp....plysqrkghpaligrhrpfp......v.....thqaaerwlehmqnalddsvd.........idqdskikmmkffrspavtlfpplmdsdssksr**

**.........|..||..|................|..||..||...|.......|..|..................|..||..||..|..||...............|..|...||.........|.........|..|..|||.||......................||..||..|...|............**

**Oryza sativa ssp. indica**

**gi|17366135|sp|O04986|HBL1_ORYSA 166aa NsHb**

**sfseeqealvlkswailkk.........dsanialrfflkifevapsasqm..fsfl......rnsdvpleknpklkthamsvfvmtceaaaqlrkagkvtvrdttlkrlgathlkyg......v.....gdahfevvkfalldtikeevp.......admwspamksawseaydhlvaaikqemkpae....**

**.........|..||..|................|..||..||...|.......|..|..................|..||..||..|..||...............|..|...||.........|.........|..|..|||.||......................||..||..|...|............**

**gi|17432965|sp|O04985|HBL2_ORYSA 169aa**

**sfseeqealvlkswaimkk.........dsaniglrfflkifevapsasqm..fsfl......rnsdvpleknpklkthamsvfvmtceaaaqlrkagkvtvrdttlkrlgathfkyg......v.....gdahfevtrfalletikeavp.......vdmwspamksawseaynqlvaaikqemkpae....**

**.........|..||..|................|..||..||...|.......|..|..................|..||..||..|..||...............|..|...||.........|.........|..|..|||.||......................||..||..|...|............**

**gi|22001645|sp|Q94FT8|HBL3_ORYSA 169aa**

**rfteeqealvlkswaimkn.........dsahighrfflkifevapsarql..fsfl......rnsdvpleknpklkihamavfvmtceaaaqlrktgrvtvrdttikrlgsthfkng......v.....sdahfevakfalletikeavp.......asmwspamkgawgeaydhlvaaikqgmkpaaa...**

**.........|..||..|................|..||..||...|.......|..|..................|..||..||..|..||...............|..|...||.........|.........|..|..|||.||......................||..||..|...|............**

**gi|22001644|sp|Q94FT7|HBL4_ORYSA 167aa**

**rfteeqealvlkswaimkd.........dsanighrfflkifevapsarhl..fsfl......rnsdvpleknpnlkkhamavfvmtceaaaqlrktgrvtvrdttikrlgsthfkng......v.....sdthfevarfalletikdgip.......asmwspemknawgeayehlvaaikegmkpvall..**

**.........|..||..|................|..||..||...|.......|..|..................|..||..||..|..||...............|..|...||.........|.........|..|..|||.||......................||..||..|...|............**

**gi|50932383|ref|XP_475719.1 Scaffold874_6 (1-143) 145aa**

**GFSETQEELVLRSWQSMKK.........DSESIALKFFLRIFEIAPAAKQM..FSFL..........RDSGDDVPLESHPKACESATQLRKTGD.....VKVREATLRRLGATHVKAG......V.....ADAHFEVVKTALLDTIKDAVP........EMWSPEMKGAWEEAYDQLAAAIKEEMKKAA....**

**.........|..||..|................|..||..||...|.......|..|..................|..||..||..|..||...............|..|...||.........|.........|..|..|||.||......................||..||..|...|............**

**gi|50725383|BAD32857.1 172aa 2/2Hb**

**aagdafaiddgnvfealgg......ttqpfvdlstnfytrvyede.eew....frqi.............fagskkedairnqyeflvqrmggp....qlfsqrrghpaliarhrpfp......v.....thqaaerwlhhmqqavdttds.........idaatktkmmyffrhtayflvagnemtrqghgt**

**.........|..||..|................|..||..||...|.......|..|..................|..||..||..|..||...............|..|...||.........|.........|..|..|||.||......................||..||..|...|............**

**ALGAL SDgbs and 2/2Hbs**

# Chlamydomonas reinhardtii

# 160981|C_240146 136aa 2/2Hb ..MAADTAPADSLYSRMGG.......EAAVEKAVDVFYERIVAD.PQLAPF..FANV.................DMKKQRRKQVAFMTYVFGGS........GAYEGRDLGASHRRL.......IREQGMNHHHFDLVAAHLDSTLQELGVA..........QELKAEAMAIVASARPLIFGTGEAGAAN...

**.........|..||..|................|..||..||...|.......|..|..................|..||..||..|..||...............|..|...||.........|.........|..|..|||.||......................||..||..|...|............**

# 160982|C_240147 147aa AAGVVDAAAPPSIFDRLGG.......EAAVEKAVDVFYERIVAD.PQLAPF..FEGV.................DMRTQRRKQQAFMTYAFGGA........TGYTGRDLAAAHRRL.......IRDKGLKEEHFDMVAGHLAGTLQLLGIG..........ADLVDEVIALVATTKPVIFERV.........

**.........|..||..|................|..||..||...|.......|..|..................|..||..||..|..||...............|..|...||.........|.........|..|..|||.||......................||..||..|...|............**

**157690|C_1780015 231aa (65-203)**

**GQSKSAATTRSTLHAKLGG.......AAAVAATVDVFYKKLMND.PDLEPF..FRGV.................DMVTLIAKQNRFLAYAFGAT........THYHGKDIVMGHAHL.......IINRGLNLTHFDKVAGHFVDSLKEMGVG...........QELIDEAAGVLIGVRPLFDPERYKGKVDVE**

**.........|..||..|................|..||..||...|.......|..|..................|..||..||..|..||...............|..|...||.........|.........|..|..|||.||......................||..||..|...|............**

**168933|C_60168 651aa (42-180)**

**KVNQRALPTNGPLLDRMGG.......ADYVKRVAERFYHKLYADE.QLAP...FLG................EHDVVSLRAKQNAFLIWAFGPP.......NRPYPGRHLRTAHLRL.......IKQKGFTVEHFEMGLKHFEEALKEMDAP........PTVVFTPSSARDAEEELRWVKEERMRAQ.....**

**168934|C_60169 476aa (1-132)**

# .........MITLLKRIGG.......EKGMKDVVEMFYRKIYANE.RLRH...FLKD................KDVSLLRSKQAAFMTWLFGPS.......TATYTGRSVRCAHLRM.......IKQRGFSEDDFLLGMSCFEEAMRDYGAP..........QRLTNEIMAKILPFKDIIFSPSAADAGEEA.

**.........|..||..|................|..||..||...|.......|..|..................|..||..||..|..||...............|..|...||.........|.........|..|..|||.||......................||..||..|...|............**

**168800|C_60035 432aa (34-174)**

**ARNGGPQSSTGPLLQRVGG.......SDVVKHVVELFYRHLYAD.PSLIK...FLH................DQEMTHLRAKQSAFISWLFGPP.......NVPYTGKSVRIAHLRI.......IKQRGFSPEDFDLGMKYFAESMRELGAP..........EILINEVLRRITPYKDAIFTPSAGDAAEEA.**

**.........|..||..|................|..||..||...|.......|..|..................|..||..||..|..||...............|..|...||.........|.........|..|..|||.||......................||..||..|...|............**

**153190|C_100138 837aa D1 (28-162)**

**KKATNDLNHAGGIVERLGG.......ADRVHDVIQAFYRKLFQN.AMVRHY..FENL.................SSDRMRSKQMKFMRYIMGGP.........GTYTGNLRCIHAQM.......VKEEGLDKAKFQVVIGMLLDTFKELNVP..........QDVVSEILGHVESAKSAIFTPQPDEL.....**

**.........|..||..|................|..||..||...|.......|..|..................|..||..||..|..||...............|..|...||.........|.........|..|..|||.||......................||..||..|...|............**

**153190|C_100138 837aa D2 (168-306) VAAAADLGRSCPLVAKLGG.......PEPVQGIITAFYRRLFSSE.ELKYF..FVGL.................STERLRTMQFRFMRYLLAGP......GWYATTGGNMRCVHARM.......IAEGGLDLPKFQAVTGMLRAACDEHLVS..........PPLIREIMLNVKAAEQAIFTPGPDEL.....**

**.........|..||..|................|..||..||...|.......|..|..................|..||..||..|..||...............|..|...||.........|.........|..|..|||.||......................||..||..|...|............**

**168904|C_60139 347aa (74-235) AAEQAHKRIKNSEE**

**QVHGLAPPQEVAAEGSDGG..(14)..FSMKFLVDKFYGKVLKIK.EIKPF..FDGV.................DMHKLKYHQEALMLLVFGGQ..ELLDDELPGLSADLRLIHLHL.......L.FEGLNLSHWQIFADTFCETLDELPQI.......PADVKAHAKAYMRATKQHFRPIEPEEWPPKVV..**

**.........|..||..|................|..||..||...|.......|..|..................|..||..||..|..||...............|..|...||.........|.........|..|..|||.||......................||..||..|...|............**

**Cyanidioschyzon merolae**

**CMR319C 185aa (46-185) SDgb**

**EPQQESIAIVQSTWSQAVQ.........QRERLGQVFYDRLFALYPELQPM..FRS..................DPALQRIRLVDMVDAGVKLL...NSRRDLEQALRDLGKRHVK........Y...GTQEEQYPIVGENLLHALESILGS.......KHFSEDMRKAWLDVYAYWSSVMLEGAREAQ...**
.**........|..||..|................|..||..||...|.......|..|..................|..||..||..|..||...............|..|...||.........|.........|..|..|||.||......................||..||..|...|............**

**Phaeodactylum tricornutum**

**PTMM05212_1 167aa 2/2Hb**

**DESLEAQRLVPSIFERIGG.......EKGFRGLSEMFYNFVFDDREATW....FLNI.............FSSSTRAEAIENQYLFFVQTFGGP...DLYKQKKGKYTRLVGRHANYN......I...GPQAADRWVYHMKRALDQNEHLSS...DAETREKLHNYFLYMAHYIVIAGEYMRPDQLSGGTKVD**

.**........|..||..|................|..||..||...|.......|..|..................|..||..||..|..||...............|..|...||.........|.........|..|..|||.||......................||..||..|...|............**

**PTMM07792_1 171aa 2/2Hb1**

**VLFSGESERKRTLLGKLGG.......KDILNEAVDVFYERLLQDDDMNQF...FRG..............TDMQILKWHQLNLMSVAFTKVPDN.........FDLASMILRQHRRFFE.....M...GMTEFHFDIFVGHFKAAFQTLNV...........EAQLVDEASTVIRXLRFCLXARCDXRQXXEX**

.**........|..||..|................|..||..||...|.......|..|..................|..||..||..|..||...............|..|...||.........|.........|..|..|||.||......................||..||..|...|............**

**PTMM03909_1 157aa SDgb**

**...........................GLDVDCTRIFYTELFRKYPSVQPM..FQH...............SNMEVQAQKLYEVIRVAVRFLDN.....VQELIPVLKDLGMRHAKHYGV....L......REHYDAVTEVFISVLNNYILTELDCGNAGIWAMEVADAWHWVLTFIGNTMADAGDQAMVKDV**

.**........|..||..|................|..||..||...|.......|..|..................|..||..||..|..||...............|..|...||.........|.........|..|..|||.||......................||..||..|...|............**

**Thalassiosira pseudonana**

**Scaff_137 127aa 2/2Hb**

**GLSPEDLSLVQKSWAKVVP.........IASVAADLFYTKLFELDPELRPL..FPS..................DLADQKKKLMAMISVAVDGL....TDLEALVPAVQDLGRRHAKY......Y...KVTAPMFDTVGAALLDTLEKGLGEG........WDEEHKEAWTLVYGVLSKTMIDAGEESTAA..**

**Scaff_18 160aa SDgb**

**QSLLATTSLTPTLLERIGG.......SSGFITLSTLFYNRVFNDKANPW....FLGI.............FATSTKDEAIDNQFRFLVQTFGGE...ELYRQKKGKYTRLVGRHANYQIQ....IAAAERWVVHMEGAIDEHEALVDDEEARE.........CLKLYFRYTAFYIVVASEFMRDDQ.......**

**.........|..||..|................|..||..||...|.......|..|..................|..||..||..|..||...............|..|...||.........|.........|..|..|||.||......................||..||..|...|............**

**FUNGAL FHbs**

**Aspergillus nidulans gi|40739893|gb|EAA59083|AN3522.2 410aa**

**ALTPQQKQIVKATIPALEQ.............HGVTITTLFYKEMLERHPELNNI..FNSS...............HQATGVQPAALAHAVWAYASNI...DHPEALATAVSRIGHKHASLG......V.....RSEHYPIVGEHLLRAIKKVLGDAV........TPPVADAWTAAYQQLADIFISFEDDLYKQATQ**

**.........|..||..|....................|..||..||...|.......|..|..................|..||..||..|..||...............|..|...||.........|.........|..|..|||.||......................||..||..|...|............**

**Aspergillus nidulans gi|40742231|gb|EAA61421|AN7169.2 426aa**

**SLSPEQIQLIKATVPVLQE.............HGTTITKVFYDNMLTAHPELKTV..FNVS...............NKVHGHQPQALAGALFAYASYI...DNLGVLSPAVERICHKHASLA......I.....QPDGYQIVGKFLLEAMGQVLGDAL........TPPLLEAWAAAYWQLANIMIGKEAELYKSADG**

**.........|..||..|....................|..||..||...|.......|..|..................|..||..||..|..||...............|..|...||.........|.........|..|..|||.||......................||..||..|...|............**

**Aspergillus niger gi|60417044|CAF32309 412aa**

**pltpeqikiikatvpvlqe.............ygtkittaxymnmstvhpelnav..fnta...............nqvkghqaralagalfayashi...ddlgalgpavelicnkhasly......i.....qadeykivgkylleamkevlgdac........tddildawgaaywaladiminreaalykqsqg**

**.........|..||..|....................|..||..||...|.......|..|..................|..||..||..|..||...............|..|...||.........|.........|..|..|||.||......................||..||..|...|............**

**Aspergillus niger gi|58864970|CAF25490 455aa (45-**

**pltpeqikiikatvpvlqe.............ygtkittaxymnmstvhpelnav..fnta...............nqvkghqaralagalfayashi...ddlgalgpavelicnkhasly......i.....qadeykivgkylleamkevlgdac........tddildawgaaywaladiminreaalykqsqg**

**.........|..||..|....................|..||..||...|.......|..|..................|..||..||..|..||...............|..|...||.........|.........|..|..|||.||......................||..||..|...|............**

**Candida albicans gi|46432319|EAK91807.1 398aa**

**qltpaqvkiildtvpilee.............agetltqkfyqrmignydevkpf..fnt...............Tdqkllrqpkilafallnyakni...edltpltdfvkqivvkhiglq......v.....lpehypivgtcliqtmvellppeia.......nkdfleawtiaygnlakllidleaaeyaKQPW**

**.........|..||..|....................|..||..||...|.......|..|..................|..||..||..|..||...............|..|...||.........|.........|..|..|||.||......................||..||..|...|............**

**Candida albicans gi|46432322|EAK91810.1 400aa**

**pltpehiqiiidsvpileh.............ldvqltekfykrllkqnpefkpf..fne...............Thqkllrqprimihfliqyakni...qdltpmidfikkiaskhvglq......v.....kpehypklgqvlinviinlfpkqlv.......hdefieawtlayqnlanllikleseqyvEKPW**

**.........|..||..|....................|..||..||...|.......|..|..................|..||..||..|..||...............|..|...||.........|.........|..|..|||.||......................||..||..|...|............**

**Candida albicans gi|46441212|EAL00511.1 400aa**

**pltkgerkiikdsipiley.............ldiqfgekffkrlikqfpqykpy..fne...............Thlkllrqprsfhhcllefarni...ddlrpmkdfimriaskhvarq......v.....spdqyrvfgqvlvevmmdqfpkefv.......dqefveawtmafrilanilinievreyqDKPW**

**.........|..||..|....................|..||..||...|.......|..|..................|..||..||..|..||...............|..|...||.........|.........|..|..|||.||......................||..||..|...|............**

**Candida albicans gi|46433276|EAK92722.1 563aa (298-437)**

**GSTNTNTMTRLDSTTIAS................SLFCRQLYFNLLSKDPTLEKM..F...................PSIKHQAANMAGILSLTISQL...ENLSILDEYLAKLGKLHSRVLN.....I.....EEAHFKLMGEAFVQTFQERFGSKF........TKELENLWIKLYLYIANTLLQTGIDpvlKLTR**

**.........|..||..|....................|..||..||...|.......|..|..................|..||..||..|..||...............|..|...||.........|.........|..|..|||.||......................||..||..|...|............**

**Candida glabrata gi|50291201|XP_448033.1 432aa (124-267)**

**RKRLIKDksipyqisntsti............egslfcaqfyenliamdqnverm..f...................psirhqavsfsgvlntavdnl...enihvldsylrgigkrhsrilg.....i.....kpayfetmgkafirtfqdrfgiff........tielediwsrlysylangmiafgvdPVLPQSI**

**.........|..||..|....................|..||..||...|.......|..|..................|..||..||..|..||...............|..|...||.........|.........|..|..|||.||......................||..||..|...|............**

**Candida glabrata gi|50293309|XP_449066 398aa mlseqtrtiikatvpvlek.............qgtqitkcfyghmlteheellni..fnr...............Tnqkigaqptalattvlaaakhi...dnlaallphveqighkhralq......i.....lpehypivgkylliaikevlgdva........tpeiitawgeayqeiadvfisveakmykEAAW**

**Cryptococcus neoformans gi|50259618|EAL22289.1 504aa**

**Plteaqkdiikstapileq.............hgvtitthfyknmirahpelrdv..fse...............Saqklghqpaalaaavyayacni...hdltpilpvveriahkhtslh......i.....tanqygivgkhliqaivdilgdav........tpdiadawyngywnlahvfikrerelyDSAIE**

**Debaromyces hansenii gi|50428015|XP_462620.1 403aa**

**ELTPREKELIKASVPILEE.............SGDVLTSKFYNHMLTDFPEVKPF..FNE...............SNQKTMKQPKILAFALLHYAKNI...DDIAPLTAFVNQIVVKHVGLQ......V.....KPEHYPIVGQCLLDTMETLLGPEIA.......TEEFLTAWATAYGNLAQILINAEAEKYKEMEW**

**Debaromyces hansenii gi|50304451|XP_452175.1 395aa**

**mlsvntknivkatvpvleq.............hgvtitkifynnmlsenpvllnt..fnrv...............nqkkgrqptalamtvlaaakni...ddlsvllpavneigqkhralq......v.....epaqydivgkylllaikevlgsaa........tpeiiqawgeaykviadifisvekemykKAAW**

**.........|..||..|....................|..||..||...|.......|..|..................|..||..||..|..||...............|..|...||.........|.........|..|..|||.||......................||..||..|...|............**

**Debaromyces hansenii gi|50428041|XP_462633.1 401aa ELTPREKKLIKASIPILDE...............DVLTSKFYNHMLTDFPEVRPF..FNE...............THQKTMKQPRILAFGLLHYVKNI...DDLAPLTTFVNQIVVKHVGLQ......V.....KPDHYPIVGQCILDTMKTLLGDRIA.......TEEFLTAWATAYGNLAQTLINAEADKYKSNEW**

**.........|..||..|....................|..||..||...|.......|..|..................|..||..||..|..||...............|..|...||.........|.........|..|..|||.||......................||..||..|...|............**

**Eremothecium gossypii gi|45185029| NP_982746.1 436aa (184-339)**

**asmvsqpatpapaldnvntnti..........asslfcaqfyanlldmdanlekl..y...................psikhqavayagvlttaishl...ENlslmdgylsdlgkrhnrilg.....i.....epphfelmgiaflktiqdrfgiyc........tieltetwsrlysylansilqfgidpvlELNH**

**.........|..||..|....................|..||..||...|.......|..|..................|..||..||..|..||...............|..|...||.........|.........|..|..|||.||......................||..||..|...|............**

**Fusarium oxysporum gi|3551511|BAA33011 415aa**

**ALTAAQVAIVKSTAPILKE.............HGKTITTTFYRNMLGAHPELKNY..FS.L..............RNQQTGAQQAALANSVLAYATYI...DDLGKLSHAVERIAHKHVSLF......I.....KPEHYPIVGTHLIGAIGEVLGSAL........TTEIKDAWVAAYGQLADIFIQREGQMYEAAGE**

**.........|..||..|....................|..||..||...|.......|..|..................|..||..||..|..||...............|..|...||.........|.........|..|..|||.||......................||..||..|...|............**

**Giberella zeae gi|3551511|EAA73242 415aa**

**ALTAAQVAIVKSTAPILKE.............HGKTITTTFYRNMLGAHPELKNY..F...................SLRGAQQAALANSVLAYATYI...DDL...SHAVERIAQKHVSLF......I.....KAEHYPIVGTHLIGAIGEVLGSAL........TTEIKDAWVAAYGQLADIFIQREGQLYDAAGE**

**.........|..||..|....................|..||..||...|.......|..|..................|..||..||..|..||...............|..|...||.........|.........|..|..|||.||......................||..||..|...|............**

**Giberella zeae gi|42547868|EAA70711 457aa**

**ALSYQQTKLIRGTIPALTD.............HGERITTIFYRNMLRDHPELNDY..F.....................VGRQPRALTAVILSYANNI......NHLIPKMERMCHKHCSLG......I.....KPEHYAIVEKYLIAAFAEVLGPTM........TTQIREAWTKAYWMLAKMLIGREAQLYRDFGK**

**.........|..||..|....................|..||..||...|.......|..|..................|..||..||..|..||...............|..|...||.........|.........|..|..|||.||......................||..||..|...|............**

**Kluyveromyces lactis gi|50304451|XP_452175.1 395aa**

**mlsvntknivkatvpvleq.............hgvtitkifynnmlsenpvllnt..fnrv...............nqkkgrqptalamtvlaaakni...DDLsvllpavneigqkhralq......v.....epaqydivgkylllaikevlgsaa........tpeiiqawgeaykviadifisvekemykKAAW**

**.........|..||..|....................|..||..||...|.......|..|..................|..||..||..|..||...............|..|...||.........|.........|..|..|||.||......................||..||..|...|............**

**Kluyveromyces lactis gi|50307891|XP_453939 430aa (181-323)**

**ntttskpqtittatisnsm.............ssslfcaqfysnflsmdpdleka..f...................pslkhqdtafagvltmainnl...edltameaylnnlgkrharvlg.....i.....hppqfelmgvallrtirdrfgvyc........tfeleetwarlysylansilqfgidpilKVDY**

**.........|..||..|....................|..||..||...|.......|..|..................|..||..||..|..||...............|..|...||.........|.........|..|..|||.||......................||..||..|...|............**

**Kluyveromyces waltii Kwal_9681 396aa**

**.MVSEQTRTIVKATVPVLE............EHGTAITSVFYKNMLTDH.KELLNI.FNK...............TNQTRGLQPTALATTVLAAAKNI...DDLSVLAPHVVQIGHKHRALQ......V.....KPEHYPIVGQYLLGAIKEVLGDAA........TDEILDAWKEAYGAIAQIFIDVEAGLYKQAAW**

**.........|..||..|....................|..||..||...|.......|..|..................|..||..||..|..||...............|..|...||.........|.........|..|..|||.||......................||..||..|...|.............**

**Kluyveromyces waltii Kwal_22190 421aa (171-314)**

**APAPAAAGMPRKSKYAVTS.............CNSLFGAQFLENIIALAPDLARV..F....................PTIRHVAIGVTGVLSIAISNL..EDLSVLDNYLIGLGKRHARILG.....V.....TADMFEFAGAAFLKTVQERFGVAC........TPELEETWRRIYSFLANSLLQFGIDPTMETRV**

**.........|..||..|....................|..||..||...|.......|..|..................|..||..||..|..||...............|..|...||.........|.........|..|..|||.||......................||..||..|...|.............**

**Kluyveromyces waltii Kwal_4395 460aa (202-352)**

**KISKWGSSFTTGPLPDSDV....KPISSSSALASSIFCHQFYGNLLYMEPSIESM..F...................PSIRHQAVAFAGVLTMTVNNL...ENLSVLEAYLCSMGKRHARILA.....I.....EPPHFELMGMAFLKTIKDRFGVHS........TLELEETWSRLYSYLANSILQFGIDPVLKIDA**

**.........|..||..|....................|..||..||...|.......|..|..................|..||..||..|..||...............|..|...||.........|.........|..|..|||.||......................||..||..|...|.............**

**Kluyveromyces waltii Kwal_24852  543aa (173-315)**

**FSGSRLHDEVERATSAGGG.............PSFVFGTQFLNNILDLAPPIENT..F....................PTIKHAAASVTDVISMAISSL...EDLSVLNDYLDSLGKHHSR.......I.....LGVYAVHFDLAGVAFLKTLRDRFG.....FHCTRELEQLWSRLYVYLANSMLQLGIDPIIDEKA**

**.........|..||..|....................|..||..||...|.......|..|..................|..||..||..|..||...............|..|...||.........|.........|..|..|||.||......................||..||..|...|.............**

**Magnaporthe grisea gi:38101598|EAA48540 447aa**

**SITPEQVAVVKSTAPVLKE.............HGVTITTVFYKNMIDAHPELHNV..FST...............TSQATGAQPRALASAVLAYATHI...DDLARLSHAVSRIAHKHVSLH......I.....TPDQYDIVGKYLIEAIGQVLGDAA........TPEIVAAWIAAYGVLADVFIGVEGKMYQEHSD**

**.........|..||..|....................|..||..||...|.......|..|..................|..||..||..|..||...............|..|...||.........|.........|..|..|||.||......................||..||..|...|............**

**Neurospora crassa gi|28919225|EAA28703 418aa**

**TLTDAQISIVKSTAPVLKQ.............HGEAITTVFYNDLITENPSLKNI..FS.L..............TSQATGAQPRALAHAVLAYATYI...DNLSALSEAVARIAHKHVSLQ......V.....EPAQYAIVGQYLIQAIGKVLGDAA........TPEIVDAWTAAYGVLANVFIGVEGGMYEENEK**

**.........|..||..|....................|..||..||...|.......|..|..................|..||..||..|..||...............|..|...||.........|.........|..|..|||.||......................||..||..|...|............**

**Neurospora crassa gi|28925744|EAA34752 537aa**

**ALTYQQSKLVRDTIPALKE.............HGERITSIFYKTMLTDHPELNNY..FNSV...............NQKNGRQPRALTAVILGFASNI...NHLSELVPKFERMCNKHCSLG......I.....QPEHYEVVGKYLIQAFGEVLGPAM........TPEVQTAWTKAYWMLAKMLIGREAQLYRDFES**

**.........|..||..|....................|..||..||...|.......|..|..................|..||..||..|..||...............|..|...||.........|.........|..|..|||.||......................||..||..|...|............**

**Saccharomyces bayanus ORFP:9664  399aa**

**MLSEHIRTIIKATVPVLEQ.............QGTVITRTFYKNMLAEHTELLNI..FNR...............TNQKAGAQPNALATTVLAAAKNI...DDLSVLMDHVKQIGHKHRALQ......I.....KPEHYPIVGEYLLKAIKEVLGDAA........TPEIISAWGEAYQAIANIFITVENRMYEKASW**

**.........|..||..|....................|..||..||...|.......|..|..................|..||..||..|..||...............|..|...||.........|.........|..|..|||.||......................||..||..|...|............**

**Saccharomyces bayanus ORFP:20532  424aa (171-303)**

**NFDGLISKTSAPTVKPGKI....KKRDNDDKVDTALFCSQFYDNLISMDPLLEEY..F...................PSLRHQAVSFCKVLNSAINNL...EDVHVLDDYIVKLGKRHSR........I..LGIKTVGFEVMGMAFMTTLQDRFGSFF........TLELKNLWGQLYSYLANCMIKAGKDPMGKTKF**

**.........|..||..|....................|..||..||...|.......|..|..................|..||..||..|..||...............|..|...||.........|.........|..|..|||.||......................||..||..|...|............**

**Saccharomyces cerevisiae gi|1362285|S57699|FHP_YEAST 399aa**

**MLAEKTRSIIKATVPVLEQ.............QGTVITRTFYKNMLTEHTELLNI..FNR...............TNQKVGAQPNALATTVLAAAKNI...DDLSVLMDHVKQIGHKHRALQ......I.....KPEHYPIVGEYLLKAIKEVLGDAA........TPEIINAWGEAYQAIADIFITVEKKMYEEALW**

**.........|..||..|....................|..||..||...|.......|..|..................|..||..||..|..||...............|..|...||.........|.........|..|..|||.||......................||..||..|...|............**

**Saccharomyces cerevisiae gi|6324095|NP_014165.1 426aa (154-302)**

**APDGMISKTIDPTARPRKT....KQRDNDNKVDTALFCSQFYDNLIAMDPLLEEY..F...................PSLKHQAVSFCKVLDSAIDNL...ENVHVLDDYIVKLGKRHSRILG.....I.....KTVGFEVMGKAFMTTLQDRFGSFL........TLELKNLWGQLYSYLANCMITAGKDPMEKIQP**

**.........|..||..|....................|..||..||...|.......|..|..................|..||..||..|..||...............|..|...||.........|.........|..|..|||.||......................||..||..|...|............**

**Saccharomyces mikatae ORFP:9249  399aa**

**MLAEKTRSIIKATVPVLEQ.............QGATITRTFYKNMLTEHTELLNI..FNR...............TNQKIGAQPNALAVTVLAAAKNI...DDLSVLIEHVKQIGHKHRALQ......V.....KPEHYPIVGKYLLRAIKEVLGDAA........TPDIINAWGEAYQTIADIFITVEKQMYEEASW**

**.........|..||..|....................|..||..||...|.......|..|..................|..||..||..|..||...............|..|...||.........|.........|..|..|||.||......................||..||..|...|............**

**Saccharomyces mikatae ORFP:18051  410aa (135-290)**

**FLNTTFDGMITKTIDPAVKARKGKKKGNDDKIDTALFCSQFYDNLIAMDPLLEEY..F...................PSLRHQAVSFCKVLNSAIENL...EDVHVLDDYIVKLGKRHSR........I..LGIKTVGFEVMGKAFMTTLQDRFGSFL........TLELKNLWGKLYSYLANCMITAGKDPMEKTKS**

**.........|..||..|....................|..||..||...|.......|..|..................|..||..||..|..||...............|..|...||.........|.........|..|..|||.||......................||..||..|...|............**

**Saccharomyces paradoxus ORFP:9231  399aa**

**MLAEKTRAIIKATVPVLEQ.............QGTVITRTFYKNMLTEHTELLNI..FNR...............TNQKVGAQPNALAVTVLAAAKNI...DDLSVLMNHVKQIGHKHRALQ......I.....KPEHYPIVGEYLLKAIKEVLGDDA........TPDIINAWGEAYQAIADIFITVEKKMYEEALW**

**.........|..||..|....................|..||..||...|.......|..|..................|..||..||..|..||...............|..|...||.........|.........|..|..|||.||......................||..||..|...|............**

**Saccharomyces paradoxus ORFP:18484 426aa (155-306)**

**TLDGMISNTIDPTVRLRKS....KQKDNDSKVDTALFCSQFYDNLIAMDPLLEEY..F...................PSLKHQAVSFCKVLNSAIDNL...ENVHVLDDYIVKLGKRHSRILG.....I.....KTVGFEVMGKAFVTTLQDRFGSFL........TLELKNLWGQLYSYLANCMITAGKDPMEKIEP**

**.........|..||..|....................|..||..||...|.......|..|..................|..||..||..|..||...............|..|...||.........|.........|..|..|||.||......................||..||..|...|............**

**Schizosaccharomyces pombe gi|19115929|NC003424 427aa**

**ELNESQKQYIRSSIPILES.............SGVNLTKAFYQKMLGNYPEVLPY..FNKA...............HQISLSQPRILAFALLNYAKNI...DDLTSLSAFMDQIVVKHVGLQ......I.....KAEHYPIVGHCLLSTMQELLPSDVA.......TPAFLEAWTTAYGNLAKILIDSEKKVYQSQPW**

**.........|..||..|....................|..||..||...|.......|..|..................|..||..||..|..||...............|..|...||.........|.........|..|..|||.||......................||..||..|...|............**

**Yarrowia lipolytica gi|50548235|XP_502088 374aa**

**mltdaqikiikdsapflae.............ngtdfaakmykymfatypevirf..fnq...............Sdqknlaqpkilahalvayasni...dnlgvlsdfveqivvkhvglq......i.....qpeqypivaasiihtlkemlgeaa........tpefieawtvaytqlanilidaekklyeKQAS**

**.........|..||..|....................|..||..||...|.......|..|..................|..||..||..|..||...............|..|...||.........|.........|..|..|||.||......................||..||..|...|............**

**Yarrowia lipolytica gi|50550817|XP_502881 463aa (186-325)**

**lmsdpesleqstaygtpt................alfceqfytnlmashaelasi..f...................psikrqsvavagvfglaissl...erieemdeflrsvgkrhnrm.......i...gvepihyrwlgeamvktfadrfgdrf........tlevetawikiysylahkllasdeepnvLMFA**

**.........|..||..|....................|..||..||...|.......|..|..................|..||..||..|..||...............|..|...||.........|.........|..|..|||.||......................||..||..|...|............**

**Yarrowia lipolytica gi|50543406|XP_499869 471aa (194-333)**

**lmndpetlessaaygtpt................alfceqfytnlmashaeltsi..f...................psikkqsvavagvfglaiksl...dhiekldeflwsvgkrhnrm.......i...gvepihyrwlgeamiktfadrfgdsF........tlemetawikiysylankllaadeePNVLMFA**

**.........|..||..|....................|..||..||...|.......|..|..................|..||..||..|..||...............|..|...||.........|.........|..|..|||.||......................||..||..|...|.............**

**DIPLOMONAD FHb**

**Giardia lamblia (intestinalis) gi|27981493| 458aa**

**TLSEDTLRAVEATAGLIAA.............QGIEFTRAFYERMLTKNEELKNI..FNLA...............HQRTLRQPKALLDSLVAYALNIRRINELYELKGKGLPVPPEHWAELQGFFSAAERVANKHTSFG......I.....QPAQYQIVGAHLLATIEDRITK..........DKDILAEWAKAYQFLADLFIKREEEIYAATEG**

**.........|..||..|....................|..||..||...|.......|..|..................|..||..||..|..||.................................|..|...||.........|.........|..|..|||.||......................||..||..|...|.............**

**MYCETOZOAN FHbs**

**Dictyostelium discoideum gi|5821408|BAA83810**

**SLSQQSISIIKATVPVLQV.............HGVNITTTFYRNMFKANPQLLNI..FNH...............SNQREGKQQNALANTVLQAAIHI.....DKLNELNLAPIVHKHVALG......V.....LPEHYPIVGTNLLGAIKEVLQDAA........TDEILGAWGEAYGVIAQAFIDAEAALYKVTEE**

**.........|..||..|....................|..||..||...|.......|..|..................|..||..||..|..||...............|..|...||.........|.........|..|..|||.||......................||..||..|...|............**

**Dictyostelium discoideum gi|5821410|BAA83811**

**MLSQKSIQIIKSTVPLLEK.............YGVEITSLFYKNMFEAQPQFLNI..FNH...............SNQRNQKQPVALANTILQSAIHI.....EKLNEINLMPIVHKHVALG......I.....TPEMYPIVGAHLLGAMKTVMQDEA........TPEIMAAWTEAYRAVAQAFMDAEEDLYFETEE**

**.........|..||..|....................|..||..||...|.......|..|..................|..||..||..|..||...............|..|...||.........|.........|..|..|||.||......................||..||..|...|............**

**ARCHAEAL GCSs/Pgbs**

**Aeropyrum pernix (Crenarcheota) gi|14600601|NP_147118 Pgb 195aa (30)**

**>Aerper**

**.vmlgekdvmylkkacdvlkdqvdeildlwygwvasne.hliyyfsnp.dtgepikeylervrarfgawildttc.rdynrewldyqyevglrhhrskkgvtdgv.rtvphiplryliafiypitati.kpFlakkggspediegmynawfksvvlqvaiwshpytk.**

**..........|..||..|........|..||..||...|.....|..|.......|..||..||..|..||...............|..|...||.........|.....|..|..|||.||.......................||..||..|...|..........**

**Haloarcula marismortui (Euryarcheota) gi|55229828|AAV45247 GCS 497aa (44)**

**>Halmar**

**tgfdgsdvdnltamadetna.raeavvddfydhlqsfd.etveifgrs....tksvdqlkntqtqylrdlvagty....dkqyfenrarigkihdmldlgpkiyl...gaysiyfehflrtivedlqsg...........daardealeemqsralsvfkllnldqq.187**

**..........|..||..|........|..||..||...|.....|..|.......|..||..||..|..||...............|..|...||.........|.....|..|..|||.||........................||..||..|...|.........**

**Haloferax volcanii (Archaea; Euryarchaeota) gnl|TIGR_309800|contig:441:h_volcanii**

**VRLSDADERRLSDLEPLLDE.FADEFAAEFYDHLGEHATTKF..FGR....STKTTEMLRADQATYLRELGRGEYG....EDYFAKRARIGKLHDMIDLGPKFYLGAY**

**..........|..||..|........|..||..||...|.....|..|.......|..||..||..|..||...............|..|...||.........|.....|..|..|||.||........................||..||..|...|.........**

**Halobacterium salinarum (Euryarcheota) gi:15790497|NP_280321 GCS 489aa (39)**

**>Halsal**

**tgidddtmaalaaeqplfea.Tadalvtdfydhlesye.rtqdlfan....stktveqlketqaeyllglgrgey....dteyaaqrarigkihdvlglgpdvyl...gaytryytglldaladdvvad..........rgeeaaaavdelvarflpmlklltfDQQ.183**

**..........|..||..|........|..||..||...|.....|..|.......|..||..||..|..||...............|..|...||.........|.....|..|..|||.||........................||..||..|...|.........**

**Methanosarcina acetivorans (Euryarcheota) gi|20091705|NP_617780 Pgb 195aa (31)**

**>Metace**

**.vmftaedeeyiqkagevLEdqveeildtwygfvgsh.phllyyftsp.d.gtpnekylaavrkrfsrwildtcn.rsydqawldyqyeiglrhhrtkknqtdnv.esvpnigyrylvafiypitatm.kpflarkghtpeevekmyqawfkattlqvalwsypyvk.**

**..........|..||..|........|..||..||...|.....|..|.......|..||..||..|..||...............|..|...||.........|.....|..|..|||.||.......................||..||..|...|..........**

**Methanosarcina barkeri (Euryarcheota) gi|48837815|ZP_000294773195 Pgb 197aa (31)**

**>Metbar**

**.vmfteedekylkkagevledqveeildtwygfvgsh.phllyyftsp.d.gtpneeylaavrkrfskwildtcn.rnydqawldyqyeiglrhhrtkknrtdnv.esvpninyrylvafiypitati.kpflarkghtseevekmhqawfkatvlqvalwsypyvk.**

**..........|..||..|........|..||..||...|.....|..|.......|..||..||..|..||...............|..|...||.........|.....|..|..|||.||.......................||..||..|...|..........**

**BACTERIAL Pgbs**

**Chloroflexus aurantiacus (Chloroflexi) gi|22974473|ZP00020712 227aa (63)**

**.VLWTAEDEQYLRMAGEVLGDQVDAILDLWYGFVASH.AHLVYYFTGS.D.GQPIADYLSRVRQRFGQWILDTCR.RPYDQDWLNYQMEIALRHYRTKKNQTDGV.QSVPMIPLRYMIAFIYPITATI.REFLARKGHSAAEVDRMHQAWFKSIVLQVTLWSYPYTR.**

**..........|..||..|........|..||..||...|.....|..|.......|..||..||..|..||...............|..|...||.........|.....|..|..|||.||.......................||..||..|...|..........**

**Rubrobacter xylanophilus (Actinobacteria) gi|46106825|ZP_00200180 196aa (32)**

**.vlftgederylrmagevlegrldelldvwygfvadh.shlvyyfssp.e.gepiqeylervrerfkrwvldacr.rpydqewldyqqeialrhtrekknrtdgv.eapeevslrymisfiypitatv.rpfleeggrpaedvekmhqawfkavvlhvtlwsqpyar.**

**..........|..||..|........|..||..||...|.....|..|.......|..||..||..|..||...............|..|...||.........|.....|..|..|||.||.......................||..||..|...|..........**

**Thermobifida fusca (Actinobacteria) gi|48836482|ZP_00293478 197aa (33)**

**.vmftsadeealrmagdvledqvedvldvwygfvadh.phllayfstp.d.ghpiqeyldrvrerfgqwildtcr.rpynqewldyqqeialrhtpekknvtdna.nsvdniplryviafiypvtatl.rpflakkghsadqveamyqawfksvtmqialwsqpytr.**

**..........|..||..|........|..||..||...|.....|..|.......|..||..||..|..||...............|..|...||.........|.....|..|..|||.||.......................||..||..|...|..........**

**BACTERIAL GCSs**

**Acidiothiobacillus ferrooxidans (Gammaproteobacteria) TIGR243159|contig:10428 880aa (13)**

**LGLQDSDFQVIDRYRDALDK.EASALAHAFYDYLLSH.PATAAVFRDFSSA...RLDALIQKQTEHAKGLLVSRLD....RPWRESMRKIGALHHHLGIGPSW.I..AGAYILYWRHWQKILQVDVVA....DRGEEAAAAVDERDLLRDALFRLLVGDLMVQLEGY.153**

**..........|..||..|........|..||..||...|.....|..|.......|..||..||..|..||...............|..|...||.........|.....|..|..|||.||........................||..||..|...|.........**

**Agrobacterium tumefaciens (Alphaproteobacteria) gi|15888368|NP_354049 499aa (18)**

**LGLGHGERQNLSDMKGVITG.SLDASLDRFYTKVRAV.PETAKFFSSEAHIHHAKSMQLKHWSRIASGTFNEDYTN.........AVTAIGRTHARLGLEPRWYI...GGYALMLDGIVKAVIESMWP....RGLLAKGGSDRVKDALSATIKAALLDMDYSISVYL.168**

**..........|..||..|........|..||..||...|.....|..|.......|..||..||..|..||...............|..|...||.........|.....|..|..|||.||........................||..||..|...|.........**

**Azoarcus sp. EbN1 (Betaproteobacteria; Rhodocyclales) 698aa YP_158656.1 GI:56477067**

**........mrlramdagvae.arsgfadafydylrsyaplaal.lhd.....edavsrlkaahdryfaeltege....ygfdyvqrrlkvglvhaqig......lepkwyvgafrkyladmlpalwd....qtggerdrflaacdsllkvvvfdlcltldtyihadkr**

**..........|..||..|........|..||..||...|.....|..|.......|..||..||..|..||...............|..|...||.........|.....|..|..|||.||........................||..||..|...|.........**

**Azotobacter vinelandii (Gammaproteobacteria) gi|23104391|ZP_00090857 472aa (23)**

**LLLGQFPAPVVAQIRELATT.HQSELPGYFYEQMLQDE.QAML.FLTHE....QVKSRLHGTLRQWIVSVFSMSDDDAALQALIAQQKQIGEIHARIKIPIHLVL...RGARHLRERLFVLLRESDI.........PRQRKLFGQRLISETVDLAMEIMSRAFSDAY.168**

**..........|..||..|........|..||..||...|.....|..|.......|..||..||..|..||...............|..|...||.........|.....|..|..|||.||........................||..||..|...|.........**

**Bacillus anthracis (Firmicutes) gi|30265443|BA5673 434aa (42)**

**LHISKEDLQIVKVLQPFIYE.EIDWITEKFYANITKQ.PNLITIIERYSS.....IPKLKQTLKTHIKELFSGDMH....EDFIEQRVKIAKRHVQIGLHRKW.Y..TAAYQELFRSIMKILKTQVP..........ESDFSYSINVINKLFTLEQELVIAAYESky.185**

**..........|..||..|........|..||..||...|.....|..|.......|..||..||..|..||...............|..|...||.........|.....|..|..|||.||........................||..||..|...|.........**

**Bacillus cereus (Firmicutes) gi:30023454|NP_835085 434aa (42)**

**LHISKEDLQIVKVLQPFIYE.EIDWITEKFYSNITKQ.PNLITIIERYSS.....IPKLKQTLKTHIKELFSGDMH....ENFIEQRVRIAKRHVQIGLHRKW.Y..TAAYQELFRSIMKILKTSSD........YSAAEFSYSINVINKLLTLEQELVIASYESEY.185**

**..........|..||..|........|..||..||...|.....|..|.......|..||..||..|..||...............|..|...||.........|.....|..|..|||.||........................||..||..|...|.........**

**Bacillus clausii (Firmicutes) 449aa YP_176973.1 GI:56965241 (36-**

**fidlhasdleyihllyplvnehiapiteafyeklleq.phlrqlvtt.....hssidalkqtlkqhilemfegkvd....aaffakrkriaqthvriglktkwyi...gafhnlqlelqrmivkhfk.............sgneqflafcavgkfinfeqqlvleaye**

**..........|..||..|........|..||..||...|.....|..|.......|..||..||..|..||...............|..|...||.........|.....|..|..|||.||........................||..||..|...|.........**

**Bacillus halodurans (Firmicutes) gi|15613068|NP_241371 441aa (44)**

**IHLTLDDLKRMKALQPLVEE.NMEVLADAFYSNIIKQ.PNLNEIIETHSS.....VERLKETLKQHILEMFNGEID....QAFLQKRLQIAQAHVRIGLQTKW.Y..VSAFQQLTDSLIQLLEQSSD........YSAAEIVLATRSLLKLLNLEQQLVLEAYENKR.188**

**..........|..||..|........|..||..||...|.....|..|.......|..||..||..|..||...............|..|...||.........|.....|..|..|||.||........................||..||..|...|.........**

**Bacillus licheniformis 430aa YP_090716.1 GI:52784887 (39)**

**vdltdedlwvlsrlkplvte.hietivtrfyqnlehesslme..iih....dnssvdrlkktltihiqemfsgvid....eaflekririahvhlrigllpkw.y..lgafqdlllsmlkifenals..........deeyrrsvkavtkilnieqqivleafedeha**

**..........|..||..|........|..||..||...|.....|..|.......|..||..||..|..||...............|..|...||.........|.....|..|..|||.||........................||..||..|...|.........**

**Bacillus subtilis (Firmicutes) gi:37927254|NP388919 GCS 433aa (40)**

**VRLGDAELYVLEQLQPLIQE.NIVNIVDAFYKNLDHE.SSLMDIINDHSS.....VDRLKQTLKRHIQEMFAGVID....DEFIEKRNRIASIHLRIGL.....L.PKWYMGAFQELLLSMIDIYEAS............ITNQQELLKAIKATTKILNLEQQLVLE.178**

**..........|..||..|........|..||..||...|.....|..|.......|..||..||..|..||...............|..|...||.........|.....|..|..|||.||........................||..||..|...|.........**

**Bacillus thuringiensis (Firmicutes) gi|49481265|YP_039413 433aa (41)**

**lhiskedlqivkvlqpfiye.eidwitekfytnitkq.pnlitiieryss.....ipklkqtlkthikelfsgdmh....edfieqrikiakrhvqiglhrkw.y..taayqelfrsimkilqtkit.........tiddfsysinvinklftleqelviaayesey.185**

**..........|..||..|........|..||..||...|.....|..|.......|..||..||..|..||...............|..|...||.........|.....|..|..|||.||........................||..||..|...|.........**

**Bordetella bronchiseptica (Betaproteobacteria) gi|33600945|NP_888505 475aa (13)**

**DTCAHYSPHEWAAARNVVTA.NKAALADYFYECMLAD.PNAAF.FLS....DQLVKTKLHASMQDWLESVYAAAPTEEY.ERTVAFQRKVGEVHARIDIPVHL.V..MRGACALIRRICELLDRDAS.......LSAAQAAATCRYVADVTMTAVEMMCHAYSVSHD.162**

**..........|..||..|........|..||..||...|.....|..|.......|..||..||..|..||...............|..|...||.........|.....|..|..|||.||........................||..||..|...|.........**

**Bordetella pertussis (Betaproteobacteria) gi|33594381|NP_882025 475aa (69)**

**DTCAHYSPHEWVAARNVVTA.NKAALADYFYECMLAD.PNAAF.FLS....DQLVKTKLHASMQDWLESVYAAAPTEEY.ERTVAFQRKVGEVHARIDIPVHL.V..TRGACALIRRICELLDRHYR.......KDPKRAAATCRYVADVTMTAVEMMCHAYSVSHD.162**

**..........|..||..|........|..||..||...|.....|..|.......|..||..||..|..||...............|..|...||.........|.....|..|..|||.||........................||..||..|...|.........**

**Burkholderia fungorum (Betaproteobacteria) gi|22984913|ZP_00030046 724aa (22)**

**HLYSQARASALTSLTEVLRH.NAVEIVKRFYDGLIRL.PKSKHTLAA...LSEHELQHLKTQQIQNLYALASPDLT...AMDHRTMALRVGRIHAIVGLEWEDLIRSRGILSAAIHDTLDTTvhgial.......avlgrrltqdlawqteayqrlqtsrqdVLMRV.173**

**..........|..||..|........|..||..||...|.....|..|.......|..||..||..|..||...............|..|...||.........|.....|..|..|||.||........................||..||..|...|.........**

**Carboxydothermus hydrogenoformans (Firmicutes) TIGR246194|contig:4013 251 (17)**

**LNLTAEDLQLMAEFKELFIQ.KAQEFVNKFYQHLTKF.PYLQELIKK.....HSTVEKLSKTQAEYFISLTSEKID....ADYIKNRLAVGKKHMEIALYPNWYI...GAYRLYYEVVGELVARKYS...........PGTELYFKAVNAFYKRINFDIQLAIENYI..92**

**..........|..||..|........|..||..||...|.....|..|.......|..||..||..|..||...............|..|...||.........|.....|..|..|||.||........................||..||..|...|.........**

**Caulobacter crescentus (Alphaproteobacteria) gi|16124683|NP_419247 537aa (17)**

**MGIDDKARSALRDLRPVIR.AEIGKALDNFYGKVRAT.PETRKFFSD.....DRHMNAASSRQQAHWGVIAEGQFS....DDYVQAVRAIGQTHARIGLEPRWYI..GGYAVVGDHLVRAVIDSELK..GLFMEKKAKKAGEAVAALMKAIFLDMDFAISIYLETLE.170**

**..........|..||..|........|..||..||...|.....|..|.......|..||..||..|..||...............|..|...||.........|.....|..|..|||.||........................||..||..|...|.........**

**Caulobacter crescentus (Alphaproteobacteria) gi|16126556|NP_421120 555aa (55)**

**MRFDERSRAHLRAIKPVID.AEIGAALGQFYSQVRLF.PDTRVKFRD.....DGHMAGAERAQAAHWRRIAEAGYG....ESYVRDVERIGRSHADADIAPQWYI..GGYAVVVEEVMRALVAKRAK.GLFNSAKSDAELADGLSALIKAAFLDMDLSVStyidvll.211**

**..........|..||..|........|..||..||...|.....|..|.......|..||..||..|..||...............|..|...||.........|.....|..|..|||.||........................||..||..|...|.........**

**Chromobacterium violaceum (Betaproteobacteria) gi|34495694|NP_899909 375aa (25)**

**falaprdemllraagnlves.hleelvtrfyelqtst.peialligda.....dtlqrlrsaqrryvvdlfsgiy....dleyvnirlriglvhkrigvepk..l....ylaavdslkfllaeklte.lipdaevrlhtlqaldklmmfdvalvfetyirslvAEIE.174**

**..........|..||..|........|..||..||...|.....|..|.......|..||..||..|..||...............|..|...||.........|.....|..|..|||.||........................||..||..|...|.........**

**Chromobacterium violaceum (Betaproteobacteria) gi|34496333|NP_900548 295aa (15)**

**faiqeddlkrvramgeavlp.rldeamdrfyewlpsl.peyeglfarp.....salrnareaqaaywrsffsgvv....daaylaervcagethariglp....l..ssyfagvnyaftlfcgylks.........gsretasqtllstakllhmdtalvvetysrl.156**

**..........|..||..|........|..||..||...|.....|..|.......|..||..||..|..||...............|..|...||.........|.....|..|..|||.||........................||..||..|...|.........**

**Desulfitobacterium hafniense (Firmicutes) gi|23118817|ZP_00102172 157aa (15)**

**lkleqdqllllkefkpvmeq.dvdqivdqfyahitki.sqlngiikef.....ssverlrglqrnyllsifpdvi....degyimsririgdvhkrinlppfvyl...ssyqtffdlilprifahyr........kkpeqalrlslavlrifsfdqqvvmasyi....157**

**..........|..||..|........|..||..||...|.....|..|.......|..||..||..|..||...............|..|...||.........|.....|..|..|||.||........................||..||..|...|.........**

**Desulfotalea psychrophila (Deltaproteobacteria) gi|51244053|YP_063937 363aa (24)**

**LFFTDEDVQVLVSLKEVVSE.NIEEIVEAFYERVIPFD.EMDGVIGDA.....ETLRRLKNYQRSYIKTLFAGQY....DEEYVHSRLRVGVVHKRIG......V.EPKFYISAIHTLLTILREIIT....KNSSNDCQACVRNLRAVEKIIMFDLSLTFDTYINSL.170**

**..........|..||..|........|..||..||...|.....|..|.......|..||..||..|..||...............|..|...||.........|.....|..|..|||.||........................||..||..|...|.........**

**Desulfuromonas acetoxidans (Deltaproteobacteria) 301aa ZP_00550754.1 GI:68177608**

**yqftveeadilhklqprmga.laekfisefydyiwgfgktaq..flk....nkdiishhrvkikqwflnlfcgdy....dltyftnlykigeihvkig......l..pthyvnsaftftrtfiikns....vdekvdkkkriaelaaiekiidmnldvltssyREEEL**

**..........|..||..|........|..||..||...|.....|..|.......|..||..||..|..||...............|..|...||.........|.....|..|..|||.||........................||..||..|...|.........**

**Erwinia carotovara (Deltaproteobacteria) gi|50120615|YP_049782 442aa**

**.miattsqqsfnllrtlavq.kasdfadefyiymlkdq.easl.flss....qqvhdrlhgsmskwiadiltntgds..ladlinhqkkigqiharigipvd..l.vergarrlkwhlyehiaqvad.....dkalCfdamrfasismdiaieimsktysqshDLAA.149**

**..........|..||..|........|..||..||...|.....|..|.......|..||..||..|..||...............|..|...||.........|.....|..|..|||.||........................||..||..|...|.........**

**Escherischia coli (Gammaproteobacteria) gi|15801648|NP_287665 460aa (13)**

**GLVEQADPPIRAKAAEIAVA.HAHYLSIEFYRIVRID.PHAEE.FLSN....EQVERQLKSAMERWIINVLSAQVDD..VERLIQIQHTVAEVHARIGIPVE..I.VEMGFRVLKKILYPVIFHAKC............EKARKLQVYHFSINSIDIAMEVMTRAFT.156**

**..........|..||..|........|..||..||...|.....|..|.......|..||..||..|..||...............|..|...||.........|.....|..|..|||.||........................||..||..|...|.........**

**Exiguobacterium (Firmicutes) gi|45532973|ZP_00183969 430aa (37)**

**IDLtvedlqivrlirhdvae.wmpqmvdafyaelvrv.pelkqlierh.....stlerlkgtlakhilqmfdgqv....tpdyinvrkriadrhvriglqnkwyi...aafqkvlnvlsrqiedstlpes..eqvrivrsagklfnleqqivltmyeemVdaerqtv.188**

**..........|..||..|........|..||..||...|.....|..|.......|..||..||..|..||...............|..|...||.........|.....|..|..|||.||........................||..||..|...|.........**

**Exiguobacterium (Firmicutes) gi|46113120|ZP_00182337 425aa (30)**

**mgyteehlqtlkamapvvns.ildevleqvldhlllh.pemvqiaqts.....strerlkkvfadyfgslltgnm....ddkflamrtrmgkthnrnfvpvtwfi...asyaafntllipkivehfqhepa........qltnailalnhamnldaqivtsqyvdar.175**

**..........|..||..|........|..||..||...|.....|..|.......|..||..||..|..||...............|..|...||.........|.....|..|..|||.||........................||..||..|...|.........**

**Geobacillus kaustophilus (Firmicutes) 324aa BAD76281.1 GI:56380373 GCS**

**maqieiemvgdgiyrvpipvpfpmkyvycyvgretdgwsivdvgfrypeairaweavfrqlgikprhvraiyithfhpdhfglagwmqqqteapvwisepdyamaervwgeesvqacevgamfrrhgvpdelvtdieesmwklslrvspf**

**..........|..||..|........|..||..||...|.....|..|.......|..||..||..|..||...............|..|...||.........|.....|..|..|||.||........................||..||..|...|.........**

**Geobacter metallireducens (Deltaproteobacteria) gi|48844286|ZP_00298606 300aa (11)**

**YLFGDEDAETLKSLLSIAQA.NRELMIEDFYDYLLGI.PETAA.FLQ....DDTVLQRLKLSHGGWFVNLFRGVY....DNQYLHDLQRVGHVHVKIGLNAHF.V..NAAMQKVRRFAVGMIRENFPDRD.....ERRKKTEAVEKILDINLDIMTASYIEEELKKV.158**

**..........|..||..|........|..||..||...|.....|..|.......|..||..||..|..||...............|..|...||.........|.....|..|..|||.||........................||..||..|...|.........**

**Geobacter sulfurreducens (Deltaproteobacteria) gi|39998400|NP_954351 300aa (11)**

**YRFTDEDAELLGSLFPLAET.NKERLADQFYDYLLGI.PETAE.FLK....EDLVLQKLKQTHQDWFVSLFAGSY....DNRYIHNLQKIGHAHVRVGLNAHY.V..NVAMNVVRQFTLSIIQHLQSPSD......RRQRREAVEKILDINLDIMSASYREEEMRKF.158**

**..........|..||..|........|..||..||...|.....|..|.......|..||..||..|..||...............|..|...||.........|.....|..|..|||.||........................||..||..|...|.........**

**Gluconobacter oxydans (Alphaproteobacteria) 458aa YP_191196.1 GI:58039232**

**lgigaaekahlarirpqalg.vlgpaldrfygrihkt.palnrffan....eqhriga.rtqqekhwkrilqgdfg....adyvestraignthariglkpqwyv..ggyalliegmvtdllrqywpq.........prrsfldrllrrpargtsgeeaaaqiglli**

**..........|..||..|........|..||..||...|.....|..|.......|..||..||..|..||...............|..|...||.........|.....|..|..|||.||........................||..||..|...|.........**

**Magnetococcus sp.MC-1 (Unclassified proteobacteria) gi|48832082|ZP_00289126507 507aa (20)**

**VGFTEKDASILKKLRPVAAK.HATAVVNTFYTRLSGFA.HLEKIIGG....AGSSVERLKRTQEEYLVQLFDGEY....GRDYFVRRWRIGQIHNKIGLEPDWYL...GGYSLYRQLLLPILLDKITTID.........DVQRAMAAIDKILTLDSELAIGSYIDAV.166**

**..........|..||..|........|..||..||...|.....|..|.......|..||..||..|..||...............|..|...||.........|.....|..|..|||.||........................||..||..|...|.........**

**Magnetococcus sp.MC-1 (Unclassified proteobacteria) gi|22998615|ZP_00042662 467aa (14)**

**DIYLGVDAEKVNFIGDLIKD.RLNQTVERFYIELLEVESARFF.LDS.....ALVKERLHGSLTEWLQMLFSHKD.DDTLEQMFAFQKNIGNVHARINIPMHLVV...EGMRILRREIICFLSKYSPGTE.........LLVDLVVLVGEVLDHNLSLINESYVRNN.159**

**..........|..||..|........|..||..||...|.....|..|.......|..||..||..|..||...............|..|...||.........|.....|..|..|||.||........................||..||..|...|.........**

**Magnetospirillum magnetotacticum (Alphaproteobacteria) gi|23014982|ZP_00054774 443aa (11)**

**LQIDEDTKRALREFREVLSR.HIDGVLDTFYRHVSNN.PATAKMFAN.....PDRMAHARSMQKKHWMESVFLGQ...FDDRYFAQVTEIGKVHQRIGLDPKWYT...AGYCFVLNMVIGVAVEGSLYSD........ELTQVLAAVNKAAFLDMDLATSVYIETNT.157**

**..........|..||..|........|..||..||...|.....|..|.......|..||..||..|..||...............|..|...||.........|.....|..|..|||.||........................||..||..|...|.........**

**Magnetospirillum magnetotacticum (Alphaproteobacteria) gi|23014248|ZP_00054075 732aa (35)**

**LRLDDDAISTVKSVRQMVES.SLPGIADGFYAHLMQW.PALKALLGG.....GAKIGHLKETQQAHWASLFSGRF....DDDYFTRAVAIGAAHERIGLEVNWYL..GGYCFVLEKLMAELAKGLRSEA..........FPQMAGAVLRAAFLDMDLAISTYIEHGE.178**

**..........|..||..|........|..||..||...|.....|..|.......|..||..||..|..||...............|..|...||.........|.....|..|..|||.||........................||..||..|...|.........**

**Moorella thermoacetica (Firmicutes) gi|49235973|ZP_00330036 245aa (13)**

**ltlseaelglmeaekeifik.eadavvktfydhllqy.pylenmikqh.....stlerltqtqkayfisltapri....dgeyisgrlrigkkhqkiglypkwyl...gayriylseirrvIwhyhaddp...........dlclrlleaftkriifdmqlaIENYI.156**

**..........|..||..|........|..||..||...|.....|..|.......|..||..||..|..||...............|..|...||.........|.....|..|..|||.||........................||..||..|...|.........**

**Novosphingobium aromaticivorans (Alphaproteobacteria) gi|48849793|ZP_00304036 481aa (17)**

**FNIDHKDFERFPHIAKVLEN.YAPPALDKLYDQIATT.PETASFFGS.....RQAMRHARDKQIEHWAGMFSGRA....DRSYFESAERIGNVHARIGLEPGWYI...GGYAMVLEQVINAMFSGIGGIL.....GAKRTARSVGSLVKMALLDMEVALSTYFRAEE.167**

**Pelobacter propionicus (Deltaproteobacteria) 299aa ZP_00677957.1 GI:71838196 (9)**

**hyrftdgdaelllslqslaqehldsfsqefydylygl.pdtaa.ilnk.....cnrvhlrqmhntwflslfsgiy....dnhylshlvrighahvrvglnvhf.v..naamnqvrhfllnlidgsyhdre.....qrrllreavekmldmnldvmgssyreeemkkvf**

**..........|..||..|........|..||..||...|.....|..|.......|..||..||..|..||...............|..|...||.........|.....|..|..|||.||........................||..||..|...|.........**

**Rhodobacter sphaeroides (Alphaproteobacteria) gi|46192658|ZP_00207372 341aa**

**..................MN.EAVGRALDRFHERMRQT.SAAG.FFAD....ATHMDSAKSRQARHWARLASGEI....DAAYVEEAVRVGRTHARIGLEPRWYL..GGYALILEEIVQTMLPRMAGRGF.FGRRRATRAAHALGYIVKVALLDMDYGVSTYFDAVQ.135**

**..........|..||..|........|..||..||...|.....|..|.......|..||..||..|..||...............|..|...||.........|.....|..|..|||.||........................||..||..|...|.........**

**Rhodospirillum rubrum (Alphaproteobacteria) gi|22966565|ZP_00014161 442aa (11)**

**LKADARLTEDLNEIHPLMVS.MIDDLLGEFYDTVSRT.PELYAMFGS.....AQSVERARLAQRRHWVEVLFKGD....WKAHASQAQRIGKAHVDRGITPS..I.YFAAYSHVLCGLTGRMAQDNFPD...PEELARGLRAAIRAVYIDMLAVLDVYFAEERDRAS.160**

**..........|..||..|........|..||..||...|.....|..|.......|..||..||..|..||...............|..|...||.........|.....|..|..|||.||........................||..||..|...|.........**

**Shigella flexneri (Gammaproteobacteria) gi|24113095|NP_707605 381aa (13)**

**GLVEQADPLIRAKAAEIALA.HAHYLSIEFYRIVRID.PHAEE.FLS....NEQVERQLKSAMERWIINVLSAQV..DDVERLIQIQHTVAEVHARIGIPVE..I.VEMGFRVLKKILYPVIFDASL............SAAQKLQVYHFSINSIDIAMEVMTRAFT.156**

**..........|..||..|........|..||..||...|.....|..|.......|..||..||..|..||...............|..|...||.........|.....|..|..|||.||........................||..||..|...|.........**

**Silicibacter sp. TM1040 (Alphaproteobacteria) gi|52011617|ZP_00338974 308aa (25)**

**fditpadqervrslapiadh.vgeeiiadfyahilrfe.daaqqfad.....dtqiervkrgqkkyfqelinael....desyaeerkriggiheragitptlyv..gsyayylqrlsqsilengke............dphklfglvlsmmkiahydmalaletYV.167**

**..........|..||..|........|..||..||...|.....|..|.......|..||..||..|..||...............|..|...||.........|.....|..|..|||.||........................||..||..|...|.........**

**Silicibacter sp. TM1040 (Alphaproteobacteria) gi|52008958|ZP_00336325 485aa (11)**

**ydldganqallqkrakmilg.llpevldhfydrvgre.pemaaffkt.....eammerakgeqlqhwsrlfsgdf....gedyiqsarnigrvhtriglpfs.fF..nagyahanahiqalilkrhsg...glmrratethallgvlsramaldiqlifdahaEAMQ.157**

**..........|..||..|........|..||..||...|.....|..|.......|..||..||..|..||...............|..|...||.........|.....|..|..|||.||........................||..||..|...|.........**

**Silicibacter sp. TM1040 (Alphaproteobacteria) gi|52008710|ZP_00336080 487aa (12)**

**fglgeedrssivgmaplver.hidavlddfydlclsr.petkgyfpt.....eqivahakaaqrahwvklfsgrf....ddsymssadkvgrvhfevdlpfhlFL...ggyatvgdrilkvvltqkng.....wrgtkarmkqaralqrlilfdcerviagyidaql.160**

**..........|..||..|........|..||..||...|.....|..|.......|..||..||..|..||...............|..|...||.........|.....|..|..|||.||........................||..||..|...|.........**

**Sinorhizobium meliloti (Alphaproteobacteria) gi|15964389|NP_384742 533aa (23) *REMOVED INSERT yaervkvladtes**

**agldedacallrdhrqalsp.rielalralshrlqas.pdaarhfdsd..rqidrlhdlqsshwnvltdarfdglrmgldprwqiashaivlehlilgaiedaWP...ksilslgkarrrelrdlv............aalvraafvdteiavslrfnalrqqHQRQ.184**

**..........|..||..|........|..||..||...|.....|..|.......|..||..||..|..||....*..........|..|...||.........|.....|..|..|||.||........................||..||..|...|.........**

**Sulfurihydrogenibium azorense (Aquificales) gnl|TIGR_204536|contig:97:s_azorense**

**.........KLKPIFERYVD....DFIEKFYDFILRF.PQAKN.FLK.......NEEVIKRHREKVKKWFLDLF.SGNYDLEYFLKLRKIGETHVK........I...GLPTHYVNASMNFIRRYIID.....VIDKEVEDRKERNAYVASVGKLLDINLDIL.....**

**Thermodesulfovibrio yellowstonii gnl|TIGR_289376|contig:156:t_yellowstonii**

**..........LIELKPLMEK.RAEKVVGALYQWIIQTESAKRV.FKN.....ESLIKHVMRLVRTWFIALFSGK....YDNYFYDFLIRIGQRHER........V...GVDPHFMTRAINIIRNACVD......ILCEEIEKDREKYIISINKILD............**

**Thermomicrobium roseum (Chloroflexi) gnl|TIGR_309801|contig:2179:t_roseum**

**..LDEAERAELERLAPWAER.VADRIAQRFYDYQFSFEPTLRF.FQRY.....AQARGLPLEEVRRRLEQSQAAYFREIFQAARRGYDLEYFQRRLRIGARHNLI..DLPMKWYLGSYGRYLDFVRAE........LRRSFPFQPWRWWRVERALAKVFNLDQQAVVD**

**..........|..||..|........|..||..||...|.....|..|.......|..||..||..|..||.......................|..|...||.|.....|..|..|||.||........................||..||..|...|.........**

**Thermosynechococcus elongatus (Cyanobacteria) gi|22299532|ref|NP_682779 194aa (24)**

**RVQLTdedksllaeaapwgkeiapqmadtfydylgrde.emnailnat....egrihrlhqtfvdwfyemftgmDs.wg.kayaerrwkiglvhvrig......i....gpqhvvpamavvvnavrqk..........lreanksealsdalgkicmidlafieqay.164**

**..........|..||..|........|..||..||...|.....|..|.......|..||..||..|..||...............|..|...||.........|.....|..|..|||.||........................||..||..|...|.........**

**Thermus thermophilus (Deinococcus/Thermus) gi|46199407|YP_005074 203aa (32)**

**rtgfteahaallrelgevmvpiahevalafydylgrd.pelgallhae....pgrverlyrtfarwygelfsgvy....drayaerrrriglvharlg......i....gpramipamgivqelsleh......mrmalrghevysaveafeklvamevalieesyl.174**

**..........|..||..|........|..||..||...|.....|..|.......|..||..||..|..||...............|..|...||.........|.....|..|..|||.||........................||..||..|...|.........**

**Vibrio vulnificus (Gammaproteobacteria) gi|27366532|NP_762059 306aa (20)**

**HDLTEADLALIRKFGQIMVP.KLDEYVKHFYDWLRNT.PEYEQYF........GDAQKLQRVQDSQVRYWKTFFDARI..DSAYLERRDVGEIHARVGLP....L...PTYFAGMNISMVIFTKRMYD......TVHGIAYSSLVTAFTKLLHLDTTIVVDTYSRLI.163**

**..........|..||..|........|..||..||...|.....|..|.......|..||..||..|..||...............|..|...||.........|.....|..|..|||.||........................||..||..|...|.........**

**BACTERIAL SDgbs and FHbs**

**.........|..||..|................|..||..||...|......|..|..............|..||..||..|..||...................|..|...||......|......|..|..|||.||.....................||..||..|...|.............**

***Acinetobacter sp.ADP1 gi|50086221|YP_047731 254aa (1-141) (Gammaproteobacteria)**

**.mtpqqielvkstvpvlre.........hgvtltsyfynrmlnnhpelknv.fnld...........hqstgrqpralaaavlayaehi.......eqpeklakaverittkhvsln...i..qpeqyaivgenllhsisevlnvpf.......eselieawkqaylqladilievekekyhQLAK**

***Aquifex aeolicus gi|15605769|NP_213146 139aa (1-137) (Aquificales)**

**MLSEETIRVIKSTVPLLKE.........HGTEITARMYELLFSKYPKTKEL.F..............AGASEEQPKKLANAIIAYATYI..........DRLDNAISTIARSHVRRN...V..KPEHYPLVKECLLQAIEEVLNP.........GEEVLKAWEEAYDFLAKTLITLEKKLYSQP..**

***Bradyrhizobium japonicum gi|27377918|NP_769447 142aa (Alphaproteobacteria)**

**.MTPEQITLIQQSFAKVAP.........ISETAAVLFYDRLFEVAPSVRAM.F..............PEDMTEQRKKLMGMLAAVVGGL.......SNLDSILPAASALAKRHVA.....YGAKAEHYPVVGATLLWTLEKGLGEA.......WTPELATAWTDAYGVLSGYMISEAYGAQAQAAE**

***Bradyrhizobium japonicum gi|27379994|NP_771523 139aa (Alphaproteobacteria)**

**TLMTISPNPIEQSFERAAS.........RCADLTPLVYRRLFDEHPETRAM.F..................RSQGSELVKGSMLALT.I...........EAILDFAGTRSGHFRLIACEV.VSHDAYGTPRELFIAFFAVIRDTLRDLLGDAWSAEIAQAWDTLLTDIEAYVTVPA.........**

**.........|..||..|................|..||..||...|......|..|..............|..||..||..|..||....................|..|..||......|......|..|..|||.||.....................||..||..|...|.............**

***Campylobacter jejuni gi|15792891|NP_282714 140aa (Epsilonproteobacteria)**

**.MTKEQIQIIKDCVPILQK.........NGEDLTNEFYKIMFNDYPEVKPM.FNME...........KQISGEQPKALAMAILMAAKNI.......ENLENMRSFVDKVAITHVNLG...V..KEEHYPIVGACLLKAIKNLLNPDE.........ATLKAWEVAYGKIAKFYIDIEKKLYDK...**

***Campylobacter coli gi|32527608|AAP86201.1 142aa**

###### .mtqeqiqiikdcvpilqk.........ngedltkefykimfndypevkpm.fnme...........kqasgeqpkalamailmaakni.......enlenmrsfvdkvaithtkln...v..keehypivgacllkaikvvlyade.........ttlkaweeankaiaqfyidiekeiyakak.

***Campylobacter lari (Epsilonproteobacteria; Campylobacterales) 142 aa ZP_00369669.1 GI:57241724**

**.mtqeqiqiikdcvpilqk.........ngevltkefykimfeeypevkpm.fnme...........kqasgeqpkalamailmaaknv.......enlenmrsfvdkvaithtnln...v..keehypivgacllkaikvvlnade.........ttlkaweeaykaiakfyidiekeiyaknk.**

**.........|..||..|................|..||..||...|......|..|..............|..||..||..|..||....................|..|..||......|......|..|..|||.||.....................||..||..|...|.............**

***Chromobacterium violaceum gi|34498391| 148aa (Betaproteobacteria)**

**PLTQQQIRLVQESFAKVEP.........IADEAAKLFYDKLFEYAPDLRPL.F..............KKDMAAQRQMLMSTLKLAVKGL.......DDLEQLTPVLRKLAQRHVDYG...V..KPGDYTPVGNALLWTLKQGLGEAS......WTQELRAAWVDAFRLMATVMKQAAYPEIQpiqd**

***Clostridium perfringens gi|18310935|NP_562869 144aa (Firmicutes)**

**MLDQKTIDIIKSTVPVLKS.........NGLEITKTFYKNMFEQNPEVKPL.FNMN...........KQESEEQPKALAMAILAVAQNI.......DNLEAIKPVVNRIGVIHCNAK...V..QPEHYPIVGKHLLGAIKEVLGDGA.......TEDIINAWAKTYGVIAEVFINNEKEMYASR..**

***Desulfitobacterium hafniense gi|23119439|ZP_00102525 267aa (Firmicutes)**

**ALNEAAAAVVRATVPALAA.........HGHAVTTAMYRRLFRDE.RIAAL.FNHAN..........QGGDNAQAHALAGAILAYAQHI.......DDLPALSGALERIAQKHVGYH...I..LPEHYPFVAGALLGAIEETLGEAA.......TPQVLAAWGEAYWFLADLLQRREAELRGELAQ**

***Gloeobacter violaceus gi|37521141|NP_924518 142aa (Cyanobacteria)**

**TAMALQVKLLEQSFEGVKP.........NAHAFAASFYDNLFSDFPQTQAL.FA.............HSDMQAQQQKLLASLVLVVENL.......RQPQVLSTALQDLGNRHAGYG...I..VPEHYPMVGTSLLKTFETYLGDAW.......TPEVKQAWVDAYGAITGLMLTGAES.......**

**.........|..||..|................|..||..||...|......|..|..............|..||..||..|..||....................|..|..||......|......|..|..|||.||.....................||..||..|...|.............**

***Jannaschia sp. CCS1 (Alphaproteobacteria; Rhodobacterales)148aa ZP_00556752.1 GI:68183780**

**plaatqitpapitplqialvnatlprltcrpeqsamafrmhlaryaptltrsap.fspvallsdaiavidapdallarvaplahtlral...gmsprgymalhaalmdmvsghlagd...l.eveeaysdvigqilatmlaeahspraraiplva**

**.........|..||..|................|..||..||...|......|..|..............|..||..||..|..||....................|..|..||......|......|..|..|||.||.....................||..||..|...|.............**

***Nostoc punctiforme gi|23129187|ref|ZP_00111020.1| 126aa (Cyanobacteria)**

**....................................MYEIMFQNHPEVKEQ.FSMA...........AQADGSQPARLATAVYSYANQI.......DNLPALKSMVEKIAHRHVQTH...V..TPEQYPIVGESLLQAMKDVLGEAA.......TEEVMAAWTEAYQALSEVFIHREHDIYVGEDK**

***Novosphingobium aromaticivorans gi|23110290|ZP_00096448 148aa (Alphaproteobacteria)**

**IASEHAKTIVKATAPAIEK.........HGVDITTAMYKRLFQND.EVKAM.FDQA...........AQDSGEQPRRLAGAILAYAKNI.......DKLENLGSAVQRMVQRHVDTG...V..KAEHYPYVAEALLPAIRDVLGAEVA......TDEVLAAWGEAYWMLADILIAAEKQAYLEAVA**

***Photobacteriumm profundum gi|46915547|CAG22319.1 133 aa (Gammaproteobacteria)**

**...mdihevfndsytrcnr..........nphfmplfyerflekdetfrsm.f.............thvdmenqmkmikaslliimlaa........tseqaratvkkfgkrhgpdg...l...gikpldidlwfeclidtikecdpa.....ynqsveqawrqcfnegitimkaecks.......**

***Rhodopirellula baltica gi|32471656|ref|NP_864649 166aa (Planctomycetes)**

**DMTPEQVTLVKESWEKVKP.........ISEQAAELFYGRLFTLDPSLRSL.F..............KGDMSEQGKKLMSTITLAVTSL.......DRLETILPTVQALGRKHAVEYE..V..PDSSYATVGEALIWTLGQGLGDDF.......TEDVKEAWLLTYTTLSGAMLSGKSTAA.....**

***Pseudomonas aeruginosa gi|15599162|ref|NP_252656 130aa (Gammaproteobacteria)**

**...mnaadrvmqsygrcc..........astgffddfyrhflasspqirak.fA.............TTdmtaqkhllragimnlvmya.........rgmsdsklralgashsraald.i..rpelydlwldallmavaehdrdc........daetrdawrdvmgrgiaviksyy.........**

**.........|..||..|................|..||..||...|......|..|..............|..||..||..|..||....................|..|..||......|......|..|..|||.||.....................||..||..|...|.............**

***Rhodopseudomonas palustris gi|3993677|NP_949047 141aa (Alphaproteobacteria)**

**MMTPQAIALVQQSFARLTP.........ISDETATLFYERLFATAPELRPL.F..............HGDMRRQGKKLMSTLNVVVTGL.......TDLTTILPATGRLARLHVAYG...V..SAAHYTPFGAALLWTLERELGAEW.......TPELAAAWRDAYAMLSETMLAAAYGDTTPS..**

***Rhodopseudomonas palustris gi|22960924|ZP_00008530 146aa (Alphaproteobacteria)**

**TLSPADIHRVRTSFDLMWP.........RSTEMADQFYARLFEIAPDSRTL.FRSD........MTRMKDKFIQTLAVLVGSLDNLTGL.............YAVAGKLAVDHVRYG...V..RPDHYAPVGEALLWSLGQQLAGFW.......NDDVEQAWRRVYAVISARMIGAAYPDHRQG..**

***Shewanella amazonensis (Gammaproteobacteria) 141aa ZP_00584675.1 GI:68545124**

**sltndqislinqsfglvrp.........iaddaaalfyrnlfeidpslrsl.fks..............dlkiqgrklmamldaavkgl.......nnpdklvpvlqdlarrhvqyg...v..kthhfspvgnallytlaeglgdkf.......tkevkdawiavlhlvadvmkaemkkqgca...**

**.........|..||..|................|..||..||...|......|..|..............|..||..||..|..||...................|..|...||......|......|..|..|||.||.....................||..||..|...|.............**

***Silicibacter gi|52009457|ZP_00336820 143aa (Alphaproteobacteria)**

**ilrqievqlikvsfnrvfa.........qkaalaekfyhhlflelpdaevm.f..............trdfshqtemfarvlttgmqsl.......grdremmvlvddllqrhkhlg...l.tldqmytaqralhlafcevmqael........taaevsawdnaigrlcralaagieppaa....**

***Thermobifida fusca gi|48835900|ZP_00292898 177aa (Actinobacteria)**

**rpdlrtvslvqalcadlld.........qpevladrfyhhlfrllpscrdl.f..............Pedmgeqhvrmarvlvevvnhl.......DEPektwdtlrklgeyhyvrwg..l..gleeyrcvghalieaardislew........apsvgsawvtvyewivsamlsgaaeaaqARSG**

***Vibrio parahaemolyticus gi|28900962|NP_800617 142aa (Gammaproteobacteria)**

**...mnvievfndsyercvl..........sehfferfyakfwskgksfrqk.f.............egvdmqkqvrmlkgsivffmmtd........tssdarkmmekygkkhgignig.i..kpedidiwfeslleavqecdyey........nsdvekawracfksgleimkrecgtppsksdt**

***Vitreoscilla stercoraria gi|70622|pir||GGZLB[70622] 146aa (Betaproteobacteria)**

**MLDQQTINIIKATVPVLKE.........HGVTITTTFYKNLFAKHPEVRPL.FDMG...........RQESLEQPKALAMTVLAAAQNI.......ENLPAILPAVKKIAVKHCQAG...V..AAAHYPIVGQELLGAIKEVLGDAA.......TDDILDAWGKAYGVIADVFIQVEADLYAQAVE**

**.........|..||..|................|..||..||...|......|..|..............|..||..||..|..||...................|..|...||......|......|..|..|||.||.....................||..||..|...|.............**

**BACTERIAL FHbs**

**Anaeromyxobacter dehalogenans (Deltaproteobacteria; Myxococcales) ZP_00399684.1 GI:66855611 379aa**

**kryvhfdaaderalralhg.....aaapqldriaqvfydrilghegartal.vgg............esrvghlkvtliawmdtlltgp........wdeaywehrtrigrvhvr.....iglpqhymfgamnvirtelmrvswerf.nadppelervrnalakildlelaimlhtyrddllaqqa**

**.........|..||..|................|..||..||...|......|..|..............|..||..||..|..||...................|..|...||......|......|..|..|||.||.....................||..||..|...|.............**

**Arthrobacter sp. FB24 (Actinobacteria; Actinomycetales) ZP_00413395.1 GI:66965830 386aa**

**mlsdksfpvieatlplvgs.........rigeitpkfyarlfaahpelldglfsr...........snqrngnqqqalagsiaafathl.....vnnpgtlpetvlariahrhaslg...i..tepqyqvvyehlfaaiaedlaevit.......peiaeawtevywlmadaliklekglyaaqan**

**.........|..||..|................|..||..||...|......|..|..............|..||..||..|..||...................|..|...||......|......|..|..|||.||.....................||..||..|...|.............**

**Azotobacter vinelandii gi|23104720|ZP_00091182 393aa (1-144) (Gammaproteobacteria)**

**MLTEQQRAIVKATVPLLET.........GGEALTSHFYRIMLAEHPEVRPL.FNQA...........HQASGEQPRALANAVLMYARHI.......DRLEALGPLVAQIVNKHVSLQ...V..LPEHYPIVGACLLRAIREVLGAETA......TDAVLDAWGAAYGMLAELLIGAEEAAYAGAAV**

**.........|..||..|................|..||..||...|......|..|..............|..||..||..|..||...................|..|...||......|......|..|..|||.||.....................||..||..|...|.............**

**Bacillus anthracis gi|30261545|NP_843922 403aa (Firmicutes; Bacilli)**

**MLSEKTIEIVKSTVPLLQE.........KGVEITTRFYEILFSEHPELLNI.FNHT...........NQKKGRQQQALANAVYAAATYI.......DNLEAIIPVVKQIGHKHRSLG...I..KAEHYPIVGTCLLRAIKEVAGA.........PDEVLNAWGEAYGVIADAFISIEAEMYEEAAH**

**Bacillus cereus gi|30019597|NP_831228 402aa (Firmicutes; Bacilli)**

**MLSAKTIEIVKSTVPLLQE.........KGVEITTRFYQILFSEHPELLNI.FNHT...........NQKKGRQQQALANAVYAAATYI.......DNLEAIIPVVKQIGHKHRSLG...I..KAEHYPIVGTCLLRAIKEVAGA.........PDEVLNAWGEAYGVIADAFISIEAEMYEEAAH**

**.........|..||..|................|..||..||...|......|..|..............|..||..||..|..||...................|..|...||......|......|..|..|||.||.....................||..||..|...|.............**

**Bacillus clausii (Firmicutes) 411aa YP_173853.1 GI:56962130**

**mlkeeskrlvkatvpilaa.........hgetitkhfykrmfhhhpellnv.fnr...........tnqkigrqpqalansiyaaaqhi.......dnleailpvvkriahkhrsln...i..kpeqypivgenllaamkevlgeAA.......sddvieawreayeiiagvfievekqmydqagk**

**.........|..||..|................|..||..||...|......|..|..............|..||..||..|..||...................|..|...||......|......|..|..|||.||.....................||..||..|...|.............**

**Bacillus halodurans (Firmicutes) gi|15613621|NP_241924 411aa (Firmicutes; Bacilli)**

**TLSQETKQIVKATVPILAE.........HGEAITKHFYKRMFSHHPELLNI.FNQT...........HQKQGRQPQALANSIYAAAEHI.......DNLEAILPVVSRIAHKHRSLN...I..KPEQYPIVGENLLAAMREVLGDAA.......SDDVLEAWREAYELIADVFIQVEKKMYEEASQ**

**.........|..||..|................|..||..||...|......|..|..............|..||..||..|..||....................|..|..||......|......|..|..|||.||.....................||..||..|...|.............**

**Bacillus licheniformis (Firmicutes) 404aa YP_078209.1 GI:52079418**

**mlsektmqivkstapvlke.........kgteittcfykrmfdahpelkni.fn.m..........srqqtggqpkalaftvlqaaeni.......drledllpvvkqighkhkslh...v..kpehypivgqhlleaieivlgeaa.......tedilqawaeayeeiarvfievekqmyeedkk**

**.........|..||..|................|..||..||...|......|..|..............|..||..||..|..||....................|..|..||......|......|..|..|||.||.....................||..||..|...|.............**

**Bacillus subtilis gi|16078369|NP_389187 399aa (Firmicutes; Bacilli)**

**MLDNKTIEIIKSTVPVLQQ.........HGETITGRFYDRMFQDHPELLNI.FNQT...........NQKKKTQRTALANAVIAAAANI.......DQLGNIIPVVKQIGHKHRSIG...I..KPEHYPIVGKYLLIAIKDVLGDAA.......TPDIMQAWEKAYGVIADAFIGIEKDMYEQAEE**

**Bacillus thuringiensis gi|49481712|YP_035665 402aa (Firmicutes; Bacilli)**

**mlsektieivkstvpllqe.........kgveittrfyeilfsehpellni.fnht...........nqkkgrqqqalanavyaaatyi.......dnleaiipvvkqighkhrslg...i..kaehypivgtcllraikevaga.........pdevlnawgeaygviadafisieaemyeEAAH**

**.........|..||..|................|..||..||...|......|..|..............|..||..||..|..||....................|..|..||......|......|..|..|||.||.....................||..||..|...|.............**

**Bordetella pertussis gi|33593213|ref|NP_880857 402aa (Betaproteobacteria)**

**MLSPEVRALVKATAPVLKE.........HGEALTRHFYTRMLGGNPELRQL.FNQG...........HQQSGQQQQALAAAVAAYAEHI.......DDPSVLLPVVERIAHKHVS.....LGVRAEHYAIVGKHLLASIREVLGEAA.......TDELIDAWAAAYGQLADLLIGRERALYAAAAS**

**Burkholderia ambifaria (Betaproteobacteria; Burkholderiales) 402aa EAO46513.1 GI:72610566**

**hitadqmarvkatapvlae.........hgatitkhfyqrmfarhpelknl.fnqt...........hqktgsqpetlakavyayaani.......dnlgalggavsrishkhasln...i..rpehypivgenllasivevlgdav.......daetleawrvaygqlaqimigaeadlyanaaw**

**.........|..||..|................|..||..||...|......|..|..............|..||..||..|..||....................|..|..||......|......|..|..|||.||.....................||..||..|...|.............**

**Burkholderia cenocepacia gi|46316131|ZP_00216711 394aa (Betaproteobacteria)**

**......marvkatapvlav.........hgatitkhfyqrmfarhpelknl.fnqt...........hqktgsqpetlakavyayaani.......dnlgalggavtkiahkhasln...i..rpehypivgenllasivevlgdav.......daetleawrvaygqlaqiligveadlyaGAAW**

**Burkholderia cenocepacia gi|46324061|ZP_00224423 403aa (Betaproteobacteria)**

**mltqktkdivkatapvlaq.........hgydiitcfyrklsdahpelrna.fnma...........hqeqgqqqqalaravyayaeni.......edpgslaavlkniankhaslg...v..rpehypivgenllaaiketlgdaa.......tddivsawaqaygnladllmgmestlyeRSSE**

**Burkholderia fungorum gi|22983511|ZP_00028694 414aa (Betaproteobacteria)**

**MLSAEHRAIVKATVPLLES.........GGEALTTHFYRILIGEHPEVRPM.FNLA...........NQASGAQPRALANSVLMYARHI.......DQLEQLGGLVSQIINKHVALN...I..LPEHYPIVGQCLLRAIREVLGAEIA......TDAVIAAWAAAYRQLADLLIGLEEKIYAEREA**

**.........|..||..|................|..||..||...|......|..|..............|..||..||..|..||....................|..|..||......|......|..|..|||.||.....................||..||..|...|.............**

**Burkholderia mallei (Betaproteobacteria; Burkholderiales) 402aa ZP_00451964.1 GI:67654548**

**qmtaeqmarvkatapvlae.........hgatitkhfyqrmfgrhpelknv.fnqt...........hqktgsqpetlakavyayaani.......dnlgalggavsriahkhasln...i..rpehypivgenllasivevlgdavd.......adtleawriaygqlaailigaeanlyenaaw**

**.........|..||..|................|..||..||...|......|..|..............|..||..||..|..||....................|..|..||......|......|..|..|||.||.....................||..||..|...|.............**

**Burkholderia pseudomallei (Betaproteobacteria; Burkholderiales)402 aa CAH36850.1 GI:52210862**

**qmtaeqmarvkatapvlae.........hgatitkhfyqrmfgrhpelknv.fnqt...........hqktgsqpetlakavyayaani.......dnlgalggavsriahkhasln...i..rpehypivgenllasivevlgdavd.......adtleawriaygqlaailigaeanlyenaaw**

**.........|..||..|................|..||..||...|......|..|..............|..||..||..|..||....................|..|..||......|......|..|..|||.||.....................||..||..|...|.............**

**Burkhollderia vietnamiensis 402aa ZP_00425530.1 GI:67547629**

**ditadqmarvkatapvlav.........hgatitkhfyqrmftrhpelknl.fnq...........thqktgsqpetlakavyayaani.......dnlgalggavshiahkhasln...i..rpehypivgenllasivevlgdavd.......aqtleawrvaygqlaqiligaeadlyaGAAW**

**.........|..||..|................|..||..||...|......|..|..............|..||..||..|..||....................|..|..||......|......|..|..|||.||.....................||..||..|...|.............**

**Burkholderia vietnamiensis 403aa EAM32431.1 GI:67535704**

**mltqktkdivkatapvlaqh.........gydiitcfyrklsdahpelrna.fnma...........hqeqgqqqqalaravyayaeni.......edpgslaavlkniankhaslg...v..rpehypivgenllaaiketlgdaat.......ddivsawaqaygnladllmgmestlyersse**

**.........|..||..|................|..||..||...|......|..|..............|..||..||..|..||....................|..|..||......|......|..|..|||.||.....................||..||..|...|.............**

**Chromobacterium violaceum gi|34498943|NP_903158 404aa (Betaproteobacteria)**

**mlseqtrslvkatvpvlQQ.........hgvaltshfyrrmfehnpelknv.fnqg...........hqhsgqqqqalamavlayarhi.......ddpspllpvltrvahkhvslg...i..raehypivgkhllasirellgeaa.......gddliqawaeayglladtligiengmygDATS**

**Chromohalobacter salexigens (Gammaproteobacteria) 393aaZP_00473490.1 GI:67676744**

**mltsaqesviaattpvvae.........hieaiaqrfyplmfarypevktl.fn.a..........thqhtggqpralagavvgyvqlr.......htpqrldevlgiivdkhvslg...i..rpehypivgeclmaaigevlgdav.......tdevadawgalyeelaglliekearryrdfag**

**.........|..||..|................|..||..||...|......|..|..............|..||..||..|..||....................|..|..||......|......|..|..|||.||.....................||..||..|...|.............**

Colwellia psychoerythrae (Gammaproteobacteria) 398aa YP_267597.1 GI:71278489 FHb

**mltdkhidiikstipllen.........agpaltghfyqrmfshnpelqdi.fnma...........nqhsgrqqvalfeaiaayakni.......enlaalttaveriaqkhtsfn...i..kadhyaivghhlietlrelasdvf.......tadveeawtvayqflaqifinreaelyqqraE**

**.........|..||..|................|..||..||...|......|..|..............|..||..||..|..||....................|..|..||......|......|..|..|||.||.....................||..||..|...|.............**

**Corynebacterium efficiens gi|25029214|NP_739268 404aa (Actinobacteria)**

**RLSEAHAEMIKATLPLVGS.........KINDITPVFYNRMFTAHPELLRSTFNRA...........NQKQGDQQKALAASIATFATML.....VDPEAPDPVDMLARIGHKHVSLG...I..TADQYNIVHEHLFGAIVEVLGADVV......TPPVAEAWDAVYWLMANVLIDLESDLYASNDL**

**Corynebacterium glutamicum gi|19554028|NP_602030 386aa (Actinobacteria)**

**ALSAEHAEVIKATLPLVGG.........KINEITPVFYNKMFAAHPELIANTFNRG...........NQKQGDQQKALAASIATFATML.....VTPDAPDPVQLLSRIGHKHVSLG...I..TADQYDIVHEHLFAAIVEVLGAETV......TAPVAEAWDAVYWIMANVLIGFENNLYASNDL**

**Corynebacterium jeikeium (Actinobacteria) 133aa CAI36697.1 GI:68263209**

**....mvggmtssfydavge........etfrtivhefyrqvrtdd.ilgpm.y.............peddlegaedrlrwflvqywggp........aefnarrghprlrmrhaqfr...i..dtaardrwlelmgnamatidkeqlp......dehraamwehmervahmlvntpdqf.......**

**.........|..||..|................|..||..||...|......|..|..............|..||..||..|..||....................|..|..||......|......|..|..|||.||.....................||..||..|...|.............**

**Deinococcus radiodurans gi|15807907|NP_285566 403aa (Thermus/Deinococcus)**

**MLTPEQKAIVKATVPALEA.........HGETITRTFYASMFAAHPELLNI.FNPA...........NQQTGKQARSLAASVLAYAAHI.......DHPEALGGMVGRIAHKHVSLE...V..LPEHYPIVGQYLLGAIAGVLGDAA.......KPEILDAWAAAYGELADLMIGIEKGMYDAGAG**

**Erwinia chrysanthemi gi|628646|pir||S44277 395aa (Gammaproteobacteria)**

**MLDQQTIATIKSTIPLLAE.........TGPALTAHFYQRMFHHNPELKDI.FNMS...........NQRNGDQREALFNAICAYATHI.......ENLPALLPAVERIAQKHASFN...I..QPEQYQIVGTHLLATLEEMFQP.........GQAVLDAWGKRYGVLANVFIQRESDIYQQSAG**

**Escherischia coli gi|16130477|NP_417047 396aa (Gammaproteobacteria)**

**MLDAQTIATVKATIPLLVE.........TGPKLTAHFYDRMFTHNPELKEI.FNMS...........NQRNGDQREALFNAIAAYASNI.......ENLPALLPAVEKIAQKHTSFQ...I..KPEQYNIVGEHLLATLDEMFSP.........GQEVLDAWGKAYGVLANVFINREAEIYNENAS**

**Exiguobacterium gi|46114160|ZP_00184406.2 384aa (Firmicutes)**

**Mltpsaitiikstvpvlae.........hghsitrvfyqrlfenhpemkhi.fnqs...........nqkndrqsqalatavyaaaayi.......dqletlkptllpvlhkhrslq...i..kpymydivgteligaiqdvlkdaa.......tpdiidawtagygeiaklfisleaelyqQDEQ**

**Gluconobacter oxydans (Alphaproteobacteria)393aa YP_191291.1 GI:58039327**

**mstsadrsltpetiaiika..tipaleahgvtitsemyrrlladpaiaem..fdp...........thqstgtqpralamavlayakni.......dnlgvmgsaveriaqkhasle...i..qpehyphvatallgaissvlgeaa.......tpeildawgkaywaladiligrehqlydssah**

**.........|..||..|................|..||..||...|......|..|..............|..||..||..|..||....................|..|..||......|......|..|..|||.||.....................||..||..|...|.............**

**Magnetospirillum magnetotacticum gi|23006958|ZP_00049038 403aa (Alphaproteobacteria)**

**PLSARTIAVVKATIPALET.........HGLTITRRMYERLFQNT.DIRDL.FNQSH..........HGETGSQPKALALAVLAYARNI.......DNLGVLTGAVERIQK.HVALN...I..LPEHYSFVADALIGAIQDVLGEAA.......TPEIVEAWGEAYWFLAELLIGREATIYRDLAS**

**Methylobacillus flagellatus gi|45521753|ZP_00173271 371aa (Betaproteobacteria)**

**........................................mlsahpelknl.fnmg...........nqangiqqqslaaavfayaani.......dnpaalapvvkrivhkhaavg...i..kpehypivgqyligaikqvlgeaa.......tdellaawgeaywslanllieeekslyaSTAS**

**Mycobacterium tuberculosis gi|15607526|NP_214899 390aa (Alphaproteobacteria)**

**..MGLEDRDALRVLQNAFKL........DDPELVRRFYAHWFALDASVRDL.F..............PPDMGAQRAAFGQALHWVYGEL.....VAQRAEEPVAFLAQLGRDHRKYG...V..LPTQYDTLRRALYTTLRDYLG...........HPSRGAWTDAVDEAAGQSLNLIIGVMSGAAD**

**Oceanobacillus iheyensis gi|23097746|NP_691212 406aa (Firmicutes)**

**LLDKKTTEIIKATVPVLKE.........HGEAITKHFYKILLENNPELKNV.FNQT...........NQRKGAQSKALANTVYAAAANI.......EKLEEILPHVKQIAHKHVSLN...I..KPEQYPIVGKYLLIAIKEVLGDAA.......TDEIIEAWEKAYFVIADIFISVEKEMYNEKKN**

**Paracoccus denitrificans (Alphaproteobacteria) 402aa ZP_00629436.1 GI:69934358**

**plsaqtialvkatvpaiea.........hgldivremyarmfqnpeirdl..fnq..........shqdgaeaqpraltgailayanni.......dnvtalvpaveriaqkhvglq...i..lpehyphvaeallgairavlgda.......atdevleawgeaywflaniliarerriydeqqa**

**.........|..||..|................|..||..||...|......|..|..............|..||..||..|..||....................|..|..||......|......|..|..|||.||.....................||..||..|...|.............**

**Photobacterium profundum gi|46914864|CAG21641.1 394aa (Gammaproteobacteria)**

**misqqtidivkatapvvae.........tgpkltahfyermfnhnpelkdi.fnms...........nqrngdqrealfnaicayanni.......dnlaallpavekiahkhtsfm...i..taeqyqivgghllatidellspg.........QevldawgeaygvlanvfitreeaiyqENEE**

**Photorhabdis luminescens gi|37527172|NP_930516.1 396aa (Gammaproteobacteria)**

**MLDNQTIATVKSTIPLLSA.........TGPKLTAHFYERMFKHNPELKNI.FNMS...........HQLNGDQREALFNAICAYAANI.......DNLKVLLPAVEKIAHKHASLN...I..QPEHYQIVGTHLLATLNEMFQP.........GNEILDAWGKAYGVLADIFINREEQIYHSGEL**

**Polaromonas sp. JS666 (Betaproteobacteria) 393aa ZP_00508092.1 GI:67909697**

**mltaqqraivkstvplles.........ggealtthfyqilltehpevrpf.fnqa...........hqangdqpralangvlmyarhi.......dkleqlgglvaqiinkhvslq...i..epahypivgacllrairevlga......eiatgevlaawgaaygqladiligaeekiyaetaa**

**.........|..||..|................|..||..||...|......|..|..............|..||..||..|..||....................|..|..||......|......|..|..|||.||.....................||..||..|...|.............**

**Pseudomonas aeruginosa gi|15597860|NP_251354 393aa (Gammaproteobacteria)**

**MLSNAQRALIKATVPLLET.........GGEALITHFYRTMLGEYPEVRPL.FNQA...........HQASGDQPRALANGVLMYARHI.......DQLQELGPLVAKVVNKHVSLQ...V..LPEHYPIVGTCLLRAIREVLGEQIA......TDEVLEAWGAAYQQLADLLIEAEESVYAASAQ**

**.........|..||..|................|..||..||...|......|..|..............|..||..||..|..||....................|..|..||......|......|..|..|||.||.....................||..||..|...|.............**

**Pseudomonas fluorescens gi|23058447|ZP_00083507 393aa (Gammaproteobacteria)**

**MLSVQDRAIVKSTVPLLES.........GGEALITHFYRMMLSEYPEVRPL.FNQA...........HQASGDQPRALANGVLMYARHI.......DQLDQLGDLVAKIINKHVALQ...I..LPEHYPIVGTCLLRAISEVLGEEIA......TPEVMSAWGAAYGQLAEILIGAETAIYDQKEQ**

**Pseudomonas putida gi|26987544|NP_742969 392aa (Gammaproteobacteria)**

**MLNAEQRAIIKATVPLLES.........GGEALTTHFYKMMLSEYPEVRPL.FNQA...........HQASGDQPRALANGVLMYARHI.......DQLEQLGGLVGQIINKHVALQ...I..LPEHYPIVGSCLLRAIEEVLGKDIA......TPAVIDAWGAAYGQLADILIGAEENLYKEKEE**

**Pseudomonas syringae (Gammaproteobacteria) 395aa YP_273509.1 GI:71736900**

**mlstahrdiikatvpilet.........ggealtthfyrimlndypqvrpl.fnqa...........nqangaqpralanavlmyarnv.......drlenlgslaaqivnkhvalq...i..lpehypivgacllrairevlgaeia......tdavidawaaayqqladmlisaerdvyesiaa**

**Psychrobacter cryohalolentis (Gammaproteobacteria) 399aa ZP_00654949.1 GI:71364357**

**maslqtlaivkatvpvlee.........hgtaittvfyknmfhehpelldi.fnet...........nqklgrqqtalamtvlaaakhl.......enlatllpqvtqishkhralq...i..lpehypivgkhllaaikevlgdaat.......ddiltawaeaydeiasifiqiehgmyeqamw**

**.........|..||..|................|..||..||...|......|..|..............|..||..||..|..||....................|..|..||......|......|..|..|||.||.....................||..||..|...|.............**

**Ralstonia (Alcaligenes, Wautersia) eutropha gi|729747|P39662 403aa (Betaproteobacteria)**

**mltqktkdivkatapvlaeh.........gydiikcfyqrmfeahpelknv.fnma...........hqeqgqqqqalaravyayaeni.......edpnslmavlkniankhaslg...v..kpeqypivgehllaaikevlgnaa.......tddiisawaqaygnladvlmgmeselYERSAE**

**Ralstonia metallidurans gi|48771323|ZP_00275665 406aa (Betaproteobacteria)**

**mlsaasrpyidasvpvlre.........hglaitthfyremfaarpelkri.fnmg...........nqangsqqqslasavfayaani.......dradvlapvverivhkhvavg...l..tpahypivgkylleaiaavlgdaa.......tppllaawdeaywllageliaaearlyeRHGV**

**Ralstonia solanacearum gi|17548115|NP_521517 401aa (Betaproteobacteria)**

**MLSEQSKPLIDASVPVLRE.........HGLTITQTFYRNMFASHPELTNL.FNMG...........NQANGSQQQSLASAVFAYAANH.......GNNAALAPVVGRIVHKHAAVG...I..RPSHYPIVGRHLLGAIAEVLGDAA.......TPELLAAWDEAYWLLAAELIAAEARLYAHTQS**

**.........|..||..|................|..||..||...|......|..|..............|..||..||..|..||....................|..|..||......|......|..|..|||.||.....................||..||..|...|.............**

**Rhodopirellula baltica gi|32477410|NP_870404 417aa (Planctomycetes)**

**MLSEKTIRIVKEITPLVAA.........NAETITRRFYERMFEANPEVKAF.FNQA...........HQHSGGQQKALAGAICAYFTHI.......DNPAVLMPAVELIAQKHVSLG...I..KPEHYPIVGSNLLAAIGDVMGDAA.......TPEIVEAVSEAYGFLADIFIGREGAIYEEQAS**

**Salmonella typhimurium gi|16765876|NP_461491 396aa (Gammaproteobacteria)**

**MLDAQTIATVKATIPLLVE.........TGPKLTAHFYDRMFTHNPELKEI.FNMS...........NQRNGDQREALFNAIAAYASNI.......ENLPALLPAVEKIAQKHTSFQ...I..KPEQYNIVGTHLLATLDEMFNP.........GQEVLDAWGKAYGVLANVFIHREAEIYHENAS**

**Shewanella baltica (Gammaproteobacteria)397aa ZP_00584368.1 GI:68544761 FHb**

**mldsrtiqvikstiplles.........agpaltthfyqrmfkhnpelkdv.fnla..........hqhsggqpvalfnavaayaknie........nlavlsstveriaqkhtgfl...i..kpeqyaivgshllatlkelggdav.......teevleawaqaygflanifiqreaqiyqahae**

**Shewanella frigidimarina (Gammaproteobacteria) 393aa ZP_00638128.1 GI:69950229**

**mldtatvqiikatvpalql.........yanditshfypllfaqhpevlpy.fnq...........tnqgkgtqpkalanaviaygani.......dalgnlseavskivqkhvalg...i..lpeqydavgscliqaikavlgdaa.......tdevidawtkaygqlaniliaaeesiytakeq**

**.........|..||..|................|..||..||...|......|..|..............|..||..||..|..||....................|..|..||......|......|..|..|||.||.....................||..||..|...|.............**

**Sinorhizobium meliloti gi|16263102|NP_435895 403aa (Alphaproteobacteria)**

**MLTQKTKDIVKATAPVLAQ.........HGYAIIQHFYKRMFQAHPELKNI.FNMA...........HQERGEQQQALARAVYAYAANI.......ENPESLSAVLKDIAHKHASLG...V..RPEQYPIVGEHLLASIKEVLGDAA.......TDEIISAWAQAYGNLADILAGMESELYERSEE**

**.........|..||..|................|..||..||...|......|..|..............|..||..||..|..||....................|..|..||......|......|..|..|||.||.....................||..||..|...|.............**

**Staphylococcus aureus gi|15923230|NP_370764 381aa (Firmicutes)**

**MLTEQEKDIIKQTVPLLKE..........GTEITSIFYPKMFKAHPELLNM.FNQT...........NQKRGMQSSALAQAVMAAAVNI.......DNLSVIKPVIMPVAYKHCALQ...V..YAEHYPIVGENLLKAIQDVTGLEE.......HDPVIQAWAKAYGVIADVFIQIEKEIYDQMMW**

**Staphylococcus epidermidis gi|27467358|NP_763995 381aa (Firmicutes)**

**MLTEKEQDIIKQTVPLLQD.........KGTEITSIFYPKMFEANPELLNM.FNQT...........NQKKGMQSAALAQAVLAAAMNI.......NNLGAIKPAIMPVAHKHCALQ...V..YPEHYPIVGENLLAAIQDVTGLES.......DDPVIQTWAKAYGEIADVFIKLEQEIYNHMLW**

**Staphylococcus haemolyticus (Firmicutes) 381aa YP_253814.1 GI:70726900 FHb**

**mlnekekgiiketvpvlqek.........gteitsyfynrmfnqhpelknm.fnq...........tnqqkgfqstalaqsvlaaavni.......dnlgnimpvvkeiaykhcalevppa.....gydivgenlieaikavvgled.......dhpiiqtwkkayqeiadvfiqvekdiyasmlw**

**Staphylococcus saprophyticus (Firmicutes) 381aa YP_301818.1 GI:73663037**

**mlnekekaiiketvpvlqe.........rgveitsffynrmftqhpelknm.fnq...........tnqkkglqstalaqsvlaaamni.......ddltrilpvvkeiafkhcalqvppa.....gydivgenllaaiqnvlklsd.......ddaildtwakaygeiakvfievekdiyktmaw**

**.........|..||..|................|..||..||...|......|..|..............|..||..||..|..||....................|..|..||......|......|..|..|||.||.....................||..||..|...|.............**

**Streptomyces avermitilis gi|29832496|NP_827130 400aa (Actinobacteria)**

**MLSEQSAATVRATLPAVGA.........AVGEITARFYDRLFAARPELLRDLFNRG...........NQAAGTQRQALAGSIAAFATYL.....VEHPDERPDAMLDRIAHKHASLG...I..APGQYAVVHEHLFAAIAEVLGDAV.......TPEVAAAWDEVYWLMANALIAIERRLYAQHEN**

**Streptomyces avermitilis gi|29830819|NP_825453 565aa (Actinobacteria)**

**PEASPDAVLIRRTMAEVGP.........VAGKVTSYFYALLFVRHPDLRSL.F..............PAAMDTQRDRLLKALLTAAEHI.......DNTEVLVAYLQNLGRGHRK.....YGTRPEHYPAVGECLIGALSRYASGIW.......DEETEAAWVRAYTTISQVMIDAAAADELRAPA**

**Streptomyces coelicolor gi|21218667|NP_624446 435aa (Actinobacteria)**

**SVTDEDVALVRASLTVVTP.........HVSELAAHFYSILFSRYPQVRDL.F..............PAELDVQRERLVRALLRIVELV.......DDPDNLVAFCSRLGRGHRK.....FGTQSGHYPAVGECLLQALSHFAGPAW.......HPALATAWQRAYTAAADVMVRAAEEDARSRPA**

**Streptomyces coelicolor gi|21225376|NP_631155 403aa (Actinobacteria)**

**MLSSQSAQIVRDTLPTVGA.........SLGTITDLFYRRMFEERPELLRDLFNRA...........NQASGVQREALAGAVAAFATAL.....VKHPDERPDAVLGRIANKHASLG...I..TSDQYTLVGRHLLAAVAEVLGDAV.......TPAVAAAWDEVYWLMANALIAMEARLYARSDV**

**.........|..||..|................|..||..||...|......|..|..............|..||..||..|..||....................|..|..||......|......|..|..|||.||.....................||..||..|...|.............**

**Streptomyces coelicolor gi|21225698|NP_631477 398aa (Actinobacteria)**

**MLSEQSVPVVRATLPAVGA.........AIGDIAALFYRKLFDAHPELLRDLFNRG...........NQANGEQQRALAGSVAAFAGLL.....LENPDERADVLLSRISHKHASLG...I..TPDQYTIVHRHLMAAVVDVLGDAV.......TPEVARAWDEVYWLMANALIALEARLYTEKGV**

**Thermobifida fusca gi|4883571|ZP_0002715 394aa (Actinobacteria)**

**MLSTQSADIVRATLPVVGA.........HLDEITSRFYSTMFSERPELLDGLFNRG...........NQANGEQRRALAGSIAGFASAL.....LANPDERPDALLARIAHKHAAVA...V..TDDQYVIVHKYLFGAIADVLGDAA.......TPEVIAAWDEVYWLMGGALIALEARLYAEKNA**

**Vibrio cholerae gi|15600953|NP_232583 394aa (Gammaproteobacteria)**

**MLTQEHINIIKSTIPLLES.........AGPALTQHFYQRMFSHNPELKHI.FNMT...........HQKTGRQSVALFEAIAAYAKHI.......DNLAALTSAVERIAHKHTSFN...I..QPEHYQIVGHHLLETLRELAPDAF.......TQPVEEAWTAAYFFLAQVFIDREGALYLERKQ**

**Vibrio parahaemolyticus gi|28899583|NP_799188 394aa (Gammaproteobacteria)**

**MLSNQTIEIVKATAPLIAET.........GPKLTAHFYDRMFTHNPELKDI.FNMS...........NQRNGDQREALFNAICAYAANI.......ENLPALLGAVEKIAHKHTSFL...I..TKDQYQIVGKHLIATIDELFNP.........GQEVLDAWAEAYGVLANVFIQREEQIYQANAS**

**Vibrio vulnificus gi|27364701|NP_760229 394aa (Gammaproteobacteria)**

**MLSENTINIVKSTAPLLAET.........GPKLTAHFYQRMFEHNPELKDI.FNMS...........NQRNGDQREALFNAICAYASNI.......DNLPALLGAVEKIAHKHSSFL...I..TADQYQIVGGHLLATIDELFSP.........GQAVLDAWAEAYGVLANVFIQREEQIYQDNQS**

**Xylella fastidiosa gi|15836658|NP_297346 397aa (Gammaproteobacteria)**

**SFSPHTITLIKSTVPLLAE.........HGTTIIEAMYHRLFED.PQIEAL.FNQA...........NQKNGTQIHALAGAILAYARNI.......DNPGVLASAIERIAQKHVGYA...I..HPEHYPHVATALLGAIKKVLGDVA.......TSEVLEAWGEAYWFIANLLKDREAVIREGIMT**

**Yersinia pestis gi|16123099|NP_406412 396aa (Gammaproteobacteria)**

**MLDTQTIAIVKSTIPLLAA.........TGPKLTAHFYERMFKHHPELKNI.FNMS...........NQSSGDQREALFNAICAYATNI.......ENLAALLPTVERIAQKHTSLN...I..QPEHYPIVGEHLIATLDELFSP.........GQAVLDAWAKAYGVLADVFIQRESQIYQQSET**

**.........|..||..|................|..||..||...|......|..|..............|..||..||..|..||...................|..|...||......|......|..|..|||.||.....................||..||..|...|.............**

**ARCHAEAL 2/2Hbs**

**Haloarcula marismortui (Euryarcheota) gi|55379358|YP_137208 120aa**

**>Halmar**

**.......masqsiferigg.....rdavdavvsdfydrvrdd.pllepy...feet......dmdqlrshqtqfistvaggp....vdydgddmqtahegmg.....i....tedafasvathleaalrangvp....dddveailtevaamkddivea......**

**.........|..||..|..............|..||..||...|........|..|.......|..||..||..|..||...........|..|...||........|........|..|..|||.||...............||..||..|...|.........**

**Haloferax volcanii (Archaea; Euryarchaeota) gnl|TIGR_309800|contig:441:h_volcanii**

**.....RRMPAQSLYARIGG.....REAVEAVVSDFYDRVLADDSVAHY....FDDI......DMVEQRAHQVKLISAVAGGP....VEYDGADMRAAHDHLD.....L....SGEDFDAIAEHLAAALDEN....GVAPEDADAILDEVAALRAPI**

**.........|..||..|..............|..||..||...|........|..|.......|..||..||..|..||...........|..|...||........|........|..|..|||.||...............||..||..|...|.........**

**BACTERIAL 2/2 Hbs**

**2/2Hb1s**

**Anaeromyxobacter dehalogenans (Deltaproteobacteria; Myxococcales) 351aa ZP_00401147.1 GI:66857090 (210-) C-TERMINAL 2/2HB1 DOMAIN**

**rcrqrsvamaaslyerlgg......eekiaqivndvldlhlknpiigtr...frlalargaqafggdeaaaaarlkrvtveffasgsggp....qaytgrdlrevhtgmn.....v....neqelvaaiddivlalerngig....apernevvailyslkgevlri......**

**.........|..||..|..............|..||..||...|........|..|...............|..||..||..|..||...........|..|...||........|........|..|..|||.||...............||..||..|...|.........**

**Frankia sp. EAN1pec (Actinobacteria)141aa ZP_00568165.1 GI:68228966**

**wahpakarplmsiydaigg.....asavqaavdefyvrvtad.pelapf...fag..............kdiprlkahqqafisaaiggp....evyqggaiasvhsglr.....i....tdanfnavvdhlvsalsglgvpaettgqigaalaplradivtak.........**

**.........|..||..|..............|..||..||...|........|..|...............|..||..||..|..||...........|..|...||........|........|..|..|||.||...............||..||..|...|.........**

**Frankia sp. EAN1pec 119 aa ZP_00549258.1 GI:68176033**

**..........msiydtigg.....atavqaavddfyvrvtad.pvlapl...fan..............rdlprlkehqrafiaaviggp....evyrgrdmaavhatlg.....l....tdahfdavvdhllaaltglgvptettgqigaalaplrsdvvtisk........**

**.........|..||..|..............|..||..||...|........|..|...............|..||..||..|..||...........|..|...||........|........|..|..|||.||...............||..||..|...|.........**

**Frankia sp. CcI3 (Actinobacteria)119aa ZP_00549258.1 GI:68176033**

**..........msiydtigg.....atavqaavddfyvrvtad.pvlapl...fan..............rdlprlkehqrafiaaviggp....evyrgrdmaavhatlg.....l....tdahfdavvdhllaaltglgvptettgqigaalaplrsdvvtisk........**

**.........|..||..|..............|..||..||...|........|..|...............|..||..||..|..||...........|..|...||........|........|..|..|||.||...............||..||..|...|.........**

**Gemmata obscuriglobus (Planctomycete) gnl|TIGR_214688|contig:4788:g 172aa (31-161)**

**>Gemobs**

**KNTQEPKDAKKVLWDRLGG.....ESVVKAVVHDFALAALED.PKVN.....FTRG.GKYKPSAAALEKSIVDMISDVSGGP....FKYSGKSMKEAHKGMA.....I....TDAEFDALAVHLVASLKKF....KVADPEIAELVKVVAGTREAIVE**

**.........|..||..|..............|..||..||...|........|..|.......|..||..||..|..||...........|..|...||........|........|..|..|||.||...............||..||..|...|.........**

**Hyphomonas neptunium (Alphaproteobacteria)**

**>Hypnep**

**............LYEVFGG.....EAGVSRIVDDLVERSLND.PRIADI...FAAS......DLVRLRRTLKEQFCFILAGP....CDYTGMDMASSHKDHG.....I....TNREFNALVENLQHAMNAESVPF...RAQNKLLAALAPMQRDVVT........**

**.........|..||..|..............|..||..||...|........|..|.......|..||..||..|..||...........|..|...||........|........|..|..|||.||...............||..||..|...|.........**

**Idiomarina loihiensis (Gammaproteobacteria) gi|56460041|YP_155322.1 135aa**

**>Idiloi**

**vllfsplsfaqslyqqlge.........qqgietiveemlyrvggderiahhfdgv......dimrvhkliseqvcdlsggp....cdysgedmktshrnmg.....v....dnadfnalvehliaamekedvpv...saqnqllgvlapmhgdivee.......**

**.........|..||..|..............|..||..||...|........|..|.......|..||..||..|..||...........|..|...||........|........|..|..|||.||...............||..||..|...|.........**

**Legionella pneumophila (Gammaproteobacteria) 124aa**

**>Legpne**

**........MTESLFERLGG.....QNAVNTAVDIFYRKMLMDD.RVNYF...FDDV......DMEQQILKQKGFLTMVFGGP....NQYTGKSMREGHQHL......LARGLNDSHVDIVIEHLGETLKELGANE....EDIQKVAAIANSVRGDVLGRSS....**

**.........|..||..|..............|..||..||...|........|..|.......|..||..||..|..||...........|..|...||........|........|..|..|||.||...............||..||..|...|.........**

**Methylococcus capsulatus (Gammaproteobacteria) gi|53803300|YP_114918.1 134aa**

**>Metcap1**

**KLADWWNESHGSLYEKLGG.....EAAVNAAVDIFYRKVLAD.PRINRF...FEGV......DMEKQAAKQKAFLTMAFGGP....HNYTGMDMRRGHAHL......VKQGLNDAHFDAVMEHLGATMKELN.......VPDELIAQAAAIAESTRNDVLGR...**

**.........|..||..|..............|..||..||...|........|..|.......|..||..||..|..||...........|..|...||........|........|..|..|||.||...............||..||..|...|.........**

**Microbulbifer degradans (Gammaproteobacteria) gi|4886128|ZP_00315184 145aa (18-145)**

**>Micdeg1**

**LGcsslqgqnqtlykqlgg.....qdgvsaltrqllinmaadn.riapr...frgv......nigkfktgldtylcsitdgg....cvyggdsiktihsgyn.....y....tatefnalvenlmqametlqipt...atqnkllaklapsyqdvvyq.......**

**.........|..||..|..............|..||..||...|........|..|.......|..||..||..|..||...........|..|...||........|........|..|..|||.||...............||..||..|...|.........**

**Mycobacterium avium subsp. paratuberculosis(Actinobacteria) gi|41407351|NP_960187 136aa (HbN)**

**>Mycavi1**

**ILARFRKAEPASIYDRIGG.....HEALEVVVEDFYVRVLADE.QLSGF...FTGT......NMNRLKGKQVEFFAAALGGP....HPYTGAPMKQVHQGRG.....I....TMHHFGLVAGHLADALTAAGVPS...ETVSEILGAIAPLAPEIATGEAGKVTV**

**.........|..||..|..............|..||..||...|........|..|.......|..||..||..|..||...........|..|...||........|........|..|..|||.||...............||..||..|...|.........**

**Mycobacterium bovis (Actinobacteria) gi|31618318|NP_855221 136aa (HbN)**

**>Mycbov1**

**llsrlrkrepisiydkigg.....heaievvvedfyvrvladd.qlsaf...fsgt......nmsrlkgkqveffaaalggp....epytgapmkqvhqgrg.....i....tmhhfslvaghladaltaagvps...etiteilgviaplavdvtsgesttapv**

**.........|..||..|..............|..||..||...|........|..|.......|..||..||..|..||...........|..|...||........|........|..|..|||.||...............||..||..|...|.........**

**Mycobacterium marinum (Actinobacteria) gnl|Sanger_216594|mar288e12.s1k 136aa(HbN)**

**>Mycmar1**

**LLSRFRKRAPVSIYDKIGG.....YEAIEAVVEDFYVRVLADD.QLGGF...FTGT......NMNRLKGKQAEFFAAALGGP....EPYTGAPMKQVHQGRG.....I....TMAHFSLVAGHLGDSLTAAGVPS...ETVTDILKLVAPLATDIASGETTTAGV**

**.........|..||..|..............|..||..||...|........|..|.......|..||..||..|..||...........|..|...||........|........|..|..|||.||...............||..||..|...|.........**

**Mycobacterium smegmatis (Actinobacteria) (HbN) 125aa**

**>Mycsme1**

**.........MTSIYEQIGG.....AEALEVVVEDFYRRVLADD.ELAGF...FTGT......NMSRLKGRQVEFFATALGGP....DEYTGAPMRQVHQGRG.....I....TMHHFDLVAGHLGDALSAAMPG....ATTSQIIAAIAPLAPEIATARTA....**

**Mycobacterium tuberculosis (Actinobacteria) gi|15608680|NP_216058 HbN 136aa**

**>Myctub1**

**LLSRLRKREPISIYDKIGG.....HEAIEVVVEDFYVRVLADD.QLSAF...FSGT......NMSRLKGKQVEFFAAALGGP....EPYTGAPMKQVHQGRG.....I....TMHHFSLVAGHLADALTAAGVPS...ETITEILGVIAPLAVDVTSGESTTAPV**

**.........|..||..|..............|..||..||...|........|..|.......|..||..||..|..||...........|..|...||........|........|..|..|||.||...............||..||..|...|.........**

**Myxococcus xantus (Deltaproteobacteria) gnl|TIGR_246197|contig:581:m_xanthus 116aa**

**>Myxxan1**

**...........SVYEQLGG.....EPAMAAAVEVFYRKVLADD.HISHF...FEDV......DMERQAAKQKAFLTMVTGGP....VHYSGKDMRAGHAPL......VKRGLNDSHFDAVAGHLKATLEELGV.......AAPLVARVMTIAESARADV......**

**.........|..||..|..............|..||..||...|........|..|.......|..||..||..|..||...........|..|...||........|........|..|..|||.||...............||..||..|...|.........**

**Nostoc punctiforme (Cyanobacteria) gi|23130505|ZP_00112318 118aa**

**>Nospun1**

**.........MSTLYDNIGG.....QPAIEQVVDELHKRIATD.SLLSPI...FAGT......DMAKQRNHLVAFLGQIFEGP....KQYGGRPMDKTHAG.......L..NLQQPHFDAIAKHLGEAMAVRGVSAE....NTKAALDRVTNMKGAILNK......**

**.........|..||..|..............|..||..||...|........|..|.......|..||..||..|..||...........|..|...||........|........|..|..|||.||...............||..||..|...|.........**

**Nostoc punctiforme (Cyanobacteria)gi|23126739|ZP_00108627 159aa (26-159)**

**>Nospun2**

**RVTIahnsagsslykrlgg.....ynaiaaviddsaknifad.pligky...figl...stnskqrlrelliaqfcqaaggp....ciytgrtmklshsg.......igrgltngefyafvndialaldkngvkqpe...knqvlgfanslrdqivekp......**

**Nostoc sp.PCC7120 (Cyanobacteria) 118aa AAB41122.1 GI:1100220**

**.........mstlydnigg.....qpaieqvvdelhkriatd.sllapi...fagt......dmakqrnhlvaflgqifegp....kqyggrpmdkthag.......l..nlqqphfdaiakhlgeamavrgvsaed....tkaaldrvtnmkgailnk......**

**Pelagibacter ubique (Alphaproteobacteria) YP_266627.1 GI:71083907 133aa**

**.nisiyfkimtqtlfekygg.....kktvgqivinfyqklmade.nlkkf...ftns......dmkvlishqtnfisqalggp....keytgldmktahqgmk.....i....sqedfngvaghlkqtleelnvesqd....vdtiigvvaplsehivsk......**

**.........|..||..|..............|..||..||...|........|..|.......|..||..||..|..||...........|..|...||........|........|..|..|||.||...............||..||..|...|.........**

**Pseudomonas syringae (Gammaproteobacteria) gi|28871348|NP_793967 140aa**

**>Psesyr1**

**AGCAQQPPRDDSLYQDLGQ.....RAGIQRIVEGMLLNIAKDE.RIVEH...FKKV......NIVRLRDKLVEQLCVEAGGP....CRYTGDSMAESHKGQN.....L....TPSDFNALVENLIAAMDTENVPVP...VQNRLIARLAPMRGEVLGK.......**

**.........|..||..|..............|..||..||...|........|..|.......|..||..||..|..||...........|..|...||........|........|..|..|||.||...............||..||..|...|.........**

**Pseudomonas syringae gi|23471254|ZP_00126585 142aa**

**>Psesyr2**

**caqqqqppkddslyqdlgq.....ragiqrivegmlinvardq.riveh...frkv......divrlrdklveqlcvesggp....CRYTGDSmaeahkgqn.....l....tpsdfnalvedliasmdtekvpvp...aqnrliarlapmrgevigk.......**

**Rhodoferax ferrireducens (Betaproteobacteria) 151aa EAO39787.1 GI:72603787 (24-**

**pqyqkefpmtttlyqrlgg.....adgiarlvddiiathlan.plvktr...feki.....tdmahikklsceflttgsggp....qtytgqgmlaahkgmn.....i....seqeflvvvddifvalnkngided...skkdvlailyslkdqiirv.......**

**.........|..||..|..............|..||..||...|........|..|.......|..||..||..|..||...........|..|...||........|........|..|..|||.||...............||..||..|...|.........**

**Rhodopirellula baltica gi|32475444|NP_868438 127aa**

**>Rhobal**

**.....MSQSESDLLDQLGG.....MDGVRRVVDEMYVRVLAD.PELTHF...FDGV......PIEKLARMQTEFIASITSGD....IQYTGADLTKVHAGRG.....I....TGVHFSKFCGHLTDALEANNVSSH...AIDQVLGKLAMYSDKVTGSSNVDG..**

**Rubrivivax gelatinosus (Betaproteobacteria) 143aa ZP_00245120.1 GI:47575085**

**saaaqstppddalyralgg.....tpgltklmadfvprlaad.prigef...fke......tklselqkqlvdqfcvvsggs....cvyegetmknshaemk.....i....rkadfnalvevlqesmdrqgipfs...vqnrllarlapmhrdivntr......**

**Shewanella amazonensis (Gammaproteobacteria)158aa EAN39521.1 GI:68515807 (30-**

**tttpgenaspptlyqalgg.....etgvaavvdgllariard.privqh...fee.....tdiaifrerliehlcavtdggc.....vyqgesmadshkgln.....i....tqadfdalvghlieslkaqqipts...trnallkrlapmysdvtyq.......**

**Shewanella baltica (Gammaproteobacteria)158aa ZP_00583042.1 GI:68543333**

**vnlnngnssqqtlyqelgg.....eaglsaivdgllariatd.privhh...fqe.....tdialfreraiehfclvadggc.....vyhgenmalshqgln.....i....tqadfdalvghliesmkeqhipls...trnallnrlapmysdityh.......**

**Shewanella denitrificans (Gammaproteobacteria) 159aa ZP_00633441.1 GI:69941507 2/2Hb1**

**saetakdsslysalgkqgg........iaaivdgllagiaadq.rivqh...frd.....tditlfrqrlieylclvtdggc.....vylgesmaeshqglg.....i....tqahfdalvghlihsmkqqsvplg...trnallkrlaskyedisyr.......**

**Shewanella frigidimarina (Gammaproteobacteria) 143aa ZP_00639119.1 GI:69951378**

**llsvacasqpttlyqqlggd.....kgvsmitddflvnlsqdk.rilhh...feq......tdiqifrqrliehlcvvsggecvyqgasmynahiglhisqadfdavv.....ghliealkqqhiplatrnallae...laplyqdiieh...............**

**.........|..||..|..............|..||..||...|........|..|.......|..||..||..|..||...........|..|...||........|........|..|..|||.||...............||..||..|...|.........**

**Silicibacter pomeroyi (Alphaproteobacteria) gi|56696447|YP_166804.1 150 aa**

**>Silpom**

**.........mtsaltsigg.....edalrdlvetfydrmetdpeawplhrlhfrg......hglnhtrqaqfeflsgflggrayyrerfghmnlreihahvp.....i....readaelwlktfdaalaecglrgevvdkfratlrraalmlvndvpdwreesd.**

**.........|..||..|..............|..||..||...|........|..|.......|..||..||..|..||...........|..|...||........|........|..|..|||.||...............||..||..|...|.........**

**Solibacter usitatus (Acidobacteria) gi|67932871|ZP_00526004133aa**

**>Solusi1**

**......msdekslyarlgg.....ydaisavvdnllprlmsd.sllgr....fw.a.hrgedgilrekqllvdflcqsaggP....vyytgrdmklshkgmq.....i....tesdwqaflghltatldafav....papeketvlafihstkadiidppgskaa**

**.........|..||..|..............|..||..||...|........|..|.......|..||..||..|..||...........|..|...||........|........|..|..|||.||...............||..||..|...|.........**

**Solibacter usitatus (Acidobacteria) gi|67929246|ZP_00522429 339aa**

**>Solusi2**

**ntrqfrydvrvaiydaigg.....magcrrlaaafyarverd.pvvrpl...ypk.......slhcavdaiamflvqqfggpceysenratlslyeshlrfa.....i....gqaerdawlrdmtaamddigi...geperaemvlyfeqtsaylinrpkpdrap**

**.........|..||..|..............|..||..||...|........|..|.......|..||..||..|..||...........|..|...||........|........|..|..|||.||...............||..||..|...|.........**

**Synechococcus sp.PCC7002 (Cyanobacteria) gi|18766961|AAL79195.1 124aa**

**>Syncoc**

**.........maslyeklgg.....aaavdlavekfygkvlade.rvnrf...fvnt......dmakqkqhqkdfmtyafggt....drfpgrsmraahqdlvenag.l....tdvhfdaiaenlvltlqelnv.......SQDlidevvtivgsvqhrndvlnr.**

**.........|..||..|..............|..||..||...|........|..|.......|..||..||..|..||...........|..|...||........|........|..|..|||.||...............||..||..|...|.........**

**Synechocystis sp.PCC6803(Cyanobacteria) gi|16330583|ref|NP_441311 124aa**

**>Syncys**

**.........mstlyeklgg.....ttavdlavdkfyervlqdd.rikhf...fadv......dmakqrahqkafltyafggt....dkydgrymreahkelvenhg.l....ngehfdavaedllatlkemgv.......pedliaevaavagapahkrdvlnq.**

**.........|..||..|..............|..||..||...|........|..|.......|..||..||..|..||...........|..|...||........|........|..|..|||.||...............||..||..|...|.........**

**Xanthomonas axonopodis (Gammaproteobacteria) gi|21243436|NP_643018 131aa**

**>Xanaxo**

**TSACASTQSRQTLYDELGG.....QAGIEALVETMLSRIADD.PRIVDK...FARV......NIVMLNERLVQKFCHVADGP....CPDTAKSMQQAHAHLA.....I....REGDFNALVEDLNWAMDQRKIP...RRTQNRLLARLAAMHGEIVNH.......**

**.........|..||..|..............|..||..||...|........|..|.......|..||..||..|..||...........|..|...||........|........|..|..|||.||...............||..||..|...|.........**

**Xanthomonas campestris (Gammaproteobacteria) gi|21231965|NP_637882 142aa**

**>Xancam**

**LSACATTQPRQTLYEELGG.....QPGIEALVETMLSRIADD.PRIVEH...FARV......NIVMLNERLVQKFCHVADGP....CPDTAKSMAQAHQHMA.....I....REDDFNALVEDLNWAMDQRNIP...RRTQNRLLARLAAMHGEIVNH.......**

**.........|..||..|..............|..||..||...|........|..|.......|..||..||..|..||...........|..|...||........|........|..|..|||.||...............||..||..|...|.........**

**Xanthomonas oryzae (Gammaproteobacteria) gi|58582863|YP_201879 199aa (73-199)**

**>Xanory**

**tsacastqprqtlydelgg.....qagiealvetmlsriaddq.rivdk...farv......nivmlnerlvqkfchladgp....cpdtaksmqqahahla.....i....regdfnalvedlnwamdqrkip...rrtqnrlltrlaamhgdivnh.......**

**2/2Hb2s**

**Acidithiobacillus ferrooxidans (Gammaproteobacteria) TIGR_243159|contig:10428:a 129aa**

**>Acifer1**

**.....MLEPESTPYVRLGG.....EEAVRNLVNRFYDLMDSE.AQWQTPLRDIHAK......DLSESREKLFLFLSGWLGGP..YVQRFGHPRLRARHASVP.....V....DDQARDQWMACMLQAMHEV.......GIEPELYGHLQGAFQRTADFMRNR...**

**.........|..||..|..............|..||..||...|........|..|.......|..||..||..|..||...........|..|...||........|........|..|..|||.||...............||..||..|...|.........**

**Agrobacterium tumefaciens (Alphaproteobacteria) gi|15889651|NP_355332 TrHb2**

**>Agrtum1**

**SKEATMSSETVTLYEAIGG.....DATVRALTRRFYELMDTL.PEAARCRA.IHPA......DLSGSEAKFYDYLTGYLGGPPVYVEKHGHPMLRRRHFVAP.....I....GPAERDEWLLCFRRAMDETIEN....AKLREIIWAPVERLAFHMQNQEADNP.**

**.........|..||..|..............|..||..||...|........|..|.......|..||..||..|..||...........|..|...||........|........|..|..|||.||...............||..||..|...|.........**

**Azotobacter vinelandii (Gammaproteobacteria) gi|23105627|ZP_00092083 131aa**

**>Azovin**

**......minvltpyqllgg.....eegvrrlcnafydnmeql.peaegirr.m......hgadttavreklfeylsgwlggp..ylekygtvsmglphrpfa.....i....gpkerdqwmlclnkalddigas....ETakglikapihafadhvrnretsd..**

**.........|..||..|..............|..||..||...|........|..|.......|..||..||..|..||...........|..|...||........|........|..|..|||.||...............||..||..|...|.........**

**Bacillus anthracis (Firmicutes) gi|30261307|NP_843684 132aa**

**>Bacant**

**.....MSKQPMTPFEAIGG.....EQCIEILVDTFYSYVSKH.PDLSPI...FP.......DDLTETARKQKQFLTQYLGGP..YTEEHGHPMLRARHLPFE.....I....TPKRAEAWLSCMEQAMDDTGVHG...HIREFVFERLALTAQHMVNTPNETGEI**

**.........|..||..|..............|..||..||...|........|..|.......|..||..||..|..||...........|..|...||........|........|..|..|||.||...............||..||..|...|.........**

**Bacillus cereus (Firmicutes) gi|30019349|NP_830980 132aa**

**>Baccer**

**.....MSKQPMTPFEAIGG.....EQCIAILVDTFYSYVSKH.PDLSPI...FP.......EDLTETARKQKQFLTQYLGGP..YTEEHGHPMLRARHLPFE.....I....TPKRAEAWLSCMEQAMDDTGVHGH...IREFVFERLALTAQHMVNTPNETGEV**

**.........|..||..|..............|..||..||...|........|..|.......|..||..||..|..||...........|..|...||........|........|..|..|||.||...............||..||..|...|.........**

**Bacillus clausii gi|56964289|YP_176020.1 131aa**

**>Baccla**

**...mpdqrneqspfealgg.....eekiselvdafyryiaahe.dvrhl...fp.......edlthtaykqkrfltqffggpplyteefghprlrarhmpfv.....i....spvqaeawlscmeramddvhlsgd...irdymmhrlrltahhmvnhptpg...**

**Bacillus halodurans (Firmicutes) gi|15615417|NP_243720**

**>Bachal**

**.....mvelnrtvyerigg.....dktvsalvdafytnvagd.pilapi...fp.......ndltetkrkqqqfltqflggp..yteehghpmlrarhlpfp.....i....tprrakawltcmeramdeinlPSP...ereemmarltmtahhmvnqpeehr..**

**.........|..||..|..............|..||..||...|........|..|.......|..||..||..|..||...........|..|...||........|........|..|..|||.||...............||..||..|...|.........**

**Bacillus licheniformis (Firmicutes) gi|52079659|YP_078450 132aa**

**>Baclic**

**.....mgqsfnapyeaig......eellsqlvdtfyervkrh.pllypi...fp.......ddltetarkqkqfltqylggpplyteehghpilrarhlpfp.....i....tperadawlscmkeamdhvglKGE...ireflferltltarhmvnqpkmedgs**

**.........|..||..|..............|..||..||...|........|..|.......|..||..||..|..||...........|..|...||........|........|..|..|||.||...............||..||..|...|.........**

**Bacillus stearothermophilus gnl|OUACGT_1422|bstearo.fasta.screen.Contig264:6736:7023 96aa**

**..........QTLYEAIGG.....EETVAKLVEAFYRRVAAH.PDLRPI...FPD.......DLTETAHKQKQFLTQYLGGPPLYTAEHGHPMLRARHLRFE.....I....TPKRAEAWLACMRAAMDEI**

**.........|..||..|..............|..||..||...|........|..|.......|..||..||..|..||...........|..|...||........|........|..|..|||.||...............||..||..|...|.........**

**Bacillus subtilis (Firmicutes) gi|16078221|NP_389038**

**>Bacsub2**

**.....MGQSFNAPYEAIGE......ELLSQLVDTFYERVASH.PLLKPI...FP.......SDLTETARKQKQFLTQYLGGP..YTEEHGHPMLRARHLPFP.....I....TNERADAWLSCMKDAMDHVGLEGE...IREFLFGRLELTARHMVNQTEAEDRS**

**.........|..||..|..............|..||..||...|........|..|.......|..||..||..|..||...........|..|...||........|........|..|..|||.||...............||..||..|...|.........**

**Bacillus thuringiensis (Firmicutes) gi|49477128|YP_035435.1 132aa TrHb2**

**>Bacthu**

**.....mskqpmtpfeaigg.....eqciailvdtfysyvskh.pdlspi...fp.......ddltetarkqkqfltqylggpnlyteehghpmlrarhlpfe.....i....tpkraeawlscmeqamddtgvHGH...irefvferlaltaqhmvntpnetgei**

**.........|..||..|..............|..||..||...|........|..|.......|..||..||..|..||...........|..|...||........|........|..|..|||.||...............||..||..|...|.........**

**Bdellovibrio bacteriovorus (Deltaproteobacteria)gi|39576266|NP_969438 137aa**

**>Bdebac1**

**......mseekkpyellgg.....eevlrqlckrfyeimdti.peakgird.mh......pgnlrgseeklfmflsgwlggpglfvekyghprlrmrhfpfk.....i....gkserdqwmmcmvqafdelniAEP...lrsellhsllrladhmrnveepesse**

**.........|..||..|..............|..||..||...|........|..|.......|..||..||..|..||...........|..|...||........|........|..|..|||.||...............||..||..|...|.........**

**Bordetella pertussis (Betaproteobacteria) gi|33593344|NP_880988 141aa**

**>Borper1**

**qpisepldtsktvfdmlgg.....epgvrelvdrfydlmdme.sd.......fkalrdahGpsleqardklfwflcgyfggph.yierfghprlrarhlpfs.....i....geierdqwvacmgramqdqql.......paalvdrllqafygtadwmrnrag.**

**.........|..||..|..............|..||..||...|........|..|.......|..||..||..|..||...........|..|...||........|........|..|..|||.||...............||..||..|...|.........**

**Bradyrhizobium japonicum (Alphaproteobacteria) gi|27376186|NP_767715 131aa**

**>Brajap1**

**..MMTDSDVAISMFERIGG.....SATIDLLVDRFYERMDTL.PEAKV....IRAM...HAADLGLIRDVLKRYLTEWTGGPRLYSVEKGHPRLRQRHIGFA.....I....GDAERDAWLTCMRGALEETVAD...AAARQDLDKALSGLADWMRNR.......**

**.........|..||..|..............|..||..||...|........|..|.......|..||..||..|..||...........|..|...||........|........|..|..|||.||...............||..||..|...|.........**

**Brevibacterium linens (Actinobacteria) gi|62423976|ZP_00379129.1 133 aa**

**>Brelin**

**....mtqtsnetifeavgg.....hptftrlvdvfyehvdqde.vliam...ype.....ghdltgakhrlqmfleqyfggpttyqeerghprlrmrhfpfp.....v....dfdardrwlasmraalddvala....plydeifwdyfqraatamvntnsnpv.**

**.........|..||..|..............|..||..||...|........|..|.......|..||..||..|..||...........|..|...||........|........|..|..|||.||...............||..||..|...|.........**

**Burkholderia ambifaria (Betaproteobacteria) 165aa EAO43582.1 GI:72607624 (30)**

**tdvnddapsqptafelvgg.....earvremvdrfydlmdle.pe.......faeiralhpasldgsrdklfwflcgwlggpdhyisrfghprlrarhlpfp.....i....asverdqwlrcmawamedtglpep...lrerlmhsfydtadwmrnrpg.....**

**..........|..||..|..............|..||..||...|........|..|.......|..||..||..|..||...........|..|...||........|........|..|..|||.||...............||..||..|...|........**

**Burkholderia cepacia (Betaproteobacteria)gi|46323938|ZP_00224300 142aa**

**>Burcep1**

**ahslhaahappspfdrlgg.....aaalarivdafyrqmdtr.pdaagira.mhga......dlgpvktvlvtylcewlggprhysaqrghprlrmrhrafa.....i....gmaerdawlacmraaldecgvep...alrdelmqalfkladwlrntgr.....**

**.........|..||..|..............|..||..||...|........|..|.......|..||..||..|..||...........|..|...||........|........|..|..|||.||...............||..||..|...|.........**

**Burkholderia cepacia (Betaproteobacteria)gi|46311329|ZP_00211937 136aa**

**>Burcep2**

**tdvnddapsqptafelvgg.....earvremvdrfydlmdle.pe.......faqiralhpdsldgsrdkffwflcgwlggpdhyisrfghprlrarhlpfp.....i....asverdqwlrcmawamddiglpe...plrerlmhsfydtadwmrnrpg.....**

**.........|..||..|..............|..||..||...|........|..|.......|..||..||..|..||...........|..|...||........|........|..|..|||.||...............||..||..|...|.........**

**Burkholderia fungorum (Betaproteobacteria) gi|48786054|ZP_00282263 139aa**

**>Burfun1**

**aipssqqlprathfdrlgge.....pavirlvdafyrrmdtr.pdaqrirA.Mhhp......dlsstkavlvlylcewlggdkqysaqrghprlrmrhaafa.....i....glaerdawltcmrgaldetgvdp...alrdelmqaffktadwmrntpt.....**

**.........|..||..|..............|..||..||...|........|..|.......|..||..||..|..||...........|..|...||........|........|..|..|||.||...............||..||..|...|.........**

**Burkholderia fungorum (Betaproteobacteria) gi|48784261|ZP_00280627 137aa**

**>Burfun2**

**dlndevsaaqptafelvgge.....arvrelvdrfydlmdle.ad.......fagiralhppsldgsrdkffwflcgwlggp.hyisrfghprlrarhlpfa.....i....asserdqwlrcmawamedvglaep...lrerllgsffetadwmrnrng.....**

**.........|..||..|..............|..||..||...|........|..|.......|..||..||..|..||...........|..|...||........|........|..|..|||.||...............||..||..|...|.........**

**Burkholderia mallei (Betaproteobacteria) gi|53725407|YP_103467.1 148aa (14-148)**

**>Burmal**

**IDVTDDAPSPPTAFELVGGE.....ARVRELVDRFYDLMDLE.PE.......FAGIRALHPPTLEGSRDKLFWFLCGWLGGP.HYIERFGHPRLRARHLPFP.....I....ASSERDQWLRCIAWAMQDVGLDEP...LRERLMHSFHDTADWMRNRPG.....**

**.........|..||..|..............|..||..||...|........|..|.......|..||..||..|..||...........|..|...||........|........|..|..|||.||...............||..||..|...|.........**

**Burkholderia pseudomallei (Betaproteobacteria) gi|53718816|YP_107802.1 148aa (36-170)**

**>Burpse**

**tdvtddapspptafelvgge.....arvrelvdrfydlmdle.pe.......Fagiralhpptlegsrdklfwflcgwlggp.hyierfghprlrarhlpfp.....i....asserdqwlrciawamqdvgldep...lrerlmhsfhdtadwmrnrpg.....**

**.........|..||..|..............|..||..||...|........|..|.......|..||..||..|..||...........|..|...||........|........|..|..|||.||...............||..||..|...|.........**

**Burkholderia vietnamiensis 142aa EAM32321.1 GI:67535594**

**ahslhaahappspfdrlgg.....aaalarivdafyrqmdtr.pdaagira.mhga......dlgpvktvlvtylcewlggprhysaqrghprlrmrhrafa.....i....gmaerdawlacmraaldecgvepa...lrdelmqalfkladwlrntgr.....**

**.........|..||..|..............|..||..||...|........|..|.......|..||..||..|..||...........|..|...||........|........|..|..|||.||...............||..||..|...|.........**

**Chloroflexus aurantiacus (Chloroflexi; Green non-sulfur bacteria) gi|22973316|ZP_00020053 140aa**

**>Chlaur**

**hygevyimseptiyeqigg.....eatfrrivdifyarvead.prlrhl...fp.......adlepgkehqrlflmqyfggp.tyserrghprlrmrhapfp.....i....gprerdawlehmlaalneagvpe....parsvmenyfrhaaqammnrlged..**

**.........|..||..|..............|..||..||...|........|..|.......|..||..||..|..||...........|..|...||........|........|..|..|||.||...............||..||..|...|.........**

**Chromobacterium violaceum (Betaproteobacteria) gi|34499146|NP_903361 129aa**

**>Chrvio**

**....MSEVQEMTPYQLLGG.....EGVVRWLTDRFYDIMDSE.PSVKPLRD.M.HP.....ADLAGSRQKLFMFLSGWLGGP.LYMEAFGHPRLRMRHMPFA.....V....DEDARDQWMFCMRRAVSEL.......VVEDWLKDKLLEAFHNTADFMRNR...**

**Chromohalobacter salexigens (Gammaproteobacteria)139aa ZP_00473252.1 GI:67676504**

**mkphrvfgvgdstlravgg.....eaavrelvdrfydamerl.pearg....i...ralhpadldesrdklttfligwmggpsryrerfgpiaipaahrhld.....i....gpeerdawlacmataleei.......gaaddlkrylltqlrhpaemcrrr...**

**.........|..||..|..............|..||..||...|........|..|.......|..||..||..|..||...........|..|...||........|........|..|..|||.||...............||..||..|...|.........**

**Corynebacterium diphtheriae (Actinobacteria) gi|38234371|NP_940138.1 130aa**

**>Cordip**

**......MNTPASFYESVGG.....EETFHLIVHRFYERMRNDD.LIGPM...YP......DDDWEGAEDRLRWFLAQYWGGP.TFSENRGHPRLRMRHAHFP.....I....GMNEAQRWLDIMSDTLDSIEATLP.PAHRAAMWDHMQRVAQMLINQAP.....**

**.........|..||..|..............|..||..||...|........|..|.......|..||..||..|..||...........|..|...||........|........|..|..|||.||...............||..||..|...|.........**

**Corynebacterium efficiens (Actinobacteria) gi|25028901|NP_738955 136aa**

**>Coreff**

**......MTSTENFYDSVGG.....EETFNLIVHRFYEQVRTDD.ILGPM...YPQ......DDWEGAEARLRMFLVQYWGGP.DYSEQRGHPRLRMRHAHFP.....I....GVTAAERWLELMTRALDSLEEGTLTDDQRAAIWDHMVRAADMLINSNPDPHQR**

**.........|..||..|..............|..||..||...|........|..|.......|..||..||..|..||...........|..|...||........|........|..|..|||.||...............||..||..|...|.........**

**Corynebacterium glutamicum (Actinobacteria) gi|19553644|NP_601646 131aa**

**>Corglu**

**......MTTSENFYDSVGG.....EETFSLIVHRFYEQVPNDD.ILGPM...YPP......DDFEGAEQRLKMFLSQYWGGP.DYQEQRGHPRLRMRHVNYP.....I....GVTAAERWLQLMSNALDGVDLTAE...QREAIWEHMVRAADMLINSNPDPHA.**

**.........|..||..|..............|..||..||...|........|..|.......|..||..||..|..||...........|..|...||........|........|..|..|||.||...............||..||..|...|.........**

**Cytophaga hutchinsonii (Bacteroideted/Chlorobi; green sulfur bacteria) gi|23136839|ZP_00118553 125aa**

**>Cythut**

**.......mekealyyrlgk......enldllvdrfydlvfvnd.qiahl...fkn.......dkeeikgkqrlfltqflggp.lysekfghpqmrarhmphp.....I....tetdaiawlhcmsqaigslpvsea...lkdelfarfpptamfmvnkeeq....**

**.........|..||..|..............|..||..||...|........|..|.......|..||..||..|..||...........|..|...||........|........|..|..|||.||...............||..||..|...|.........**

**Dechloromonas aromatica (Betaproteobacteria)gi|41725549|ZP_00152307 129 aa**

**>Decaro**

**......mentattyekigg.....epvvgklcdrfyelmgti.pqfaelra.mhp......edlqgsrdklymflsgwfggpdlfvekfghprlrgrhmpfa.....i....gvkerdqwvacmalamedvglsed...irkvllnnffntadfmrnkse.....**

**Deinococcus radiodurans (Thermus/Deinococcus) gi|15806025|NP_294726 149aa (1-134)**

**>Deirad**

**.MTTPLDLARGSLYDRIGP......DTLALLVHRFYAHVARN.PDLAPI...FP.A......DLSETARKQLAFLTGFTGGP.LYHELYGHPRLRARHLPFP.....I....TPGRARAWLACMNAALRETPGLAEA..DAHELYAALARVAVHMVNTEEAPDSD**

**.........|..||..|..............|..||..||...|........|..|.......|..||..||..|..||...........|..|...||........|........|..|..|||.||...............||..||..|...|.........**

**Deinococcus geothermalis (Deinococcus-Thermus)144aa EAL82974.1 GI:66782009 2/2Hb2**

**mtapiplreggslydrigp......ealarlvqhfyarvaah.pdlapi...fp.a......dltltaekqlafltgflggpplyhqrfgpprlrarhlpfp.....i....tptrarawlacmqaalretpeiaea..earelyaalsrvavhmvntpeaepep**

**.........|..||..|..............|..||..||...|........|..|.......|..||..||..|..||...........|..|...||........|........|..|..|||.||...............||..||..|...|.........**

**Exiguobacterium (Firmicutes) gi|45532753|ZP_00183753 126aa**

**>Exiguo**

**...........mlyedlgq.....kqaieqlvarfyervyadh.llrpl...fp.......sdrrqveqaqtrfliqltggpkqyesydermnlamihrllp.....i....tevhairwielmtmtieetisdsea...atrlierlrigalnvlricdahqnr**

**Frankia sp. EAN1pec 139 aa ZP_00571626.1 GI:68232479 2/2Hb2**

**vnqpsparpradfyeavgg.....eatfralvarfyegvasd.pvlrpl...ypd......edlaaaeerlrlfliqywggpttyseqrghprlrmrhvpfa.....i....gpaerdawlrimesavdslglapeh...raqlwdyllmaanslqnrpg.....**

**.........|..||..|..............|..||..||...|........|..|.......|..||..||..|..||...........|..|...||........|........|..|..|||.||...............||..||..|...|.........**

**Frankia sp. EAN1pec 140 aa ZP_00548400.1 GI:68175146 2/2Hb2**

**nqspprtlpissfydaagg.....eptfrklvarfyqgvand.pvlrpl...ype......edltgaeerlrmfliqywggptdyqeqrghprlrrrhapfa.....i....gptqrdawlkimraavdsldlppdld...rqlwdylsmaanslqnrpd.....**

**.........|..||..|..............|..||..||...|........|..|.......|..||..||..|..||...........|..|...||........|........|..|..|||.||...............||..||..|...|.........**

**Frankia sp. CcI3 (Actinobacteria) 140aa ZP_00548400.1 GI:68175146 2/2Hb2**

**nqspprtlpissfydaagg.....eptfrklvarfyqgvand.pvlrpl...ype......edltgaeerlrmfliqywggptdyqeqrghprlrrrhapfa.....i....gptqrdawlkimraavdsldlppdld...rqlwdylsmaanslqnrpd.....**

**.........|..||..|..............|..||..||...|........|..|.......|..||..||..|..||...........|..|...||........|........|..|..|||.||...............||..||..|...|.........**

**Jannaschia sp. CCS1 (Alphaproteobacteria; Rhodobacterales) 177aa ZP_00414580.1 GI:66967031 (43-**

**ndpfsrpgytdnfydavgg.....hetfvklidvfydgvatd.p...llrpmypea......dlgpakrrflmfleqywggpttygeerghprlrmrhmpfr.....v....tpeakdrwlhhmrtavdalelppl....yegtlwdymeraalsminspsda..**

**.........|..||..|..............|..||..||...|........|..|.......|..||..||..|..||...........|..|...||........|........|..|..|||.||...............||..||..|...|.........**

**Kineococcus radiotolerans (Actinobacteria) gi|46365419|ZP_00227907.1 149aa**

**>Kinrad**

**rkpagpdggtrsffdevgg.....hetfvrlvdafyegvaade.vlrpm...yp......eadlgparqrmlmfleqywggpatyseqrghprlrmrhasfk.....v....npdardrwlthmraavvslglapA....qegvlwdyldraahsmlntfed...**

**.........|..||..|..............|..||..||...|........|..|.......|..||..||..|..||...........|..|...||........|........|..|..|||.||...............||..||..|...|.........**

**Leifsonia xylii (Actinobacteria) gi|50954582|YP_061870 135aa**

**>Leixyl**

**tgipvgppagptffeqagg.....hdtfrrlvdafyrgvaad.pvlrpm...yp......eedlepaaerltlfleqywggpaayseqrghprlrmrhlpfr.....v....npdardrwlmnmraavlelalppl....qeetlwsyleraafamvntfee...**

**.........|..||..|..............|..||..||...|........|..|.......|..||..||..|..||...........|..|...||........|........|..|..|||.||...............||..||..|...|.........**

**Leptospira interrogans (Spirochaetes) gi|24216675|NP_714156 145aa**

**>Lepint**

**GGPPGPIPGLQLVFGSVGE......NSLRKLVSDFYDQIPSS.SIS.FM...FP.......ENLEDSKIKSADFLIQVTGGP.LYSQNYGPPKMRARHLPFP.....I....DEKARRVWLSCYRKVLDDWDAEVS....AKEVLWIFFKDFSTWMVNLESKTEE**

**.........|..||..|..............|..||..||...|........|..|.......|..||..||..|..||...........|..|...||........|........|..|..|||.||...............||..||..|...|.........**

**Mesorhizobium loti (Alphaproteobacteria) gi|13470534|NP_102102 160aa (1-133)**

**>Meslot**

**.....MSRDVPTLYEWAGG.....SDALNRLTQTFYDKVAKD.PVVGPV...FKAM.......SPDHPSHVAAFIGEVFGGP.YSEKFGGHREMVMHHLGKH.....L....TEEQRRRWINLLADAADEVGLPDDPE..FRSAFMGYVEWGSRLAKMNSNLGET**

**.........|..||..|..............|..||..||...|........|..|.......|..||..||..|..||...........|..|...||........|........|..|..|||.||...............||..||..|...|.........**

**Methylobacillus flagellatus (Betaproteobacteria) gi|46120937|ZP_00201861 137aa**

**>Metfla**

**dkigepgsskatlfellgg.....aeqvraiverfydimdtd.praagira.mhAP......dltsareklfmfltgwtggpqlymeryghprlrmrhmsfp.....i....ddsardqwmycmvkamhdtgvdeAI....iekmgaalygvadfmrnreg....**

**.........|..||..|..............|..||..||...|........|..|.......|..||..||..|..||...........|..|...||........|........|..|..|||.||...............||..||..|...|.........**

**Methylococcus capsulatus (Gammaproteobacteria) 131aa**

**>Metcap2**

**....MQEPVIQTPYQRLGG.....EAVLHELVERFYGYMDEL.PEAAPIRA.MHA......DDLSGAKSKLFKFLSGWLGGP.LFVQEFGHPRLRARHFPFS.....I....GVPERDQWLLCMRKALDDIPLDGPF...REALYEALARTAHHMINRED.....**

**.........|..||..|..............|..||..||...|........|..|.......|..||..||..|..||...........|..|...||........|........|..|..|||.||...............||..||..|...|.........**

**Microbulbifer degradans (Gammaproteobacteria) gi|48862691|ZP_00316586 137aa**

**>Micdeg2**

**..mneygygqgdasykaage....iegitrlvdafyhfmdtl.peaaklrs.mhAQ......slaesrkklayflsgwlggprlyaqhfgsinipgahrh.......l..aatpedadawmlcmakavdlqpyep...sfkqyllqqlrvpadriveagrkpl..**

**.........|..||..|..............|..||..||...|........|..|.......|..||..||..|..||...........|..|...||........|........|..|..|||.||...............||..||..|...|.........**

**Mycobacterium avium (Actinobacteria)gi|41408389|NP_961225 (HbO) 129aa**

**>Mycavi2**

**...........SFYDAVGG.....AETFQAIVSRFYAQVPEDE.ILREL...YPL......DDLEGAEERLRMFLEQYWGGP.TYSDRRGHPRLRMRHVPFR.....I....TPLARDAWLRCMHTAVASIDSKTLD.DEHRRELLDYLEMAAHSLVNSP.....**

**.........|..||..|..............|..||..||...|........|..|.......|..||..||..|..||...........|..|...||........|........|..|..|||.||...............||..||..|...|.........**

**Mycobacterium bovis (Actinobacteria) gi|31793651|NP_856144 128aa (HbO)**

**>Mycbov2**

**........mpksfydavgg.....aktfdaivsrfyaqvaede.vlrrv...ype......Ddlagaeerlrmfleqywggp.tyseqrghprlrmrhapfr.....i....slierdawlrcmhtavasidsetld.DEhrrelldylemaahslvnspf....**

**.........|..||..|..............|..||..||...|........|..|.......|..||..||..|..||...........|..|...||........|........|..|..|||.||...............||..||..|...|.........**

**Mycobacterium leprae (Actinobacteria) gi|15827640|NP_301903 128aa**

**>Myclep2**

**........MQQSFYDAIGG.....AETFKAIVSRFYAQVPEDE.ILREL...YPA......DDLAGAEERLRMFLEQYWGGP.tyssqrghprlrmrhapfr.....i....taierdawlrcmhtavasidshtld.nehrrelldylemaahslvnsas....**

**.........|..||..|..............|..||..||...|........|..|.......|..||..||..|..||...........|..|...||........|........|..|..|||.||...............||..||..|...|.........**

**Mycobacterium smegmatis (Actinobacteria) 136aa (HbO)**

**>Mycsme2**

**..MGDVTQVQRSFYDEVGG.....HDTFHAIVSRFYQLVREDE.ILHPL...YPE......DDFEGAEERLRMFLEQYWGGP.TYSDQRGHPRLRMRHAPFR.....I....GFLERDAWLRCMHTAVAEIDSQTLD.DAHRRALLDYLMADSMVNSAF......**

**.........|..||..|..............|..||..||...|........|..|.......|..||..||..|..||...........|..|...||........|........|..|..|||.||...............||..||..|...|.........**

**Mycobacterium tuberculosis (Actinobacteria) gi|15609607|NP_216986 HbO 128aa**

**>Myctub2**

**........MPKSFYDAVGG.....AKTFDAIVSRFYAQVAEDE.VLRRV...YPE......DDLAGAEERLRMFLEQYWGGPRTYSEQRGHPRLRMRHAPFR.....I....SLIERDAWLRCMHTAVASIDSETLD.DEHRRELLDYLEMAAHSLVNSPF....**

**.........|..||..|..............|..||..||...|........|..|.......|..||..||..|..||...........|..|...||........|........|..|..|||.||...............||..||..|...|.........**

**Nocardia farcinica (Actinobacteria) gi|54014789|BAD56159 134aa**

**>Nocfar**

**..mssgeqtatsfyeavgg.....aetfrrlvaafyrevaade.ilrpl...ypE......Ddlgpaerrlrmfleqywggprtysderghprlrmrhmpfk.....v....gplerdawlrcmriavaelepevld.dehrkalldylemaahslmnspi....**

**Nocardioides sp. JS614 (Actinobacteria) 167aa ZP_00656798.1 GI:71366253 (35-**

**spgdrqngpvstfyeeigg.....fetirrivarfyegvaad.pvlrpm...ype......edlgpaeerfllflvqywggpttysdsrghprlrarhapfa.....v....tpeakehwlrhfragldevaltpeqdaqfwdyithaaqfmvnsle........**

**.........|..||..|..............|..||..||...|........|..|.......|..||..||..|..||...........|..|...||........|........|..|..|||.||...............||..||..|...|.........**

**Novosphingobium aromaticivorans (Alphaproteobacteria) gi|48850360|ZP_00304602 131aa**

**>Novaro1**

**...Mateapalspydrigg.....revlrritdrfydlmdtd.pa.......yaelramhapdlspmrealagflsgwcggpRgwfeanpgkcmmsmhkpfp.....i....trqtatqwadcmsraiadaapedte...vadamaqvlgqmakgmard......**

**.........|..||..|..............|..||..||...|........|..|.......|..||..||..|..||...........|..|...||........|........|..|..|||.||...............||..||..|...|.........**

**Novosphingobium aromaticivorans (Alphaproteobacteria) gi|48847810|ZP_00302059 133aa .**

**>Novaro2**

**......mtvpgtpyeafgg.....eaairalcgrfyslmdel.pgaaacra.vhpp......sleraeerlfeyltgwlggpplytdkyghprlrmrhfiap.....i....greevegwllcfrqawsetipaspl....adaimekveglawhmgnkpdiags**

**.........|..||..|..............|..||..||...|........|..|.......|..||..||..|..||...........|..|...||........|........|..|..|||.||...............||..||..|...|.........**

**Oceanobacillus iheyensis (Firmicutes) gi|23098673|NP_692139 128aa**

**>Oceihe2**

**....MEQMEHRTIYEAIGG.....YRTMNNIVHAFYTRVEVH.PDLKPI...F.......PDDLTETIRRQMMFLTQFFGGP.LYEEERGHPMLRRRHLPFP.....I....TPTRRDAWLTCMAAALEEAEIKEP...YRTAMMERLTLTANHMMNTPE.....**

**.........|..||..|..............|..||..||...|........|..|.......|..||..||..|..||...........|..|...||........|........|..|..|||.||...............||..||..|...|.........**

**Parachlamydia sp. UWE25 (Chlamydiae) gi|46446613|YP_007978.1 146aa**

**>Parach**

**sgppqgitpdsriyslmg......edniykmledfyleleks.sirhl....fp.......ADmkaaskksaaffvficggpplyqqqfgsprmrqrhlpfa.....i....deearqvwlhsfkhilqgaeqkyc...fpmeymgsfyrfldqfstwmintqse**

**.........|..||..|..............|..||..||...|........|..|.......|..||..||..|..||...........|..|...||........|........|..|..|||.||...............||..||..|...|.........**

**Photobacterium profumdum (Gammaproteobacteria) gi|46913965|CAG20747 160aa**

**>Phopro**

**qkttpvygmgdatllaagg.....eegvkklvdafydymdtl.peaevvra.mhkp......dltdsreklttfltgwmggpsryaekygsmnipgahrhls.....i....giaekeawllcmqkalddqgydd.....vfkryvmvqlsfpaemcrnrs....**

**.........|..||..|..............|..||..||...|........|..|.......|..||..||..|..||...........|..|...||........|........|..|..|||.||...............||..||..|...|.........**

**Polaromonas sp. JS666 (Betaproteobacteria) gi|54032073|ZP_00364205 146aa TrHb2**

**>Polaro**

**eenppgnppfetpfawigg.....eervkalverfydlmdle.pg.......yaalraahgstldnarqrlfwflcgwlggpqhyterfghprlrmrhmpqqtgggai.gsigilerdqwlacmdqamretgvdd....alrtrlnasffqtadwmrnrge....**

**.........|..||..|..............|..||..||...|........|..|.......|..||..||..|..||...........|..|...||........|........|..|..|||.||...............||..||..|...|.........**

**Ralstonia eutropha (Betaproteobacteria) gi|46132529|ZP_00202928 139aa**

**>Raleut**

**pestgqagtevtafdlvgg.....earvrelvdrfydlmdle.pe.......faglralhpasldgsrdklfwflcgwlggpnhfierfghprlrarhmpfe.....i....giserdqwmrcmalamqdvglsed....lqmrlmqaffqtadwmrnvar....**

**.........|..||..|..............|..||..||...|........|..|.......|..||..||..|..||...........|..|...||........|........|..|..|||.||...............||..||..|...|.........**

**Ralstonia metallidurans (Betaproteobacteria) gi|22977370|ZP_00023178**

**>Ralmet**

**snqpeeqreqitayeligg.....earvrelvdrfydlmdle.te.......faglralhppslegsrdklfwflcgwlggpNyfierfghprlrarhlpfe.....i....gtserdqwmrcmalamqdvglsed....lqmrlmqalfqtadwmrnvqr....**

**.........|..||..|..............|..||..||...|........|..|.......|..||..||..|..||...........|..|...||........|........|..|..|||.||...............||..||..|...|.........**

**Ralstonia solanacearum (Betaproteobacteria) gi|17546809|NP_520211 TrHb2**

**>Ralsol**

**.MTDAASGQETLAFDLLGG.....EARVRELADRFYDLMDLE.PA.......FA..RALHPPSLDGSRDKLFWF..HYI.......ERFGHPRLRARHLPFE.....I....GTRERDQWMRCMALAMQDLGIDDA....LQMRLMQAFWQTADWMRNVPR....**

**Rhodoferax ferrireducens (Betaproteobacteria) 134aa EAO42563.1 GI:72606580**

**.mniekkpvadtpfdqigg....eakvqalverfydlmdre.ps........yravraahgnvlddarqrlfwflcgwlggpqhyaerfghprlrrrhlpfk.....i....gtlerdqwlacmdqamgetgvpda....lrsqlreaffktadlmrnphd....**

**.........|..||..|.............|..||..||...|.........|..|.......|..||..||..|..||...........|..|...||........|........|..|..|||.||...............||..||..|...|.........**

**Rubrivivax gelatinosus (Betaproteobacteria) gi|47573541|ZP_00243579 136aa**

**>Rubgel**

**..tppestapatpyewvgg.....ehavralvdrfydlmdle.pg.......fhdlrrlhpstldgsrdklfwflcgwlggpdlyverfghprlrarhlpys.....i....gvrerdqwmacmeqamheceldp....llvsrlkasfantadwmrnrgt....**

**Shewanella amazonensis (Gammaproteobacteria)143 aa ZP_00588023.1 GI:68548528**

**qpqddrdpsqsnayemigg.....detiraiakqfyarmqqge.dtqklla.mhp......gpieqseqklyeflsgwlggpqlfqqkyghpalrarhlpfa.....i....deqmrdqwltcmqyaleqevane....qhreviyqaiatladhmrnq......**

**Shewanella baltica (Gammaproteobacteria) 145aa ZP_00582742.1 GI:68543024**

**ppqddrdpsqsnaydligg.....ekviraitkcfyqkmass.aetttlla.ihra......piaeseqklfeflsgwlggp.......qlyqqkyghpalrarhmhv..dideamrdqwlfcmkfaieenikkp....ehraaifeaistladhmrnq......**

**Shewanella denitrificans (Gammaproteobacteria) 156aa ZP_00636272.1 GI:69945085**

**kvdddrdvsqsnaydligg.....dkvikaiahefyqqmqtre.dtqalla.ihka......piaeseqklyeflsgwlggp.......qlyqrkhgnpalrarhmpf..aidesmrdqwltcmqaaiethikkp....ehrqaiiqaistladhmrnq......**

**Shewanella frigidimarina (Gammaproteobacteria) 144aa ZP_00640540.1 GI:69953395**

**qavddrdtaqsnaydligg.....dkviraiahdfyqqmqtr.petqalla.ihr......spmqeseqklyeylsgwlggp.......qlyqqkhghpalrarhmpf..aidesmrdqwlicmraaiqnnikkp....ehqqviiqaistladtmrnc......**

**.........|..||..|..............|..||..||...|........|..|.......|..||..||..|..||...........|..|...||........|........|..|..|||.||...............||..||..|...|.........**

**Shewanella oneidensis (Gammaproteobacteria) gi|24371639|NP_715681 145aa**

**>Sheone**

**KVQDDRDPNQSNAYDLIGG.....DKVIRAIANSFYQKMASSE.ETRAL...FA....IHRAPIAESEQKLYEFLTGWLGGP..QKYGHPALRARHMHFA.......V....DEAMRDQWLFCMKFAIEKHIKKP....EHRAAIYEAISTLADHMRNQ......**

**.........|..||..|..............|..||..||...|........|..|.......|..||..||..|..||...........|..|...||........|........|..|..|||.||...............||..||..|...|.........**

**Sinorhizobium meliloti (Alphaproteobacteria) gi|15966172|NP_386525 130aa**

**>Sinmel1**

**....MTEPQTTTLYEAIGG.....DATVRALTQRFYELMDSL.PEAARCRA.VHPP......DLTGSEEKFYEYLTGWLGGP..YVQKRGHPMLRRRHFIAG.....I....GPAERDEWLFCFTRALEETVSHPK....LREIILEPITRLAHHMQNKE.....**

**.........|..||..|..............|..||..||...|........|..|.......|..||..||..|..||...........|..|...||........|........|..|..|||.||...............||..||..|...|.........**

**Staphylococcus aureus (Firmicutes) gi|15923993|NP_371527 121aa**

**>Staaur**

**.........MTTTPYDIIG.....KEALYDMIDYFYTLVEKDE.RLNHL...FP.......GDFAETSRKQKQFLTQFLGGP..YTEEHGHPMLRKRHMDFT.....I....TEFERDAWLENMQTAINRAAFPQG...VGDYLFERLRLTANHMVNS.......**

**.........|..||..|..............|..||..||...|........|..|.......|..||..||..|..||...........|..|...||........|........|..|..|||.||...............||..||..|...|.........**

**Staphylococcus epidermidis (Firmicutes) gi|27467610|NP_764247 121aa**

**>Staepi**

**.........MSKTPYELIG.....QKALYQMIDHFYQLVEKDS.RINHL...FP.......GDFKETSRKQKQFLTQFLGGPDLYTQEHGHPMLKRRHMEFT.....I....SEYERDAWLENMHTAIQHAKLPAG...VGDYLFERLRLTANHMVNS.......**

**Staphylococcus haemolyticus (Firmicutes) 121aa YP_253870.1 GI:70726956**

**........msktpydiige......talynmidhfyalvekdd.rinhl...fpg.......dfaetsrkqkqfltqflggpnlyteehghpmlkkrhmefk.....i....ttyerdawlenmhtaiteaqfpag...vgeylyerlrltanhmvns.......**

**.........|..||..|..............|..||..||...|........|..|.......|..||..||..|..||...........|..|...||........|........|..|..|||.||...............||..||..|...|.........**

**Streptomyces avermitilis (Actinobacteria) gi|29831913|NP_826547 134aa**

**>Strave**

**NEIRRGTLQEQTFYEQVGG.....EETFRRLVHRFYEGVAGD.PLLKPM...YP......EEDLGPAEERFTLFLIQYWGGP..YSEQRGHPRLRMRHAPFA.....V....DRAAHDAWLKHMRVAVDELGLSE...EHEHTLWNYLTYAAASMVNTES.....**

**.........|..||..|..............|..||..||...|........|..|.......|..||..||..|..||...........|..|...||........|........|..|..|||.||...............||..||..|...|.........**

**Streptomyces coelicolor (Actinobacteria) gi|21221127|NP_626906 137aa**

**>Strcoe**

**NEIRRGTLQEQTFYEQVGG.....EETFRRLVHRFYEGVAED.PILRPM...YP......EEDLGPAEDRFALFLMQYWGGP..YSDNRGHPRLRMRHAPFA.....V....DRAAHDAWLKHMRVALDELGLSE...EHEQTLWKYLTYAAASMINTPG....,**

**.........|..||..|..............|..||..||...|........|..|.......|..||..||..|..||...........|..|...||........|........|..|..|||.||...................||..||..|...|.........**

**Thermobifida fusca (Actinobacteria) gi|48835434|ZP_00292434 163aa**

**>Thefusc**

**..mtsqhddqmtfyeavgg.....eetftrlarrfyegvaad.pvlrpm...yp......Eedlgpaeerlrlflmqywggp..yserrghprlrmrhfpyr.....i....gaeerdrwlthmraavddlalpa...hleqqlweylvyaayamvnvpedaqpp**

**.........|..||..|..............|..||..||...|........|..|.......|..||..||..|..||...........|..|...||........|........|..|..|||.||...............||..||..|...|.........**

**Thiobacillus denitrificans (Betaproteobacteria) gi|52006448|ZP_00333828 126aa**

**>Thiden**

**.........mqthydrlgg.....adtirrlvdrfydlmded.pdy......yg.irklhptdltesrnklawflsgwtggppeytdrfghpflrrrhlpfv.....i....geserdqwmgcmiramqdvglda...alqqeltaalfqtadfmrnqpq.....**

**.........|..||..|..............|..||..||...|........|..|.......|..||..||..|..||...........|..|...||........|........|..|..|||.||...............||..||..|...|.........**

**Verrumicrobium spinosum (Chlamydiae/Verrummicrobia) gnl|TIGR_240016|contig:335 121aa**

**>Verspi**

**........MVDTFYTRLGE......EALTRLVAAFYRRVKADD.LLGPM...YPP......EDWEGAERRLRDFLIFRFGGPDQYIQERGHPRLRMRHAPFK.....I....GVAERDRWLDLMGAAMKEVGIPE.......DLAPTLGGFFAQVADFMRN....**

**.........|..||..|..............|..||..||...|........|..|.......|..||..||..|..||...........|..|...||........|........|..|..|||.||...............||..||..|...|.........**

**2/2Hb3s**

**Acidithiobacillus ferrooxidans (Gammaproteobacteria) TIGR_243159|contig:10428:a 132aa**

**>Acifer3**

**...MEQVSPPKRRLEPLCEK..ITLPVIRAVVDDFYNRIQHH.PTLAEP...FSIV.....QDWELHKDRLVHYWWTVSGGL...PYKDYRYALGDKHAPLG.....I....THSLVDDWLALFHETMLDHMDAD.....MARRWHGMAAGIGESLRLMFAPR......**

**.........|..||..|..............|..||..||...|........|..|.......|..||..||..|..||...........|..|...||........|........|..|..|||.||...................||..||..|...|.........**

**Agrobacterium tumefaciens (Alphaproteobacteria) gi|15887600|NP_353281 157aa (9-150)**

**>Agrtum2**

**VHNAEIQDRAEKAMAEIGV....DAAFIDLLVETFYGRVLEHQTLGPV....FD......AGRWPEHMARMKQFWTAIAFKN.....GSYGGKPVQAHLG.......V.QGMSAELFPQWLALFSVTLDDIAPSRKAHDW....FMETAERIARSLTLSLFYNPAMDDP**

**.........|..||..|..............|..||..||...|........|..|.......|..||..||..|..||...........|..|...||........|........|..|..|||.||...................||..||..|...|.........**

**Agrobacterium tumefaciens (Alphaproteobacteria) gi|17934166|NP_530956 135aa**

**>Agrtum3**

**.............maeigv....daafidllvetfygrvlehqtlgpv....fdarl...agrwpehmarmkqfwtaiafkn.....gsyggkpvqahlg.......v.qgmsaelFpqwlalfsvtlddiapsrkahdw....fmetaeriarsltlslfynpamddp**

**.........|..||..|..............|..||..||...|........|..|.......|..||..||..|..||...........|..|...||........|........|..|..|||.||...................||..||..|...|.........**

**Bdellovibrio bacteriovorus (Deltaproteobacteria) gi|42523942|NP_969322 128aa**

**>Bdebac2**

**.......mttptsrkples.....redvmvlvdsfyakvrad.gyigpi...ftDV...akvdweehlpklynfwsdlllge.....dsyrgrpfpphtkln.....l....erghfeqwlrlfvetvdehfvglkanea.....kerayriarnfminlqllsle...**

**.........|..||..|..............|..||..||...|........|..|.......|..||..||..|..||...........|..|...||........|........|..|..|||.||...................||..||..|...|.........**

**Bordetella pertussis** **(Betaproteobacteria) gi|33593122|ref|NP_880766 134aa**

**>Borper2**

**..........MGAPDLCTE......EDITQLVHQFYAEVRRD.AELGPI...FN....THVDDWDVHLAKLVDFWSSILRGT.RGTPMPRHVALPGLHAG.......L........FERWLRLFRATAAAQPNQAMAEQA.....CLMAGRIAQSLWYGYQLHRSPDQA**

**.........|..||..|..............|..||..||...|........|..|.......|..||..||..|..||...........|..|...||........|........|..|..|||.||...................||..||..|...|.........**

**Bradyrhizobium japonicum (Alphaproteobacteria)(gi|27381333|NP_772862 154aa (11-154)**

**>Brajap2**

**AAAARRNLLTQDAIERTGI....TEEMIAELVTRFYGRVRED.ALLGPV...FAV.....VQNWDEHLAKLGDFWSSVVLMSS.PMRAHLPLGLVGDHFDRW.....L......DLFEQTARDVCPPAAAALFIDKA.....RRIADSFEMASATVAGRIASPRHVLRS**

**.........|..||..|..............|..||..||...|........|..|.......|..||..||..|..||...........|..|...||........|........|..|..|||.||...................||..||..|...|.........**

**Brucella abortus (Alphaproteobacteria; Rhizobiales) 141aa YP_223127.1 GI:62317274**

**.........mtilinqphp..sidrdsidrlveifygrarede.iigpi...fnr....tvkdwdhhlarisefwssvilktgrpmpphlalnlenehfdlw.....l......elfeqtaqeifppeaaiifvdra.....rriadsfemaiathsgriraprhsrlp**

**Brucella melitensis (Alphaproteobacteria) gi|17988743|NP_541376 141aa**

**>Brumel3**

**.........MTILINQPHP..SIDRDSIDRLVEIFYGRAREDE.IIGPI...FNR....TVKDWDHHLARISEFWSSVILKTR.PMPPHLALNLENEHFDRW.....L......ELFEQTAREIFPPEAAIIFVDRA.....RRIADSFEMAIATHSGRIRAPRHSRRP**

**Burkholderia ambifaria (Betaproteobacteria) 222aa EAO44088.1 GI:72608134 (34)**

**famtalpaspdtaparprd.aeptednirdlvyafydrvrad.pllgpv...fd.a..klegrwddhlpkmvsfwsslvlgtkvqqahqpldgiepahfsrw.....l......slflktvegrytppaairfmepa.....lriaqslqlsrfgwdyqippeqqalld**

**.........|..||..|..............|..||..||...|........|..|.......|..||..||..|..||...........|..|...||........|........|..|..|||.||...................||..||..|...|.........**

**Burkholderia cepacia (Betaproteobacteria) gi|46315472|ZP_00216054 186aa (1-155)**

**>Burcep3**

**..mtalpaspdsaparprd.tepteenirdlvyafydrvrad.pllgpv...fd.a..Klegrwddhlpkmvtfwsslvlgtk.....gyrgnvqqahqpldg....i....epahfsrwlslflktvetryappaa.....irfmepalriaqslqlsrfgwdyqipp**

**.........|..||..|..............|..||..||...|........|..|.......|..||..||..|..||...........|..|...||........|........|..|..|||.||...................||..||..|...|.........**

**Burkholderia cepacia (Betaproteobacteria) gi|46323547|ZP_00223911 190aa (1-155)**

**>Burcep4**

**..mtslpassdtaparird.aepteanirdlvyafydrvrad.pllgpv...fd.a..kldgrwdthlpkmvsfwsslvlgtr.....gyrgnvqqahqpldg....i....epahfsrwlslflktvearytpaa.....avrfmepalriaqslqlsrfgwdyripa**

**.........|..||..|..............|..||..||...|........|..|.......|..||..||..|..||...........|..|...||........|........|..|..|||.||...................||..||..|...|.........**

**Burkolderia fungorum (Betaproteobacteria) gi:48783812|ZP_00280193 181aa (1-154)**

**>Burfun3**

**...mnpsfpaapaveraahp...teanirdlvyafyervrad.allgpv...fdat...ladrwddhlpkmctfwgslvlga.vqqahqplegiepqhfsrw.....l......ylfldtvqsryepaaavr............fmePAlriaqslqlsrfgwdykipe**

**.........|..||..|..............|..||..||...|........|..|.......|..||..||..|..||...........|..|...||........|........|..|..|||.||...................||..||..|...|.........**

**Burkholderia pseudomallei (Betaproteobacteria) gi|53719116|YP_108102 180aa (36-170)**

**>Burpse2**

**tqafsassaaakpardaep....Tpeniralvdafydrvrdd.allgpv...fe...sklagrwdqhlpkmgefwsslvlgt.....ksyrgnvqaahqpldg....l....epvhfsrwlalflktvearyepaa.....avrfmepalriaqslqlsrfgwdyr...**

**.........|..||..|..............|..||..||...|........|..|.......|..||..||..|..||...........|..|...||........|........|..|..|||.||...................||..||..|...|.........**

**Burkholderia vietnamiensis 190aa EAM31672.1 GI:67534934 2/2Hb3**

**tslpassdtaparirdaep....teanirdlvyafydrvrad.pllgpv...fd.a..kldgrwdthlpkmvsfwsslvlgt.....rgyrgnvqqahqpldg....i....epahfsrwlslflktvearytpaa.....avrfmepalriaqslqlsrfgwdyripa**

**.........|..||..|..............|..||..||...|........|..|.......|..||..||..|..||...........|..|...||........|........|..|..|||.||...................||..||..|...|.........**

**Campylobacter coli 150 aa ZP_00367734.1 GI:57168601**

**ltinfypkantmkyetinq......esiaklmelfydkirkdk.dlgpi...fnnaigtsdeawkehkakignfwagmllge.....gdyigqplkkhldlpp....f....pqeffgiwlglfeesldkiynnee....mknvilqraqmiashfqnmlyrfggh...**

**.........|..||..|..............|..||..||...|........|..|.......|..||..||..|..||...........|..|...||........|........|..|..|||.||...................||..||..|...|.........**

**Campylobacter jejuni (Epsilonproteobacteria) gi|15791829|NP_281652**

**>Camjej**

**...........MKFETINQ......ESIAKLMEIFYEKVRKDK.DLGPI...FNNAIGTSDEEWKEHKAKIGNFWAGMLLGE.....GDYNGQPLKKHLD.......L.PPFPQEFFEIWLKLFEESLNIVYNEE.....MKNVILQRAQMIASHFQNMLYKYGGH...**

**.........|..||..|..............|..||..||...|........|..|.......|..||..||..|..||...........|..|...||........|........|..|..|||.||...................||..||..|...|.........**

**Campylobacter lari (Epsilonproteobacteria; Campylobacterales) 127aa ZP_00368630.1 GI:57240682**

**...........mkfetinh......egikklmdvfyakvradk.dlgpi...fnekigtddeswkkhkekiasfwagmflad.....psysgsplrahhe.......l.ppfpreffdiwlnlfdeslqevfede.....prdiiierarmiaqrfqmiiydhrfa...**

**.........|..||..|..............|..||..||...|........|..|.......|..||..||..|..||...........|..|...||........|........|..|..|||.||...................||..||..|...|.........**

**Campylobacter upsaliensis (Epsilonproteobacteria; Campylobacterales) 129aa ZP_00370192.1 GI:57242253**

**...........mkfeaisi......esihklmdifyakvrvdksglgei...fnnkigtndeqwnshkikiasfwegmllgs.....gnfkgnpmkahid.......l.pafprelfavwlklfkeslecvyke....pehqrvifqkaemiaqrfqhvlyesdvr...**

**.........|..||..|..............|..||..||...|........|..|.......|..||..||..|..||...........|..|...||........|........|..|..|||.||...................||..||..|...|.........**

**Caulobacter crescentus (Alphaproteobacteria) gi|16127286|NP_421850**

**>Caucre**

**.....MSLSPRSQARADAA..PFTDEEIDRLVETFYARIRQHH.RLGPI...FEAA..IGPDGWPEHLAKLKDFWSSVLNTS.....GRYKGQPVAAHQGVEG....V....TEGLFTPWLGLFHMTCAELFDAP.....RAAIIGQKAERIAATLKLGLFHRPGAVA.**

**.........|..||..|..............|..||..||...|........|..|.......|..||..||..|..||...........|..|...||........|........|..|..|||.||...................||..||..|...|.........**

**Deinococcus geothermalis (Deinococcus-Thermus) 135aa ZP_00397127.1 GI:66798373 2/2Hb3**

**..mtlplthpdalfarlgd......etlrrvlwafyakatrde.llgpv...ftrrigpfprggwpmhimrlegfwravtggp....sayrgqpgpahadlg.....i....apehfdrwlalweetlmefldpp.....eaaalltlarrmrgtlerhaarepv....**

**.........|..||..|..............|..||..||...|........|..|.......|..||..||..|..||...........|..|...||........|........|..|..|||.||...................||..||..|...|.........**

**Deinococcus geothermalis (Deinococcus-Thermus) 274aa ZP_00396100.1 GI:66797340 (139-274) C-terminal 2/2Hb3 at ~150aa**

**rlmqaeprapgsplevvgg.....eaalrtmladfyararad.tllgpv...fa.a...hvqdweahldrvtafwvtmlgggp.....awrgnlnsvhaglg.....l....rgthlrrwlalfreaaedclgpeaa.....apltaraeamghrlgqrnaphvgrvp**

**.........|..||..|..............|..||..||...|........|..|.......|..||..||..|..||...........|..|...||........|........|..|..|||.||...................||..||..|...|.........**

**Desulfitobacterium hafniense (Firmicutes) gi|23119438|ZP_00102524**

**>Deshaf**

**.......mfkrgamrsshd...ldeaalaalvrrfyaavredd.rlgpv...fa....aaiddwdaherrlidfwtslmlgs..Gryrgnpaqmhlrhagr......i....qpelfahwlalwrrcsehtlppaqa.....aavqaragriaehlqrllaqa......**

**.........|..||..|..............|..||..||...|........|..|.......|..||..||..|..||...........|..|...||........|........|..|..|||.||...................||..||..|...|.........**

**Helicobacter hepaticus (Epsilonproteobacteria) gi|32261711|gb|AAP76761**

**>Helhep**

**.......MTFEKINVDSIR..........KLMDIFYAKVRADKSGLGDI...FNTKIGTSDEVWEVHKAKIANFWQGMLLNS.....GDYNGQPLKAHLD.......L.PPFPRELFNVWLNLFEESLRAVYAKEE....HISLILQRAQMIAQRFQYIIYESGLHH..**

**.........|..||..|..............|..||..||...|........|..|.......|..||..||..|..||...........|..|...||........|........|..|..|||.||...................||..||..|...|.........**

**Hyphomonas neptunium gnl|TIGR_228405|contig:1064:h_neptunium sequence 1:**

**..............MGIDE......AYISTLVDTFYTRIRAH.PVLGPV...FGAA....ITDWTPHLETMKDFWSSVALGT.....GRYSGRPVPAHAKHRH....I....QQFHFNLWLALFEQTLRDTAKTPEA....IPFFLDRANRIAQSLQYALFGMDGLPLS**

**.........|..||..|..............|..||..||...|........|..|.......|..||..||..|..||...........|..|...||........|........|..|..|||.||...................||..||..|...|.........**

**Mesorhizobium sp. BNC1 (Alphaproteobacteria) gi|45680999|ZP_00192444 154aa**

**>Mesorhi**

**.mskqsagrttiivdgvplpeildermihdvvhgfyeeirndd.llgpi...fnga..inseawphhlakmcdfwsatllrt.....kryegrplpphla.......i.pglgevhfrrwlmlfratvdrlcpadva.....vlfmdralriahsfrlavafsrgddtl**

**.........|..||..|..............|..||..||...|........|..|.......|..||..||..|..||...........|..|...||........|........|..|..|||.||...................||..||..|...|.........**

**Methylococcus capsulatus (Gammaproteobacteria) gi|53804116|YP_114018 140aa**

**>Metcap3**

**....MATHCTSSMSTHLPT.....EEQIAEMVRRFYRQVLADD.RLRPI...FD....AAITDWDTHHRVVQDFWSRTLLDT.....DRYRGHPYPVHAQLP.....L....QPEHFDIWLELFRKAAREVLPADSAA.....RAIARAEHMAESFKAGMFSFPRYEGP**

**.........|..||..|..............|..||..||...|........|..|.......|..||..||..|..||...........|..|...||........|........|..|..|||.||...................||..||..|...|.........**

**Mycobacterium avium (Actinobacteria) gi|41409276|NP_962112 154aa (HbP)**

**>Mycavi3**

###### .......mvsgltmpdlrd.....radveallrrfygraldde.vlaep...farl...ratglddhvptmcdfwetvlfra.....gryrgsalqahrdihrrtp.l....sdrhfrrwltlwhntvdemyrgpaad.....rakiqaariawamhrrltgadspell

**.........|..||..|..............|..||..||...|........|..|.......|..||..||..|..||...........|..|...||........|........|..|..|||.||...................||..||..|...|.........**

**Nitrosomonas europaea (Betaproteobacteria) gi|30249138|NP_841208 141aa**

**>Niteur3**

**...MIPRPIPEPSPDLYTE......EEVSKLVHEFYAKARKD.AALGPI...FEEH....VIDWDAHFVQMTNFWSAQLRGT.....SRFRGAPMPKHIALPE....L....NETLFKRWLQLFRQTTLELGNPLL.....KQHADTVAEFIAGRLWMGYQMSHFPHRE**

**.........|..||..|..............|..||..||...|........|..|.......|..||..||..|..||...........|..|...||........|........|..|..|||.||...................||..||..|...|.........**

**Novosphingobium aromaticivorans (Alphaproteobacteria) gi|48849793|ZP_00303907 127aa**

**>Novaro**

**............MNDLSITE.....GDIDRLVPEFYARVRKDE.VLGPI...FNGA....IEDWPDHLERLKAFWSSIMLTS.....GRYKGQPLIAHVKHEAH...M....TPENFARWLELWRQTTGDILGPD.....kaelfrqkaariaesltmgvqfyrerqra**

**Paracoccus denitrificans (Alphaproteobacteria)140 aa ZP_00632465.1 GI:69937879**

**hirrmipprfdvtpeqidr...........vvatfyaairrhe.vlgpv...fag....hvadwpeheakiarfwrnailye.....rgydgnpmqvhmragd....v....kaqhfapwlmlfdetlrrtlpae.........tarawsalahrigaglrmgvedlre**

**.........|..||..|..............|..||..||...|........|..|.......|..||..||..|..||...........|..|...||........|........|..|..|||.||...................||..||..|...|.........**

**Rhodopseudomonas palustris (Alphaproteobacteria) gi|39935786|NP_948062 139aa**

**>Rhopal**

**........mayivamslahsDILNDDQIAQLVRTFYGRAREDE.VIGPI...FASA....VHDWDHHIAQITDFWSSMLLKT..PMRPHLMLNLAPEHFDRW.....L......KLFEATATELFTPEVAT............EFIIRARRIAdsfemgiattqgkvat**

**.........|..||..|..............|..||..||...|........|..|.......|..||..||..|..||...........|..|...||........|........|..|..|||.||...................||..||..|...|.........**

**Silicobacter pomeroyi (Alphaproteobacteria) TrHb3**

**>Silpom3**

**...........ITPAQIDH...........VVRVFYARIRTH.AELGPV...FAAH....VRDWPAHEARIAAFWRNAILKE..MRVHVSRPDIRPDHFP.......L.......WLALFHEVLTQELAAETAA.........RWGALADRIGHGLKMGL.........**

**.........|..||..|..............|..||..||...|........|..|.......|..||..||..|..||...........|..|...||........|........|..|..|||.||...................||..||..|...|.........**

**Silicibacter sp. TM1040 (Alphaproteobacteria) gi|52009563|ZP_00336926 137aa**

**>Silici**

**......mqdvqplarfqisa.....ddirrvvsvfygevrrh.pvlapv...fna....hvedwpqheekianfwrnailrer.....vydgfpmrehlsrpd....v....raehfpvwlglfhavlerevtae.....qalqwghladrigegfrtgiesirq....**

**.........|..||..|..............|..||..||...|........|..|.......|..||..||..|..||...........|..|...||........|........|..|..|||.||...................||..||..|...|.........**

**Sinorhizobium meliloti (Alphaproteobacteria) gi|15966331|NP_386684 155aa**

**>Sinmel2**

**VQSAAMRERAEAEMKALGI....DEAFIGKLVDTFYARVLAH.PELGPV...FDA...RLSGRWPEHMEKMKSFWSAVAFRS.....GAYGGKPVQAHLG.......V.ANMSPELFPKWLGLFAATLDDIAPNEE....AKAWFMATAERIARSLTLSLFYNP.....**

**.........|..||..|..............|..||..||...|........|..|.......|..||..||..|..||...........|..|...||........|........|..|..|||.||...................||..||..|...|.........**

**Sphingopyxis alaskensis (Alphaproteobacteria; Sphingomonadales) gi|68539244|ZP_00579017.1 161aa 2/2Hb3**

**hphaaaaraarradakvig...ideafiaafvddfyaairada.llgpv...faa....riddwpahlaqmnrfwqsillaa.....gsfsgnpmvkhla.......i.pglddahfahwldlfyatlhrlaptpe....aaklvgekarmiaeslltgirlhrdrdad**

**.........|..||..|..............|..||..||...|........|..|.......|..||..||..|..||...........|..|...||........|........|..|..|||.||...................||..||..|...|.........**

**Fig. 2**

**Caenorhabditis elegans/briggsae**

**-------------------------------------------------------------------------------------C--------------------------D------------------------------------------------------P------------F------------------------------------------------------------------------------------------------------**

**--aaaaaaaAaaAAaaAa-----------------bbbbbBbbBBbbBBbbcCccccc---------------------------D-------------dddddddeeeEeeEEeeEEeeEeeEEe----------------------------------FffFfffFFfggg-------G----------ggggGggGggGGGgGGggg------------------------------------------hhhhhhHHhhHHhhHhhhHhhhhhhh-----**

**---------|--||--|-----------------------|--||--||---|--------------------------------|--|--------------------|--||--||--|--||-----------------------------------|--|---||-----------|--------------|--|--|||-||---------------------------------------------------||--||--|---|------------**

**MYWHP**

**vlsegewqlvlhvwakvea----------------dvaghgqdilirlfkshpetlek---------------------------fdrf------khlkteaemkasedlkkhgvtvltalgailkkk--------------------------ghheaelkplaqshatkhk-------i----------pikylefiseaiihvlhsrhpgdf-------------------------------------gadaqgamnkalelfrkdiaakykelggyqg**

**(1)**

***CE36418/ZK637.13 gi|42734289|CAA77458 159aa Does not cluster with other C. elegans globins**

**-MSMNRQEISDLCVKSLEG---------RMVGTEAQNIENGNAFFRYFFTNFPDLRVY---------------------------FKGA-------EKYTADDVKKSERFDKQGQRILLACHLLANVY-----------------------TNEEVFKGYVRETINRHRIYK--------M----------DPALWMAFFTVFTGYLESVGC---------------------------------------LNDQQKAAWMALGKEFNAESQTHLKNSNLPHV**

**---------|--||--|-----------------------|--||--||---|--------------------------------|--|--------------------|--||--||--|--||-----------------------------------|--|---||-----------|--------------|--|--|||-||---------------------------------------------------||--||--|---|------------**

**CBP07494/CBG0687 gi|39585062|CAE62713 160aa**

**-MSMTRQEIQDLCVKSLEE---------KMVGTTDKGIANGNGFYQYFFTNFPDLRVY---------------------------FKGA-------EKFTAEDVKKSERFDKQGQRILLACHLIANVF-----------------------TNEEVFKAYVRETINRHRIYK--------M----------DPALWMAFFTVFTGYLESTGS---------------------------------------LNDQQKAAWMALGKEFNAECQVHLKNSNLPYV**

**---------|--||--|-----------------------|--||--||---|--------------------------------|--|--------------------|--||--||--|--||-----------------------------------|--|---||-----------|--------------|--|--|||-||---------------------------------------------------||--||--|---|------------**

**(2)**

**CE01012/F49E2.4 gi|3877381|CAA86423 216aa (13-205)**

**EITDEEVTAIRDVWRRAKT------------------DNVGKKILQTLIEKRPKFAEY---------------------------FG-I-----QSESLDIRALNQSKEFHLQAHRIQNFLDTAVGSL---------------------GFCPISSVFDMAHRIGQIHFYRG--------V--------NFGADNWLVFKKVTVDQVTTGTTDSSKEKE-D-TNSNGTANGKVDTDASLIPIADINNVYSGENCLARLGWNKLMTVIVREMKRGFLEEAMRNC**

**---------|--||--|-----------------------|--||--||---|--------------------------------|--|--------------------|--||--||--|--||-----------------------------------|--|---||-----------|--------------|--|--|||-||---------------------------------------------------||--||--|---|------------**

**CBP03870/CBG16082 gi|39596154|CAE6979 218aa (13-207)**

**EMSDEEVSAIREVWIRAKT------------------DNVGKKILQTLIEKRPKFAEY---------------------------FG-I-----QSESLDIRALNQSKEFHLQAHRIQNFLDTAVGSL---------------------GFCPISSVYDMAHRIGQIHFYRG--------V--------NFGADNWLVFKKVTVDQVTTGATDSSKEKDKDETNSNGTANGKVDTEANPIPIADINNVYSGENCLARLGWNKLMTVIVREMKRGFLEEAMRNC**

**---------|--||--|-----------------------|--||--||---|--------------------------------|--|--------------------|--||--||--|--||-----------------------------------|--|---||-----------|--------------|--|--|||-||---------------------------------------------------||--||--|---|------------**

**(3)**

**CE01528/C52A11.2 gi|3875223|CAA8676 266aa (31-215)**

**ALNKKDRTLLRETWQRLDD----------------PKDIVGLIFLDIVNDIEPDLKKV---------------------------FG-V-------DRAPRAAMLKMPKFGGHILRFYEFMEQLTSML--------------------GTSENLTGAWQLVRKTGRSHVRQGFLEQNQNQM------EKNYFEIVINVFIERLIPFLTGEQE---------------------LPSSEGKENKKVRFAQNYTTSQITDVWKKFLNTVISQMTDSFELERAKQK**

**---------|--||--|-----------------------|--||--||---|--------------------------------|--|--------------------|--||--||--|--||-----------------------------------|--|---||-----------|--------------|--|--|||-||---------------------------------------------------||--||--|---|------------**

**CBP00622/CBG03023 gi|39597385|CAE59614 266aa (31-215)**

**NLNKKDRTLLRETWQRLEE----------------PKDIVGLIFLDIVNDIEPDLKKV---------------------------FG-V-------DRAPRAAMLKMPKFGGHILRFYEFMEQLTSML--------------------GTSENLTGAWQMVRKTGRSHVKQGFLEQNQNQM------EKNYFEVVINVFIERLIPYLTGEQE---------------------LPPAEGKEQKKVRFAQNYTTSQIADVWKKFLNIVISQMTDSFELERAKQK**

**---------|--||--|-----------------------|--||--||---|--------------------------------|--|--------------------|--||--||--|--||-----------------------------------|--|---||-----------|--------------|--|--|||-||---------------------------------------------------||--||--|---|------------**

**(4)**

**CE01816/C28F5.2 247aa (52-211)**

**SLTFSQKQALNLSWRLLPQ-----------------ASACFRKIFLELEIASPKVKQIFYKAALVDA------------------FNK--------------DDDNSATMEVHIKLTTKFFDELLVSL-----------------------DDETEFVNKIRGIGSAHAILAKGSN----F----------SSDIWERLGEIAMERVCSHEV-------------------------------------VTKTREASRAWRTLIAILIDELRGGFEGERQHRK**

**---------|--||--|-----------------------|--||--||---|--------------------------------|--|--------------------|--||--||--|--||-----------------------------------|--|---||-----------|--------------|--|--|||-||---------------------------------------------------||--||--|---|------------**

**CBP09240/CBG13047 gi|39596023|CAE67526 244aa (48-207)**

**SLTFSQKQALNLSWRLLKP----------------QASACFRKIFLELEIASPKVKQIFYKAALVDA------------------FNK--------------DDDNTATLEVHIKLTTKFFDELLATL-----------------------DDENEFVAKIRGIGSAHAILAKGSN----F----------SSDIWERLGEIAMERVCSHEV-------------------------------------VTKTREASRAWRTLIAILIDELRGGFEGELRQHR**

**---------|--||--|-----------------------|--||--||---|--------------------------------|--|--------------------|--||--||--|--||-----------------------------------|--|---||-----------|--------------|--|--|||-||---------------------------------------------------||--||--|---|------------**

**(5)**

**CE02371/W01C9.5 gi|3880381|CAA90269 224aa (23-178)**

**QLTPSQVSVVRRSWRHINT---------------KGLIIVLTRCFSRLESNCPIVSQC---------------------------FQSA-------TYSLSTNPNGVRTVADHAKYLLQLLDKIIEGD---------------------------VDSEFLREIGANHVCLKHESG----F----------STQEWDRFQEIMVEVILKQDG-------------------------------------VKQSKETSRAWRLLICSFIELIRDGFDAQVRQFR**

**---------|--||--|-----------------------|--||--||---|--------------------------------|--|--------------------|--||--||--|--||-----------------------------------|--|---||-----------|--------------|--|--|||-||---------------------------------------------------||--||--|---|------------**

**CBP00206/CBG00571 gi|39586983|CAE62918 224aa (23-178)**

**QLSPSQVSVIRRSWRHINT---------------KGLITVLTRCFSRLESNCPIASQC---------------------------FQSA TYSLSTNPCGVRTVADHAKYLLQLLDKIIEGD---------------------------VDSEFLREIGANHVGLQRDTG----F----------SSLEWDRFQEIMVEVILKQDG-------------------------------------VKQSKEATRAWRLLICSFIELIRDGFDAQVRQFR**

**---------|--||--|-----------------------|--||--||---|--------------------------------|--|--------------------|--||--||--|--||-----------------------------------|--|---||-----------|--------------|--|--|||-||---------------------------------------------------||--||--|---|------------**

**(6)**

**CE02514/C29F5.7 gi|17532151|NP_495268 198aa (27-187)**

**PLNAKTKKLVIQEWPRVLA----------------QCPELFTEIWHKSATRSTSIKLA---------------------------FGIA-----ENESPMQNAAFLGLSSTIQAFFYKLIITYELNDD---------------------------QVREACEQLGARHVDF---------I------SRGFNSHFWDIFLVCMAEKIDETLS--------------------------------AYIPDEDKRNEMILAWQRVINSIVHQMRNGYSDRRKQQL**

**---------|--||--|-----------------------|--||--||---|--------------------------------|--|--------------------|--||--||--|--||-----------------------------------|--|---||-----------|--------------|--|--|||-||---------------------------------------------------||--||--|---|------------**

**CBP00844/CBG02622 gi|39597062|CAE59289 198aa (27-187)**

**PLNAKTKKLVIQEWPRVLA----------------QCPELFTEIWHKSATRSTSIKLA---------------------------FGIA-----ENESPMQNAAFLGLSSTIQAFFYKLIITYELNDD---------------------------QVREACEQLGARHVDF---------I------SRGFNSHFWDIFLVCMAEKIDETLS--------------------------------SYMIEEDKKNEMILAWQRVVNCIVHQMRNGYSDRRKQQL**

**---------|--||--|-----------------------|--||--||---|--------------------------------|--|--------------------|--||--||--|--||-----------------------------------|--|---||-----------|--------------|--|--|||-||---------------------------------------------------||--||--|---|------------**

**(7)**

**CE03233/F19H6.2 gi|3876112|CAA92164 231aa (49-205)**

**FLTRRERILLEQSWRKTRK---------------TGADHIGSKIFFMVLTAQPDIKAI---------------------------FG-L-------EKIPTGRLKYDPRFRQHALVYTKTLDFVIRNL-----------------------DYPGKLEVYFENLGKRHVAMQGRG-----F----------EPGYWETFAECMTQAAVEWEA--------------------------------------NRQRPTLGAWRNLISCIISFMRRGFDEENGKKK**

**---------|--||--|-----------------------|--||--||---|--------------------------------|--|--------------------|--||--||--|--||-----------------------------------|--|---||-----------|--------------|--|--|||-||---------------------------------------------------||--||--|---|------------**

**CBP00055/CBG00138 gi|39588027|CAE57258 209aa (27-183)**

**FLTRRERILLEQSWRKTRK---------------TGADHIGSKIFFMVTAQ-PDIKAI---------------------------FG-L-------EKIPTGRLKYDPRFRQHALVYTKTLDFVIRNL-----------------------DYPGKLEVYFENLGKRHVAMQGRG-----F----------EPGYWETFAECMTQAAVEWEA--------------------------------------NRQRPTLGAWRNLISCIISFMRRGFDEENGKKK**

**---------|--||--|-----------------------|--||--||---|--------------------------------|--|--------------------|--||--||--|--||-----------------------------------|--|---||-----------|--------------|--|--|||-||---------------------------------------------------||--||--|---|------------**

**(8)**

**CE03523/R01E6.6 gi|3878772|CAA92187 311aa (95-241)**

**PISAQGREIITQCFENPHS-------------------EFANKVVQRIFEKR-EDYQKYIMN-----------------------L-------------GKERSSIVNNRLKQLVEDIVAHIHDADFI-----------------------------ESVSKQYGEEHVELKQYG-----F-------KPDFWVAVADAMTLEGVILDMANQ---------------------------------------HPADTVSAWSSLVTMIFSSVRDGYYSELRRHR**

**---------|--||--|-----------------------|--||--||---|--------------------------------|--|--------------------|--||--||--|--||-----------------------------------|--|---||-----------|--------------|--|--|||-||---------------------------------------------------||--||--|---|------------**

**CBP15914/CBG07422 gi|39596505|CAE6312 353aa (95-241)**

**PITSQCREIISQCFDNPHS-------------------EFANKVVQRIFEKR-EDYQKYIMN-----------------------L-------------GKERSVIVNNRLKILVEDIVAHIHDSDYI-----------------------------ELVSKQYGEEHVELKQYG-----F-------KPDFWVAVADAMTLEGVILDMANH---------------------------------------QPADTVSAWSSLVTLIFSSVRDGYYSELRRHR**

**---------|--||--|-----------------------|--||--||---|--------------------------------|--|--------------------|--||--||--|--||-----------------------------------|--|---||-----------|--------------|--|--|||-||---------------------------------------------------||--||--|---|------------**

**(9)**

**CE04582/F46C8.7 gi|17567747|NP_509275 322aa (60-222)**

**RLSKIQKRAIRFTWHRLQT-----------RNGGKRVENVFEEVFDKLVKNLPNIRDM---------------------------FST-------RMFLCAMSRGTTSTLRDHSKNCVKMIDSVIKNF-----------------DVEKSKRTDTSSENDPRVIGRAHSILKPYG-----L----------AGNYWEKFGEVMIDVVLAQEA-------------------------------------VRDLPGAGQAWVIFTACLVDQMRAGFDENRKTDH**

**---------|--||--|-----------------------|--||--||---|--------------------------------|--|--------------------|--||--||--|--||-----------------------------------|--|---||-----------|--------------|--|--|||-||---------------------------------------------------||--||--|---|------------**

**CBP03989/CBG16720 gi|39594123|CAE70233 314aa (61-223)**

**RLSKIQKRAIRFTWHRLQT-----------RNGGKRVENVFEEVFDKLVKNLPNIRDM---------------------------FST-------RMFLCAMSRGTTSTLRDHSKNCVKMVDSVIKNF-----------------DVEKSKRSDTGTENDPRVIGRAHSILKPYG-----L----------AGNYWEKFGEVMIDVVLAQEA-------------------------------------VRDLPGAGQAWVIFTACLVDQMRAGFDENRKTDH**

**---------|--||--|-----------------------|--||--||---|--------------------------------|--|--------------------|--||--||--|--||-----------------------------------|--|---||-----------|--------------|--|--|||-||---------------------------------------------------||--||--|---|------------**

**(10)**

**CE04843/R13A1.8 gi|1065933|AAA81477 342aa (160-326)**

**LIDKESCEVVADSWRLVES----------RSSAAETSACFGLFVFQRVFSKIPMLRPL---------------------------FG-L------SESDDVFDLPDNHPVRRHARLFTSILHISVKNV----------------------DELEAQVAPTVFKYGERHYRPD--------I------TPHMTEENVRVFCAQIVCTVFDFLR------------------------------------DTEATPKCAESWIELMRYLGQKLLDGFDFAKLTAE**

**---------|--||--|-----------------------|--||--||---|--------------------------------|--|--------------------|--||--||--|--||-----------------------------------|--|---||-----------|--------------|--|--|||-||---------------------------------------------------||--||--|---|------------**

**CBP01576/CBG05809 gi|39593545|CAE61837 342aa (160-326)**

**LIDKECCEIIGDSWRIVES----------RASSTFPTACFGLFVLRRVLQRIPILCPL---------------------------FS-L------SESDDIFKLPENHPVRRHARLFTNILHISVKNV----------------------DELEAQVAPTVFKYGERHYRPD--------I------TPHMTEENVRIFCAQIVCTVFDFLC------------------------------------ETEATPKCAESWIELMRYLGQKLLDGFDFAKLTAE**

**---------|--||--|-----------------------|--||--||---|--------------------------------|--|--------------------|--||--||--|--||-----------------------------------|--|---||-----------|--------------|--|--|||-||---------------------------------------------------||--||--|---|------------**

**(11)**

**CE05237/C06H2.5 gi|3874041|CAA99771 209aa (33-189)**

**HLSPHQVQLLTSTWPRIKT-----------------QSSLFTQVFKVLMQRSPVCREM---------------------------FQKM-------SIVGGFSSNSVCDLNSHTKLLCELLDSLMTDL----------------------HQPAKIVLAKCQDVGAAHVNMNEKCCG---V-------------VFDQLGEAFTELITKVEC-------------------------------------VRSKREAVKSWMCVISYMADSIKSGYMEEWAKKR**

**---------|--||--|-----------------------|--||--||---|--------------------------------|--|--------------------|--||--||--|--||-----------------------------------|--|---||-----------|--------------|--|--|||-||---------------------------------------------------||--||--|---|------------**

**CBP05578/CBG23115 gi|39591955|CAE75175 206aa (33-189)**

**HLSPHQVQLLTSTWPRIKT-----------------QSHLFTQVFKVLMQRSPVCREM---------------------------FQKM-------SIVGGFSSNSVCDLNSHAKLLSELLDSLMTDL----------------------QQPAKIVLAKCQDVGAAHVNMNEKCCG---V-------------VFDQLGEAFTDQKVECVR---------------------------------------SKREAVKSWMCVISYMADSIKSGYMEEWAKKR**

**---------|--||--|-----------------------|--||--||---|--------------------------------|--|--------------------|--||--||--|--||-----------------------------------|--|---||-----------|--------------|--|--|||-||---------------------------------------------------||--||--|---|------------**

**(12)**

**CE05316/C26C6.7 370aa (194-353)**

**PLTCAQIHLVRALWRQVYT--------------TKGPTVIGASIYHRLCFKN-VMVKEQ--------------------------MKQV--------ELPPKFQNRDNFIKAHCKAVAELIDQVVENL-----------------------DHLDNVTGELMRIGRVHAKVLRGE-----L----------TGKLWNTVAETIIDCTLEWGD------------------------------------RRCRSETVRKAWALIVAFVIEKIKAGHHEQRKLML**

**---------|--||--|-----------------------|--||--||---|--------------------------------|--|--------------------|--||--||--|--||-----------------------------------|--|---||-----------|--------------|--|--|||-||---------------------------------------------------||--||--|---|------------**

**CBP02907/CBG11881 gi|39582475|CAE66566 377aa (206-361)**

**PLTCAQIHLVRALWRQVYT--------------TKGPTVIGASIYHRLCFKN-LMVKEQ--------------------------MKQV--------ELPPKFQNRDNFIKAHCKAVAELIDQVVENL-----------------------DHLDNVTGELMRIGRVHAKVLRGE-----L----------TGKLWNTVAETIIDCTLEWGD----------------------------------------RRTVRKAWALIVAFVIEKIKAGHHEQRKLMG**

**---------|--||--|-----------------------|--||--||---|--------------------------------|--|--------------------|--||--||--|--||-----------------------------------|--|---||-----------|--------------|--|--|||-||---------------------------------------------------||--||--|---|------------**

**(13)**

**CE05911/F52A8.4 gi|3877400|CAA95823 238aa (69-232)**

**EPNVYEKELLRRTWSDEFD----------------NLYELGSAIYCYIFDHNPNCKQL---------------------------FPFI-------SKYQGDEWKESKEFRSQALKFVQTLAQVVKNI-----------------------YHMERTESFLYMVGQKHVKFADRG-----F----------KHEYWDIFQDAMEFALEHRLS-------------------------------IMTDLDDNQKRDAVTVWRTLALYTTVHMRNGFIDGGLKGVN**

**---------|--||--|-----------------------|--||--||---|--------------------------------|--|--------------------|--||--||--|--||-----------------------------------|--|---||-----------|--------------|--|--|||-||---------------------------------------------------||--||--|---|------------**

**CBP02911/CBG11915 gi|39586180|CAE6659 237aa (68-231)**

**EPNVYEKELLRRTWSDEFD----------------NLYELGSAIYCYIFDHNPNCKQL---------------------------FPFI-------SKYQGDEWKESKEFRSQALKFVQTLAQVVKNI-----------------------YHMERTESFLYTVGQKHVKFADRG-----F----------KHEYWDIFQDAMEFALEHRLS-------------------------------IMTDLDDNQKKDAVTVWRTLALYTTVHMRNGFIDGGLKGVN**

**---------|--||--|-----------------------|--||--||---|--------------------------------|--|--------------------|--||--||--|--||-----------------------------------|--|---||-----------|--------------|--|--|||-||---------------------------------------------------||--||--|---|-------------**

**(14)**

**CE06490/T22C1.2 gi|3880027|CAA99921 183aa (26-172)**

**ILNSYQKSIVRNAWRHMSQ---------------KGPSNCGSTITRRMMARKSTIGD----------------------------IL------------------DRSTLDYHNLQIVEFLQKVMQSL-----------------------DEPDKISKLCQEIGQKHAKYRRSKGMK--I------------DYWDKLGEAITETIREYQG---------------------------------WKIHRESLRAATVLVSYVVDQLRFGYSRGLHVQGSRDT**

**---------|--||--|-----------------------|--||--||---|--------------------------------|--|--------------------|--||--||--|--||-----------------------------------|--|---||-----------|--------------|--|--|||-||---------------------------------------------------||--||--|---|------------**

**CBP02022/CBG08252 gi|39582084|CAE63727 181aa (24-170)**

**ILNSYQKSILRNAWRHMSQ---------------KGPSNCGSTITRRMMARKSTIGD----------------------------VL------------------DRSTLDYHNIQIVEFLQKVMQSL-----------------------DEPDKISKMCQEIGQLHAKYRRSKGMK--I------------DYWDKLGEAITETIREYQG---------------------------------WKIHRESLRAATVLVSYVVDQLRFGYSRGLHVQGSRDT**

**---------|--||--|-----------------------|--||--||---|--------------------------------|--|--------------------|--||--||--|--||-----------------------------------|--|---||-----------|--------------|--|--|||-||---------------------------------------------------||--||--|---|------------**

**(15)**

***CE36271/C18C4.1 258aa (74-234)**

**RIVDDDFELARTHWIQLQK--------------SNKQGLAIRGCFLTMLEKYPQVRPIWG-------------------------FGK---RIEGRGDETWKPEIVEDFYFRHHCASLQAALNMIIQN----------------------KDDKSGMRRMLNEMGAHHFFYDACEPH---F------------EVFQDSLLESMKLVLNGGD--------------------------------------SLDDDIEQSWICTSLRIPPGSFEYDNSK**

**---------|--||--|-----------------------|--||--||---|--------------------------------|--|--------------------|--||--||--|--||-----------------------------------|--|---||-----------|--------------|--|--|||-||---------------------------------------------------||--||--|---|-------**

**CBP22220/CBG09371 gi|39593145|CAE64614 364aa (88-248)**

**RIVDDDFELARAHWIQLQK--------------SNKQGLAIRGCFLTMLEKYPQVRPIWG-------------------------FGK---RIEGRIDETWKPELVEDFYFRHHCASLQAALNMIIQN----------------------KDDRNGMRRMLNEMGAHHFFYDACEPH---F------------EVFQDCLLESMRLVLNGGD--------------------------------------ALDDEIEHSWICLLQTIRLHMGEGIEIQRANYL**

**---------|--||--|-----------------------|--||--||---|--------------------------------|--|--------------------|--||--||--|--||-----------------------------------|--|---||-----------|--------------|--|--|||-||---------------------------------------------------||--||--|---|------------**

**(16)**

**CE12774/R11H6.3 gi|3879147|CAB07647 387aa (133-305)**

**NLKHEHIRALKTTWARLCE-------------PPRANCKGIVSLVERVWEKL-DTKDKDVRNI----------------------FYNAAFVDSMHERCERRRSGSIATLRDHTHFFVSLVSQVVSSL----------------------EKEPAKILEHLDHIGQSHAYLKRYG-----F----------KSSHWEKVGEYFVDHVVIQDC-------------------------------------VRGFPEACRAWTVLVSSIVDRLRAAPRRGSFLNS**

**---------|--||--|-----------------------|--||--||---|--------------------------------|--|--------------------|--||--||--|--||-----------------------------------|--|---||-----------|--------------|--|--|||-||---------------------------------------------------||--||--|---|------------**

**CBP21478/CBG04577 gi|39589868|CAE6086 330aa (73-245)**

**WNLKHEHIRALKTTWARLC------------EPPRANCKGIVSLVERVWEKL-DTKDKDVRNI----------------------FYNAAFVDSMHERCERRRSGSIATLRDHTHFFVSLVSQVVSSL----------------------EQEPAKILDHLDHIGQSHAYLKRYG-----F----------KSSHWEKVGEYFVDHVVIQDC-------------------------------------VRGFPDACRAWTVLVSSIVDRLRAAPRRGSFLNS**

**---------|--||--|-----------------------|--||--||---|--------------------------------|--|--------------------|--||--||--|--||-----------------------------------|--|---||-----------|--------------|--|--|||-||---------------------------------------------------||--||--|---|------------**

**(17)**

**CE17437/C23H5.2 gi|32453028|AAP82661 278aa (6-168)**

**RFSQEEKDILRRSWKVLDK----------------NLNHTAYNIFEMIFNQSPDTRQL---------------------------FPFM----------KFNTGGRSKEIEFHALRFMQVLESVVKTL-----------------------DNPETLNPLCDNLGRVHGRLSESRG----F----------RTHHWGVFIECTLFHFRKVLG------------------------------QDTYFHRMDALDKVIINWRIIIRLLIKQMKRGFNTDIKNRQ**

**---------|--||--|-----------------------|--||--||---|--------------------------------|--|--------------------|--||--||--|--||-----------------------------------|--|---||-----------|--------------|--|--|||-||---------------------------------------------------||--||--|---|------------**

**CBP02580/CBG10551 gi|39580931|CAE65561 278aa (6-168)**

**RFSPEEKDIIRRSWKVLDK----------------NLNNTAYNIFEMIFNQSPDTKQL---------------------------FPFM----------KFQQSGKSKEIEFHGLRFMQVLESVVKTL-----------------------DNPESLNPLCDNLGRVHGRLSESRG----F----------RTHHWGVFIECTLFHFRKVLS------------------------------QDSYFHRMETLDKVIINWRTILRLLIKQMKRGFNTDIKNRQ**

**---------|--||--|-----------------------|--||--||---|--------------------------------|--|--------------------|--||--||--|--||-----------------------------------|--|---||-----------|--------------|--|--|||-||---------------------------------------------------||--||--|---|------------**

**(18)**

***CE18207/T06A1.3 136aa**

**--------------------------------------MTSEKDNHIYLNDFPQNKDL---------------------------YLKL----KNVNAQTVDMNCSDPGFEAIAAQYLKVFDDVITAV-------------------EEKPGDVQTACDRLQAVGKMHRQK---------V-------SGMDGTMFQNMEEPFIQMVSHILQ-------------------------------------DRFNEKAEMLYRKFFQFCLKYLLEGFNG------**

**---------|--||--|-----------------------|--||--||---|--------------------------------|--|--------------------|--||--||--|--||-----------------------------------|--|---||-----------|--------------|--|--|||-||---------------------------------------------------||--||--|---|------------**

**CBP13608/CBG24799 gi|39579284|CAE56950 147aa**

**-----------------------------------ISKSQNFRISELYLNDFPENKDL---------------------------YPKL----KNVKAATVDMNCSDPGFEAVAAQYLKVFDDVITAV-------------------EEKPGDVQTACDRLVAVGKMHRQK---------V-------SGMNVSMFQNMEEPFILMVKHVLQ-------------------------------------DRFNEKAEMLYRKFFQFCLKYLLEGFNG------**

**---------|--||--|-----------------------|--||--||---|--------------------------------|--|--------------------|--||--||--|--||-----------------------------------|--|---||-----------|--------------|--|--|||-||---------------------------------------------------||--||--|---|------------**

**(19)**

**CE19039/Y17G7B.6 gi|3947607|CAA19459 216aa (37-201)**

**NLSVKQKKLLRQSFNAMNS--------------GGTFLKLMEKIFRRLETKCPDMRSI---------------------------FLT--TAFVNSLSRERQTPPLVKTEYDHCKCMVGIFERLIENL-----------------------ENINEQLTMIRHYGEKHAQMAESG-----F----------TGAMIEQFGEISVFVIGSQDV-------------------------------------VKFNHETVKAWRLLLACVTDEMKVGFDRMSRING**

**---------|--||--|-----------------------|--||--||---|--------------------------------|--|--------------------|--||--||--|--||-----------------------------------|--|---||-----------|--------------|--|--|||-||---------------------------------------------------||--||--|---|------------**

**CBP04921/CBG21021 gi|39591498|CAE73552 216aa (37-201)**

**NLTAKQRKLLRQSFNAMNS--------------GGTFLKLMEKIFRRLENKCPDMRSI---------------------------FLT--TAFVNSLSRERQTPPLVKTEHDHCKCMVGIFERLIDNL-----------------------DNINEQLNMIRHYGEKHAQMAESG-----F----------TGAMIEQFGEISVFIIGSQDV-------------------------------------VKFNHETVKAWRLLLACVTDEMKVGYDRMTRING**

**---------|--||--|-----------------------|--||--||---|--------------------------------|--|--------------------|--||--||--|--||-----------------------------------|--|---||-----------|--------------|--|--|||-||---------------------------------------------------||--||--|---|------------**

**(20)**

**CE19638/Y57G7A.9 gi|17537679|NP_493944 169aa**

**RRPKLDIDRVRSVWMDHIN----------------GNDQYFQEVIHRICKRN-EGIRCA--------------------------MLAPNAQHAESVAEEDFVLSNIADRISQFFHQLVEDDVLMDTV---------------------------ELKKACYDLGRQHSSYSKQQ-----F----------KMSYWEEFTLTMMGVLEQNYP--------------------------------------ETTKEEQKAWLHFLRFVNENMLDGYLDAISRSN**

**---------|--||--|-----------------------|--||--||---|--------------------------------|--|--------------------|--||--||--|--||-----------------------------------|--|---||-----------|--------------|--|--|||-||---------------------------------------------------||--||--|---|------------**

**CBP01805/CBG07112 gi|39586983|CAE62918 169aa**

**RRPKLDVERIRTVWMEHIN----------------GNDDYFQEVIRRICKRN-EGIRCA--------------------------MLTQNAQHAESVAEEDFVLSNIADRISQFFHQLIEDDVLMNTV---------------------------ELKKSCYDLGRQHAAYSKKQ-----F----------KISFWEEFTLTMMDVLEQNYP--------------------------------------QTTKEEQKAWLHFQRFINENMLDGYLDALSYNN**

**---------|--||--|-----------------------|--||--||---|--------------------------------|--|--------------------|--||--||--|--||-----------------------------------|--|---||-----------|--------------|--|--|||-||---------------------------------------------------||--||--|---|------------**

**(21)**

**CE21369/Y15E3A.2 gi|6425266|CAB60330 217aa (46-199)**

**TISPEHQKLIKRSWNRIPK---------------AQFGRASLEAFITAAQV-THAI-----------------------------FVD------------------KETENRHVKYFVDLVQSCVDNL----------------------ENLETGVKPWLDLIGRGHANFK--------I----------TGKHWEKFGESLLTTATEWNG------------------------------------PGRRHKETVKAWMVMSSFLADRLAHASRLAHHSPM**

**---------|--||--|-----------------------|--||--||---|--------------------------------|--|--------------------|--||--||--|--||-----------------------------------|--|---||-----------|--------------|--|--|||-||---------------------------------------------------||--||--|---|------------**

**CBP07717/CBG07681 gi|39596678|CAE63297 210aa (48-201)**

**NLVPEQQRLIRQSWNRIPK---------------WQFAKSVLTAFFRACHA-PQLI-----------------------------FPN------------------QETEQRHIKYIVELVQSCVGKL----------------------DNLEEGLKPLVELLGRGHSNFR--------I----------TGPHWEKFAEALMSVASEYNG------------------------------------PGRRHRDIGRAWMLLSSFLADRLAHASRTSHQSPM**

**---------|--||--|-----------------------|--||--||---|--------------------------------|--|--------------------|--||--||--|--||-----------------------------------|--|---||-----------|--------------|--|--|||-||---------------------------------------------------||--||--|---|------------**

**(22)**

**CE36044/Y58A7A.6 gi|17566038|NP_504363 230aa (91-230)**

**GLSRDDKRIIETCWFKCSQ---------------KQLRKCSCDMFWDILHTD-EDILRL--------------------------FR-L-------DHVAPNRLKDNDYFKSHASNLALVLNLVVTNL----------------------QDNFDQAQDALQALGYQHLHL---------I----------DRTHFQTMYWDIFTDCFE-------------------------------------------RNPPPSFKKGAEREVALKFHRF---------**

**---------|--||--|-----------------------|--||--||---|--------------------------------|--|--------------------|--||--||--|--||-----------------------------------|--|---||-----------|--------------|--|--|||-||---------------------------------------------------||--||--|---|------------**

**CBP02078/CBG08670 gi|39583978|emb|CAE64068.1 246aa (114-246)**

**gltrddkrvietcwfkcsq---------------kqlrkcscdmfwdilhtd-eeilrl--------------------------fr-l-------dhvapnrlkendyfkshaanlalvlnlvvtnl----------------------qdnfeqaqdalqalgyqhlhl---------i----------drthfqsmywdiftdcfe-------------------------------------------rnpppsfkkgaerev----------------**

**---------|--||--|-----------------------|--||--||---|--------------------------------|--|--------------------|--||--||--|--||-----------------------------------|--|---||-----------|--------------|--|--|||-||---------------------------------------------------||--||--|---|------------**

**(23)**

**CE06847/C18C4.9 gi|17558104|NP_504469 389aa (197-354)**

**HLTQPQILFVRKTWNHARN---------------QGALEPAISIFRNSFFKNPEIRQMIM-------------------------FGT--------------KNEGHERLKKHAQLFTVLMDDLIANL-----------------------DSPSATVAGLREAGEKHVWPTRNQ-----Y------GCPFHAHLLDQFATAMIERTLEWGE------------------------------------KKDRTETTQRGWTKIVLFVTEQLKEGFQDEQKRAR**

**---------|--||--|-----------------------|--||--||---|--------------------------------|--|--------------------|--||--||--|--||-----------------------------------|--|---||-----------|--------------|--|--|||-||---------------------------------------------------||--||--|---|------------**

**CBP02293/CBG09371 gi|39593143|CAE64612 390aa (197-354)**

**HLTQPQILFVRKTWNHARN---------------QGALEPAISIFRNSFFKNPEIRQMIM-------------------------FGT-------------KNEGHERLKAEHAQLFTVLMDDLITNL-----------------------DSPSATVAGLREAGEKHVWHVKNQ-----Y------GCPFHAHLMDQFATAMIERTLEWGE------------------------------------KKDRTETTQRGWTKIVLFVTEQLKEGFQDEQKRAR**

**---------|--||--|-----------------------|--||--||---|--------------------------------|--|--------------------|--||--||--|--||-----------------------------------|--|---||-----------|--------------|--|--|||-||---------------------------------------------------||--||--|---|------------**

**(24)**

**CE30683/R90.5 gi|20803774|CAD31696 322aa (142-303)**

**GLSPYQQKLLVQCWPNIYT--------------TGASGPFANSLYSTLSSRNAKAKELLAKADGVAV------------------FSK----------------SDFDCSVMHCRVTVEILDTVIKNL----------------------DNDHARITQYLTEIGKQHRHLKAEG-----L----------SSAVWDDLGDTIMDCARRRCE------------------------------------AVRKHKELRRAWLAIIAYIMDNLKQGQSMTRSSST**

**---------|--||--|-----------------------|--||--||---|--------------------------------|--|--------------------|--||--||--|--||-----------------------------------|--|---||-----------|--------------|--|--|||-||---------------------------------------------------||--||--|---|------------**

**CBP02390/CBG09511 gi|39593254|CAE64724 322aa (142-303)**

**GLTAYQQKLLTQSWPNIFT--------------TGASGPFANSLFATLSARNAKAKELLTKANGVAV------------------FAK----------------SDMDCSMMHCRVTVEVLDTVIKNL----------------------DADHSRVTQYLTEVGKQHRHLKAEG-----L----------SSAVWDDLGDTIMDCARRRCE------------------------------------AVRKHKELRRAWLAIIAYIMDNLKQGQSVTRASSS**

**---------|--||--|-----------------------|--||--||---|--------------------------------|--|--------------------|--||--||--|--||-----------------------------------|--|---||-----------|--------------|--|--|||-||---------------------------------------------------||--||--|---|------------**

**(25)**

**CE30888/C36E8.2 gi|25146396|NP_497802 254aa (42-188)**

**ILSVNQRQIIKGCMDNSKD-------------------DLGERIFRRALERR-DDFKQ---------------------------FI-------------------DNLSKGQRYENSQYLKQFLLGI-------------------VENIMDIDEINRISEEFGCNHVQFRANG-----F---------KPDFFACTADAVTTECTFLDQA-------------------------------------AHPTSETAAAWSLLTSHVFSAVRDGYYAELRRQR**

**---------|--||--|-----------------------|--||--||---|--------------------------------|--|--------------------|--||--||--|--||-----------------------------------|--|---||-----------|--------------|--|--|||-||---------------------------------------------------||--||--|---|------------**

**CBP14781/CBG03635 gi|39583310|CAE60102 235aa (45-191)**

**LLSVNQRQIIKGCMDNSKD-------------------DLGERIFRRALERR-DDFKQ---------------------------FI-------------------DKLTKKQRYENSSYLKQFLLGV-------------------VDNLTDIDEINRLAEDFGCNHVQFRANG-----F---------KPDFFACTADAVTTECTFLDQA-------------------------------------AHPTSETAAAWSLLTSHVFSAVRDGYYAELRRQR**

**---------|--||--|-----------------------|--||--||---|--------------------------------|--|--------------------|--||--||--|--||-----------------------------------|--|---||-----------|--------------|--|--|||-||---------------------------------------------------||--||--|---|------------**

**(26)**

**CE31132/Y75B7AL.1 gi|25148647|NP_503405 542aa (373-539)**

**QLLGDRLSILKSSWEKANE---------------MTNGEIGVRVAWNMVRKHPNLCKNDEPEK----------------------VSLL--------NGSCKRSIDHAKFQEIGGRITSFISELLELM--------------------QNNQPESYIVMRIRRVGAVHYDKG--------I--------VFTSSVWKEFKHTIQTIISEVQF-----------------------------------SSPQEREAALDAWNIFISFIIREMKMGIWAIGDTIG**

**---------|--||--|-----------------------|--||--||---|--------------------------------|--|--------------------|--||--||--|--||-----------------------------------|--|---||-----------|--------------|--|--|||-||---------------------------------------------------||--||--|---|------------**

**CBP15597/CBG06424 gi|39592734|CAE62348 542aa (371-539)**

**QLLGDRLSILISSWEKANE---------------MTNGEIGVRVAWNMVRKHPTMCKKTLPEPEK--------------------VSLL--------NGSCKRSIDHAKFQEIGGRISGFIAELLELM--------------------RTNQAESYIIMRIRRVGAVHYDKG--------I--------VFSSSVWKEFKYTIQSIISEVQF-----------------------------------SSPQEREAALDAWNIFISFIIREMKMGIWAIGDTIV**

**---------|--||--|-----------------------|--||--||---|--------------------------------|--|--------------------|--||--||--|--||-----------------------------------|--|---||-----------|--------------|--|--|||-||---------------------------------------------------||--||--|---|------------**

**(27)**

**CE31930/F35B12.8 gi|25809206|CAD57695 234aa (35-191)**

**ICEQEEVNKMIESYQKIDD-----------------KYALFEQMFLTIFLEQ-EVEMAYS-------------------------FG-L-------ENLNEQQLKVEQKFRTHVGKFQRFITGIIDML-------------------SKGVESSDQIVEILRIVGRQHGNVRTMS-----F----------TAEKWLIFKNVLLDLLCKDA-----------------------------------------NEKVGATWNKLISFMISEVKDSYLEHVRHAR**

**---------|--||--|-----------------------|--||--||---|--------------------------------|--|--------------------|--||--||--|--||-----------------------------------|--|---||-----------|--------------|--|--|||-||---------------------------------------------------||--||--|---|------------**

**CBP12391/CBG23302 (CBP19339/CBG18593) 234aa (35-191)**

**ICEQEEVNKMIESYQKIDD-----------------KYALFEQMFLTIFLEQ-EVEMAFS-------------------------FG-L-------ENLNEQQLKVEQKFRTHVGKFQRFITGIIDML-------------------SKGVESSDQIVEILRIVGRQHGNVRTMS-----F----------TAEKWLIFKNVLLDLLCKDA-----------------------------------------NEKVCGTWNKLISFMISEIKDSYLEHVRHAR**

**---------|--||--|-----------------------|--||--||---|--------------------------------|--|--------------------|--||--||--|--||-----------------------------------|--|---||-----------|--------------|--|--|||-||---------------------------------------------------||--||--|---|------------**

**(28)**

***CE34541/F21A3.6 gi|32697997|CAB04152 236aa, (84-236)**

**RLSDRQRDVLQKTFAPILQ----------------DCVRNGLKIFVRLFSEYPRYKLIWPQ------------------------F----------RAIPDSSLMNAVELRRHASVYLNGLGKIIDSM-----------------------RDEEALGKSMSRIAVAHIKWN--------V----------QRNHVIHMIEPVLEVVKECNG-------------------------------------YQLDDETRQAWTVLYQVIADLIEVFRCRALND--**

**---------|--||--|-----------------------|--||--||---|--------------------------------|--|--------------------|--||--||--|--||-----------------------------------|--|---||-----------|--------------|--|--|||-||---------------------------------------------------||--||--|---|------------**

**CBP19339/CBG18593 gi|39582852|CAE71628 227aa (75-227)**

RLSDRQREILQKTFTSIEQ----------------DCVRNGLKIFVRLFAEYPRYKLIWPQ------------------------F----------RAIPDSSLMNAVELRRHASVYLKGLGKIIESM-----------------------RDEEELRKSMSRIALAHIKWN--------V----------QRNHVIHMIEPVLEVVKECNG-------------------------------------YQLDDETRQAWTVLYQVIANLIEVFRCRALND--

**---------|--||--|-----------------------|--||--||---|--------------------------------|--|--------------------|--||--||--|--||-----------------------------------|--|---||-----------|--------------|--|--|||-||---------------------------------------------------||--||--|---|------------**

**(29)**

**CE34658/C06E4.7 gi|33285222|AAA82476 230aa (27-209)**

**NLSVAERQCICASWEKAST-----------------QSDIGCELVARLLNDN-RTRFRALLECKSGS------------------FLG-------SGNYTTEDVNGMKRARSVADGVNCFFNKVISKL--------------------MDANYVEEIQDLSLQLGAMHFRMK--------V--------WFQAENWLCVKNCLLDSVVSALL---------------------KDTKGTYVICGGLKKVQTVEKHTTHAWFKFVQFIIQNMKKGFLAEALNSD**

**---------|--||--|-----------------------|--||--||---|--------------------------------|--|--------------------|--||--||--|--||-----------------------------------|--|---||-----------|--------------|--|--|||-||---------------------------------------------------||--||--|---|------------**

**CBP07299/CBG05824 gi|39593557|CAE61849 220aa (26-208)**

**NLSIAERQVICASWEKAST-----------------QSDIGCELVARLLNDN-RTRFRALLECKSGS------------------FLG-------SGNYTTEDVNGMSRARSVADGVNCFFNKVISKL--------------------MDHNYIEEIQDLSLQLGAMHFRMK--------V--------WFQAENWLCVKNCLLDSVVSALL---------------------KDTKGTYLICGGIKKVQTVEKHITHAWFKFVQFIIQNMKKGFLSEALNSD**

**---------|--||--|-----------------------|--||--||---|--------------------------------|--|--------------------|--||--||--|--||-----------------------------------|--|---||-----------|--------------|--|--|||-||---------------------------------------------------||--||--|---|------------**

**(30)**

**CE34888/F56C4.3 gi|33300043|CAE17840 214aa (10-172)**

**SFTQEEKNDLEHSWNLVEG----------------KKNHIACDIYEMIFNQCPEARRL---------------------------FPKL-------KFVGSKPDRKNNEFAFQAMRFMQVIEGAVKAL-----------------------DHLTSLDVILDNLGRRHGKLEVNGK----F----------RSYYWSVFLECSIYCLRHAFS---------------------------------KRMNDKEVDHVIILWRYLLRDVMKKIKAGTTADIAHRL**

**---------|--||--|-----------------------|--||--||---|--------------------------------|--|--------------------|--||--||--|--||-----------------------------------|--|---||-----------|--------------|--|--|||-||---------------------------------------------------||--||--|---|------------**

**CBP06748/CBG04428 gi|39581850|CAE60743 220aa (24-186)**

**CFTQEEKNDLEHSWNLVEG----------------KKNHIACDIYEMIFNQCPEARRL---------------------------FPKL-------KFVGSKPDRKNNEFAFQAMRFMQVIEGAVKAL-----------------------DHLTSLDVILDNLGRRHGKLEVNGK----F----------RSYYWSVFLECSIYCLRHAFS---------------------------------KRMNDKEVDHVIILWRYLLRDVMKKIKAGTTADIAHRM**

**---------|--||--|-----------------------|--||--||---|--------------------------------|--|--------------------|--||--||--|--||-----------------------------------|--|---||-----------|--------------|--|--|||-||---------------------------------------------------||--||--|---|------------**

**(31)**

**CE34964/R102.9 gi|33300310|CAE17922 196aa (16-189)**

**ELTTDEMQAVRDAWKRAKE------------------REIGKHILRALIERKPQ-------------------------------FKDY---FGIHVDEKNHDVYSCREFQLQAHRIQNFLDTAVSSL-------------------------------GFCPIGNIHQMAYRIGQ----I------------HFYRGVNFGADNWLTFKKV----------TVEIVTKDCGNSESSSMDLKSVPSLFPSSSSTVIIIGWEKFMSSVIREMKKGFLDEARRNC**

**---------|--||--|-----------------------|--||--||---|--------------------------------|--|--------------------|--||--||--|--||-----------------------------------|--|---||-----------|--------------|--|--|||-||---------------------------------------------------||--||--|---|------------**

**CBP23619/CBG17640 gi|39594987|CAE70855 112aa**

**ELSSDEMQAVRDSWKRAKE------------------REIGKHILQALIERKPQ-------------------------------FKDY---FGIHVDEKKDDVFSCREFQLQSHRIQNFLDTAVSSL-------------------------------GFCPIGNIHQMAYRIGQ----I-------------HFYR-------------------------------------------------------------------------------------**

**---------|--||--|-----------------------|--||--||---|--------------------------------|--|--------------------|--||--||--|--||-----------------------------------|--|---||-----------|--------------|--|--|||-||---------------------------------------------------||--||--|---|------------**

**(32)**

**CE35828/C09H10.8 344aa (26-190)**

**HDPQLLSDAISEAWLKSAE--------------------LTPTWVWALVDL-PETMHATYKENAQ--------------------FLNI--------------INQVRDFLNILIKVHKCDPEMIKTL------------------------------SFRLGARHRHYMNEGNDNC---F----------WAPFAQQLPIAMSKMYMRVVT--------------------EDSKIRRILrirsraaekseeeevceswrqfscmliesmkrgyegcASEKT**

**---------|--||--|-----------------------|--||--||---|--------------------------------|--|--------------------|--||--||--|--||-----------------------------------|--|---||-----------|--------------|--|--|||-||---------------------------------------------------||--||--|---|------------**

**CBP14482/CBG02965 gi|39597340|CAE59568 325aa (9-173)**

**LSTHRKHVVHTEAWLKSAE--------------------LTPTWVWALVDL-PETMHSRYKENAQ--------------------FLNI--------------INQVRDFLNILIKVHKCDPEMIKTL------------------------------SFRLGARHRHYMNEGNDNC---Y----------WAPFAQQLPIAMSKMYMRVVN--------------------EDSKIRRILRIRSRAAEKSEEEEVCESWRQFSCMLIESMKRGYEGCASEKT**

**---------|--||--|-----------------------|--||--||---|--------------------------------|--|--------------------|--||--||--|--||-----------------------------------|--|---||-----------|--------------|--|--|||-||---------------------------------------------------||--||--|---|------------**

**(33)**

**CE23430/Y22D7AR.5 272aa (6-175)**

**HLTPIDREILNKSWGIVSK----------------DMQQVAVNIFQMIFEQAPDAKLM---------------------------FSFM--------MKDYKEDKKSNEFIFHAVRFLQVIESTMTHL-----------------------EDPAQLDAVFLNLGKIHAKHEEQ------L--------GFSAHYWSVFKECVLFHFRKAMK-------------------------SHNKFHKRNEMSFAEIDSAIILWREVLRFIIDRMKVGYSESGAIRK**

**---------|--||--|-----------------------|--||--||---|--------------------------------|--|--------------------|--||--||--|--||-----------------------------------|--|---||-----------|--------------|--|--|||-||---------------------------------------------------||--||--|---|------------**

**cb25.fpc2587.0.059.0 (corrected CBP02869/CBG11737 298aa (12-181)**

**HLTPIDREILNKSWGIVSK----------------DMQQVAVNIFQMIFEQAPDAKLM---------------------------FSFM--------MKDYKEDKKSNEFIFHAVRFLQVIESTMSHL-----------------------DDPSQLDAVFLNLGKIHAKHEEQ------L--------GFSahywsvfkecvlfhfrkamk-------------------------shnkfTkhkemsfaeidsaiilwrevlrfiidrmkvgycesGAIRK**

**Fig. 3**

**Mb fold**

**nn...........................................................Cccc............................................................................F**

# *aaaaaaaaaAaaAAaaAa..........bbbbbBbbBBbbBBbbcCccccc..........Dddd..........dddddddeeeEeeEEeeEEeeEeeEEe...................FffFfffFFf..........G.....ggggGggGggGGGgGGggg..........................................hhhhhhHHhhHHhhHhhhHhhhhhhh*

**VLSEGEWQLVLHVWAKVEA.........DVAGHGQDILIRLFKSHPETLEK..........FDRF......khlkteaeMKASEDLKKHGVTVLTALGAILKKK...........GHHEAELKPLAQSHATKHK.......I.....PIKYLEFISEAIIHVLHSRHP.....................................GDFGADAQGAMNKALELFRKDIAAKYKelgyqg**

**---------|--||--|----------------|--||--||---|---------------|--|--------------------|--||--||--|--||--------------------|--|---||-----------|---------|--|--|||-||---------------------------------------------------||--||--|---|-----------**

**NsHbs**

**>Marchantia gi|15809396|AAK07743**

**----------------------------DSATHSVTFFAKIFEIAPGAKAL----------FSFL--------KDSDIPFDKNPKLKAHALTVFRLTGDSACM-----LGEKGAIDALHPKLKELGKKHVGYG--------V-----IAAHFDVVKAALLSTIESLLPED---------------------------------WPAEKKVATCGAWSQAYDELAGVMINEMQAIRDAE**

**>Euryale Hb1 gi|33590381|AAQ22728**

**-FSEEQEALVVKSWGVMKK---------DAGQLGVKFFAKIFEIAPSAKRM----------FSFL--------RDSDLPLEQNPKLKPHALSVFAMTCESAVQL-----RKAGKVTVRESSLKDLGATHFKYG--------V-----VDEHFDVRFAFSRSVFSSIR--------------------------------------DLFQPEMKIAWGEAYDKLAA-------------**

**>Arabidopsis Hb1 gi|15226675|NP_179204**

**VFTEEQEALVVKSWSVMKK---------NSAELGLKLFIKIFEIAPTTKKM----------FSFL--------RDSPIPAEQNPKLKPHAMSVFVMCCESAVQL-----RKTGKVTVRETTLKRLGASHSKYG--------V-----VDEHFEVAKYALLETIKEAVP-------------------------------------EMWSPEMKVAWGQAYDHLVAAIKAEMNLSN---**

**>Trema gi|81428|S00838**

**VFTEEQEALVVKSWAVMKK---------NSAELGLKFFLKIFEIAPSAKNL----------FSYL--------KDSPIPLEQNPKLKPHAMTVFVMTCESAVQL-----RKAGKVTVRESNLKRLGAIHFKMG--------V-----VNEHFEVTRFALLETIKEAVP-------------------------------------EMWSPEMKNAWGEAYDQLVAAIKSEMKPSST--**

**>Oryza Hb1 gi|17366135|O04986|HBL1_ORYSA**

**SFSEEQEALVLKSWAILKK---------DSANIALRFFLKIFEVAPSASQM----------FSFL--------RNSDVPLEKNPKLKTHAMSVFVMTCEAAAQL-----RKAGKVTVRDTTLKRLGATHLKYG--------V-----GDAHFEVVKFALLDTIKEEVP------------------------------------ADMWSPAMKSAWSEAYDHLVAAIDQEMKPAE---**

**>Physcomitrella gi|7658243|AAF66104**

**TYSKENEQLVKQSWEILKK---------DAQRNGINFFRKVFEIAPGAKAM----------YSFL--------RDSTIPFEENPKVKNHARYVFMMTGDAAVQL-----GEKGAYQVLESKLQKLAATHVNAG--------V-----TDDQFEIVKEAILYAIEMGVP-------------------------------------DLWSPELKSAWGDAYDMLAEQVKAEMHAQRSAA**

**---------|--||--|----------------|--||--||---|---------------|--|--------------------|--||--||--|--||--------------------|--|---||-----------|---------|--|--|||-||---------------------------------------------------||--||--|---|-----------**

**SHbs**

**>Canavalia gi|1170183|P42511**

**AFSEKQESLVKSSWEAFKQ---------NVPHHSAVFYTLILEKAPAAQNM----------FSFL-----------SNGVDPNPKLKAHAEKVFKMTVDSAVQL-----RAKGEVV---LADPTLGSVHVQKG--------V-----LDPHFLVVKEALLKTFKEAVG-------------------------------------DKWNDELGNAWEVAYDELAAAIKKAMGSA----**

**>Lupin I gi|126232|P02239**

**VLTDVQVALVKSSFEEFNA---------NIPKNTHRFFTLVLEIAPGAKDL----------FSFL--------KGSSEVPQNNPDLQAHAGKVFKLTYEAAIQL-----QVNGAVA-SDATLKSLGSVHVSKG--------V-----VDAHFPVVKEAILKTIKEVVG-------------------------------------DKWSEELNTAWTIAYDELAIIIKKEMKDAA---**

**>Sesb II gi|225726|prf||1312241A**

**GFTDKQEALVNASYEAFKK---------NLPGHSVLFYSFILEKEPAAKGL----------FSFL--------KDSDGVPQNNPSLQAHAEKVFGLVHDAAAQL-----RATGVVV---LADASLGSVHVQKG--------V-----TDPHFVVVKEALLKTLKEAAG-------------------------------------ATWSDEVSIAWEVAYDGLAAAIKKAMS------**

**>Casuar I gi|454920|CAA54775**

**ALTEKQEALLKQSWEVLKQ---------NIPAHSLRLFALIIEAAPESKYV----------FSFL--------KDSNEIPENNPKLKAHAAVIFKTICESATEL-----RQKGHAVWDNNTLKRLGSIHLKNK--------I-----TDPHFEVMKGALLGTIKEAIK-------------------------------------ENWSDEMGQAWTEAYNQLVATIKAEMKE-----**

**GCSs**

**>AgrtumGCS**

**LGLGHGERQNLSDMKGVITG--------SLDASLDRFYTKVRAV-PETAKF----------FSS-------------EAHIHHAKSMQLKHWSRIASGTFNEDY--------------TNAVTAIGRTHARLGLEPRWY--I-------GGYALMLDGIVKAVIESMWP-------------------------------RGLLAKGGSDRVKDALSATIKAALLDMDYSISVYL---**

**>BacsubGCS**

**VRLGDAELYVLEQLQPLIQE--------NIVNIVDAFYKNLDHESSLMDI-----------IND------------------HSSVDRLKQTLKRHIQEMFAGV---------IDDEFIEKRNRIASIHLRIGL-------L-----PKWYMGAFQELLLSMIDIYEAS---------------------------------------ITNQQELLKAIKATTKILNLEQQLVLE---**

**>Chrvio1GCS**

**FALAPRDEMLLRAAGNLVES--------HLEELVTRFYELQTST-PEIALL----------IGDA------------------DTLQRLRSAQRRYVVDLFSGI---------YDLEYVNIRLRIGLVHKRIGVEPK----L------YLAAVDSLKFLLAEKLTELIPD------------------------------AEVRLHTLQALDKLMMFDVALVFETYIRSLVAEIE---**

**>EsccolGCS**

**GLVEQADPPIRAKAAEIAVA--------HAHYLSIEFYRIVRID-PHA-------------FLS-----------------NEQVERQLKSAMERWIINVLSAQ-------VDDVERLIQIQHTVAEVHARIGIPVE----I-----VEMGFRVLKKILYPVIFHAKC---------------------------------------EKARKLQVYHFSINSIDIAMEVMTRAFT---**

**>GeosulGCS**

**YRFTDEDAELLGSLFPLAET--------NKERLADQFYDYLLGI-PETAE-----------FLK-----------------EDLVLQKLKQTHQDWFVSLFAGS---------YDNRYIHNLQKIGHAHVRVGLNAHY---V------NVAMNVVRQFTLSIIQHLQSPSD---------------------------------RRQRREAVEKILDINLDIMSASYREEEMRKF---**

**>HalmarGCS**

**TGFDGSDVDNLTAMADETNA--------RAEAVVDDFYDHLQSFD-ETVEI----------FGR-----------------STKSVDQLKNTQTQYLRDLVAGT---------YDKQYFENRARIGKIHDMLDLGPKIY--L-------GAYSIYFEHFLRTIVEDLQSG--------------------------------------DAARDEALEEMQSRALSVFKLLNLDQQ---**

**>SinmelGCS**

**AGLDEDACALLRDHRQALSP--------RIELALRALSHRLQAS-PDAARH----------FDS---------------DRQIDRLHDLQSSHWNVLTDARFDG-----LRMGLDPRWQIASHAIVLEHLILGA-------I-------EDAWPKSILSLGKARRRELRDLV------------------------------------AALVRAAFVDTEIAVSLRFNALRQQHQRQ-**

**>VibvulGCS**

**HDLTEADLALIRKFGQIMVP--------KLDEYVKHFYDWLRNT-PEYEQY----------F---------------------GDAQKLQRVQDSQVRYWKTFF-------DARIDSAYLERRDVGEIHARVGLP------L-------PTYFAGMNISMVIFTKRMYDTVH---------------------------------GIAYSSLVTAFTKLLHLDTTIVVDTYSRLI---**

**---------|--||--|----------------|--||--||---|---------------|--|--------------------|--||--||--|--||--------------------|--|---||-----------|---------|--|--|||-||---------------------------------------------------||--||--|---|-----------**

**Pgbs**

**>AerperPgb**

**VMLGEKDVMYLKKACDVLK--------DQVDEILDLWYGWVASNE-HLIYY----------FSNP--------------DTGEPIKEYLERVRARFGAWILDTT------CRDYNREWLDYQYEVGLRHHRSKKGVTDG--V--RTVPHIPLRYLIAFIYPITATIKPFLAK-----------------------------KGGSPEDIEGMYNAWFKSVVLQVAIWSHPYTK-----**

**>MetacePgb**

**VMFTAEDEEYIQKAGEVLE--------DQVEEILDTWYGFVGSH-PHLLYY----------FTSP---------------DGTPNEKYLAAVRKRFSRWILDTC------NRSYDQAWLDYQYEIGLRHHRTKKNQTDN--V--ESVPNIGYRYLVAFIYPITATMKPFLAR-----------------------------KGHTPEEVEKMYQAWFKATTLQVALWSYPYVK-----**

**2/2Hb1s**

**>Halmar**

**-------MASQSIFERIGG-------RDAVDAVVSDFYDRVRDD-PLLEPY----------FEE-------------------TDMDQLRSHQTQFISTVAGGP-------------VDYDGDDMQTAHEGMG--------I-----TEDAFASVATHLEAALRANGVP---------------------------------------DDDVEAILTEVAAMKDDIVEA---------**

**>Mycavi1**

**ILARFRKAEPASIYDRIGG-------HEALEVVVEDFYVRVLADE-QLSGF----------FTG-------------------TNMNRLKGKQVEFFAAALGGP-------------HPYTGAPMKQVHQGRG--------I-----TMHHFGLVAGHLADALTAAGVPS---------------------------------------ETVSEILGAIAPLAPEIATGEA-------**

**>Myxxan1**

**-----------SVYEQLGG-------EPAMAAAVEVFYRKVLADD-HISHF----------FEDV-------------------DMERQAAKQKAFLTMVTGGP-------------VHYSGKDMRAGHAPLVKRG-----L-----NDSHFDAVAGHLKATLEEL-------------------------------------------GVAAPLVARVMTIAESARADV--------**

**>Nospun2**

**----AHNSAGSSLYKRLGG-------YNAIAAVIDDSAKNIFAD-PLIGKY----------FIGL----------------STNSKQRLRELLIAQFCQAAGGP-------------CIYTGRTMKLSHSGIGRG------L-----TNGEFYAFVNDIALALDKNGVKQPE---------------------------------------KNQVLGFANSLRDQIVEKP--------**

**>Syncys**

**---------MSTLYEKLGG-------TTAVDLAVDKFYERVLQDD-RIKHF----------FADV-------------------DMAKQRAHQKAFLTYAFGGT-------------DKYDGRYMREAHKELVENHG----L-----NGEHFDAVAEDLLATLKEMGV-------------------------------------------PEDLIAEVAAVAGAPAHKRDVLNQ---**

**Solibacter usitatus (Acidobacteria) gi|67932871|ZP_00526004133aa**

**>Solusi1**

**......msdekslyarlgg.....ydaisavvdnllprlmsd.sllgr....fw.a.hrgedgilrekqllvdflcqsagg....pvyytgrdmklshkgmq.....i....tesdwqaflghltatldafav....papeketvlafihstkadiidppgskaa**

**.........|..||..|..............|..||..||...|........|..|.......|..||..||..|..||...........|..|...||........|........|..|..|||.||...............||..||..|...|.........**

**>Xanaxo**

**TSACASTQSRQTLYDELGG-------QAGIEALVETMLSRIADD-PRIVDK----------FARV-------------------NIVMLNERLVQKFCHVADGP-------------CPDTAKSMQQAHAHLA--------I-----REGDFNALVEDLNWAMDQRKIP---------------------------------------RRTQNRLLARLAAMHGEIVNH---------**

**.........|..||..|................|..||..||...|...............|..|....................|..||..||..|..||....................|..|...||...........|.........|..|..|||.||...................................................||..||..|...|...........**

**2/2Hb2s**

**>Agrtum1**

**SKEATMSSETVTLYEAIGG-------DATVRALTRRFYELMDTL-PEAARCRA--------IHPA-------------------DLSGSEAKFYDYLTGYLGGP---------PVYVEKHGHPMLRRRHFVAP--------I-----GPAERDEWLLCFRRAMDETIE----------------------------------------NAKLREIIWAPVERLAFHMQNQEADNP---**

**>Borper1**

**QPISEPLDTSKTVFDMLGG-------EPGVRELVDRFYDLMDME-SD--------------FKAL-------------RDAHGPSLEQARDKLFWFLCGYFGGP----------HYIERFGHPRLRARHLPFS--------I-----GEIERDQWVACMGRAMQDQQL-------------------------------------------PAALVDRLLQAFYGTADWMRNRAG---**

**>Burcep1**

**AHSLHAAHAPPSPFDRLGG-------AAALARIVDAFYRQMDTR-PDAAG-----------IRAM----------------HGADLGPVKTVLVTYLCEWLGGP---------RHYSAQRGHPRLRMRHRAFA--------I-----GMAERDAWLACMRAALDECGVEP---------------------------------------ALRDELMQALFKLADWLRNTGR-------**

**>Chlaur**

**HYGEVYIMSEPTIYEQIGG-------EATFRRIVDIFYARVEAD-PRLRHL----------FP--------------------ADLEPGKEHQRLFLMQYFGGP----------TYSERRGHPRLRMRHAPFP--------I-----GPRERDAWLEHMLAALNEAGVPE----------------------------------------PARSVMENYFRHAAQAMMNRLGED----**

**>Deirad**

**-MTTPLDLARGSLYDRIGP--------DTLALLVHRFYAHVARN-PDLAPI----------FPA--------------------DLSETARKQLAFLTGFTGGP----------LYHELYGHPRLRARHLPFP--------I-----TPGRARAWLACMNAALRETPGLAEA--------------------------------------DAHELYAALARVAVHMVNTEEAPDSD--**

**>Exiguo**

**-----------MLYEDLGQ-------KQAIEQLVARFYERVYADH-LLRPL----------FP--------------------SDRRQVEQAQTRFLIQLTGGP---------KQYESYDERMNLAMIHRLLP--------I-----TEVHAIRWIELMTMTIEETISDSEA--------------------------------------ATRLIERLRIGALNVLRICDAHQNR---**

**>Kinrad**

**RKPAGPDGGTRSFFDEVGG-------HETFVRLVDAFYEGVAADE-VLRPM----------YP-------------------EADLGPARQRMLMFLEQYWGGP---------ATYSEQRGHPRLRMRHASFK--------V-----NPDARDRWLTHMRAAVVSLGLAP---------------------------------------AQEGVLWDYLDRAAHSMLNTFED------**

**>Lepint**

**GGPPGPIPGLQLVFGSVGE--------NSLRKLVSDFYDQIPSS-SIS-FM----------FP--------------------ENLEDSKIKSADFLIQVTGGP----------LYSQNYGPPKMRARHLPFP--------I-----DEKARRVWLSCYRKVLDDWDAEV---------------------------------------SAKEVLWIFFKDFSTWMVNLESKTEE---**

**>Mycavi2**

**-----------SFYDAVGG-------AETFQAIVSRFYAQVPEDE-ILREL----------YPL-------------------DDLEGAEERLRMFLEQYWGGP----------TYSDRRGHPRLRMRHVPFR--------I-----TPLARDAWLRCMHTAVASIDSKTLD------------------------------------DEHRRELLDYLEMAAHSLVNSP--------**

**>Staaur**

**---------MTTTPYDIIG-------KEALYDMIDYFYTLVEKDE-RLNHL----------FP--------------------GDFAETSRKQKQFLTQFLGGP-----------YTEEHGHPMLRKRHMDFT--------I-----TEFERDAWLENMQTAINRAAFPQG---------------------------------------VGDYLFERLRLTANHMVNS---------**

**---------|--||--|----------------|--||--||---|---------------|--|--------------------|--||--||--|--||--------------------|--|---||-----------|---------|--|--|||-||---------------------------------------------------||--||--|---|-----------**

**2/2Hb3s**

**>Acifer3**

**---MEQVSPPKRRLEPLCE----KITLPVIRAVVDDFYNRIQHH-PTLAEP----------FSIV------------------QDWELHKDRLVHYWWTVSGGL------------PYKDYRYALGDKHAPLG--------I-----THSLVDDWLALFHETMLDHMDAD-------------------------------------MARRWHGMAAGIGESLRLMFAPR--------**

**>Bdebac2**

**-------MTTPTSRKPLES-------REDVMVLVDSFYAKVRAD-GYIGPI----------FTDV----------------AKVDWEEHLPKLYNFWSDLLLGE--------------DSYRGRPFPPHTKLN--------L-----ERGHFEQWLRLFVETVDEHFVGLKANE-------------------------------------AKERAYRIARNFMINLQLLSLE-----**

**>Borper2**

**----------MGAPDLCTE--------EDITQLVHQFYAEVRRD-AELGPI----------FN-----------------THVDDWDVHLAKLVDFWSSILRGT----------RGTPMPRHVALPGLHAG----------L---------FERWLRLFRATAAAQPNQAMAEQ-------------------------------------ACLMAGRIAQSLWYGYQLHRSPDQA--**

**>Camjej**

**-----------MKFETINQ--------ESIAKLMEIFYEKVRKDK-DLGPI----------FNNA-------------IGTSDEEWKEHKAKIGNFWAGMLLGE--------------GDYNGQPLKKHLD----------L--PPFPQEFFEIWLKLFEESLNIVYNEE-----------------------------------------MKNVILQRAQMIASHFQNMLYKYGGH-**

**>Mycavi3**

**--------------------MPDLRDRADVEALLRRFYGRALDDE-VLAEP----------FARL----------------RATGLDDHVPTMCDFWETVLFRA--------------GRYRGSALQAHRDIHRRAP----L-----SDRHFRRWLTLWHITVDEMYRGPAAD------------------------------------RAKIQAARIAWAMHRRLTGADSPELL---**

**>Novaro**

**-------------------MNDLSITEGDIDRLVPEFYARVRKDE-VLGPI----------FNGA-----------------IEDWPDHLERLKAFWSSIMLTS--------------GRYKGQPLIAHVKHEAH------M-----TPENFARWLELWRQTTGDILGPD-----------------------------------------KAELFRQKAARIAESLTMGVQFYRERQ**

**---------|--||--|----------------|--||--||---|---------------|--|--------------------|--||--||--|--||--------------------|--|---||-----------|---------|--|--|||-||---------------------------------------------------||--||--|---|-----------**

**ALGAL HBS**

**Cyanidioschyzon CMR319C 185aa SDgb**

**>Cyanmer SDgb**

**EPQQESIAIVQSTWSQAVQ.........QRERLGQVFYDRLFALYPELQPM..........F....................RSDPALQRIRLVDMVDAGVKLL........NSRRDLEQALRDLGKRHVK..........Y...GTQEEQYPIVGENLLHALESILG....................................SKHFSEDMRKAWLDVYAYWSSVMLEGAREAQ...**

**.........|..||..|................|..||..||...|...............|..|....................|..||..||..|..||....................|..|...||...........|.........|..|..|||.||...................................................||..||..|...|...........**

**Thalassiosira pseudonana Scaffold_137:50940:51413 158aa SDgb (18-158)**

**>Thapse1 SDgb**

**GLSPEDLSLVQKSWAKVVP.........IASVAADLFYTKLFELDPELRPL..........F....................PSDLADQKKKLMAMISVAVDGL........TDLEALVPAVQDLGRRHAKYYK.......V.....TAPMFDTVGAALLDTLEKGLG.....................................EGWDEEHKEAWTLVYGVLSKTMIDAGEESTAA.**

**Chondrus crispus (Rhodophycota; Rhodophyceae) FHb 284aa (5-159)**

**>Chocri1 FHb**

**EPTPETIAIVKSTVPILRE.........NLTEVITRFYENLFSTYPELKKQ..........FN.M.......DRHRGGTVKEENGIPKQVAALGGAVISYAKNI........DRVEALLPVVERISHKHVSRG........V.....SAAQYDKIGECLLRAMKDILE.....................................EAITEDIHNAWAEAYGFLASVFINMETKLTEEL**

**Chondrus crispus 2/2Hb 169aa (18-154)**

**>Chocri2 2/2Hb**

**PFSAVEDARAAGLYVRIGP........EKLASLSAAFYERVYSDEPWFRAL..........FA...................STSRAAATRNQVEFHAQEFGGP.........RLYEERKGPTMLLGRHGPYA........I.....GKREAERWLALMEAAIDDVAI........................................VGEEAALLRGYFRHMAWYIVYGRELVNP..**

**Thalassiosira pseudonana Scaff_18**

**>Thapse2 2/2Hb**

**QSLLATTSLTPTLLERIGG.......SSGFITLSTLFYNRVFNDKANPW............FLGI...............FATSTKDEAIDNQFRFLVQTFGGE.........ELYRQKGKYTRLVGRHANYQ........I.....QIAAAERWVVHMEGAIDEHEA.....................................LVDDEEARECLKLYFRYTAFYIVVASEFMRDDQ**

**Chlamydomonas reinhardtii 2/2Hb**

**>Chlrei1 2/2Hb**

**--MAADTAPADSLYSRMGG-------EAAVEKAVDVFYERIVAD-PQLAPF----------FANV-------------------DMKKQRRKQVAFMTYVFGGS-------------GAYEGRDLGASHRRL---------IREQGMNHHHFDLVAAHLDSTLQELGV---------------------------------------AQELKAEAMAIVASARPLIFGTGEAGAAN--**

**CILIATE 2/2HBS**

**>Paramecium**

**-----------SLFEQLGG-------QAAVQAVTAQFYANIQADATVATF-----------FNGI-------------------DMPNQTNKTAAFLCAALGGP-------------NAWTGRNLKEVHANMG--------V-----SNAQFTTVIGHLRSALTGAGV-------------------------------------------AAALVEQTVAVAETVRGDVVTV-----**

**PLANT 2/2HBS**

**>Arabidopsis**

**DQAEAFAIDESNLFDKLGL--------QTFINLSTNFYTRVYDDE-EEWFQSI--------FS-------------------NSNKEDAIQNQYEFFVQRMGGP---------PLYSQRKGHPALIGRHRPFP--------V-----THQAAERWLEHMQNALDDSVD----------------------------------------QDSKIKMMKFFRHTAFFLVAGNELKNQN--**

**>Oryza**

**AAGDAFAIDDGNVFEALGG------TTQPFVDLSTNFYTRVYEDE-EEWFRQI--------FA-------------------GSKKEDAIRNQYEFLVQRMGGP---------QLFSQRRGHPALIARHRPFP--------V-----THQAAERWLHHMQQAVDTTDS--------------------------------------IDAATKTKMMYFFRHTAYFLVAGNEMTRQG--**

**---------|--||--|----------------|--||--||---|---------------|--|--------------------|--||--||--|--||--------------------|--|---||-----------|---------|--|--|||-||---------------------------------------------------||--||--|---|-----------**

**BACTERIAL FHbs**

**>BacantF**

**MLSEKTIEIVKSTVPLLQE---------KGVEITTRFYEILFSEHPELLNI----------FNH-----------------TNQKKGRQQQALANAVYAAATYI--------DNLEAIIPVVKQIGHKHRSLG--------I-----KAEHYPIVGTCLLRAIKEVAGA---------------------------------------PDEVLNAWGEAYGVIADAFISIEAEMYEEA**

**>DeiradF**

**MLTPEQKAIVKATVPALEA---------HGETITRTFYASMFAAHPELLNI----------FNPA-----------------NQQTGKQARSLAASVLAYAAHI--------DHPEALGGMVGRIAHKHVSLE--------V-----LPEHYPIVGQYLLGAIAGVLGDAA-------------------------------------KPEILDAWAAAYGELADLMIGIEKGMYDAG**

**>EsccolF**

**MLDAQTIATVKATIPLLVE---------TGPKLTAHFYDRMFTHNPELKEI----------FN-M----------------SNQRNGDQREALFNAIAAYASNI--------ENLPALLPAVEKIAQKHTSFQ--------I-----KPEQYNIVGEHLLATLDEMFSP---------------------------------------GQEVLDAWGKAYGVLANVFINREAEIYNEN**

**>ExiguoF**

**MLTPSAITIIKSTVPVLAE---------HGHSITRVFYQRLFENHPEMKHI----------FNQ-----------------SNQKNDRQSQALATAVYAAAAYI--------DQLETLKPTLLPVLHKHRSLQ--------I-----KPYMYDIVGTELIGAIQDVLKDAA-------------------------------------TPDIIDAWTAGYGEIAKLFISLEAELYQQD**

**>MagmagF**

**PLSARTIAVVKATIPALET---------HGLTITRRMYERLFQNT-DIRDL----------FNQ----------------SHHGETGSQPKALALAVLAYARNI--------DNLGVLTGAVERI-QKHVALN--------I-----LPEHYSFVADALIGAIQDVLGEAA-------------------------------------TPEIVEAWGEAYWFLAELLIGREATIYRDL**

**>MyctubF**

**--MGLEDRDALRVLQNAFKL--------DDPELVRRFYAHWFALDASVRDL----------F--------------------PPDMGAQRAAFGQALHWVYGEL------VAQRAEEPVAFLAQLGRDHRKYG--------V-----LPTQYDTLRRALYTTLRDYLG-----------------------------------------HPSRGAWTDAVDEAAGQSLNLIIGVMSGA**

**>OceiheF**

**LLDKKTTEIIKATVPVLKE---------HGEAITKHFYKILLENNPELKNV----------FNQT-----------------NQRKGAQSKALANTVYAAAANI--------EKLEEILPHVKQIAHKHVSLN--------I-----KPEQYPIVGKYLLIAIKEVLGDAA-------------------------------------TDEIIEAWEKAYFVIADIFISVEKEMYNEK**

**>RhodopirellF**

**MLSEKTIRIVKEITPLVAA---------NAETITRRFYERMFEANPEVKAF----------FNQA-----------------HQHSGGQQKALAGAICAYFTHI--------DNPAVLMPAVELIAQKHVSLG--------I-----KPEHYPIVGSNLLAAIGDVMGDAA-------------------------------------TPEIVEAVSEAYGFLADIFIGREGAIYEEQ**

**>PseaerF**

**MLSNAQRALIKATVPLLET---------GGEALITHFYRTMLGEYPEVRPL----------FNQA-----------------HQASGDQPRALANGVLMYARHI--------DQLQELGPLVAKVVNKHVSLQ--------V-----LPEHYPIVGTCLLRAIREVLGEQIA------------------------------------TDEVLEAWGAAYQQLADLLIEAEESVYAAS**

**>RalmetF**

**MLSAASRPYIDASVPVLRE---------HGLAITTHFYREMFAARPELKRI----------FN-M----------------GNQANGSQQQSLASAVFAYAANI--------DRADVLAPVVERIVHKHVAVG--------L-----TPAHYPIVGKYLLEAIAAVLGDAA-------------------------------------TPPLLAAWDEAYWLLAGELIAAEARLYERH**

**>StaaurF**

**MLTEQEKDIIKQTVPLLKE----------GTEITSIFYPKMFKAHPELLNM----------FNQ-----------------TNQKRGMQSSALAQAVMAAAVNI--------DNLSVIKPVIMPVAYKHCALQ--------V-----YAEHYPIVGENLLKAIQDVTGLEE-------------------------------------HDPVIQAWAKAYGVIADVFIQIEKEIYDQM**

**>Strave1F**

**MLSEQSAATVRATLPAVGA---------AVGEITARFYDRLFAARPELLRDL---------FNR-----------------GNQAAGTQRQALAGSIAAFATYL------VEHPDERPDAMLDRIAHKHASLG--------I-----APGQYAVVHEHLFAAIAEVLGDAV-------------------------------------TPEVAAAWDEVYWLMANALIAIERRLYAQH**

**>VibchoF**

**MLTQEHINIIKSTIPLLES---------AGPALTQHFYQRMFSHNPELKHI----------FN-M----------------THQKTGRQSVALFEAIAAYAKHI--------DNLAALTSAVERIAHKHTSFN--------I-----QPEHYQIVGHHLLETLRELAPDAF-------------------------------------TQPVEEAWTAAYFFLAQVFIDREGALYLER**

**---------|--||--|----------------|--||--||---|---------------|--|--------------------|--||--||--|--||--------------------|--|---||-----------|---------|--|--|||-||---------------------------------------------------||--||--|---|-----------**

**Bacterial SDgbs**

**>Acinet***

**.mtpqqielvkstvpvlre.........hgvtltsyfynrmlnnhpelknv..........fn.l................Dhqstgrqpralaaavlayaehi........eqpeklakaverittkhvsln........i.....qpeqyaivgenllhsisevlnvpf.....................................eselieawkqaylqladilievekekyhQL**

**>Aquaeo***

**MLSEETIRVIKSTVPLLKE---------HGTEITARMYELLFSKYPKTKEL----------F--------------------AGASEEQPKKLANAIIAYATYI-----------DRLDNAISTIARSHVRRN--------V-----KPEHYPLVKECLLQAIEEVLNP---------------------------------------GEEVLKAWEEAYDFLAKTLITLEKKLYSQP**

**>Brajap1***

**-MTPEQITLIQQSFAKVAP---------ISETAAVLFYDRLFEVAPSVRAM----------F--------------------PEDMTEQRKKLMGMLAAVVGGL--------SNLDSILPAASALAKRHVA----------Y---GAKAEHYPVVGATLLWTLEKGLGEA-------------------------------------WTPELATAWTDAYGVLSGYMISEAYGAQAQA**

**>Camjej***

**-MTKEQIQIIKDCVPILQK---------NGEDLTNEFYKIMFNDYPEVKPM----------FN-M----------------EKQISGEQPKALAMAILMAAKNI--------ENLENMRSFVDKVAITHVNLG--------V-----KEEHYPIVGACLLKAIKNLLNPDE---------------------------------------ATLKAWEVAYGKIAKFYIDIEKKLYDK-**

**>Chrvio2***

**PLTQQQIRLVQESFAKVEP---------IADEAAKLFYDKLFEYAPDLRPL----------F--------------------KKDMAAQRQMLMSTLKLAVKGL--------DDLEQLTPVLRKLAQRHVDYG--------V-----KPGDYTPVGNALLWTLKQGLGEAS------------------------------------WTQELRAAWVDAFRLMATVMKQAAYPEIQPI**

**>Deshaf***

**ALNEAAAAVVRATVPALAA---------HGHAVTTAMYRRLFRDE-RIAAL----------FNHA----------------NQGGDNAQAHALAGAILAYAQHI--------DDLPALSGALERIAQKHVGYH--------I-----LPEHYPFVAGALLGAIEETLGEAA-------------------------------------TPQVLAAWGEAYWFLADLLQRREAELRGEL**

**>Glovio***

**TAMALQVKLLEQSFEGVKP---------NAHAFAASFYDNLFSDFPQTQAL----------FA-------------------HSDMQAQQQKLLASLVLVVENL--------RQPQVLSTALQDLGNRHAGYG--------I-----VPEHYPMVGTSLLKTFETYLGDAW-------------------------------------TPEVKQAWVDAYGAITGLMLTGAES-----**

**>Rhodopirell***

**DMTPEQVTLVKESWEKVKP---------ISEQAAELFYGRLFTLDPSLRSL----------F--------------------KGDMSEQGKKLMSTITLAVTSL--------DRLETILPTVQALGRKHAVEYE-------V-----PDSSYATVGEALIWTLGQGLGDDF-------------------------------------TEDVKEAWLLTYTTLSGAMLSGKSTAA---**

**>Vitste***

**MLDQQTINIIKATVPVLKE---------HGVTITTTFYKNLFAKHPEVRPL----------FD-M----------------GRQESLEQPKALAMTVLAAAQNI--------ENLPAILPAVKKIAVKHCQAG--------V-----AAAHYPIVGQELLGAIKEVLGDAA-------------------------------------TDDILDAWGKAYGVIADVFIQVEADLYAQA**

**>Pseaer***

**...mnaadrvmqsygrcc..........astgffddfyrhflasspqirak..........fA...................TTdmtaqkhllragimnlvmya..........rgmsdsklralgashsraald......i.....rpelydlwldallmavaehdrdc......................................daetrdawrdvmgrgiaviksyy.......**

**>Thefus***

**rpdlrtvslvqalcadlld.........qpevladrfyhhlfrllpscrdl..........f....................Pedmgeqhvrmarvlvevvnhl........DEPektwdtlrklgeyhyvrwg.......l.....gleeyrcvghalieaardislew......................................apsvgsawvtvyewivsamlsgaaeaaqAR**

**---------|--||--|----------------|--||--||---|---------------|--|--------------------|--||--||--|--||--------------------|--|---||-----------|---------|--|--|||-||---------------------------------------------------||--||--|---|-----------**

**EUKARYOTE FHbs**

**>Aspnid1**

**ALTPQQKQIVKATIPALEQ---------HGVTITTLFYKEMLERHPELNNI----------FNS-----------------SHQATGVQPAALAHAVWAYASNI--------DHPEALATAVSRIGHKHASLG--------V-----RSEHYPIVGEHLLRAIKKVLGDAV-------------------------------------TPPVADAWTAAYQQLADIFISFEDDLYKQA**

**>Debhan1**

**ELTPREKELIKASVPILEE---------SGDVLTSKFYNHMLTDFPEVKPF----------FNE-----------------SNQKTMKQPKILAFALLHYAKNI--------DDIAPLTAFVNQIVVKHVGLQ--------V-----KPEHYPIVGQCLLDTMETLLGPEIA------------------------------------TEEFLTAWATAYGNLAQILINAEAEKYKEM**

**>Dicdis1 ..**

**SLSQQSISIIKATVPVLQV---------HGVNITTTFYRNMFKANPQLLNI----------FNH-----------------SNQREGKQQNALANTVLQAAIHI----------DKLNELNLAPIVHKHVALG--------V-----LPEHYPIVGTNLLGAIKEVLQDAA-------------------------------------TDEILGAWGEAYGVIAQAFIDAEAALYKVT**

**>Gialam**

**TLSEDTLRAVEATAGLIAA---------QGIEFTRAFYERMLTKN-EELKNI---------FNLA-----------------HQRTLRQPKALLDSLVAYALNI--------AELQGFFSAAERVANKHTSFG--------I-----QPAQYQIVGAHLLATIEDRITK---------------------------------------DKDILAEWAKAYQFLADLFIKREEEIYAAT**

**>Neucra1**

**TLTDAQISIVKSTAPVLKQ---------HGEAITTVFYNDLITENPSLKNI----------FS-L----------------TSQATGAQPRALAHAVLAYATYI--------DNLSALSEAVARIAHKHVSLQ--------V-----EPAQYAIVGQYLIQAIGKVLGDAA-------------------------------------TPEIVDAWTAAYGVLANVFIGVEGGMYEEN**

**>Saccer1**

**MLAEKTRSIIKATVPVLEQ---------QGTVITRTFYKNMLTEHTELLNI----------FNR-----------------TNQKVGAQPNALATTVLAAAKNI--------DDLSVLMDHVKQIGHKHRALQ--------I-----KPEHYPIVGEYLLKAIKEVLGDAA-------------------------------------TPEIINAWGEAYQAIADIFITVEKKMYEEA**

**>Schpom1**

**ELNESQKQYIRSSIPILES---------SGVNLTKAFYQKMLGNYPEVLPY----------FNKA-----------------HQISLSQPRILAFALLNYAKNI--------DDLTSLSAFMDQIVVKHVGLQ--------I-----KAEHYPIVGHCLLSTMQELLPSDVA------------------------------------TPAFLEAWTTAYGNLAKILIDSEKKVYQSQ**

**---------|--||--|----------------|--||--||---|---------------|--|--------------------|--||--||--|--||--------------------|--|---||-----------|---------|--|--|||-||---------------------------------------------------||--||--|---|-----------**

**VERTEBRATE gbs**

**>Homo Ngb**

**-MERPEPELIRQSWRAVSR---------SPLEHGTVLFARLFALEPDLLPL----------FQ-Y----NCRQFSSPEDCLSSPEFLDHIRKVMLVIDAAVTNV--------EDLSSLEEYLASLGRKHRAVG--------V-----KLSSFSTVGESLLYMLEKCLGPA--------------------------------------TPATRAAWSQLYGAVVQAMSRGWDGE----**

**>Gallus Ngb**

**MLSRTQQALIRESWRRVSG---------SPVQHGVVLFSRLFDLDPDLLPL----------FQ-Y----NCKRFASPQECLAAPEFLDHIRKVMLVIDAAVSHL--------EDLPCLEEYLCNLGKKHQAVG--------V-----KVESFSTVGESLLYMLEKCLGAA-------------------------------------FSPDVREAWIELYSAVVKAMQRGWEVLPEGD**

**>Danio Ngb**

**KLSEKDKGLIRDSWESLGK---------NKVPHGIVLFTRLFELDPALLTL----------FS-Y-----STNCGDAPECLSSPEFLEHVTKVMLVIDAAVSHL--------DDLHTLEDFLLNLGRKHQAVG--------V-----NTQSFALVGESLLYMLQSSLGPA-------------------------------------YTTSLRQAWLTMYSIVVSAMTRGWAKNGEHK**

**>Homo Cygb**

**ELSEAERKAVQAMWARLYAN---------CEDVGVAILVRFFVNFPSAKQY----------FSQF------KHMEDPLEMERSPQLRKHACRVMGALNTVVENL--------HDPDKVSSVLALVGKAHALKHK-------V-----EPVYFKILSGVILEVVAEEFASD-------------------------------------FPPETQRAWAKLRGLIYSHVTAAYKEVGWVQ**

**>Gallus Cygb**

**EISDAEKKVIQETWSRVYAN---------CEDVGVSILIRFFVNFPSAKQY----------FSQF------KHMDDTLEMERSLQLRKHAQRVMGAINTVVENL--------DDPEKVSSVLALVGKAHALKHK-------V-----EPVYFKKLTGVMLEVIAEAYGND-------------------------------------FTPEAHGAWTKMRTLIYTHVTAAYKEAGWVS**

**>Danio Cygb1**

**SLTEEDVCVIQDTWKPVYAE---------RDNAGVAVLVRFFTNFPSAKQY----------FEHF------RELQDPAEMQQNAQLKKHGQRVLNALNTLVENL--------RDADKLNTIFNQMGKSHALRHK-------V-----DPVYFKILAGVILEVLVEAFPQC------------------------------------FSPAEVQSSWSKLMGILYWQMNRVYAEVGWEN**

**---------|--||--|----------------|--||--||---|---------------|--|--------------------|--||--||--|--||--------------------|--|---||-----------|---------|--|--|||-||---------------------------------------------------||--||--|---|-----------**

**>HBA_HUMAN**

**>Homo A**

**VLSPADKTNVKAAWGKVGA---------HAGEYGAEALERMFLSFPTTKTY----------FPHF------------DLSHGSAQVKGHGKKVADALTNAVAHVD-----------DMPNALSALSDLHAHKLR-------V-----DPVNFKLLSHCLLVTLAAHLPAE-------------------------------------FTPAVHASLDKFLASVSTVLTSKYR------**

**>HBA_SQUAC**

**>Squalus A**

**VLSAADKTAIKHLTGSLRT---------NAEAWGAESLARMFATTPSTKTY----------FSKF-----------TDFSANGKRVKAHGGKVLNAVADATDHLD-----------NVAGHLDPLAVLHGTTLC-------V-----DPHNFPLLTQCILVTLAAHLTE--------------------------------------LKPETHCALDKFLCEVATALGSHYR------**

**HBAD_ANSAN**

**>Anser A**

**-LTADDKKLLAQLWEKVAG---------HQDEFGNEALQRMFVTYPQTKTY----------FPHF------------DLHPGSEQVRSHGKKVAAALGNAVKSLD-----------NISQALSELSNLHAYNLR-------V-----DPANFKLLSQCFQVVLAVHLGKD-------------------------------------YTPEMHAAFDKFLSAVAAVLAEKYR------**

**HBB_HUMAN**

**>Homo B**

**HLTPEEKSAVTALWGKV-----------NVDEVGGEALGRLLVVYPWTQRF----------FESF------GDLSTPDAVMGNPKVKAHGKKVLGAFSDGLAHLD-----------NLKGTFATLSELHCDKLH-------V-----DPENFRLLGNVLVCVLAHHFGKE-------------------------------------FTPPVQAAYQKVVAGVANALAHKYH------**

**HBB_ANSAN**

**>Anser B**

**HWSAEEKQLITGLWGKV-----------NVADCGAEALARLLIVYPWTQRF----------FSSF------GNLSSPTAILGNPMVRAHGKKVLTSFGDAVKNLD-----------NIKNTFAQLSELHCDKLH-------V-----DPENFRLLGDILIIVLAAHFAKE-------------------------------------FTPECQAAWQKLVRVVAHALARKYH------**

**>HBB_SQUAC**

**>Squalus B**

**HWTGEEKALVNAVWTKT-----------DHQAVVAKALERLFVVYPWTKTY----------FVKF----------NGKFHASDSTVQTHAGKVVSALTVAYNHID-----------DVKPHFVELSKKHYEELH-------V-----DPENFKLLANCLEVELGHALHKE-------------------------------------FTPEVQAAWSKFSNVVVDALSKGYH------**

**MYG_HUMAN**

**>Homo Mb**

**GLSDGEWQLVLNVWGKVEA---------DIPGHGQEVLIRLFKGHPETLEK----------FDKF------KHLKSEDEMKASEDLKKHGATVLTALGGILKKKG----------HHEAEIKPLAQSHATKHK--------I-----PVKYLEFISECIIQVLQSK-------------------------------------HPGDFGADAQGAMNKALELFRKDMASNYKELGFQG**

**MYG_THUAL**

**>Thunnus Mb**

**----ADFDAVLKCWGPVEA---------DYTTMGGLVLTRLFKEHPETQKL----------FPKF-------AGIAQADIAGNAAISAHGATVLKKLGELLKAKG-----------SHAAILKPLANSHATKHK-------I-----PINNFKLISEVLVKVMHEK---------------------------------------AGLDAGGQTALRNVMGIIIADLEANYKELGFSG**

**MYG_CHICK**

**>Gallus Mb**

**GLSDQEWQQVLTIWGKVEA---------DIAGHGHEVLMRLFHDHPETLDR----------FDKF------KGLKTPDQMKGSEDLKKHGATVLTQLGKILKQKG-----------NHESELKPLAQTHATKHK-------I-----PVKYLEFISEVIIKVIAEK-------------------------------------HAADFGADSQAAMKKALELFRNDMASKYKEFGFQG**

**HYPEROARTIA (AGNATHAN, LAMPREY) HBS**

**Petromyzon marinus 150aa Globin II Q9I9I3 GI:14194759**

**>Petmat Hb2**

**plsaaektkirsawapvys---------nyetsgvdilvkfftstpaaqef----------fpkf------kglttadqlkksadvrwhaeriinavndavvsm--------ddtekmsmklrdlsgkhaksfq-------v-----dpqyfkvlaaviadtvaag------------------------------------------dagfeklmsmicillrsay-----------**

**HYPEROTRETI (MYXINE) HBS**

**Myxine glutinosa 148aa Globin III P02209 GI:121224**

**>Myxglu Hb3**

**tlsegdkkaireswpqiyk---------nfeqnslavlleflkkfpkaqds----------fpkf--------sakkshleqdpavklqaeviinavnhtiglm--------dkeaamkkylkdlstkhstefq-------v-----npdmfkelsavfvstmggk-------------------------------------------aayeklfsiiatllrstyda---------**

**UROCHORDATE HBS**

**>Cioint 1**

**PFTDEELKLLRDSWDEVKK--------LGMKEVGLHIFTGLLNAAPSLRTL----------FYTI--DLPDEEELTIDVMRENKKVVAHATRIANAISKFIKFLD--------QPDELEKLLTSLGESHARRQ--------V-----DPESFEYVAPVILSVIGGHLKL-------------------------------------PSNSPTLQAWVKAYGVLRNGIVSAMEA-----**

**ECHINODERM HBS**

**Caudina arenicola gi|999806|1HLM HbC 159aa**

**>Cauare HbC**

**dltpaekdlirstwdqlmt---------hrtgfvadvfirifhndptaqrk----------fpqm-------aglspaelrtsrqmhahairvsalmttyidemdte---------vlpellatltrthdknh--------v-----gkknydlfgkvlmeaikaelgv-------------------------------------gftkqvhdawaktfaivqgvlitkhas-----**

**---------|--||--|----------------|--||--||---|---------------|--|--------------------|--||--||--|--||--------------------|--|---||-----------|---------|--|--|||-||---------------------------------------------------||--||--|---|-----------**

**ANNELID HBS EXTRACELLULAR**

**Lumbricus terrestris B28151 Lum II(b)**

**>Lumter b**

**QCGVLEGLKVKSEWGRAYG-------SGHDREAFSQAIWRATFAQVPESRSL---------FKRV-----------HGDDTSHPAFIAHAERVLGGLDIAISTL--------DQPATLKEELDHLQVQHEGRK--------I-----PDNYFDAFKTAILHVVAAQLGR----------------------------------------CYDREAWDACIDHIEDGIKGHH-------**

**Lumbricus terrestris C28151 LumIII(c)**

**>Lumter c**

**CCSEEDHRIVQKQWDILWR---DTESSKIKIGFGRLLLTKLAKDIPDVNDL----------FKRV-----------DIEHAEGPKFSAHALRILNGLDLAINLL--------DDPPALDAALDHLAHQHEVREG-------V-----QKAHFKKFGEILATGLPQVLDD-----------------------------------------YDALAWKSCLKGILTKISSRLNA-----**

**Macrobdella decora 164 aa chain B BAC82447.1 GI:34330145**

**>Macdec B**

**ecnllhrfkfykqwtqvfg------lgedridfgvkvfnklfqdhpdarkl----------ftrv-----------ngenvyshefkahikrvlssldlnalll--------skdallekqlahlkgqhdsrg--------v-----dwsyvqafkqaildvlpeylgV----------------------------------------fvsfeawdgclehiltgifkgh-------**

**Macrobdella decora 168 aa chain C BAC82448.1 GI:34330147**

**>Macdec C**

**ecssadghkvledwthlwe----dsdasfkvefaksvllkliekhpeakal----------fhev-----------gvedpssgefeahvlrifnsldllitll--------pnreglkaasehlgqqhavrhg-------v----vgthfktflsvlldsldhlldd------------------------------------------fhlfawksclktltkgivdavpq-----**

**Sabella spallanzanii 159 aa globin 3 CAC37412.1 GI:13810249**

**>Sabspa Glb3**

**gcnilqrlkvknqwqeafg------yaddrtsLgtalwrsiimqkpesvdkf---------fkrv-----------ngkdisspafqahiqrvfggfdmcisml-----------ddsdvlasqlahlhaqhverg-----i-----saeyfdvfaeslmlavestie----------------------------------------scfdkdawsqctkvissgigsgv-------**

**Sabella spallanzanii 166 aa globin 2 CAC37411.1 GI:13810247**

**>Sabspa Glb2**

**ccsmedrqevlkaweamws----aeftgrrviiaqevfqrlfekspetkel----------ftgv-----------nvanidspefrahcvrvtngfAtminma--------fdsdtlskqldhlgnqhtkyag-------m-----raeylrlfrqsfaevlpqaip-----------------------------------------cfntaawnrcitamqdvmgaslaa-----**

**Tylorrhynchus heterochaetus**

**148 aa Extracellular globin IIB P13578 GI:121226**

**>Tylhet 2B**

**ccsaadrhevldnwkgiws----aeftgrrvaigqaifqelfaldpnakgv----------fgrv-----------nvdkpseadwkahvirvingldlavnll--------edpkalqeelkhlarqhrersg-------v-----kavyfdemekallkvlpqvss-----------------------------------------hfnsgawdrcftriadvikaelp------**

**Tylorrhynchus heterochaetus 146 aa Extracellular globin IIA P09966 GI:121218**

**---------|--||--|----------------|--||--||---|---------------|--|--------------------|--||--||--|--||--------------------|--|---||-----------|---------|--|--|||-||---------------------------------------------------||--||--|---|-----------**

**>Tylhet 2A**

**hcgplqrlkvkqqwakayg------vghervelgialwksmfaqd-ndardl---------fkrv-----------hgedvhspafeahmarvfngldrvissl--------tdepvlnaqlehlrqqhiklg--------i-----tghmfnlmrtglayvlpaqLG----------------------------------------rcfdkeawaacwdeviypgikhd-------**

**ANNELID HBS INTRACELLULAR**

**Alvinella pompejana 124 aa intracellular haemoglobin CAI56311.1 GI:62990085**

**>Alvpom**

**------adniaavrgdvst-------------hamnifveyfkkfpqhqna----------fady-------kgkdpeslkslpkfkthttkvvsklldiveka-----sdsgalqsncttlakm-pqhkg----------l-----nqqqfadlgavlvpylqkalg----------------------------------------gacdsaaweqayn-----------------**

**Amphitrite ornata 137 aa 1EW6_A GI:8569636**

**>Amphorn**

**----gfkqdiatirgdlrt-------------yaqdiflaflnkypderry----------fkny-------vgksdqelksmakfgdhtekvfnlmmevadra-----tdcvplasdantlvqm-kqhss----------l-----ttgnfeklfvalveymrasgq-----------------------------------------sfdsqswdrfgknlvsalssagmk-----**

**Arenicola marina 133aa myoglobin IIa CAI56310.1 GI:62990083**

**>Aremar Mb**

**-adqmaavkaniaavkagd----------vtktagdffvflfkkfpalqdk----------fpny-------kgksvdslssvatfaphttkvvaavldlvaka--------gdagvlagaakqvvadhvsrgv-------v-----sgaeytdlfaalvpflaaalg----------------------------------------gacdqaawtaatg-----------------**

**Glycera dibranchiata 147 aa Globin P1 P23216 GI:121399**

**>Glydib P1**

**hltadqvaalkaswpevsa-------gdggaqlglemftkyfhenpqmmfi----------fg-y--------sgrtealkhssklqhhgkviidqigkavaem--------dnakqmagtlhalgvrhkgfgd-------i-----raeffpalgmclldameekvp-----------------------------------------glnrtlwaaayreisdaciaglqs-----**

**Glycera dibranchiata 148 aa Globin, monomeric component M-IV (GMH4) P15447 GI:121228**

**>Glydib M4**

**glsaaqrqvvastwkdiag-------sdngagvgkecftkflsah-hdiaav---------fg-f---------sgasdpgvadlgakvlaqigvavshlgdegk------------mvaemkavgvrhkgygykh-----i-----kaeyfeplgasllsamehrig-------------------------------------gkmtaaakdawaaayadisgalisglqs-----**

**Aphrodite aculeata 151 aa nerve myoglobin AAC47259.1 GI:1491803**

**>Aphacu Ngb**

**aglsgadiavirstwakvq------gsgsatdigrsifikffeldpaaqne----------fp-c-------kgeslaalktnvllgqhgakfmeyittavngld-------dyagkahgpltelgsrhktrgttpan---f-------gkageallailasvvggdf----------------------------------------tpaakdawtkvyntisstmqaal-------**

**---------|--||--|----------------|--||--||---|---------------|--|--------------------|--||--||--|--||--------------------|--|---||-----------|---------|--|--|||-||---------------------------------------------------||--||--|---|-----------**

**INSECT HBS**

**Anopheles gambiae gi|57914327|XP_555006.1 ENSANGP00000026474 182aa (5-159)**

**>Anogam**

**GLTASEKITLFSAWGLIRK.........DLDVHGRNVLLLLFHKHPRYIAY..........FD.F......TDDPNAQSLVDNKSLYDQAIHVFKAVGALIEYG.......FKDPVLFDATLRKITRRHKDRP........V.....YTEDILTIGEVLLNYLEQALG.....................................RQMSDSLPDAFWKLFQTIAGRFPATPKTPIADE**

**Drosophila melanogaster gi|5731771|CAB52584|CG9734-PA 153aa**

**>Dromel**

**.mnsdevqlikktweipvat.........ptdsgaailtqffnrfpsnlek..........fp.f.......rdvpleelsgnarfrahagriirvfdesiqvl-----gqdgdlekldeiwtkiavshiprt........v.....skesynqlkgvildvltaacs.......................................Ldesqaatwaklvdhvygiifkaidddgnak**

**Gasterophilus intestinalis gi|3885490|AAC80435.1 151aa**

**>Gasint**

**.mnseevndikrtwevvaa.........kmteagvemlkryfkkyphnlnh..........fpwf.......keipfddlpenarfkthgtrilrqvdegvkal-----svdfgdkkfddvwkklaqthhekk........v.....errsynelkdiiievvcscvk.......................................Lnekqvhayhkffdraydiafaemakmg...**

**Chironomus thummi CTT-IX NF00002191 145aa**

**>Chithu CTT9**

**PVSSDEANAIRASWAGVKH-------------NEVDILAAVFSDHPDIQAR----------FPQF-------AGKDLASIKDTGAFATHAGRIVGFISEIVALV-----GNESNAPAMATLINELSTSHHNRG--------I-----TKGQFNEFRSSLVSYLSSHAS---------------------------------------WNDATADAWTHGLDNIFGMIFAHL-------**

**Tokunagayusurika akamusi HbV NF00226726 152aa**

**>Tokaka Hb5**

**GLSDSEEKLVRDAWAPIHG--------DLQGTANTVFYNYLKKYPSNQDK-----------FETL-------KGHPLDEVKDTANFKLIAGRIFTIFDNCVKNV--------GNDKGFQKVIADMSGPHVARP--------I-----THGSYNDLRGVIYDSMHLDST-------------------------------------------HGAAWNKMMDNFFYVFYECLDGRCSQF**

**---------|--||--|----------------|--||--||---|---------------|--|--------------------|--||--||--|--||--------------------|--|---||-----------|---------|--|--|||-||---------------------------------------------------||--||--|---|-----------**

**MOLLUSC HBS**

**Aplysia limacina 147aa Mb P02210 GI:3915701**

**>Aplysia Mb**

**slsaaeadlagkswapvfan---------kdangdaflvalfekfpdsanf----------fadf-------kgksvadikaspklrdvssriftrlnefvnna--------adagkmsamlsqfakehvgfg--------v-----gsaqfenvrsmfpgfvasvaa---------------------------------------ppagadaawtklfgliidalkaagk------**

**.........|..||..|................|..||..||...|...............|..|....................|..||..||..|..||....................|..|...||...........|.........|..|..|||.||............-----------------------------..........||..||..|...|...........**

**Barbatia lima 308 aa two-domain hemoglobin S61518 GI:2147456 DOMAIN 1**

**>Barlim D1**

**vtqpvnkslirqtwnmmag----------dskngvdlmallfqiapeskke----------ftrl-------gdvspenipnnrklnghgitlwyaltsfvdql--------dspndledlcrkfavnhvarg--------v-----ldvrfgwikeplakllrrncg---------------------------------------ncdeaiqawwelidvicavvkenkmsvaeki**

**Barbatia lima 308 aa two-domain hemoglobin S61518 GI:2147456 DOMAIN 2**

**>Barllim D2**

**atqsdnksliretwemiag----------drkngvelmallfemapeskke----------frrl-------gdispenipnnrklnghgitlwyaltsfvdql--------dnkndledlcrkfavnhvsrg--------v-----lnvkfgwikeplaellrrkcg-------------------------------------srcedrhinawwklidvicaileeqk-------**

**Barbatia lima 158aa delta chain S61519 GI:2118933**

**>Barlim Delta**

**atqpgnrnlireswnmmaa----------drkngvelmrllfemapeskke----------ftrl-------gdispanipynrklnghgitlwyalmsfvdhl--------dspndledvcrkfavnhvarn--------v-----lddkfawikkpleallknkcn---------------------------------------ckqdvvnawcklidvicavlreg--------**

**Biomphalaria glabrata 151 aa myoglobin AAC24318.1 GI:3253145**

**>Biogla Mb**

**slsdadkkaldaswkklta------gadgkknaginlvlwmfanvpnmraq----------fskf------nanqsddalkgdaefikqvnvivaaldgllqsv--------nnpgqlqanldklakshvnlk--------i-----gleffgplqqnihsfiesalgv-------------------------------------gagsdepkawgnliaafnetlkka--------**

**Biomphalaria glabrata 388aa hemoglobin CAH23232.1 GI:51773699 domain I (73-**

**>Biogla D1**

**glsetdrraldsswkrlta------gengvqkagvnlvlwffnnipnmrer----------ftkf------danqaddalradpefqkqvnvivgglksfldsv--------ndpialqanmdrvaeahlsmdpV------v-----gvpyfsalsqnihrfieislgV-------------------------------------tadsdesqawtdllagftrvvrnravlrkv--**

**Biomphalaria glabrata 388aa hemoglobin CAH23232.1 GI:51773699 domain I (73-**

**>Biogla D1**

**kvsdsdksafvsswnelir------kaasrrnagvnlvlwlfnnvpnmrnh----------ftkf------ngnqpdaalrndqeflnqvdriaggleslvknv--------nnparfldalerlssahlnmkps------i-----gleyfgplqqnihtyiesalgva-------------------------------------agsdeanawtdvfgafneilkyssvek----**

**Calyptogena nautilei 141 aa hemoglobin III BAD34605.1 GI:50897143**

**>Calnau Hb3**

**mvtiadvknvqtswnsikd--------kwetdhglnfyttlfddvpeiksa----------fvka-------------gnktdyqvkgqavrfgrmvtewidnl--------dneealvakingmcathrtrg--------i----tnvdlfevalgelvkyiagktsft---------------------------------------kaqreswavvngciiqtmrnyf-------**

**Calyptogena nautilei 134 aa hemoglobin IV BAD34606.1 GI:50897145**

**>Calnau Hb4**

**mvtaeekalvqqtwalvyc--------tweskngvkfyekmfaastpikea----------fesf-------------gttnieemkrqailfgdmlnsfilal--------ndedalkckiqgmiathkkrn--------i----rnmglfkvamqqliafvgt----------------------------------------------ekaawtsvtdailaqmktnl-------**

**Calyptogena soyae 145 aa hemoglobin I BAD34603.1 GI:50897139**

**>Calsoy Hb1**

**mvsandiknvqdtwgklyd--------qwdavhaskfynklfkd-sedisea---------fvka-------------gtgsgiamkrqalvfgailqefvanl--------ndptaltlkikglcathktrg--------i----tnmelfafaladlvaymgttisft---------------------------------------aaqkaswtavndvilhqmssyfatv----**

**Calyptogena soyoae 137 aa hemoglobin II BAD34604.1 GI:50897141**

**>Calsoy Hb2**

**mvsqadiaavqtswrrcyc--------swdnedglkfyqtlfdsnskirha----------fesa-------------gatndtemekqanlfglmmtqfidnl--------ddttalnykisglmathktrn--------v----vdpalfaialnelvkfiGNq----------------------------------------------qpawknvtavilsqmkialssn----**

**Cerithidea rhizophorarum P02215 GI:121269 GLB_CERRH 151aa**

**>Cerrhi**

**SLQPASKSALASSWKTLAK------DAATIQNNGATLFSLLFKQFPDTRNY----------FTHF-------GNMSDAEMKTTGVGKAHSMAVFAGIGSMIDSM--------DDADCMNGLALKLSRNHIQRK--------I-----GASRFGEMRQVFPNFLDEALGGG-------------------------------------ASGDVKGAWDALLAYLQDNKQAQAL------**

**Liolophura japonica 145 aa myoglobin I AAB25285.1 GI:298242**

**>Liojap Mb**

**gisadqakalkddiavvaqnpn---------gcgkalfikmfemnpgwvek----------fpaw-------kgksldeikasdkitnhggkvinelanwinni-----------nsasgilksqgtahkgrs--------i-----gieyfenvlpvidatfaqqmgGA-----------------------------------------ytaamkdalkaawtgvivpgmka----**

**Lucina pectinata 142 aa Hemoglobin I 1EBT GI:6980602**

**>Lucpec Hb1**

**slsaaqkdnvtsswakasa---------awgtagpeffmalfdah-ddvfak---------fsgl------fsgaakgtvkntpemaaqaqsfkglvsnwvdnl--------dnagalegqcktfaanhkarg--------i-----sagqleaafkvlsgfmksyGG-------------------------------------------degawtavagalmgeiepnm-------**

**Nassa mutabilis 147 aa (Myoglobin) P31331 GI:399542**

**>Nasmut Mb**

**glsaeqktalkdswkilaa------ngetmvknsaamfgllfekypdtkkh----------fktf-------dgdhfaamkatgmgkahgmsvfsglgalvssv--------ddgecvlglakklsrnhtarg--------v-----tandfklmrsifgefldkatgg-------------------------------------katesmksawdallgvlienhq----------**

**Yoldia eightsi AAM00251.1 GI:19880109 147aa**

**>Yoleig Mb**

**sfsaaqvdtvrsnwcsmta---------didaagyrifellfqrnpdyqsk----------fkaf-------kglavsalkgnpnaekhirivlgglgrilgal---------ntpeldviykemasnhkprg--------v-----mkqqfkdmgqaivtalseiqs-------------------------------------ksggsfdratwealfesvangigqyq-------**

**NEMATODE HBS**

**Ascaris suum D1 gi|159663|AAA29374.1 383aa (24-174)**

**>Ascsuu D1**

**Lcmkslehakvdtsnearq-------------dgidlykhmfenypplrky----------fkn-------reeytaedvqndpffakqgqkillachvlcatyd--------dretfnaytrelldrhardh--------v---hmppevwtdfwklfeeylgkkttL---------------------------------------deptkqawheigrefakeinkhgrh-----**

**>Ascsuu D2**

**-avrhqcmidighsetakq-------------ngidlykhmfenypsmrea----------fkd-------renytaedvqkdpffvkqgqrillachllcasyd--------deetfhmyvhelmerherlg--------v-----qlpdqhwtdfwklfeeflekkshl-------------------------------------cehtkhawavigkefayeatrhgkeh----**

**Ascaris suum 153aa Myoglobin (Globin, body wall isoform)P49672 GI:1346136**

**>Ascsuu Mb**

**-----matacvkslesvqc-----gtcektiangtefyallfdkhpdlrhy----------fkg-------nenltgadvkksdhfkkqgqrlllachvlahlen--------dpasfkayareivdphlrms--------v---hlepklwsefwpiwldylstkesv---------------------------------------ddatknawlalgkkfsdecldhlknl----**

**Mermis nigrescens gi|7106745|AAF36101.1 147aa Eye glb**

**>Mernig eye gb**

**mvvnldilraqlaklpine------------fngpkfyvhmfssqpdwrny----------fkg-------seaikpeevptcprflrqgqrvllsmelmiela--------dkpqlfdayvrdlldkhkqikg-------i-----dydlysaffdvwygylskiigM---------------------------------------sdkekkeweafrvelflpavkkyiag----**

**Syngamus trachea cuticle globin gi|18029239|AAL56426.1 180aa (27-180)**

**>Syntra**

**maaadvkkhvvdslvnvpl---------gndqtgkdfykyfftnhpdlrky----------fkgf-------etftaddvqksekfrtlgnafilaihivanty--------dnepvfrayvrdniarhverg--------l-----epslwkdfwkiwtafleskgt--------------------------------------tlsaddkaawealrdrfndesqkelakr----**

**Toxocara canis**

**gi|18029247|AAL56429.1 153aa**

**>Toxcan**

**mataclkslesaqcgtcdk----------aiengtgfyallfdkhpelrhy----------fkg-------nenltgaevkksehfkkqgqtlllachvlahle--------ndpssfnaycreiidrhlranvh------l-----dpklwtafwpiwldylatkttV---------------------------------------ddatknawlalgkkfadeccnhlkns----**

**CRUSTACEAN HBS**

**Artemia salina 1430 aa hemoglobin C1 polymer domain 1AAC96001.1 GI:4020134**

**>Artsal D1**

**gilcsdkatikrtwsivnd----------lpsfgrnvflsvfaakpeyknl----------fvef-------rnipaselanserllyhggrvlasidevisei--------dspdsaakklvalgerhitrgt-------v------rrhfeafsyafidelkqrgvA----------------------------------------sadlaawrkgwdsivdileagllkr----**

**>Artsal D2**

**glscvdvanvqeswatvsa---------nlkntgsilfqrlindhpeyqql----------frqf-------rdvelaklgesngfvahvfrvvaafdgiikel--------dnnpfivstlkrlgeqhiargtd------i-------shfqnfrttllvylnengmn----------------------------------------qaqeaswnkafdaiekyisiglksl----**

**>Artsal D3**

**glsgleknailntwgkvrg---------nlqevgkatfgklfaahpeyqqm----------frff-------qgvqlaelvdspkfaahtqrvvsaldqtllal--------nrpsdfvymikelgldhinrgtdrsh---f-------enyqvvfveylketlgdsvde----------------------------------------ftvksfnhvfeviinflneglrqa----**

**>Artsal D4**

**hltgrqkeaikaswsvart---------dlrflgqelfmrmfnlnpeyqsl----------fvn-----kgfadvplvslrederfishmanvlrgfdtllqnl--------ddtsyfvyalrnlgdahiqrkagteh---f-------rsfeailipylqesqgldaa------------------------------------------gveawkiffdvsigviaqglkva----**

**>Artsal D5**

**glygkevvalrqafaaisp---------rnveigkrvfaklftshpeyknl----------fkkf-------eqysveelpstdafdyhislvmnrfsavgkvi--------ddnvsfvyllkklgrehikrglsrkq---f-------dqfvelyiaeispelsetgr----------------------------------------sglekvltfatgvieqglfqlGQVD----**

**>Artsal D6**

**altalekqsiqdiwtslrp--------tgleelavkmftrlfadhpeykll----------ftg--------rlgnvdninenapfrahlhrvlsafdivitsl--------dnnallirqlkdlglfhtrlgmtrah---f-------dnfataffsvaedivpnllt------------------------------------algreslgkgfklmvavieegllqlerid----**

**>Artsal D7**

**glsvrevevvkqtwnlvkp---------dlmgvgmrifkslfekfpayqav----------fpkf-------sdvpldkledipavgkhaisvttkldeliqtl--------depanlallarqlgedhivlgvnkpm---f-------ksfgevlvrllendlgqrfs----------------------------------------nfaskswhraydviveyieeglqqs----**

**>Artsal D8**

**gitdaekvlvqrswellkp---------dllglgrkifgviftkhpeyqil----------ftrv-----gfgdtpltqldnnpafgehiikvmrafdyvirnl--------gkpktllaylknvgadhiarnverrh---f-------qafsealipvmqrelkaqlk----------------------------------------peavaawrkgldriigvidqgllgl----**

**>Artsal D9**

**Afsaadieaiqktwalakp---------dlmgkgasvfrqlftdh-gyqpl----------fsnl-------veyevtglegspelntharnvmaqldtlvgsl--------qnsielgkslnqlgkdhvprk--------v-----NKVhfddfaehfvplmkanlg-------------------------------------deftplaesawkkafnvmvatieqgqrar----**

**Daphnia magna 348aa hemoglobin BAA76874.1 GI:4589710 domain 1**

**>Daphmag D1**

**ilsanerslirktwdqakk----------dgdvapqvlfrfikahpeyqkk----------fskf-------advpqselltngnflaqaytilaglnvviqsl--------ssqellanqlnvlggahqprg--------v-----tpamfeefgviveqvleeelg-------------------------------------ngfsaearqawkhgihalvagvsktlknp----**

**Daphnia magna 348aa hemoglobin BAA76874.1 GI:4589710 domain 2**

**>Daphmag D2**

**kltlhqirdvqrswenirs---------grnalvssifvklfketprvqkh----------fakf-------ssvavdslaghadyekqvalvadrldtiisam--------ddklqllgniyymkythlerg--------i-----srdtfedfgrllldvlgasgv----------------------------------------ssddldswkgvlavfvngvspkk-------**

**---------|--||--|----------------|--||--||---|---------------|--|--------------------|--||--||--|--||--------------------|--|---||-----------|---------|--|--|||-||---------------------------------------------------||--||--|---|-----------**

**NEMERTEAN HBS**

**Cerebratulus lacteus gi|23821573|O76242|GLBN_CERLA 110aa**

**>Cerelact Ngb**

**--------------------------mvnwaavvddfyqelfkahpeyqnk----------fg-f-------kgvalgslkgnaayktqagktvdyinaaiggs---------------adaaglasrhkgrn--------v-----gsaefhnakaclakacsahga-----------------------------------------pdlghaiddilshl---------------**

**PLATYHELMINTH HBS**

**Clonorchis sinensis 150aa AAN28366.1 GI:23344091**

**>Clonsin**

**-maplskdevdalfeelnp---lvstteqrtefgkavymalfsaypeyiql----------ftkm-------qgltkdnveasegikyygqtfadsilemlqca--------sddgkleavleksgkehitrn--------v-----tkqqflsaeevfikhfsgvlt----------------------------------------keenkqsmerflkhivpkvagflg------**

**Paramphistomum epiclitum 147 aa P80721 GI:2494781**

**>Parepi**

**---tltkheqdillkelgp---hvdtpahivetglgayhalftahpqyiih----------fsrl-------eghtienvmqsegikhyartlteaivhmlke---------sndaevkkiaaqygkdhtsrk--------v-----tkdefmsgepiftkyfqnlvk----------------------------------------daegkaavekflkhvfpmmaaei-------**

**---------|--||--|----------------|--||--||---|---------------|--|--------------------|--||--||--|--||--------------------|--|---||-----------|---------|--|--|||-||---------------------------------------------------||--||--|---|-----------**

**CAENORHABDITIS ELEGANS**

**>ZK637.13**

**-MSMNRQEISDLCVKSLEG--RMVGTEAQNIENGNAFFRYFFTNFPDLRVY----------FKGA-------EKYTADDVKKSERFDKQGQRILLACHLLANVY--------TNEEVFKGYVRETINRHRIYK--------M-----DPALWMAFFTVFTGYLESVGC---------------------------------------LNDQQKAAWMALGKEFNAESQTHLKNS----**

**>F49E2.4**

**EITDEEVTAIRDVWRRAKT-----------DNVGKKILQTLIEKRPKFAEY----------FG-I-------QSESLDIRALNQSKEFHLQAHRIQNFLDTAVG----SLGFCPISSVFDMAHRIGQIHFYRG--------V---NFGADNWLVFKKVTVDQVTTGTTDSSKEKE-D-TNSNGTANGKVDTDASLIPIADINNVYSGENCLARLGWNKLMTVIVREMKRGFLEE----**

**---------|--||--|----------------|--||--||---|---------------|--|--------------------|--||--||--|--||--------------------|--|---||-----------|---------|--|--|||-||---------------------------------------------------||--||--|---|-----------**

**>C52A11.2**

**ALNKKDRTLLRETWQRLDD---------PKDIVGLIFLDIVNDIEPDLKKV----------FG-V-------DRAPRAAMLKMPKFGGHILRFYEFMEQLTSML-----GTSENLTGAWQLVRKTGRSHVRQGFLEQNQNQM-EKNYFEIVINVFIERLIPFLTGEQE---------------------LPSSEGKENKKVRFAQNYTTSQITDVWKKFLNTVISQMTDSFELE----**

**>C28F5.2**

**SLTFSQKQALNLSWRLLKP---------QASACFRKIFLELEIASPKVKQIFYKAALVDA-FNK--------------DDDNSATMEVHIKLTTKFFDELLVSL--------DDETEFVNKIRGIGSAHAILAKGSN----F-----SSDIWERLGEIAMERVCSHEV-------------------------------------VTKTREASRAWRTLIAILIDELRGGFEGE----**

**>W01C9.5**

**QLTPSQVSVVRRSWRHINT--------KGLIIVLTRCFSRLESNCPIVSQC----------FQSA-------TYSLSTNPNGVRTVADHAKYLLQLLDKIIEGD------------VDSEFLREIGANHVCLKHESG----F-----STQEWDRFQEIMVEVILKQDG-------------------------------------VKQSKETSRAWRLLICSFIELIRDGFDAQ----**

**>C29F5.7**

**PLNAKTKKLVIQEWPRVLA---------QCPELFTEIWHKSATRSTSIKLA----------FGIA-----ENESPMQNAAFLGLSSTIQAFFYKLIITYELNDD------------QVREACEQLGARHVDF---------I-SRGFNSHFWDIFLVCMAEKIDETLS--------------------------------AYIPDEDKRNEMILAWQRVINSIVHQMRNGYSDR----**

**>F19H6.2**

**FLTRRERILLEQSWRKTRK--------TGADHIGSKIFFMVLTAQPDIKAI----------FG-L-------EKIPTGRLKYDPRFRQHALVYTKTLDFVIRNL--------DYPGKLEVYFENLGKRHVAMQGRG-----F-----EPGYWETFAECMTQAAVEWEA--------------------------------------NRQRPTLGAWRNLISCIISFMRRGFDEE----**

**>R01E6.6**

**PISAQGREIITQCFENPHS------------EFANKVVQRIFEKR-EDYQKYIMN------L-------------GKERSSIVNNRLKQLVEDIVAHIHDADFI--------------ESVSKQYGEEHVELKQYG-----F--KPDFWVAVADAMTLEGVILDMANQ---------------------------------------HPADTVSAWSSLVTMIFSSVRDGYYSE----**

**>F46C8.7**

**RLSKIQKRAIRFTWHRLQT----RNGGKRVENVFEEVFDKLVKNLPNIRDM----------FST-------RMFLCAMSRGTTSTLRDHSKNCVKMIDSVIKNF--DVEKSKRTDTSSENDPRVIGRAHSILKPYG-----L-----AGNYWEKFGEVMIDVVLAQEA-------------------------------------VRDLPGAGQAWVIFTACLVDQMRAGFDEN----**

**>R13A1.8**

**LIDKESCEVVADSWRLVES---RSSAAETSACFGLFVFQRVFSKIPMLRPL----------FG-L------SESDDVFDLPDNHPVRRHARLFTSILHISVKNV-------DELEAQVAPTVFKYGERHYRPD--------I-TPHMTEENVRVFCAQIVCTVFDFLR------------------------------------DTEATPKCAESWIELMRYLGQKLLDGFDFA----**

**>C06H2.5**

**HLSPHQVQLLTSTWPRIKT----------QSSLFTQVFKVLMQRSPVCREM----------FQKM-------SIVGGFSSNSVCDLNSHTKLLCELLDSLMTDL-------HQPAKIVLAKCQDVGAAHVNMNEKCCG---V--------VFDQLGEAFTELITKVEC-------------------------------------VRSKREAVKSWMCVISYMADSIKSGYMEE----**

**>C26C6.7**

**PLTCAQIHLVRALWRQVYT-------TKGPTVIGASIYHRLCFKN-VMVKEQ---------MKQV--------ELPPKFQNRDNFIKAHCKAVAELIDQVVENL--------DHLDNVTGELMRIGRVHAKVLRGE-----L-----TGKLWNTVAETIIDCTLEWGD------------------------------------RRCRSETVRKAWALIVAFVIEKIKAGHHEQ----**

**>F52A8.4**

**EPNVYEKELLRRTWSDEFD---------NLYELGSAIYCYIFDHNPNCKQL----------FPFI-------SKYQGDEWKESKEFRSQALKFVQTLAQVVKNI--------YHMERTESFLYMVGQKHVKFADRG-----F-----KHEYWDIFQDAMEFALEHRLS-------------------------------IMTDLDDNQKRDAVTVWRTLALYTTVHMRNGFIDG----**

**>T22C1.2**

**ILNSYQKSIVRNAWRHMSQ--------KGPSNCGSTITRRMMARKSTIGD-----------IL------------------DRSTLDYHNLQIVEFLQKVMQSL--------DEPDKISKLCQEIGQKHAKYRRSKGMK--I-------DYWDKLGEAITETIREYQG---------------------------------WKIHRESLRAATVLVSYVVDQLRFGYSRGLHVQ----**

**>C18C4.1**

**RIVDDDFELARTHWIQLQK-------SNKQGLAIRGCFLTMLEKYPQVRPIWG--------FGK---RIEGRGDETWKPEIVEDFYFRHHCASLQAALNMIIQN-------KDDKSGMRRMLNEMGAHHFFYDACEPH---F-------EVFQDSLLESMKLVLNGGD--------------------------------------SLDDDIEQSWICTSLRIPPGSFEYDNSK----**

**>R11H6.3**

**NLKHEHIRALKTTWARLCE------PPRANCKGIVSLVERVWEKL-DTKDKDVRNI-----FYNAAFVDSMHERCERRRSGSIATLRDHTHFFVSLVSQVVSSL-------EKEPAKILEHLDHIGQSHAYLKRYG-----F-----KSSHWEKVGEYFVDHVVIQDC-------------------------------------VRGFPEACRAWTVLVSSIVDRLRAAPRRG----**

**>C23H5.2**

**RFSQEEKDILRRSWKVLDK---------NLNHTAYNIFEMIFNQSPDTRQL----------FPFM----------KFNTGGRSKEIEFHALRFMQVLESVVKTL--------DNPETLNPLCDNLGRVHGRLSESRG----F-----RTHHWGVFIECTLFHFRKVLG------------------------------QDTYFHRMDALDKVIINWRIIIRLLIKQMKRGFNTD----**

**>T06A1.3**

**---MTSEKDNHIYLNDFPQ-----------NKDLYLKLKNVNAQT-VDMNCSDPG------F---------------------EAIAAQYLKVFDDVITAVEEK-------PGDVQTACDRLQAVGKMHRQK---------V--SGMDGTMFQNMEEPFIQMVSHILQ-------------------------------------DRFNEKAEMLYRKFFQFCLKYLLEGFNG-----**

**>Y17G7B.6**

**NLSVKQKKLLRQSFNAMNS-------GGTFLKLMEKIFRRLETKCPDMRSI----------FLT--TAFVNSLSRERQTPPLVKTEYDHCKCMVGIFERLIENL--------ENINEQLTMIRHYGEKHAQMAESG-----F-----TGAMIEQFGEISVFVIGSQDV-------------------------------------VKFNHETVKAWRLLLACVTDEMKVGFDRM----**

**>Y57G7A.9**

**RRPKLDIDRVRSVWMDHIN---------GNDQYFQEVIHRICKRN-EGIRCA---------MLAPNAQHAESVAEEDFVLSNIADRISQFFHQLVEDDVLMDTV------------ELKKACYDLGRQHSSYSKQQ-----F-----KMSYWEEFTLTMMGVLEQNYP--------------------------------------ETTKEEQKAWLHFLRFVNENMLDGYLDA----**

**>Y15E3A.2**

**TISPEHQKLIKRSWNRIPK--------AQFGRASLEAFITAAQV-THAI------------FVD------------------KETENRHVKYFVDLVQSCVDNL-------ENLETGVKPWLDLIGRGHANFK--------I-----TGKHWEKFGESLLTTATEWNG------------------------------------PGRRHKETVKAWMVMSSFLADRLAHASRLA----**

**>Y58A7A.6**

**GLSRDDKRIIETCWFKCSQ--------KQLRKCSCDMFWDILHTD-EDILRL---------FR-L-------DHVAPNRLKDNDYFKSHASNLALVLNLVVTNL-------QDNFDQAQDALQALGYQHLHL---------I-----DRTHFQTMYWDIFTDCFE-------------------------------------------RNPPPSFKKGAEREVALKFH----------**

**>C18C4.9**

**HLTQPQILFVRKTWNHARN--------QGALEPAISIFRNSFFKNPEIRQMIM--------FGT--------------KNEGHERLKKHAQLFTVLMDDLIANL--------DSPSATVAGLREAGEKHVWPTRNQ-----Y-GCPFHAHLLDQFATAMIERTLEWGE------------------------------------KKDRTETTQRGWTKIVLFVTEQLKEGFQDE----**

**>R90.5**

**GLSPYQQKLLVQCWPNIYT-------TGASGPFANSLYSTLSSRNAKAKELLAKADGVAV-FSK----------------SDFDCSVMHCRVTVEILDTVIKNL-------DNDHARITQYLTEIGKQHRHLKAEG-----L-----SSAVWDDLGDTIMDCARRRCE------------------------------------AVRKHKELRRAWLAIIAYIMDNLKQGQSMT----**

**>C36E8.2**

**ILSVNQRQIIKGCMDNSKD------------DLGERIFRRALERR-DDFKQ----------FI-------------------DNLSKGQRYENSQYLKQFLLGI-----ENIMDIDEINRISEEFGCNHVQFRANG-----F----KPDFFACTADAVTTECTFLDQA-------------------------------------AHPTSETAAAWSLLTSHVFSAVRDGYYAE----**

**>Y75B7AL.1**

**QLLGDRLSILKSSWEKANE--------MTNGEIGVRVAWNMVRKHPNLCKNDEPEK-----VSLL--------NGSCKRSIDHAKFQEIGGRITSFISELLELM-----QNNQPESYIVMRIRRVGAVHYDKG--------I---VFTSSVWKEFKHTIQTIISEVQF-----------------------------------SSPQEREAALDAWNIFISFIIREMKMGIWAI----**

**>F35B12.8**

**ICEQEEVNKMIESYQKIDD----------KYALFEQMFLTIFLEQ-EVEMAYS--------FG-L-------ENLNEQQLKVEQKFRTHVGKFQRFITGIIDML----SKGVESSDQIVEILRIVGRQHGNVRTMS-----F-----TAEKWLIFKNVLLDLLCKDA-----------------------------------------NEKVGATWNKLISFMISEVKDSYLEH----**

**>F21A3.6**

**RLSDRQRDVLQKTFAPILQ---------DCVRNGLKIFVRLFSEYPRYKLIWPQ-------F----------RAIPDSSLMNAVELRRHASVYLNGLGKIIDSM--------RDEEALGKSMSRIAVAHIKWN--------V-----QRNHVIHMIEPVLEVVKECNG-------------------------------------YQLDDETRQAWTVLYQVIADLIEVFRCRA----**

**>C06E4.7**

**NLSVAERQCICASWEKAST----------QSDIGCELVARLLNDN-RTRFRALLECKSGS-FLG-------SGNYTTEDVNGMKRARSVADGVNCFFNKVISKL-----MDANYVEEIQDLSLQLGAMHFRMK--------V---WFQAENWLCVKNCLLDSVVSALL---------------------KDTKGTYVICGGLKKVQTVEKHTTHAWFKFVQFIIQNMKKGFLAE----**

**>F56C4.3)**

**SFTQEEKNDLEHSWNLVEG---------KKNHIACDIYEMIFNQCPEARRL----------FPKL-------KFVGSKPDRKNNEFAFQAMRFMQVIEGAVKAL--------DHLTSLDVILDNLGRRHGKLEVNGK----F-----RSYYWSVFLECSIYCLRHAFS---------------------------------KRMNDKEVDHVIILWRYLLRDVMKKIKAGTTAD----**

**>R102.9**

**ELTTDEMQAVRDAWKRAKE-----------REIGKHILRALIERKPQ--------------FKDY---FGIHVDEKNHDVYSCREFQLQAHRIQNFLDTAVSSL----------------GFCPIGNIHQMAYRIGQ----I-------HFYRGVNFGADNWLTFKKV----------TVEIVTKDCGNSESSSMDLKSVPSLFPSSSSTVIIIGWEKFMSSVIREMKKGFLDE----**

**>C09H10.8**

**HDPQLLSDAISEAWLKSAE-------------LTPTWVWALVDL-PETMHATYKENAQ---FLNI--------------INQVRDFLNILIKVHKCDPEMIKTL---------------SFRLGARHRHYMNEGNDNC---F-----WAPFAQQLPIAMSKMYMRVVT--------------------EDSKIRRILrirsraaekseeeevceswrqfscmliesmkrgyegc----**

**>Y22D7AR.5**

**HLTPIDREILNKSWGIVSK---------DMQQVAVNIFQMIFEQAPDAKLM----------FSFM--------MKDYKEDKKSNEFIFHAVRFLQVIESTMTHL--------EDPAQLDAVFLNLGKIHAKHEEQ------L---GFSAHYWSVFKECVLFHFRKAMK-------------------------SHNKFHKRNEMSFAEIDSAIILWREVLRFIIDRMKVGYSES----**

**---------|--||--|----------------|--||--||---|---------------|--|--------------------|--||--||--|--||--------------------|--|---||-----------|---------|--|--|||-||---------------------------------------------------||--||--|---|-----------**
